# Alkyne Two-Phase Strategy: Rapid Generation of TK-285-Derived PROTACs as BRD4 Degraders

**DOI:** 10.1021/acs.jmedchem.5c03771

**Published:** 2026-03-19

**Authors:** Hiroyuki Yamakoshi, Ryo Watanabe, Ryosuke Segawa, Ryosuke Ishihara, Ryo Tachibana, Genki Kudo, Shota Nagasawa, Satoshi Yamanaka, Ayano Ito, Hiroyuki Takeda, Tatsuya Sawasaki, Ryunosuke Yoshino, Takatsugu Hirokawa, Takayuki Doi, Noriyasu Hirasawa, Yoshiharu Iwabuchi

**Affiliations:** † Graduate School of Pharmaceutical Sciences, 13101 Tohoku University, 6-3 Aoba, Aramaki, Aoba-ku, Sendai 980-8578, Japan; ‡ Department of Pharmaceutical Sciences, Tohoku University Hospital, Sendai 980-8574, Japan; § Graduate School of Pure and Applied Sciences, 13121 University of Tsukuba, 1-1-1 Tennodai, Tsukuba, Ibaraki 305-8577, Japan; ∥ Division of Proteo-Interactome, Proteo-Science Center, 12760 Ehime University, 3 Bunkyo-cho, Matsuyama, Ehime 790-8577, Japan; ⊥ Division of Proteo-Drug-Discovery Sciences, Proteo-Science Center, Ehime University, 3 Bunkyo-cho, Matsuyama, Ehime 790-8577, Japan; # Division of Cell-Free Sciences, Proteo-Science Center, Ehime University, 3 Bunkyo-cho, Matsuyama, Ehime 790-8577, Japan; ¶ Institute of Medicine, University of Tsukuba, 2 Amakubo, Tsukuba, Ibaraki 305-0005, Japan; ∇ Transborder Medical Research Center, University of Tsukuba, 1-1-1 Tennodai, Tsukuba, Ibaraki 305-8577, Japan

## Abstract

This study introduces a divergent synthetic strategy in linkerology using preassembled linkers to generate structural diversity. The approach was validated by developing bromodomain-containing protein 4 (BRD4)–targeting proteolysis-targeting chimeras (PROTACs) based on an “alkyne two-phase strategy,” employing the BRD4 inhibitor **TK-285** as the binding ligand. In the initial screening phase, alkyne-modified **TK-285** derivatives were subjected to click chemistry to optimize linker length and the modification site, leading to the identification of **TKP-5** as a potent degrader. **TKP-5** exhibited stronger thymic stromal lymphopoietin—more suppressive activity than **TK-285** and markedly suppressed *IL*-33 mRNA expression in a tape-stripping-induced skin injury model. In the subsequent optimization phase, late-stage diversification using 1,3-butadiyne-typed PROTAC intermediates revealed the critical contribution of the triazole moiety, supported by in silico analysis suggesting interaction with Trp81 of BRD4. The strategy is expected to be broadly applicable to modular functional molecules accessible via click chemistry.

## Graphical abstract

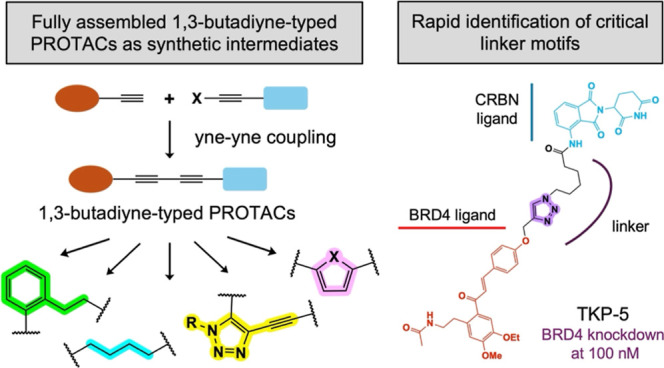

## Introduction

In the rapidly diversifying landscape of drug discovery modalities, molecules composed of multiple functional modules are being continuously developed. Modular architectures have become common across a wide spectrum of modalities, ranging from bivalent inhibitors^1^ and medium-sized molecules such as proteolysis-targeting chimeras (PROTACs)^2–6^ to large biomolecular constructs such as antibody–drug conjugates.^7^ At the core of these modular designs lies the linker, which plays a pivotal role not only in controlling the spatial arrangement of modules but also in modulating the physicochemical properties and ligand functions of the entire molecule. However, the design of linkers is not strictly constrained, allowing for vast structural diversity in terms of length, rigidity, hydrophobicity, and cyclic elements. This structural flexibility has inspired extensive efforts to establish rational strategies and methodologies for linker design. Indeed, the accumulation of such studies has given rise to a growing field, sometimes referred to as linkerology.^8–10^ Nevertheless, predicting how modular molecules interact simultaneously with multiple protein partners remains a major challenge. Therefore, the development of diversity-oriented synthetic methodologies that enable trial-and-error screening approaches is important. Particularly, for PROTACs, which represent a class of modular degraders, efficient and versatile linker construction remains a practical challenge. To address this, we herein present a new synthetic methodology, demonstrated through the development of PROTACs targeting bromodomain-containing protein 4 (BRD4).

PROTACs harness the ubiquitin–proteasome system to selectively eliminate disease-related proteins.^2–6^ Structurally, they comprise three essential components: a ligand for E3 ubiquitin ligase (E3), a ligand for the target protein (protein of interest; POI), and a linker connecting these two ligands. By simultaneously binding to E3 and POI, PROTACs bring them into proximity, facilitating the polyubiquitination of POI. This modification serves as a recognition signal for proteasomal degradation, ultimately leading to the elimination of POI.

PROTAC development requires prior identification of the target protein as well as structure–activity relationship information to appropriately install a linker with an E3 ligase, making this step a critical prerequisite for the entire process. This requirement distinguishes PROTACs from conventional small-molecule discovery, which can begin with phenotype-based screening without prior knowledge of the molecular target. Nevertheless, compounds initially discovered through phenotype-based approaches can still be adapted for PROTAC development once their targets are revealed. In this context, biotin probes are particularly useful, as they not only enable early target identification but also reveal ligand positions that tolerate modification. Identifying such modification-tolerant sites is highly advantageous as it can serve as a conjugation site for attaching an E3 ligand via a linker without disrupting target binding.

Following target identification, PROTAC development involves selecting a suitable ligand for the POI and conjugating it to an E3 ligand through a linker, a critical determinant of PROTAC performance. Linker design strategies in PROTACs often differ depending on the stage of development.^11^ In the early stages, relatively simple linkers, such as alkyl chains or polyethylene glycol (PEG) spacers, are typically employed, focusing primarily on optimizing linker length and attachment sites. PROTACs with flexible linkers tend to exhibit higher hit rates due to their enhanced conformational flexibility and the greater number of accessible low-energy conformations. As development progresses, more complex linkers, including cyclic and structurally rigid motifs, are explored for further optimization. At this stage, properties critical to drug-likeness characteristics, such as aqueous solubility, metabolic stability, and cell permeability, are given greater attention.

The structural prediction of ternary complexes involving protein–protein interactions remains a formidable challenge. Therefore, the identification and optimization of hit compounds often require the synthesis and evaluation of numerous derivatives through iterative trial-and-error processes. Efficient synthetic strategies for accommodating linker diversity are crucial for accelerating the development of PROTACs. Typically, PROTACs are synthesized by linking two ligands, either at the central region or at one terminus of the linker. Given the structural complexity of ligands, chemoselective and efficient conjugation methods are essential. Common approaches include amide bond formation, copper-catalyzed azide–alkyne cycloaddition (CuAAC),^12^ aromatic nucleophilic substitution, and reductive amination.^13,14^ For rapid library construction, bioorthogonal reactions, such as the Staudinger ligation, strain-promoted azide–alkyne cycloaddition, and inverse electron demand Diels–Alder reactions, are utilized.^12^ Advancements in these methodologies continue to drive diversity-oriented synthesis and functionalization of PROTACs. High-throughput screening systems for PROTACs have been established,^15,16^ but practical synthetic methods enabling rapid hit identification and structural diversity at the laboratory scale have also been developed. These include click chemistry,^17^ bifunctional orthogonal linkers,^18^ Ugi and Passerini multicomponent reactions,^19^ and solid-phase synthesis.^20^

Building on this background, we propose the “alkyne two-phase strategy” for the efficient and divergent synthesis of PROTACs (Figure 1). This strategy, employing readily available alkyne-modified POI ligands, consists of these two phases:

(1) Typical screening phase: determining linker length

**Figure 1:**
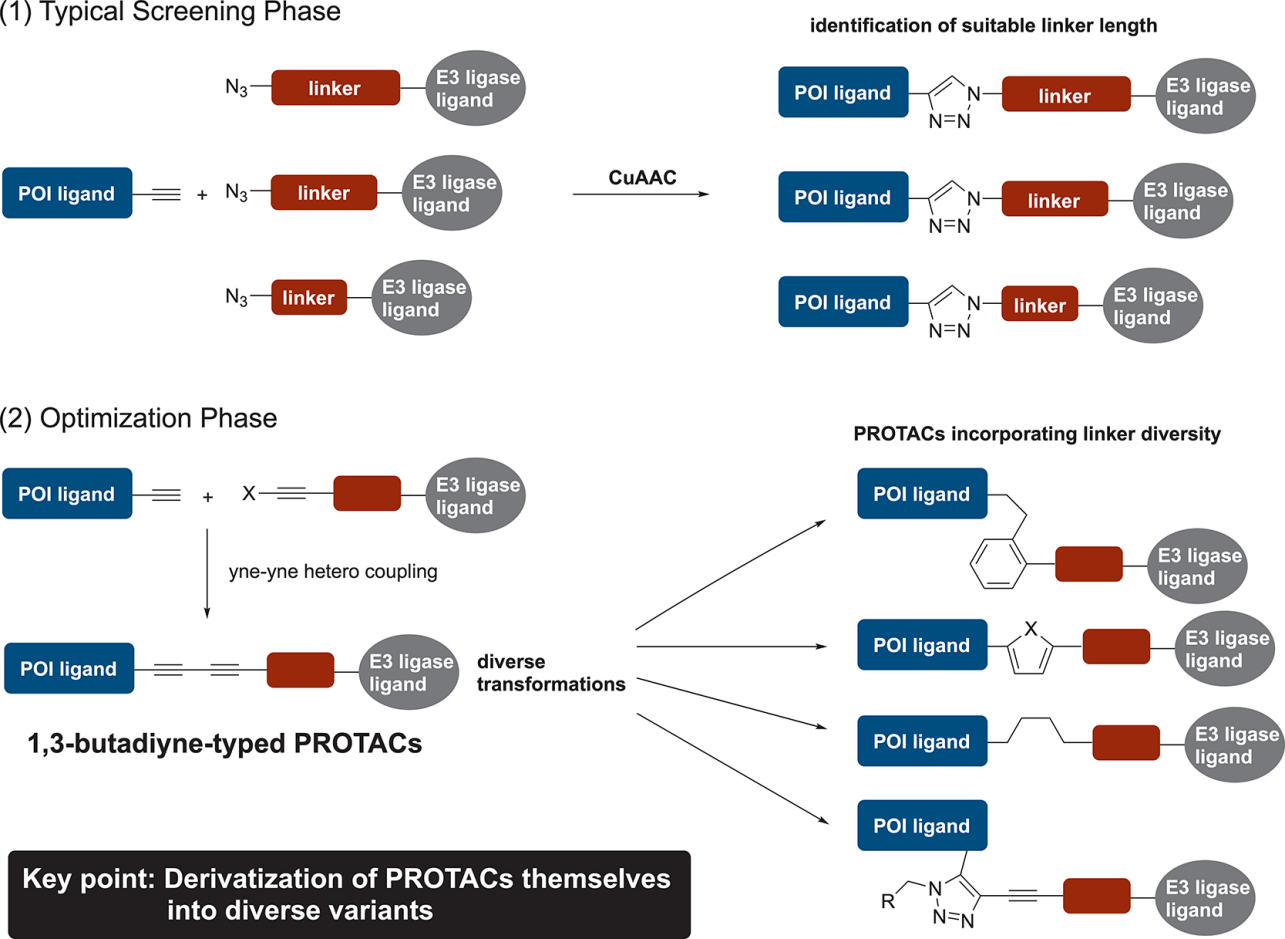
Alkyne two-phase strategy. (1) Typical screening phase employing click chemistry. (2) Optimization phase using 1,3-butadiyne intermediates that are PROTACs. POI, protein of interest; PROTACs: proteolysis-targeting chimeras.

CuAAC is utilized to generate derivatives with various modification sites and linker lengths, allowing the identification of hit compounds.

(2) Optimization phase with 1,3-butadiyne-typed PROTACs

Hit compounds guide the selection of 1,3-butadiyne intermediates with matching linker lengths. These intermediates are synthesized using the alkyne-modified compounds generated during the typical screening phase and serve as a platform for producing PROTACs with diverse linkers. Furthermore, 1,3-butadiyne can be prepared via yne–yne heterocoupling^21,22^ and transformed into various structures, including heterocycles, benzene rings, hydrocarbons, and branched chains.

In all reported diversity-oriented syntheses of PROTACs to date, structural diversity is introduced at the step of linking the ligands and linker, or even earlier.^17–20^ In contrast, the optimization phase proposed by us is different because it uses fully formed PROTACs (1,3-butadiyne) after the ligands and linker have been conjugated. In conventional approaches, modification of linker structures generally requires changes in the connection sites or linking methods. In contrast, the divergent transformation of the 1,3-butadiyne unit enables systematic variation of linker structures without altering the connection sites or overall assembly strategy. Consequently, this approach facilitates efficient midstage structural optimization and accelerates the acquisition of structure–activity relationships for PROTACs.

As a demonstration of the “alkyne two-phase Strategy,” we selected BRD4 as a biologically relevant target to explore the synthesis of PROTACs using **TK-285**, a ligand identified previously by one of our collaborators (Figure 2).^23^

**Figure 2:**
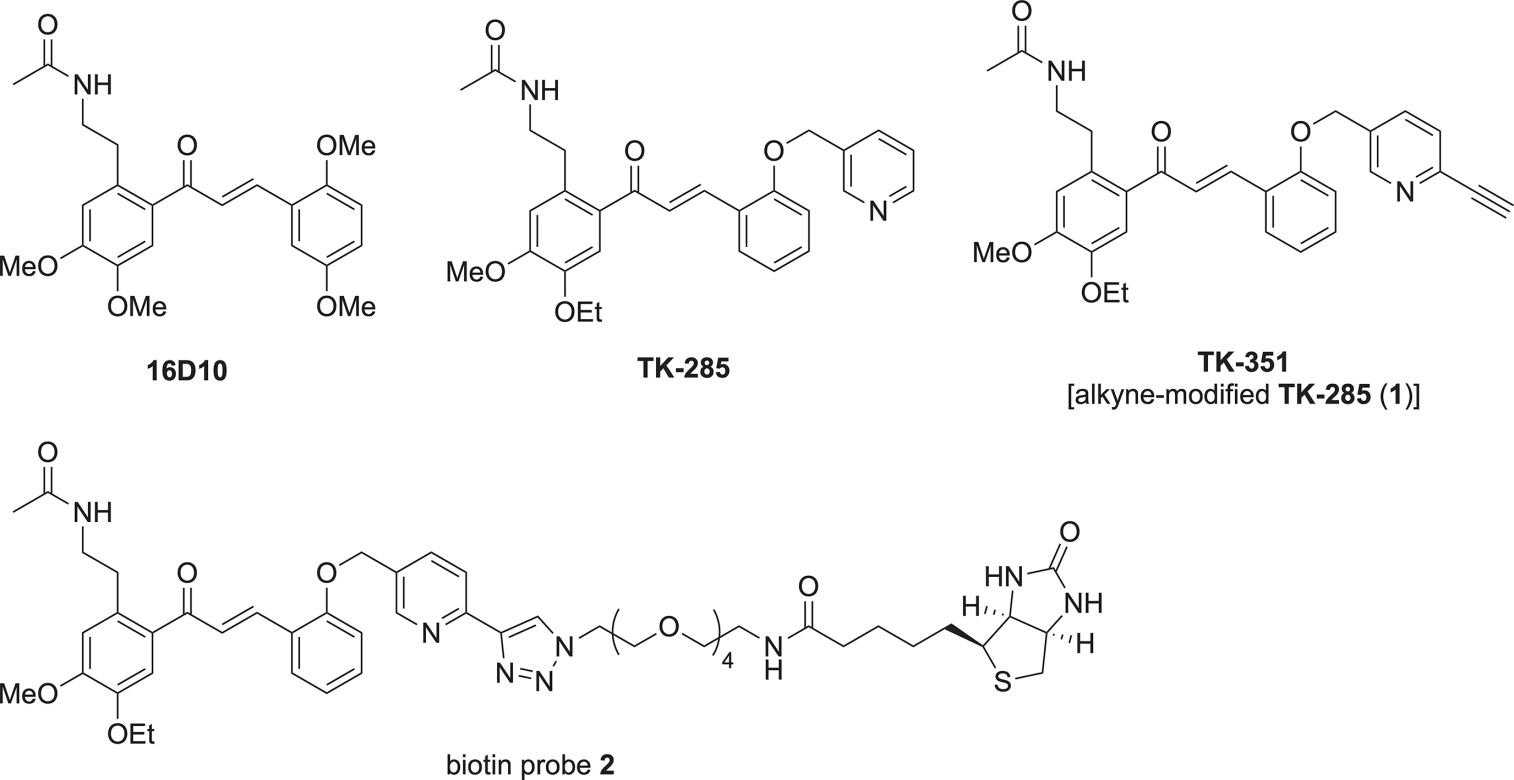
Structures of the TSLP production inhibitors and the corresponding biotin probe. TSLP, thymic stromal lymphopoietin.

**TK-285** was derived from the hit compound **16D10**, which was identified through a screening study using a keratinocyte cell line (KCMH-1) that constitutively produces thymic stromal lymphopoietin (TSLP), a key cytokine involved in inflammatory signaling in keratinocytes. This screening was conducted in the context of atopic dermatitis, aiming to discover inhibitors of TSLP production. Subsequent derivatization of **16D10** led to the development of **TK-285**, a potent inhibitor of TSLP production. To identify the target molecules of **TK-285**, we synthesized biotin probe **2** from **TK-351** [the alkyne-modified derivative of **TK-285** (**1**)] and performed an AlphaScreen assay, which revealed that the bromodomain and extra-terminal domain (BET) family proteins BRD2–4 are the molecular targets of **TK-285**.^23^ Isothermal titration calorimetry revealed a strong binding affinity between **TK-285** and the BD1 domain of BRD4, with a dissociation constant (*K*_d_) of 190 nM. Furthermore, X-ray crystallographic analysis of the BRD4–**16D10** complex demonstrated that the acetamide group of **16D10** mimics acetylated lysine residues on histones, which are recognized by BET family proteins.

BRD4 has been extensively investigated as a therapeutic target in oncology^24^ and has also been implicated in the regulation of inflammatory gene expression, including the exacerbation of allergic inflammation through enhanced IL-9 production and Th9 cell differentiation.^25,26^ We found that **TK-285** inhibits TSLP production by preventing BRD4 from binding to the TSLP promoter region, demonstrating its mechanistic relevance in keratinocytes.^23^ While systemic BRD4 targeting may raise concerns regarding potential adverse effects, such issues are less relevant in the context of keratinocyte-targeted topical interventions. Indeed, several representative BRD4-targeting PROTACs, such as dBET1 and MZ1, have been reported, primarily in oncological contexts. Despite the competitive landscape in the development of BRD4 inhibitors and degraders, our study represents the only reported effort to focus on keratinocyte-specific anti-inflammatory applications.^23,27^

Taken together, these findings motivated us to develop PROTACs employing **TK-285** as a BRD4 ligand, serving as a model system to demonstrate the feasibility of the “alkyne two-phase strategy” for efficient optimization of modular molecules.

## Results and Discussion

### Typical Screening Phase: Design, Synthesis, and Biological Evaluations

Based on the X-ray crystallographic analysis of **16D10** in complex with the BD1 domain of BRD4,^23^ we designed several alkyne-modified BRD4 ligands for use in the CuAAC reaction (Figure 3). The core scaffold of 16D10 (outlined with a gray line) represents the key binding region, with the acetamide group acting as a mimic of acetylated lysine recognized by BRD4. In contrast, the other benzene ring, marked as the site for alkyne modification, does not contribute significantly to binding. Therefore, we designed **TK-351** by introducing the alkyne moiety at the same benzene ring position as in the synthesis of biotin probe **2**, ensuring compatibility with PROTAC conjugation. Considering the formation of a ternary complex, the structural moiety around the alkyne, which interacts with the E3 ligase ligand, may exhibit structure–activity relationships distinct from those observed for BRD4 selectivity alone. To address this, we designed additional alkyne-modified BRD4 ligands by modifying the substitution pattern of the benzene ring, altering the structure of the double bond, and removing the pyridine moiety. Regarding the E3 ligand, pomalidomide was selected because of its reliability and widespread use, with the corresponding E3 ligase being cereblon (CRBN).

**Figure 3:**
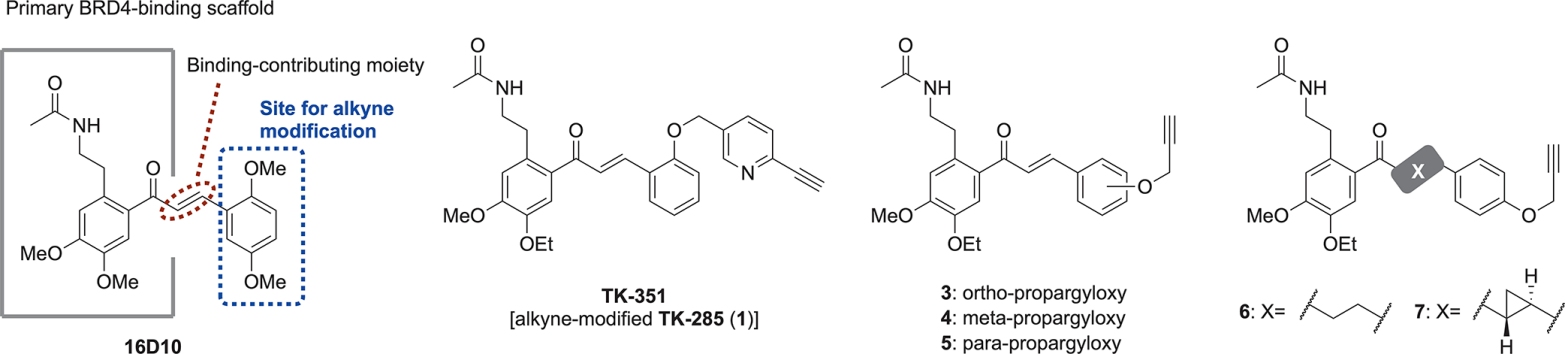
Design of alkyne-modified **TK-285** analogs.

To access **TK-351**, aldehyde **11** was obtained through the bromination of pyridylmethanol **8**, followed by a Sonogashira coupling with tetramethylsilane (TMS)–acetylene (Scheme 1). Commercially available aldehyde **12** underwent a three-step sequence, including a Henry reaction, LiAlH_4_ reduction, and *N*-acetylation, and was subsequently converted to methyl ketone **14** via Friedel–Crafts acylation. In this step, caution is required as excessive use of AlCl_3_ can lead to undesired cleavage of the ethyl ether. The aldol condensation of aldehyde **11** and ketone **14**, accompanied by TMS removal, yielded **TK**-**351**, with a 69% yield. Ketone **14** was subjected to aldol condensation with three known aldehydes **15**–**17**, affording the corresponding alkyne-modified chalcone derivatives **3**–**5**, with yields of 88%, 93%, and 93%, respectively. Similarly, using aldehyde **18** in the aldol reaction afforded chalcone **19**, with a 91% yield. Subsequent hydrogenation and removal of the ethoxyethyl group led to the formation of phenol **20** (69% yield) over two steps. Propargylation yielded derivative **6**, which possesses a reduced enone moiety. Additionally, cyclopropanation of chalcone **5** produced the *trans*-cyclopropane **7**, with 80% yield.

**Scheme 1:**
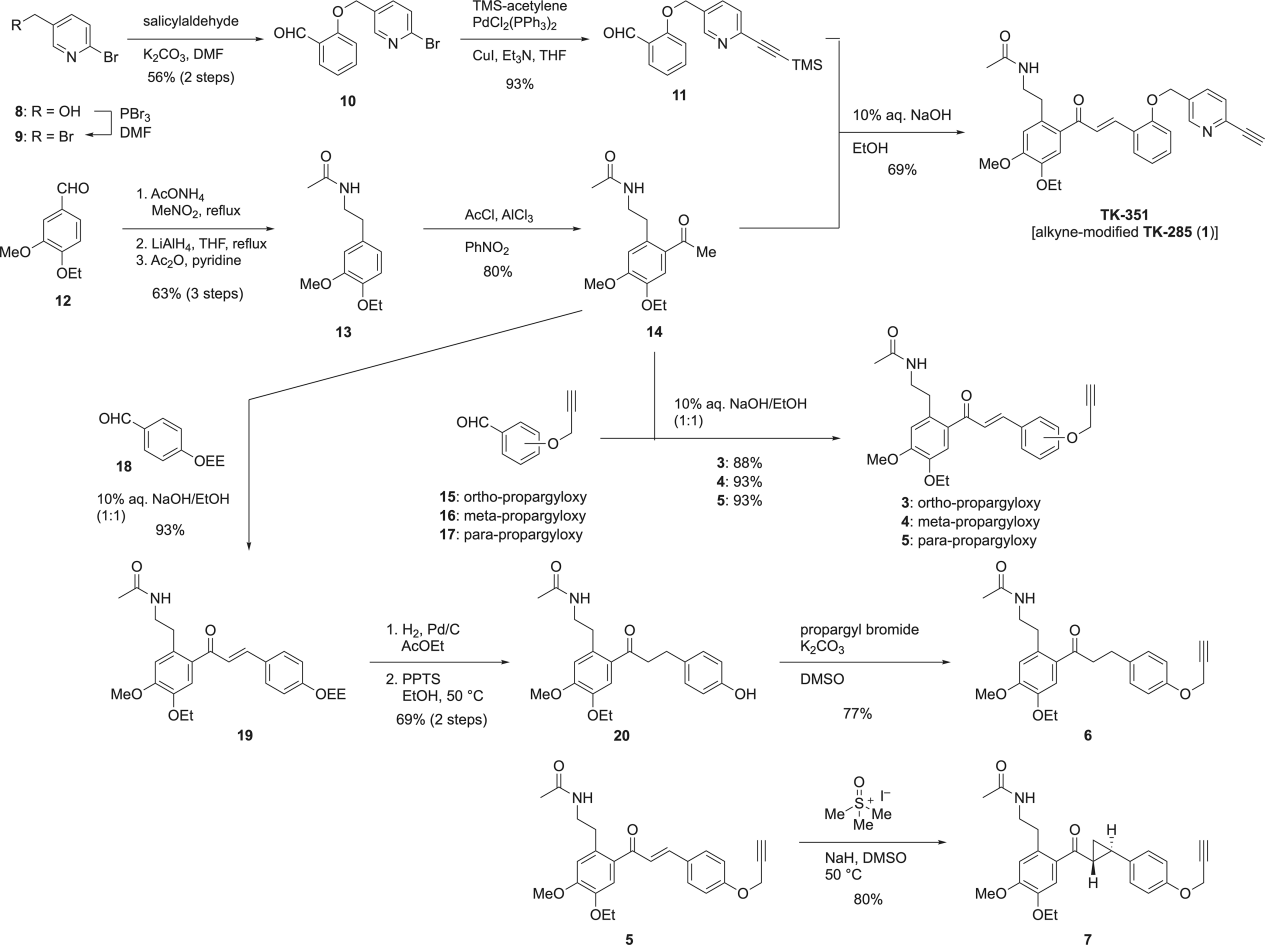
Synthesis of Alkyne-Modified **TK-285** Analogs

The synthesis of azide-modified pomalidomide derivatives **27**–**32**, incorporating a series of linkers of varying lengths, is outlined in Scheme 2. Pomalidomide was first acylated with a halogenated acid chloride **21**–**26**, followed by a nucleophilic substitution with sodium azide to generate the corresponding azide-modified E3 ligand. Additionally, the *N*-methylated derivative of pomalidomide, known as a negative control because of its inability to bind CRBN, underwent the same transformation to afford *N*-methyl derivative **32** from *N*-methyl pomalidomide.

**Scheme 2:**
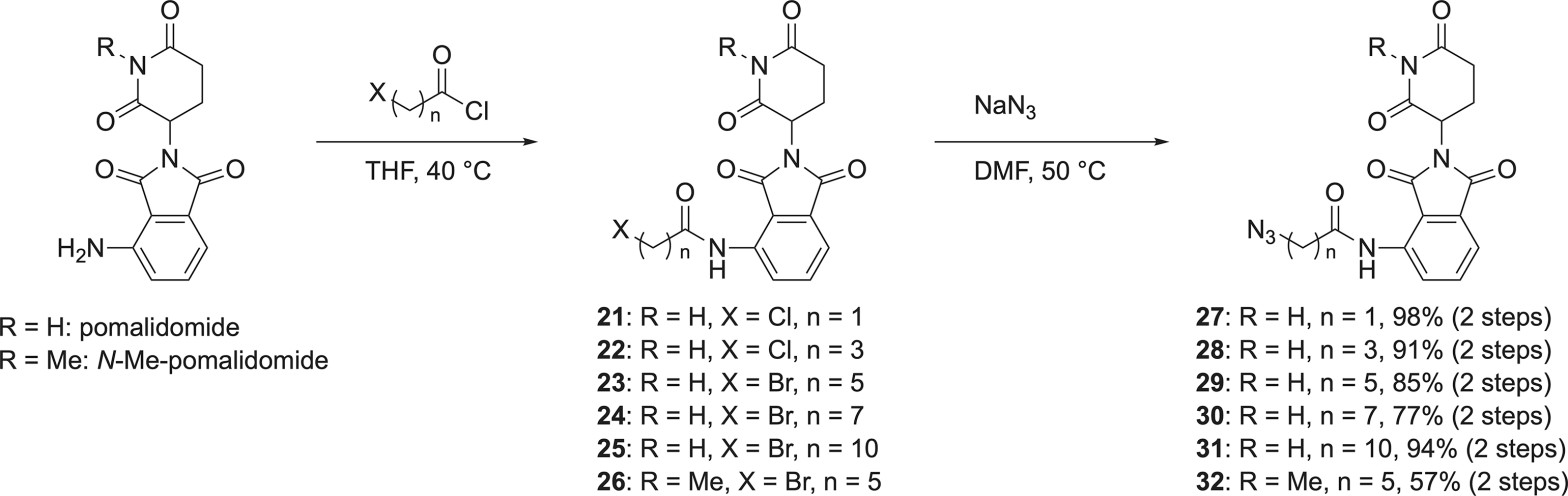
Preparations of Azide-Modified Pomalidomides

Sixteen PROTACs, collectively termed TKPs (**TK-285**-based PROTACs), were synthesized by conjugating alkyne-modified BRD4 ligands **1** and **3**–**7** with azide-modified CRBN ligands **27**–**32** and **40** via a CuAAC reaction employing copper­(I) 2-thiophenecarboxylate (Scheme 3). Western blot analysis was performed using mouse keratinocyte KCMH-1 cells to evaluate the BRD4 degradation-inducing activity of the TKP-compounds (Figure 4 and Table S1). BRD4 expression levels after 24 h treatment with 10 μM compounds were compared using dimethyl sulfoxide (DMSO) vehicle as a control. Of the two bands corresponding to BRD4, the ∼200 kDa band represents BRD4-L (long isoform), whereas the ∼120 kDa band corresponds to BRD4-S (short isoform). BRD4 expression levels were quantified using BRD4-S, which exhibited better reproducibility in Western blotting. Additionally, representative results of the Western blot analysis are provided in the Supporting Information (Figure S1).

**Scheme 3:**
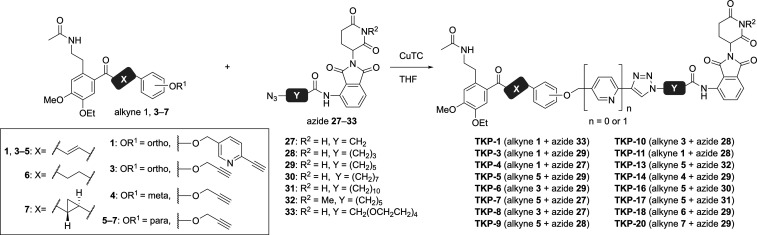
Synthesis of TKP-Compounds via the CuAAC Reaction^a^

a CuAAC, copper-catalyzed azide–alkyne cycloaddition.

**Figure 4:**
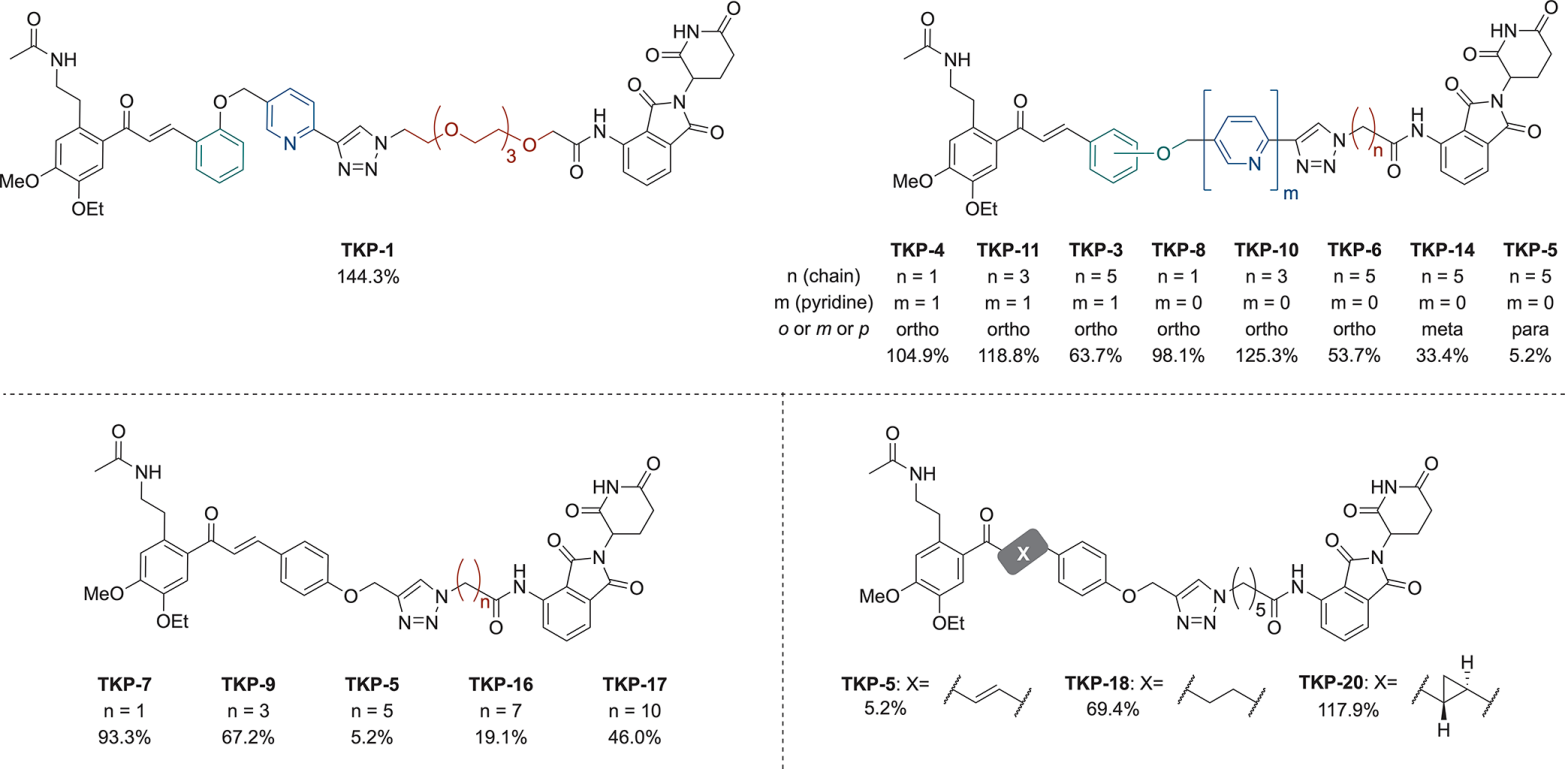
BRD4 degradation after treatment with TKP-compounds. Western blot analysis of BRD4 degradation induced by 10 μM TKP compounds in the mouse keratinocyte cell line KCMH-1 after 24 h treatment. BRD4 levels are reported as a percentage of BRD4 band densitometry relative to the DMSO vehicle. Western blot results are representative of three (*n* = 3) independent experiments. DMSO, dimethyl sulfoxide.

This analysis successfully identified **TKP-5** as a highly potent compound, with several other derivatives also exhibiting activity (Figure 4). **TKP-1**, in which pomalidomide was conjugated to **TK-285** via a PEG linker, showed no degradation activity (144.3%). When the PEG linker was replaced with an aliphatic chain and derivatives with varying chain lengths were compared (**TKP-4**, **11**, **3**), **TKP-3** with an *n* = 5 linker exhibited degradation-inducing activity. Based on a comparison of analogs bearing the same aliphatic linker but lacking the pyridine moiety, **TKP-6** with an *n* = 5 linker also exhibited degradation-inducing activity, with slightly enhanced potency upon pyridine removal. These findings suggest that, although the presence of a pyridine ring was beneficial in the development of **TK-285**, it negatively affects the degradation-inducing activity. The triazole moiety may partially substitute for pyridine, and the coexistence of both could reduce the activity. The substitution pattern on the benzene ring of the BRD4 ligand showed that para-substitution (**TKP-5**) was the most effective, followed by meta-substitution (**TKP-14**), with ortho-substitution (**TKP-6**) exhibiting the weakest activity. *K*_d_ values of these three compounds with BRD4-BD1 were evaluated using isothermal titration calorimetry. The *K*_d_ values were 150 nM for **TKP-5**, 150 nM for **TKP-6**, and 100 nM for **TKP-14** (Figure S2). The discrepancy between binding strength and degradation-inducing activity is a characteristic trend of PROTACs, underscoring the importance of reexploring the structure around the linker-introducing site of the ligand.

Focusing on the structure of **TKP-5**, the linker length was compared. The number of atoms in the alkyl linker (*n*) for **TKP-7**, **9**, **5**, **16**, and **17** is 1, 3, 5, 7, and 10, respectively. Values of *n* shorter or longer than that of **TKP-5** (*n* = 5) led to a gradual decrease in activity. Based on these results, the optimal linker length was determined to be 11 atoms, which corresponds to *n* = 5. Here, the linker length was defined as the shortest number of atoms from the oxygen atom substituted on the benzene ring of the BRD4 ligand to the carbonyl carbon of the acylated pomalidomide. Furthermore, the impact of the enone moiety in the BRD4 ligand was examined. **TKP-18**, where the enone double bond is reduced, showed weak activity, whereas cyclopropane **TKP-20** showed no activity. These results suggest that planarity in this region is crucial for activity.

To investigate whether BRD4 degradation induced by **TKP-5** occurs through CRBN-mediated polyubiquitination and subsequent proteasomal degradation, we conducted further analyses. **TKP-13**, the *N*-methylated derivative of **TKP-5**, exhibited negligible activity (Table S1), confirming the importance of CRBN binding. Furthermore, the BRD4 degradation induced by both **TKP-5** and the commercially available PROTAC MZ-1 was abolished in the presence of the proteasome inhibitor MG-132, verifying that degradation proceeds through this pathway (Figure S3). Next, the concentration and time required for **TKP-5** to exert its effect were examined (Figure 5). The effect of **TKP-5** was detected immediately after treatment, with BRD4 expression almost completely suppressed within 2 h (Figure 5a). Furthermore, BRD4 degradation-inducing activity was observed at concentrations as low as approximately 100 nM (Figure 5b,c). Consistent with the decrease in BRD4 levels, **TKP-5** also showed a marked inhibitory effect on TSLP expression at 100 nM (Figure 6a). However, **TK-285** and **TK-351** displayed little to only very weak inhibition at the same concentration, indicating that the expected potency enhancement of the PROTACs was successfully achieved. Furthermore, in a tape-stripping–induced skin injury mouse model, treatment with 1% **TKP-5** ointment for 6 h completely suppressed the skin injury-induced IL-33 mRNA expression, exhibiting a significantly stronger anti-inflammatory effect than 1.2% TK-351 ointment (Figure 6b).

**Figure 5:**
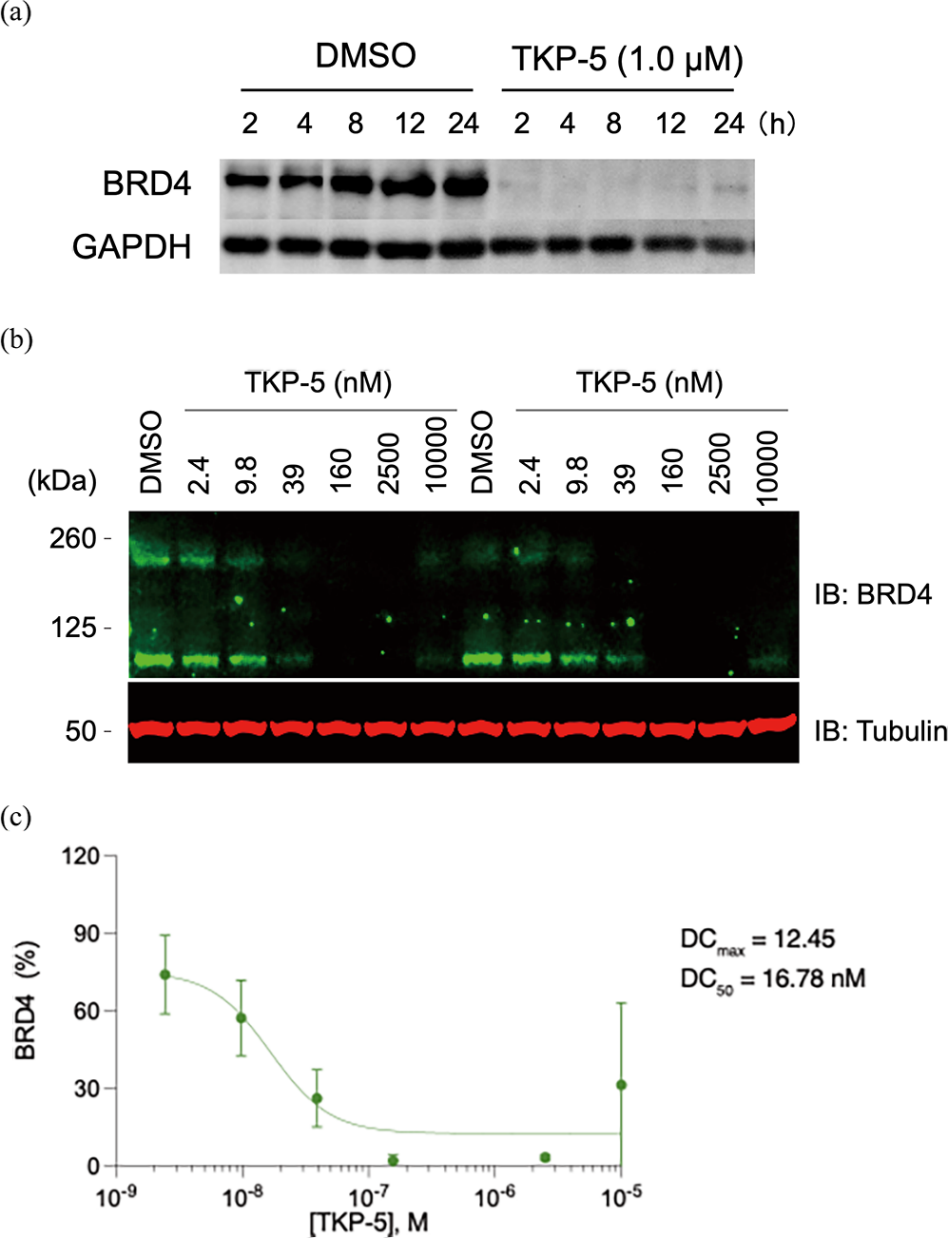
Western blot analysis of BRD4 degradation induced by **TKP-5** in the mouse keratinocyte cell line KCMH-1. (a) Time-dependent degradation after treatment with 1 μM **TKP-5**. (b) Dose-dependent degradation upon varying the concentration of **TKP-5** for 5 h. (c) Quantification of BRD4 protein levels shown in (b). DMSO, dimethyl sulfoxide.

**Figure 6:**
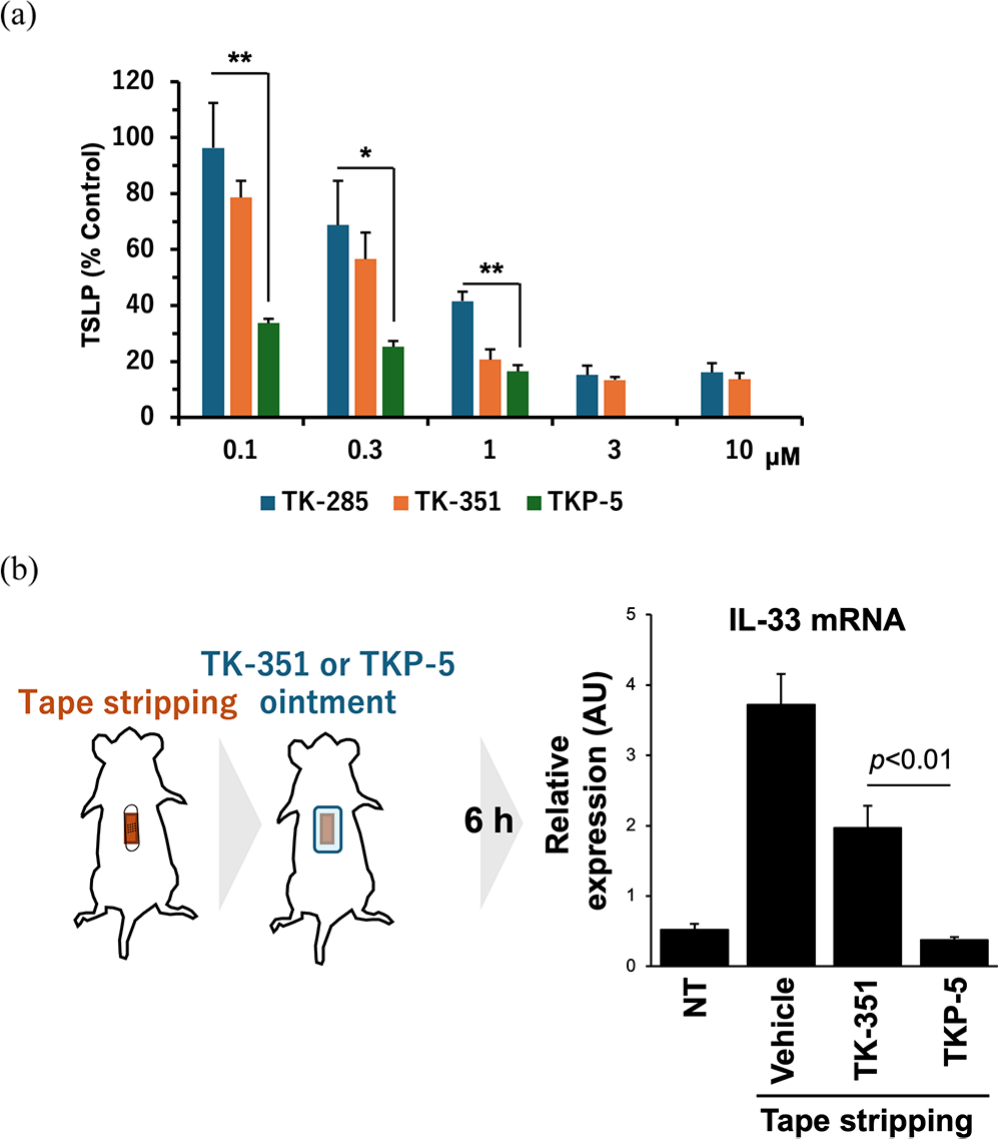
Comparison of the activities of the non-PROTAC inhibitors (**TK-285**, **TK-351**) and **TKP-5**. Statistical significance was determined using a two-sided Student’s *t*-test assuming unequal variances. Groups compared for each significance are indicated in each panel of the figure. (a) Concentration-dependent inhibition of TSLP production after 24 h treatment of KCMH cells with the compounds. (b) In vivo anti-inflammatory activity after 6 h treatment with 1% **TKP-5** or 1.2% **TK-351** ointment in the tape-stripping skin injury model. PROTAC, proteolysis-targeting chimera; TSLP, thymic stromal lymphopoietin.

The structure–activity relationship of **TKP-5**, identified during the typical initial screening phase, is summarized below. Active compounds tended to possess (i) an enone functional group and a para-substituted benzene ring in the BRD4 ligand, (ii) the absence of a pyridine ring in the linker region, and (iii) an optimal linker length of 11 atoms (Figure 7).

**Figure 7:**
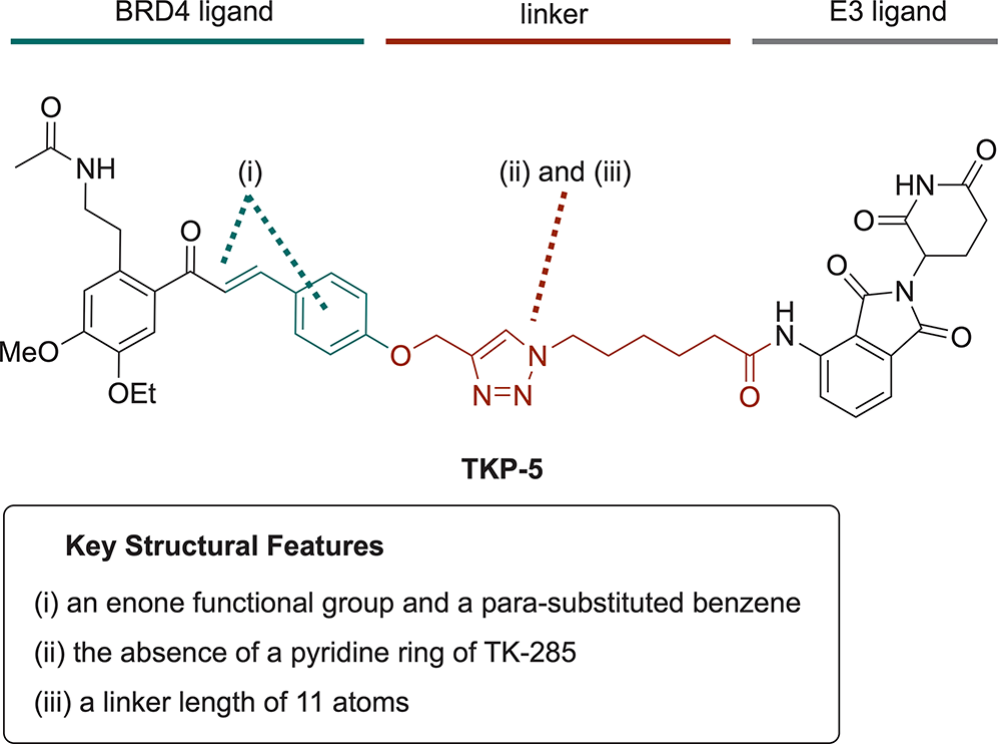
Early stage structure–activity relationships of **TKP-5**.

### Optimization Phase: Linker-Focused Structure–Activity Relationship via Divergent Synthesis

To explore the utility of 1,3-butadiyne-based linkers for divergent transformations, we designed PROTACs **TKP-21** and **TKP-30**, which incorporate alkoxymethyl-substituted and aromatic-conjugated 1,3-butadiyne units, respectively (Schemes 4 and 5). The ether oxygen in **TKP-21** and the aromatic ring in **TKP-30** were expected to contribute to the control of site-selectivity through diverse transformation processes. A linker length of 12 atoms, slightly longer than that of **TKP-5** (11 atoms), was selected to accommodate a possible shortening caused by the conversion of the linear 1,3-butadiyne unit.

**Scheme 4:**
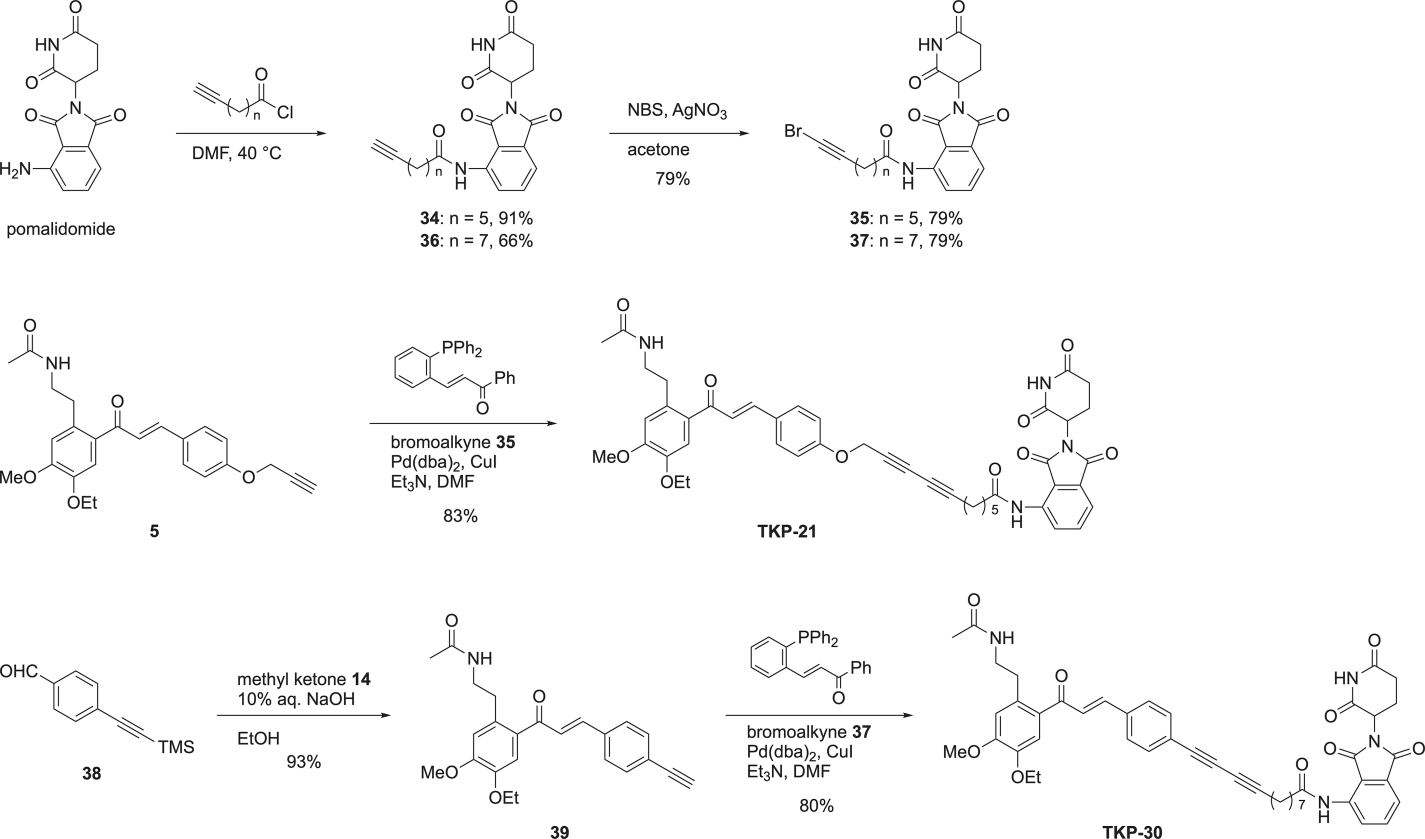
Synthesis of 1,3-Butadiyne-Typed PROTACs^a^

a PROTAC, proteolysis-targeting chimera.

**Scheme 5:**
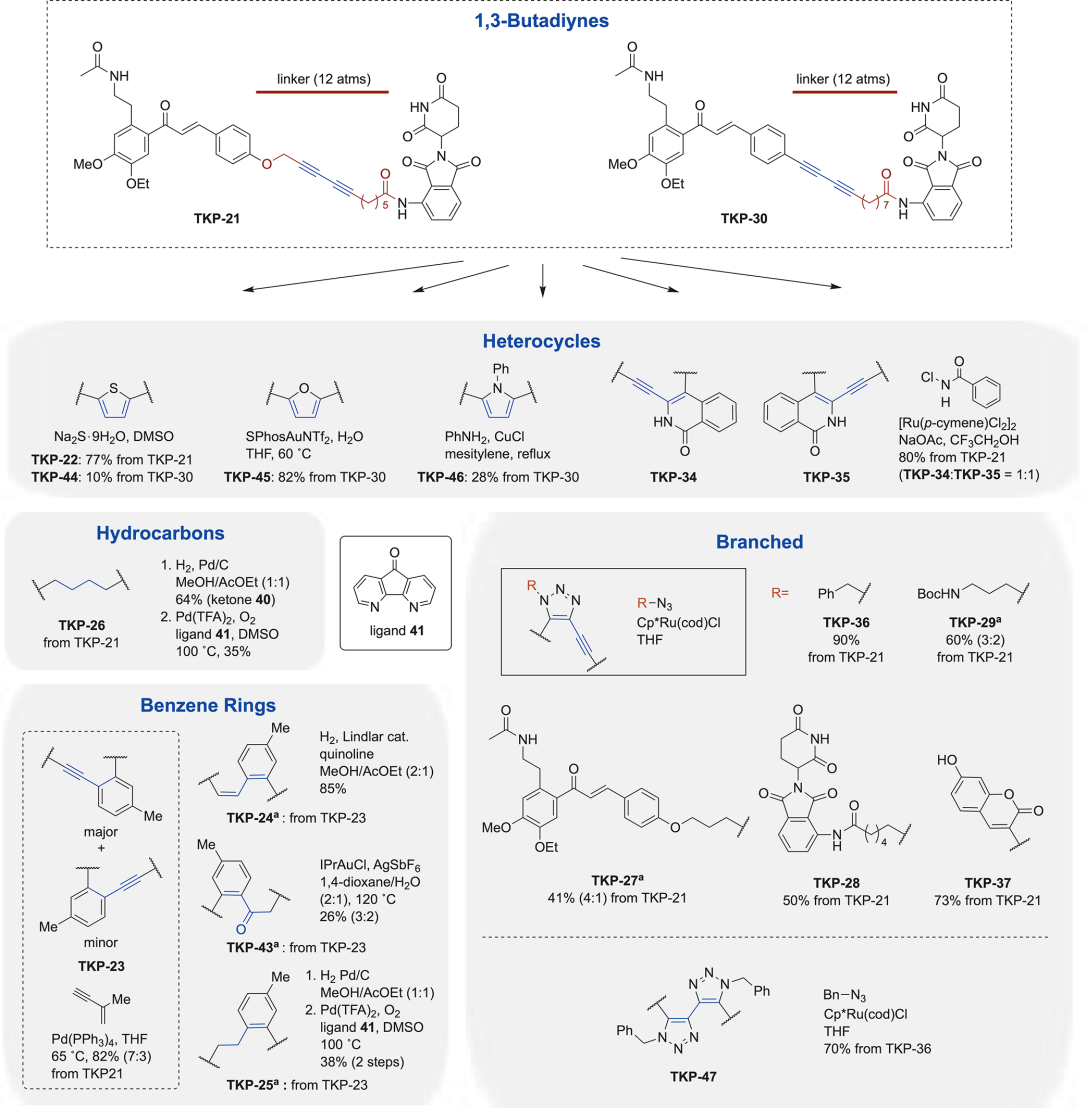
PROTAC Derivatization Enabled by Diverse Transformations of 1,3-Butadiyne-Typed PROTACs^a^

a PROTAC, proteolysis-targeting chimera.

The 1,3-butadiyne **TKP-21** was readily synthesized via a straightforward Cadiot–Chodkiewicz coupling,^21,22^ demonstrating the practical ease of its preparation (Scheme 4). The reaction employed an alkyne-modified BRD4 ligand **5**, which had been used in the initial screening phase. Notably, this ligand could be effectively repurposed, highlighting its applicability beyond the screening stage. The bromoalkyne **35**, derived from pomalidomide, was easily obtained by condensation with an alkynyl acid chloride, followed by bromination. Under the conditions reported by Lei, the Cadiot–Chodkiewicz coupling afforded **TKP-21**, with 83% yield, while effectively suppressing the undesired homocoupling; this is likely due to accelerated reductive elimination facilitated by the electron-deficient alkene in the phosphine ligand.^21^ Similarly, **TKP-30** was synthesized from aldehyde **38** via aldol condensation followed by coupling with bromoalkyne **37**.

Subsequently, divergent transformations of 1,3-butadiyne were performed using **TKP-21** and **TKP-30** (Scheme 5 and S1). Upon treatment with sodium sulfide, thiophenes **TKP-22** and **TKP-44** were obtained from **TKP-21** and **TKP-30**, with 77% and 10% yields, respectively.^28^ Concerns about functional group compatibility arose from the enone moiety in the BRD4 ligand. However, high-dilution conditions effectively minimized oligomerization caused by excess reagents. Gold-catalyzed furan formation afforded **TKP-45**, with 82% yield.^29^ However, when the same transformation was applied to **TKP-21**, it failed to provide the desired product because of cleavage of the propargyl ether. Copper-catalyzed pyrrole formation from aniline and **TKP-30** afforded **TKP-46**, with 28% yield.^30^ Furthermore, isoquinolone scaffolds were constructed, leading to **TKP-34** and **TKP-35**, which incorporate bicyclic heteroarenes as linkers.^31^ In the Rh-catalyzed construction of isoquinolones, insertion preferentially occurs into electron-rich alkynes. The ether substituent has been reported to act as a directing group, with regioselectivity governed by steric effects.^32,33^ However, such a directing effect is negligible under Ru-catalyzed conditions. To access both regioisomers, we employed nonselective conditions. Overall, four types of heteroaromatic frameworks were successfully constructed.

In the reduction to the corresponding alkane, concomitant reduction of the enone moiety could not be avoided. Therefore, **TKP-21** was first subjected to Pd/C-catalyzed hydrogenation, after which the reduced enone was reoxidized using the method reported by Diao et al.^34^ Although a larger excess of reagents was required than in the original report, the desired saturated derivative **TKP-26** was successfully obtained.

The benzene ring was constructed via the palladium-catalyzed [4 + 2] cross-benzannulation reported by Yamamoto,^35^ affording **TKP-23** as a 7:3 mixture of isomers, with 82% yield, from **TKP-21**. Electronic deactivation of the oxygen-adjacent alkyne via inductive effects favored migratory insertion at the other alkyne, resulting in the major isomer. Subjecting the isomeric mixture of **TKP-23** to Lindlar reduction furnished the *Z*-alkene **TKP-24** with an isomer ratio consistent with that of the starting material.^36^ Hydration of **TKP-23** resulted in cleavage of the propargyl ether for the major isomer, while the minor isomer afforded ketones as a mixture of carbonyl regioisomers (3:2), with 26% yield.^37^ Catalytic hydrogenation of **TKP-23**, followed by reoxidation of the BRD4 ligand, yielded **TKP-25**.^34^

Ruthenium-catalyzed azide–alkyne cycloaddition (RuAAC) reactions enabled the synthesis of PROTACs with branched linkers.^38,39^ Using the corresponding azide compounds, a variety of derivatives were obtained in good to excellent yields, including benzene-containing **TKP-36** (90%), the protected amine **TKP-29** (available for subsequent modification; 60%), BRD4- or E3-ligand-bearing compounds **TKP-27** (41%) and **TKP-28** (50%), as well as the fluorescent probe **TKP-37** (73%). In this transformation, the ether oxygen atom of **TKP-21** served as a directing group, thereby contributing to the observed regioselectivity.^40^ Notably, subjecting **TKP-36** to a second RuAAC reaction enabled the synthesis of **TKP-47**, featuring a four-branched linker, further demonstrating the versatility of this approach.

The BRD4 degradation activities of the synthesized derivatives are summarized in Figure 8 and Table S2. Compounds were tested at 1 μM for 24 h, and those that reduced BRD4 expression to below 30% were further evaluated at 0.1 μM. The intermediate 1,3-butadiyne-typed PROTAC **TKP-21** showed clear degradation at 1 μM, although less potent than **TKP-5**. All 1,3-butadiyne-derived compounds induced BRD4 degradation at 1 μM, with residual expression ranging from 0.91% to 77%. Among them, **TKP-22**, **27**, **29**, **36**, **43**, and **47** (indicated in brown) showed particularly strong activity. Notably, all of these shared a common feature: an aromatic ring (thiophene, triazole, or benzene) at the same position as the triazole of **TKP-5**. At 0.1 μM, **TKP-5** displayed the strongest degradation activity (45%), closely followed by the thiophene analog **TKP-22** (57%). Although the triazole was not strictly required, compounds lacking it generally showed weaker activity, indicating that the triazole within the linker of **TKP-5** makes an important contribution to its degradation potency.

**Figure 8:**
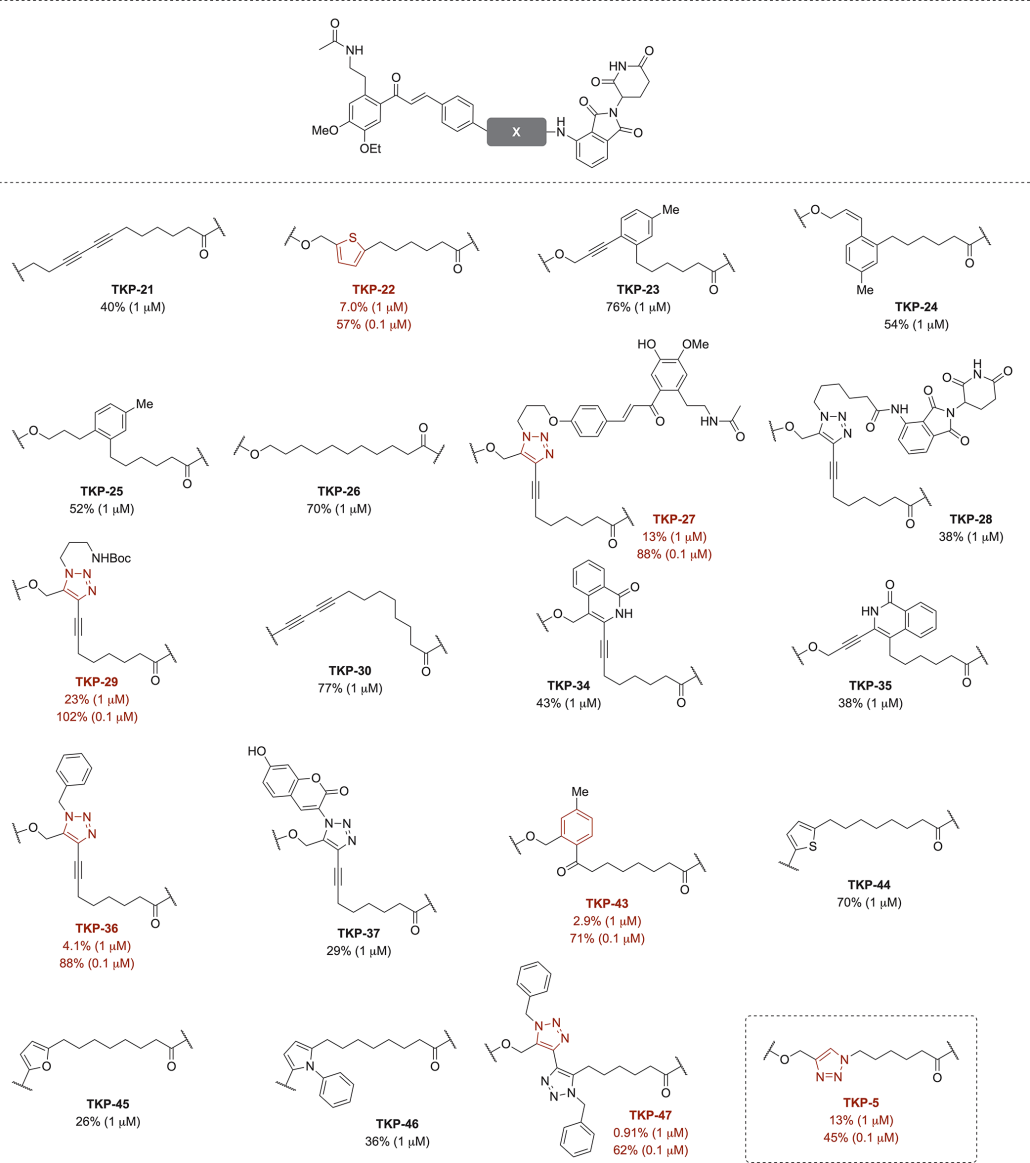
BRD4 degradation after treatment with TKP-compounds. Western blot analysis of BRD4 degradation induced by 1 or 0.1 μM TKP compounds in the mouse keratinocyte cell line KCMH-1 after 24 h treatment. BRD4 levels are reported as percentages of BRD4 band densitometry relative to the DMSO vehicle. Western blot results are representative of three (*n* = 3) independent experiments, except for **TKP-5** (*n* = 6).

To examine the importance of the triazole ring in **TKP-5**, **TKP-31** and **TKP-32** were synthesized (Scheme S2). In these derivatives, the triazole ring was shifted to different positions. For **TKP-31**, the structural modification resulted in the loss of the oxygen atom; therefore, as a control, **TKP-33** was synthesized by replacing the oxygen atom of **TKP-5** with a methylene group. Comparison of activities revealed that only **TKP-33** retained potency comparable to **TKP-5**, confirming the critical role of the triazole ring at its specific position (Figure S4).

### In Silico Analysis: the Importance of the Triazole Ring in the Ternary Complex

Based on the structural information on the DDB1B–CRBN–dBET23–BRD4BD1 complex available in the Protein Data Bank (6BN7, PDB),^41^ we modeled the CRBN–TKPs–BRD4 ternary complexes using **TKP-5** and its analogs **TKP-31–33**. These complex structures were then evaluated by molecular dynamics (MD) simulations (see the Experimental Procedures section in Supporting Information). The ligand root-mean-square deviation (RMSD) profiles indicate that the ternary complexes formed with **TKP-5** and **TKP-33** remained stable throughout the trajectories, whereas those formed with **TKP-31** and **TKP-32** progressively shifted toward unstable conformations (Figure 9a). These findings support a correlation between protein degradation activity and the stability of the ternary complex. Structural analyses further provide insights into how **TKP-5** forms the ternary complex through hydrophobic interactions involving Trp81, the E3 ligase ligand, and the triazole group in the linker (Figures 9b and S5). Trp81 displays an interaction fraction approaching 2, reflecting its sustained involvement in multiple hydrophobic contacts, which highlights its essential contribution to the stabilization of the ternary complex (Figure 9b). The linker adopts a turn-like conformation and accommodates itself within the interfacial groove between the two proteins, thereby stabilizing the interaction and effectively bridging them.

**Figure 9:**
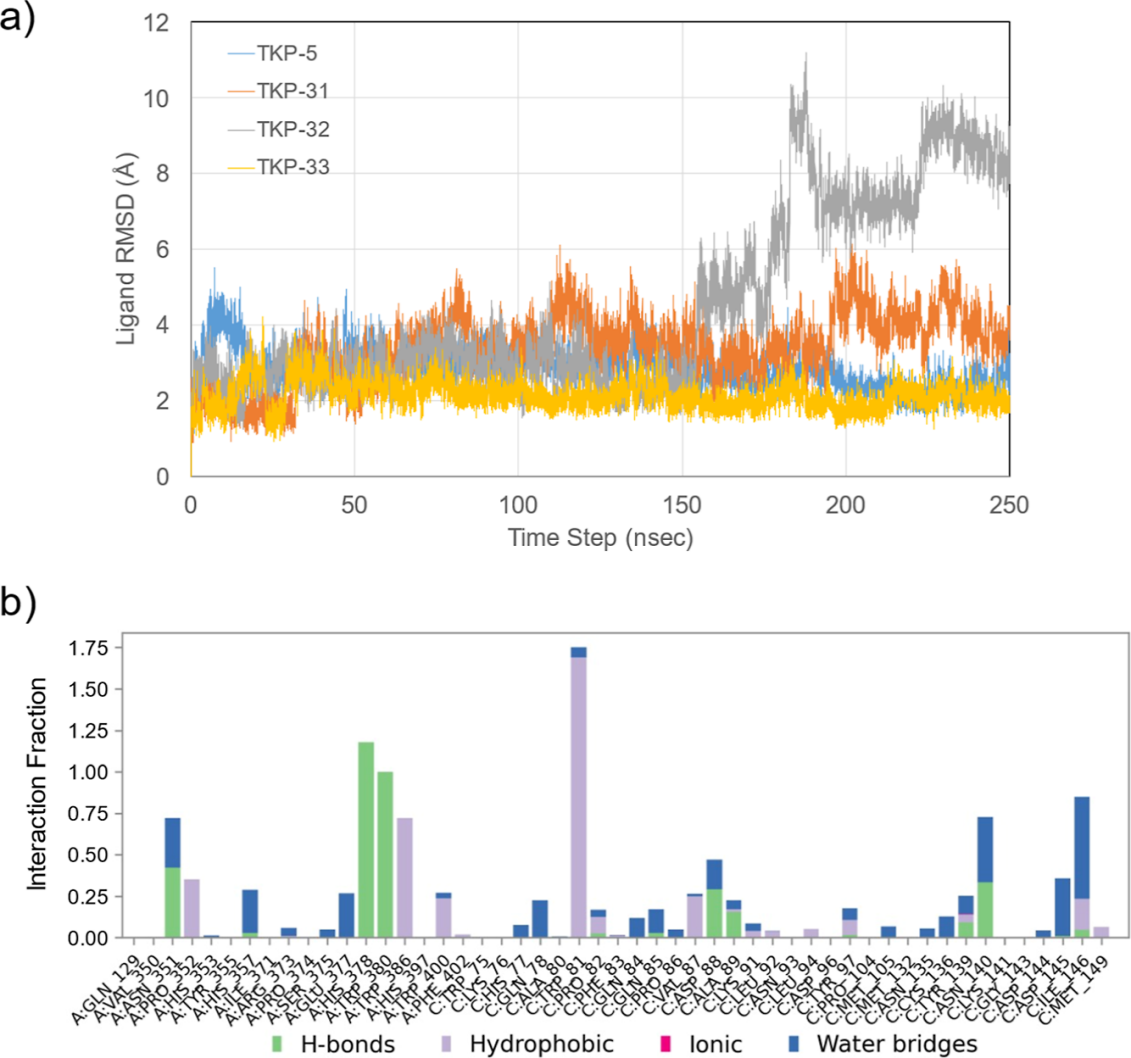
In silico analysis. The crystal structure of the DDB1B–CRBN–BRD4-BD1 complex cocrystallized with dBET23 was obtained from the Protein Data Bank (PDB ID: 6BN7), and monomers B and C were used for subsequent modeling. (a) Ligand RMSD values during the MD simulations. (b) Interaction fractions of protein–ligand contacts between CRBN–BRD4 and **TKP-5** throughout the MD simulation. MD, molecular dynamics.

### In Silico Analysis: Physicochemical Properties and ADMET Prediction

As shown in Scheme 5, a variety of transformations from 1,3-butadiyne were feasible, which consequently led to diversity in the physicochemical properties of the resulting compounds. The ADMET profiles of these compounds were therefore evaluated via in silico prediction (Figure S6). As a result of this synthetic flexibility, the compounds exhibited a broad distribution in molecular weight, ranging approximately from 800 to 1300. This wide dispersion in molecular weight was accompanied by substantial variability in multiple predicted parameters, including aqueous solubility (QPlogP and QPlogS), potential cardiotoxicity (QPlogHERG), and membrane permeability (QPPCaco). Collectively, these results suggest that the versatility of transformations accessible from the 1,3-butadiyne scaffold naturally gives rise to structural diversity, which is reflected in the broad spread of physicochemical and ADMET-related properties, while experimental evaluation will be important to fully assess the pharmacokinetic implications of this diversity.

### The Selectivity of TKP-5 against Other Bromodomains

A total of 61 bromodomain-containing proteins are expressed in the human proteome, distributed across 46 distinct proteins; therefore, bromodomain selectivity is a critical factor in the development of BET inhibitors. To enable rapid and comprehensive profiling, the BROMOscan platform is commercially available as a toolset.^42^ Using this service, we evaluated the selectivity of **TKP-5** at 1 μM and observed a strong preference for the BD1 domains of BRD2–4 and BRDT (Tables 1 and S3). These findings were highly consistent with those obtained from AlphaScreen assays using biotin probe **2**,^23^ demonstrating that **TKP-5**, despite carrying an additional CRBN ligand, retains ligand properties closely resembling those of the parent compound **TK-285**. In addition, TKP-5 was found to induce the degradation of BRD2 and BRD3 at concentrations below 100 nM, similar to its activity against BRD4 (Figure S7). Such pan-BRD (BRD2–4) degradation behavior has also been observed for known PROTACs, including dBET1 and ARV-825. Although BD2 plays a major role in regulating stimulus-responsive genes involved in inflammation and immune responses, such as IL-6 and TNF-α,^43^ **TK-285** selectively binds to BD1 and strongly suppresses TSLP production. **TKP-5** also binds preferentially to BD1, but by engaging a protein degradation mechanism, it is expected to functionally inhibit both BD1 and BD2. How **TKP-5** differs from **TK-285** in terms of in vivo effects through this additional functional blockade remains an intriguing question, but further investigation is required and falls beyond the scope of the present study.

**Table 1:** Selected Bromodomain Exhibiting Notable Binding of **TKP-5** at 1000 nM in the BROMOscan Panel (Eurofins Discovery, Fremont, CA, USA), with BD1 and BD2 Domain Data Presented for Each Protein

	% control @ 1000 nM	
BD, gene symbol	BD1	BD2
BRD2	0.95	79
BRD3	0.8	61
BRD4	9.4	66
BRDT	16	81

## Conclusions

Most studies in linkerology have focused on optimizing linker connection strategies, whereas approaches to diversifying preassembled linkers remain unexplored. In this study, we developed a divergent synthetic approach in which fully assembled 1,3-butadiyne-typed PROTACs serve as versatile intermediates, building on insights from biotin-probe-based target identification and linker-length optimization conducted in the typical screening phase. This approach, referred to as the “alkyne two-phase strategy,” allows rapid generation of linker-modified derivatives, including branched functional molecules such as fluorescent probes. Unlike conventional diversity-oriented syntheses, our method allows late-stage diversification, offering a distinct synthetic advantage. Late-stage modification is particularly efficient when selective alteration of a specific portion of an otherwise complete molecular framework is desired, as it can leverage existing intermediates. Importantly, 1,3-butadiyne intermediates can be readily obtained from alkyne-modified precursors, providing a practical and modular foundation for this strategy. Although the present study focuses on BRD4-targeting PROTACs, the underlying concept may also be applicable to PROTACs incorporating other E3 ligands, as well as to modular multicomponent functional molecules accessible through click chemistry. However, such extensions remain untested and will need to be validated in future studies.

In the context of BRD4 degraders, this study identified **TKP-5**, which exhibited stronger inhibitory activity against TSLP expression than **TK-285** at lower concentrations and further demonstrated superior efficacy in vivo. This approach also enabled rapid delineation of the structure–activity relationship of **TKP-5**, revealing the critical contribution of an aromatic moiety within the linker. To support clinical development, future efforts should focus on enhancing the metabolic stability of aliphatic linkers and incorporating conformationally constrained motifs to generate more drug-like compounds.

## Experimental Section

### Cell Culture

KCMH-1 is a mouse keratinocyte cell line that produces constitutively high levels of TSLP.^44^ The cells were cultured in minimum essential medium-α supplemented with 10% fetal bovine serum, 18 μg/mL of penicillin G potassium, and 50 μg/mL streptomycin sulfate, and maintained at 37 °C, 5% CO_2_, and 95% relative humidity. The cells were seeded at 1.5 × 10^5^ cells/mL in multiwell plates for the experiments.

### Western Blotting

KCMH-1 cells were seeded at a density of 1.5 × 10^5^ cells/well in MBS/FCS (9:1) buffer in 24-well plates; the plates were incubated for 24 h. The cells were washed with phosphate-buffered saline (PBS) and treated with 0.1, 1.0, and 10 μmol/mL of TKPs in MBS/FCS (9:1) buffer. After 24 h, KCMH-1 cells were washed twice with ice-cold PBS and lysed with ice-cold lysis buffer (20 mM HEPES buffer including 1% (v/v) Triton X-100, 10% (v/v) glycerol, 1 mM EDTA, 50 mM sodium fluoride, 2.5 mM *p*-nitrophenyl phosphate, 10 μg/mL phenylmethylsulfonyl fluoride, 1 mM Na_3_VO_4_, and 10 μg/mL leupeptin). Cell lysates were denatured and subjected to 10% (w/v) sodium dodecyl-sulfate–polyacrylamide gel electrophoresis (SDS–PAGE). Proteins were transferred onto nitrocellulose membranes (GE Healthcare, Buckinghamshire, England), and BRD4 protein levels were evaluated via immunoblotting using a rabbit polyclonal anti-BRD4 (Bethyl Laboratories) and an antirabbit horseradish peroxidase (HRP)-conjugated secondary antibody. After incubation, membranes were washed with TTBS three times for 10 min each and then incubated with avidin–biotin complex solution for 30 min at room temperature. Before detection, membranes were washed with TTBS as mentioned previously. After washing, the immunoreactive bands were detected using a chemiluminescence detection system (ECL system, PerkinElmer Life Sciences, Boston, MA).

To examine the degradation of BRD2 and BRD3 induced by TKP-5, KCMH-1 cells were cultured in 12-well plates. The cells were treated for 5 h with TKP-5, dBET1 (HY-101838, MedChemExpress, NJ, USA), dBET6 (HY-112588, MedChemExpress), MZ1 (HY-107425, MedChemExpress), or ARV-825 (HY-16954, MedChemExpress). The cells were then harvested and lysed in 120 μL of RIPA buffer (25 mM Tris–HCl, pH 8.0, 150 mM NaCl, 1% NP-40, 0.5% sodium deoxycholate, 0.1% SDS, 1 mM EDTA) supplemented with a protease inhibitor cocktail (#P8340, Sigma-Aldrich, St. Louis, MO, USA). The lysates were centrifuged at 16,100*g* for 15 min, and protein concentrations in the supernatants were determined using a BCA protein assay kit (#23227, Thermo Fisher Scientific, Waltham, MA, USA). Proteins were separated via SDS–PAGE and transferred onto membranes, followed by immunoblotting with primary antibodies against BRD4 (#83375, Cell Signaling Technology, Danvers, MA, USA; 1:1000), BRD3 (#sc-81202, Santa Cruz Biotechnology, Dallas, TX, USA; 1:500), or BRD2 (#5848, Cell Signaling Technology; 1:1000). HRP-conjugated antirabbit IgG (#7074, Cell Signaling Technology; 1:5000) or antimouse IgG (#7076, Cell Signaling Technology; 1:5000) was used as the secondary antibody.

To determine the DC_50_ value of TKP-5, KCMH-1 cells were cultured in 24-well plates and treated with various concentrations of TKP-5 for 5 h. The cells were then collected and lysed, and the lysates were denatured by boiling in 1× sample buffer (62.5 mM Tris–HCl, pH 6.8, 2% SDS, 10% glycerol) containing 5% 2-mercaptoethanol. BRD4 and α-tubulin protein levels were analyzed via fluorescent immunoblotting using primary antibodies against BRD4 (#83375, Cell Signaling Technology; 1:1000) and α-tubulin (LI-COR Biosciences, Lincoln, NE, USA, #926–42213; 1:1000). IRDye 800CW goat antirabbit IgG (LI-COR Biosciences, #925–32211; 1:10,000) and IRDye 680RD goat antimouse IgG (LI-COR Biosciences, #925–68,070; 1:10,000) were used as secondary antibodies. Fluorescent signals were detected using an Odyssey Fc imaging system (LI-COR Biosciences), and relative BRD4 protein levels were quantified using Empiria Studio software (LI-COR Biosciences).

### Ethics Statement

All animal experiments were reviewed and approved by the Institutional Animal Care and Use Committee of Tohoku University and were conducted in strict accordance with the relevant institutional guidelines and regulations for the care and use of laboratory animals (Ethics Approval Number: 2021PhA-006–01).

### Animal Experiments (Skin Barrier Disruption Model)

Eight-week-old male Institute for Cancer Research mice were purchased from Japan SLC (Shizuoka, Japan). The dorsal hair of each mouse was shaved and then removed using a depilatory cream (Veet, Reckitt Benckiser, England). 24 h after depilation, the dorsal skin was tape-stripped 10 times using adhesive tape (Cello tape, Nichiban, Japan). Mice exhibiting *trans*-epidermal water loss values greater than 140 g/(h·m^2^) after tape stripping were used for subsequent experiments. Immediately after tape stripping, 1% (w/w) **TKP-5** ointment or 1.2% (w/w) **TK-351** ointment (80 mg each) was applied to the injured skin and covered with a transparent dressing (Tegaderm, 3 M, USA). 6 h after application, dorsal skin samples were collected for RNA extraction.

### Quantitative Real-Time Polymerase Chain Reaction

Total RNA was extracted from the skin samples and subjected to quantitative PCR (qPCR) analysis of IL-33 mRNA expression. The following primer sets were used:

- mouse *Il*-33: 5′-GATGGGAAGAAGCTGATGGTG-3′ (forward) and 5′-TTGTGAAGGACGAAGAAGGC-3′ (reverse)
- mouse Gapdh: 5′-TGTGTCCGTCGTGGATCTGA-3′ (forward) and 5′-TTGCTGTTGAAGTCGCAGGAG-3′ (reverse)

*Gapdh* was used as an internal control, and relative expression levels of target genes were calculated using the comparative CT (ΔΔCT) method. Primer specificity was confirmed using melting curve analysis.

### In Silico Analysis

The proposed models of the CRBN–TKPs–BRD4-BD1 complexes were constructed using a workflow combining protein–ligand docking, conformational sampling, and MD simulations, as described below.

The crystal structure of the DDB1B–CRBN–BRD4-BD1 complex cocrystallized with dBET23 was obtained from the Protein Data Bank (PDB ID: 6BN7), and monomers B and C were used for subsequent modeling. The structure was preprocessed, minimized, and refined using the Protein Preparation Wizard implemented in the Schrödinger Suite (2020–3). This procedure included the removal of crystallographic waters, addition of missing hydrogens and side chains, and assignment of appropriate protonation and charge states using PROPKA. The OPLS3e force field was used for protein preparation.

All small molecules used for docking were prepared with the LigPrep module in the Schrödinger Suite using the OPLS3e force field, and the ionization states at pH 7.0 were generated using Epik.

To construct the complexes, we first generated a docking-based model of **TKP-5**. The POI–targeting moiety of **TKP-5** (compound **16D10**) was docked into the BRD4 ligand-binding pocket, followed by docking of the E3 ligase ligand, pomalidomide, into the CRBN-binding site of the ligase. Docking was performed using Glide^45^ in SP mode (version 8.8) with default parameters, and the top-ranked (Rank 1) pose was selected in each case.

Next, an initial **TKP-5** structure was manually constructed by connecting the docked **16D10** and pomalidomide molecules with the **TKP-5** linker while preserving their docked orientations within BRD4 and the E3 ligase, respectively. Conformational sampling of the linker was then performed using the LowModeMD method implemented in MOE 2024.0601 (Chemical Computing Group), while restraining all nonlinker ligand atoms. A stable **TKP-5** binding model was generated by selecting the conformation with the lowest potential energy for the linker. Finally, the entire complex was reoptimized using the Minimize function of the Protein Preparation Wizard. Binding models for **TKP-31**, **TKP-32**, and **TKP-33** were generated by using the **TKP-5** complex model as a template. The **TKP-5** ligand structure was edited to the corresponding **TKP-31**, **TKP-32**, and **TKP-33** structures using the Maestro Builder function, followed by full optimization with the Minimize function of the Protein Preparation Wizard. The binding stability of all complex models was subsequently evaluated by MD simulations.

MD simulations of the BRD4–E3 ligase complexes with TKP-series ligands were performed using Desmond (version 7.8.139; Schrödinger, LLC, New York, NY, USA) with the OPLS4 force field. The systems were solvated in SPC water containing 0.15 M NaCl, followed by energy minimization and equilibration. Production MD simulations were carried out as two independent 250 ns trajectories initialized with distinct random seeds. All simulations were performed in the isothermal–isobaric (NPT) ensemble at 300 K and 1 bar using the Nosé–Hoover thermostat. Long-range electrostatics were treated using the Smooth Particle Mesh Ewald method, and trajectory snapshots were saved every 10 ps. Ligand RMSD and ligand–protein interaction analyses were performed using the Simulation Interaction Diagram module in Maestro.

### Chemical Synthesis and Compound Data

HPLC analysis showed that the purities of **TK-351** and **TKP-5** used in the in vivo studies were >95% (Figure S8), while the purities of the remaining tested compounds were determined by nuclear magnetic resonance (NMR) analysis.

All reactions were carried out under an argon atmosphere with dehydrated solvents under anhydrous conditions, unless otherwise stated. Dehydrated tetrahydrofuran (THF) and CH_2_Cl_2_ were purchased, and other solvents were dehydrated and distilled according to standard protocols. Yields refer to chromatographically purified products unless otherwise stated. Reagents were obtained from commercial suppliers and used without further purification, unless otherwise stated. Reactions were monitored by thin-layer chromatography (TLC) carried out on 0.25 mm Merck silica gel plates (60F254). Column chromatography was performed on silica gel 60N (spherical, 63–210 μm or 40–50 μm).

^1^H NMR (400 and 600 MHz) and ^13^C NMR spectra (100 and 150 MHz) were recorded on a Varian 400 MR, JEOL JNM-AL400, JEOL JNM-ECZL400S, JEOL JNM-ECA600, or JEOL JNM-ECZ600 spectrometers. For ^1^H NMR spectra, chemical shifts (δ) are given from TMS (δ_H_ 0.00), CDCl_3_ (δ_H_ 7.26), DMSO-*d*_6_ (δ_H_ 2.50) or CD_3_OD (δ_H_ 3.31) as an internal standard. The following abbreviations were used to explain the multiplicities: s, singlet; d, doublet; t, triplet; q, quartet; quint, quintet; m, multiplet; br, broad. Coupling constants (*J*) are reported in hertz (Hz). Data are presented as follows: chemical shift, multiplicity, coupling constants, and integration. For ^13^C NMR spectra, chemical shifts (δ) are given from CDCl_3_ (δ_C_ 77.16), DMSO-*d*_6_ (δ_C_ 39.5), and CD_3_OD (δ_C_ 49.0) as an internal standard. Infrared spectra were recorded on a JASCO FT-IR-410 at 4.0 cm^–1^ resolution and are reported in wavenumbers. High-resolution mass spectra (HRMS) were recorded on a JEOL JMS-700 MS using electron impact (EI) with a magnetic sector or time-of-flight mass analyzer, or by fast atom bombardment (FAB) with a magnetic sector or time-of-flight mass analyzer.

### Synthesis of Alkyne-Modified TK-285 Analogs

#### 2-((5-Bromopyridin-2-yl)­methoxy)­benzaldehyde (**10**)

PBr_3_ (0.75 mL, 7.9 mmol) was added to a solution of alcohol **8** (500 mg, 2.66 mmol) in CH_2_Cl_2_ (10 mL) at 0 °C. After 4 h of stirring at room temperature, the reaction was quenched with H_2_O (10 mL). The mixture was neutralized with saturated aqueous NaHCO_3_ (until pH 8) at 0 °C and extracted with AcOEt (3 × 20 mL). The combined organic extracts were washed with brine (50 mL) and dried over anhydrous Na_2_SO_4_. Filtration and evaporation in vacuo furnished the crude bromide **9** (457 mg) as white solids. It was used without further purification.

Salicylaldehyde (280 μL, 2.66 mmol) and K_2_CO_3_ (735 mg, 5.32 mmol) were added to a solution of crude bromide **9** (457 mg) in dimethylformamide (DMF) (5.3 mL) at 0 °C. After 6 h of stirring at room temperature, the reaction was quenched with H_2_O (20 mL) at 0 °C, and the mixture was extracted with *n*-hexane/AcOEt (1:4, 3 × 20 mL). The combined organic extracts were washed with brine (3 × 50 mL) and dried over anhydrous Na_2_SO_4_. Filtration and evaporation in vacuo furnished the crude product, which was purified by flash column chromatography (4:1 → 2:1, *n*-hexane/AcOEt) to give aldehyde **10** (431 mg, 56% in 2 steps) as a colorless solid. IR (neat) 2861, 1686, 1597, 1562, 1526, 1482, 1455, 1391, 1349, 1286, 1238, 1161, 1086, 1015, 835, 757 cm^–1^; ^1^H NMR (399 MHz, CDCl_3_): δ 10.48 (s, 1H), 8.46 (d, *J* = 2.5 Hz, 1H), 7.86 (dd, *J* = 7.6, 1.9 Hz, 1H), 7.68 (dd, *J* = 8.2, 2.5 Hz, 1H), 7.59–7.53 (m, 2H), 7.10 (t, *J* = 7.6 Hz, 1H), 7.03 (d, *J* = 8.4 Hz, 1H), 5.17 (s, 2H); ^13^C NMR (100 MHz, CDCl_3_): δ 189.2 (CH), 160.3 (C), 149.2 (CH), 142.3 (C), 137.8 (CH), 136.1 (CH), 131.2 (C), 129.3 (CH), 128.5 (CH), 125.5 (C), 121.9 (CH), 112.9 (CH), 67.5 (CH_2_); HRMS (FAB) *m*/*z*: [M + H]^+^ calcd for C_13_H_11_BrNO_2_, 291.9968; found, 291.9974.

#### 2-((5-((Trimethylsilyl)­ethynyl)­pyridin-2-yl)­methoxy)­benzaldehyde (**11**)

A mixture of CuI (39.1 mg, 205 μmol), Pd (PPh_3_)_2_Cl_2_ (36.0 mg, 51.5 μmol), triethylamine (600 μL, 4.31 mmol), ethynyl trimethyl silane (350 μL, 2.48 mmol), and aldehyde **10** (300 mg, 1.03 mmol) in THF (2.7 mL) was degassed. After 8 h of stirring at 50 °C, the mixture was filtered through a Celite pad, and the Celite was washed with AcOEt. The filtrate was dried over anhydrous Na_2_SO_4_. Filtration and evaporation in vacuo furnished the crude product, which was purified by flash column chromatography (6:1, *n*-hexane/AcOEt) to acetylene **11** (296 mg, 957 μmol, 93%) as a colorless solid. IR (neat) 2959, 2898, 2861, 2164, 1687, 1598, 1583, 1561, 1482, 1456, 1393, 1286, 1249, 1237, 1161, 1016, 869, 841, 758 cm^–1^; ^1^H NMR (399 MHz, CDCl_3_): δ 10.51 (br, 1H), 8.65 (dd, *J* = 2.3, 0.9 Hz, 1H), 7.87 (dd, *J* = 7.7, 1.8 Hz, 1H), 7.77 (dd, *J* = 8.0, 2.3 Hz, 1H), 7.55 (ddd, *J* = 8.5, 7.3, 1.8 Hz, 1H), 7.51 (dd, *J* = 8.0, 0.9 Hz, 1H), 7.09 (ddd, 7.7, 7.3, 1.0, 1H), 7.03 (dd, *J* = 8.5, 1.0 Hz, 1H), 5.21 (s, 2H), 0.28 (s, 9H); ^13^C NMR (100 MHz, CDCl_3_): δ 189.3 (CH), 160.5 (C), 150.7 (C), 149.0 (CH), 143.3 (C), 136.0 (CH), 135.3 (CH), 131.3­(C), 129.2 (CH), 127.4 (CH), 121.8 (CH), 113.0 (CH), 103.4 (C), 95.6 (C), 68.0 (CH_2_), 0.16 (CH_3_); HRMS (FAB) *m*/*z*: [M + H]^+^ calcd for C_18_H_20_NO_2_Si, 310.1258; found, 310.1266.

#### *N*-(4-Ethoxy-3-methoxyphenethyl)­acetamide (**13**)

Ammonium acetate (343 mg, 4.45 mmol) was added to a solution of 4-ethoxy-3-methoxybenzaldehyde (**12**, 8.02 g, 44.5 mmol) in MeNO_2_ (40 mL), and the mixture was refluxed for 2 h. After cooling to 0 °C, the resulting yellow solid was filtrated and washed with Et_2_O to give nitro alkene (6.27 g) as a yellow solid. It was used without further purification.

The crude nitroalkene (5.24 g, 23.5 mmol) in THF (10 mL) was added dropwise to a suspension of LiAlH_4_ (2.66 g, 70.0 mmol) in THF (90 mL) at 0 °C, and the mixture was refluxed for 5 h. After cooling the reaction mixture to 0 °C, it was quenched by dropwise addition of H_2_O (3 mL), 10% aqueous NaOH (3 mL), and H_2_O (9 mL). The suspension was filtered through a Celite pad, and the filtrate was evaporated in vacuo to give amine (4.88 g) as an orange amorphous solid. It was used without further purification.

Ac_2_O (5.40 g, 53.0 mmol) and Et_3_N (8.00 g, 78.9 mmol) were added to a solution of crude amine (4.88 g, 25.0 mmol) in CH_2_Cl_2_ (50 mL). After 18 h of stirring at room temperature, the reaction mixture was concentrated in vacuo, and the residue was purified by flash column chromatography (10:1 → 0:1, *n*-hexane/AcOEt) to give amide **13** (4.21 g, 40% in 3 steps) as a colorless solid. IR (ATR) 3305, 3086, 2978, 2936, 2867, 1744, 1644, 1591, 1557, 1515, 1282, 1261, 1234, 1141, 1034, 845, 810, 715 cm^–1^; ^1^H NMR (399 MHz, CDCl_3_): δ 6.78 (d, *J* = 8.2 Hz, 1H), 6.69 (s, 1H), 6.68 (d, *J* = 8.2 Hz, 1H), 5.67 (br, 1H), 4.05 (q, *J* = 7.2 Hz, 2H), 3.83 (s, 3H), 3.48–3.43 (m, 2H), 2.73 (t, *J* = 7.0 Hz, 2H), 1.91 (s, 3H), 1.42 (t, *J* = 7.2 Hz, 3H); ^13^C NMR (100 MHz, CDCl_3_): δ 170.1 (C), 149.3 (C), 146.9 (C), 131.3 (C), 120.6 (CH), 112.9 (CH), 112.1 (CH), 64.3 (CH_2_), 55.9 (CH_3_), 40.8 (CH_2_), 35.2 (CH_2_), 23.3 (CH_3_), 14.8 (CH_3_); HRMS (EI) *m*/*z*: [M]^+^ calcd for C_13_H_19_NO_3_, 237.1365; found, 237.1349.

#### *N*-(2-Acetyl-4-ethoxy-5-methoxyphenethyl)­acetamide (**14**)

Acetyl chloride (900 μL, 12.6 mmol) was added to a solution of amide **13** (1.00 g, 4.21 mmol) and AlCl_3_ (1.17 g, 8.42 mmol) in nitrobenzene (8.4 mL) at 20 °C. After 10 h of stirring at room temperature, the reaction mixture was poured into ice-cooled (0 °C) water. The resulting mixture was extracted with CHCl_3_ (30 mL). The combined organic extracts were washed with brine (30 mL) and dried over anhydrous Na_2_SO_4_. Filtration and evaporation in vacuo furnished the crude product, which was purified by flash column chromatography (4:1, *n*-hexane/AcOEt) to give methyl ketone **14** (940 mg, 80%) as a colorless solid. IR (ATR) 3326, 2975, 2868, 1670, 1644, 1604, 1565, 1520, 1359, 1213, 1189, 1159, 1058, 726 cm^–1^; ^1^H NMR (399 MHz, CDCl_3_): δ 7.20 (s, 1H), 6.75 (s, 1H), 6.65 (br, 1H), 4.12 (q, *J* = 7.0 Hz, 2H), 3.91 (s, 3H), 3.52–3.48 (m, 2H), 2.98 (t, *J* = 6.7 Hz, 2H), 2.58 (s, 3H), 1.90 (s, 3H), 1.48 (t, *J* = 7.0 Hz, 3H); ^13^C NMR (100 MHz, CDCl_3_): δ 201.4 (C), 170.5 (C), 152.7 (C), 146.3 (C), 134.6 (C), 130.1 (C), 114.8 (CH), 114.4 (CH), 65.1 (CH_2_), 56.2 (CH_3_), 42.0 (CH_2_), 32.8 (CH_2_), 29.6 (CH_3_), 23.4 (CH_3_), 14.9 (CH_3_); HRMS (FAB) *m*/*z*: [M + H]^+^ calcd for C_15_H_21_NO_4_, 279.1471; found, 279.1459.

### General Procedure A: Aldol Condensation

A solution of aldehyde (0.9–1.1 equiv) and methyl ketone **14** (1.0 equiv) in 10% aq. NaOH/EtOH (1:1) was stirred at 20 °C. After 1 h of stirring at room temperature, the reaction mixture was diluted with H_2_O at 0 °C, and the mixture was extracted with CHCl_3_ twice. The combined organic extracts were dried over anhydrous Na_2_SO_4_. Filtration and evaporation in vacuo furnished the crude product, which was purified by flash column chromatography (100:0 → 20:1, AcOEt/MeOH) to give the enone compound.

#### **TK-351** [Alkyne-modified **TK-285**]: (*E*)-*N*-(4-Ethoxy-2-(3-(2-((6-ethynylpyridin-3-yl)­methoxy)­phenyl)­acryloyl)-5-methoxyphenethyl)­acetamide (**1**)

Following general procedure A, **TK-351** was prepared in 69% yield as a yellow solid using aldehyde **11** (1.1 equiv) and methyl ketone **14** (1.0 equiv). IR (neat) 3726, 3284, 2933, 2109, 1656, 1597, 1563, 1516, 1486, 1454, 1354, 1262, 1158, 1131, 1105, 1035, 989, 836, 753, 669, 650 cm^–1^; ^1^H NMR (399 MHz, CDCl_3_): δ 8.60 (dd, *J* = 2.3, 0.9 Hz, 1H), 7.94 (d, *J* = 16.1 Hz, 1H), 7.65 (dd, *J* = 8.1, 2.3 Hz, 1H), 7.65 (dd, *J* = 7.5, 1.7 Hz, 1H), 7.50 (dd, *J* = 8.1, 0.9 Hz, 1H), 7.39 (ddd, *J* = 8.7, 7.5, 1.7 Hz,1H), 7.19 (d, *J* = 16.1 Hz, 1H), 7.10 (br, 1H), 7.05 (td, *J* = 7.5, 1.4, 1H), 7.02 (s, 1H), 6.96 (dd, *J* = 8.7, 1.4 Hz, 1H), 6.81 (s, 1H), 5.17 (s, 2H), 4.02 (q, *J* = 7.0 Hz, 2H), 3.94 (s, 3H), 3.56–3.46 (m, 2H), 3.18 (s, 1H), 2.90–2.82 (m, 2H), 1.92 (s, 3H), 1.42 (t, *J* = 7.0 Hz, 3H); ^13^C NMR (100 MHz, CDCl_3_): δ 196.0 (C), 170.6 (C), 157.1 (C), 151.9 (C), 148.8 (CH), 146.2 (C), 142.3 (C), 141.3 (CH), 135.1 (CH), 133.2 (C), 132.3 (CH), 132.0 (C), 131.3 (C), 128.9 (CH), 127.5 (CH), 127.2 (CH), 124.1 (C), 121.9 (CH), 113.8 (CH), 113.6 (CH), 112.8 (CH), 82.5 (C), 77.8 (CH), 67.8 (CH_2_), 64.9 (CH_2_), 56.1 (CH_3_), 41.9 (CH_2_), 31.9 (CH_2_), 23.3 (CH_3_), 14.8 (CH_3_); HRMS (EI) *m*/*z*: [M]^+^ calcd for C_30_H_30_N_2_O_5_, 498.2155; found, 498.2130.

#### (*E*)-*N*-(4-Ethoxy-5-methoxy-2-(3-(2-(prop-2-yn-1-yloxy)­phenyl)­acryloyl)­phenethyl)­acetamide (**3**)

Following general procedure A, compound **3** was prepared in 88% yield as a yellow solid using aldehyde **15** (1.1 equiv) and methyl ketone **14** (1.0 equiv). IR (ATR) 3300, 3245, 2938, 2118, 1632, 1597, 1557, 1515, 1438, 1344, 1256, 1211, 1158, 1018, 745 cm^–1^; ^1^H NMR (399 MHz, CDCl_3_): δ 7.91 (d, *J* = 16.2 Hz, 1H), 7.61 (dd, *J* = 7.7, 1.7 Hz, 1H), 7.40 (m, 1H), 7.27 (m, 1H), 7.25 (d, *J* = 16.2 Hz, 1H), 7.12–6.97 (m, 3H), 6.82 (br, 1H), 4.77 (d, *J* = 2.3 Hz, 2H), 4.11 (q, *J* = 7.0 Hz, 2H), 3.93 (s, 3H), 3.58–3.49 (m, 2H), 2.88 (t, *J* = 6.2 Hz, 2H), 2.53 (t, *J* = 2.3 Hz, 1H), 1.92 (s, 3H), 1.47 (t, *J* = 7.0 Hz, 3H); ^13^C NMR (100 MHz, CDCl_3_): δ 195.9 (C), 170.7 (C), 156.7 (C), 151.9 (C), 146.1 (C), 141.7 (CH), 133.5 (C), 132.1 (CH), 131.4 (C), 129.3 (CH), 127.3 (CH), 124.2 (C), 121.9 (CH), 113.7 (2 × CH),112.9 (CH), 78.1 (C), 76.3 (CH), 64.9 (CH_2_), 56.2 (CH_2_), 56.1 (CH_3_), 42.2 (CH_2_), 31.7 (CH_2_), 23.4 (CH_3_), 14.9 (CH_3_); HRMS (EI) *m*/*z*: [M]^+^ calcd for C_25_H_27_NO_5_, 421.1889; found, 421.1897.

#### (*E*)-*N*-(4-Ethoxy-5-methoxy-2-(3-(3-(prop-2-yn-1-yloxy)­phenyl)­acryloyl)­phenethyl)­acetamide (**4**)

Following general procedure A, compound **4** was prepared in 93% yield as a yellow solid using aldehyde **16** (1.1 equiv) and methyl ketone **14** (1.0 equiv). IR (ATR) 3310, 3260, 2934, 2887, 2127, 1738, 1656, 1634, 1598, 1582, 1562, 1519, 1344, 1267, 1247, 1131, 1036, 746 cm^–1^; ^1^H NMR (399 MHz, CDCl_3_): δ 7.54 (d, *J* = 15.9 Hz, 1H), 7.36 (t, *J* = 7.8 Hz, 1H), 7.22 (dt, *J* = 7.8, 1.7 Hz, 1H), 7.19 (t, *J* = 1.7 Hz, 1H), 7.15 (d, *J* = 15.9 Hz, 1H), 7.10 (s, 1H), 7.09–7.03 (m, 2H), 6.82 (br, 1H), 4.73 (d, *J* = 2.4 Hz, 2H), 4.10 (q, *J* = 7.0 Hz, 2H), 3.93 (s, 3H), 3.58–3.49 (m, 2H), 2.86 (t, *J* = 6.2 Hz, 2H), 2.54 (t, *J* = 2.4 Hz, 1H), 1.92 (s, 3H), 1.47 (t, *J* = 7.0 Hz, 3H); ^13^C NMR (100 MHz, CDCl_3_): δ 195.1 (C), 170.5 (C), 157.9 (C), 152.0 (C), 146.1 (C), 145.8 (CH), 135.9 (C), 133.5 (C), 131.0 (CH), 130.1 (C), 126.6 (CH), 122.0 (CH), 117.4 (CH), 114.6 (CH), 113.7 (CH), 113.4 (CH), 78.1 (C), 75.9 (CH), 64.9 (CH_2_), 56.0 (CH_3_), 55.9 (CH_2_), 42.0 (CH_2_), 31.7 (CH_2_), 23.2 (CH_3_), 14.7 (CH_3_); HRMS (EI) *m*/*z*: [M]^+^ calcd for C_25_H_27_NO_5_, 421.1889; found, 421.1912.

#### (*E*)-*N*-(4-Ethoxy-5-methoxy-2-(3-(4-(prop-2-yn-1-yloxy)­phenyl)­acryloyl)­phenethyl)­acetamide (**5**)

Following general procedure A, compound **5** was prepared in 93% yield as a yellow solid using aldehyde **17** (1.1 equiv) and methyl ketone **14** (1.0 equiv). IR (ATR) 3320, 3293, 3244, 2938, 2882, 2122, 1739, 1644, 1592, 1558, 1509, 1289, 1250, 1178, 1130, 1031, 825 cm^–1^; ^1^H NMR (399 MHz, CDCl_3_): δ 7.59–7.49 (m, 3H), 7.21 (s, 1H), 7.09–6.97 (m, 4H), 6.81 (br, 1H), 4.74 (d, *J* = 2.4 Hz, 2H), 4.10 (q, *J* = 7.0 Hz, 2H), 3.92 (s, 3H), 3.57–3.48 (m, 2H), 2.86 (t, *J* = 6.2 Hz, 2H), 2.55 (t, *J* = 2.4 Hz, 1H), 1.92 (s, 3H), 1.46 (t, *J* = 7.0 Hz, 3H); ^13^C NMR (100 MHz, CDCl_3_): δ 195.6 (C), 170.6 (C), 159.9 (C), 152.0 (C), 146.2 (C), 146.2 (CH), 133.3 (C), 131.5 (C), 130.4 (CH), 128.1 (C), 124.6 (CH), 115.6 (CH), 113.7 (CH), 113.5 (CH), 78.0 (C), 76.2 (CH), 65.0 (CH_2_), 56.2 (CH_3_), 56.0 (CH_2_), 42.1 (CH_2_), 31.7 (CH_2_), 23.3 (CH_3_), 14.9 (CH_3_); HRMS (EI) *m*/*z*: [M]^+^ calcd for C_25_H_27_NO_5_, 421.1889; found, 421.1889.

#### (*E*)-*N*-(4-Ethoxy-2-(3-(4-(1-ethoxyethoxy)­phenyl)­acryloyl)-5-methoxyphenethyl)­acetamide (**19**)

Following general procedure A, compound **19** was prepared in 93% yield as a yellow solid using aldehyde **18** ^46^ (0.9 equiv) and methyl ketone **14** (1.0 equiv). IR (neat) 3297, 2977, 2935, 1654, 1598, 1568, 1508, 1444, 1348, 1261, 1176, 1131, 1041, 937, 896, 830 cm^–1^; ^1^H NMR (399 MHz, CDCl_3_): δ 7.58–7.48 (m, 3H), 7.22 (br, 1H), 7.06–7.02 (m, 4H), 6.81 (s, 1H), 5.47 (q, *J* = 5.3 Hz, 1H), 4.10 (q, *J* = 7.0 Hz, 2H), 3.92 (s, 3H), 3.76 (dq, *J* = 9.4, 7.1 Hz, 1H), 3.61–3.49 (m, 3H), 2.87 (t, *J* = 6.4 Hz, 2H), 1.92 (s, 3H), 1.53 (d, *J* = 5.3 Hz, 3H), 1.46 (t, *J* = 7.0 Hz, 3H), 1.20 (t, *J* = 7.1 Hz, 3H); ^13^C NMR (100 MHz, CDCl_3_): δ 195.4 (C), 170.5 (C), 159.4 (C), 151.8 (C), 146.1 (CH), 146.1 (C), 133.1 (C), 131.4 (C), 130.2 (CH), 127.9 (C), 124.3 (CH), 117.4 (CH), 113.6 (CH), 113.5 (CH), 99.2 (CH), 64.9 (CH_2_), 61.1 (CH_2_), 56.0 (CH_3_), 42.0 (CH_2_), 31.6 (CH_2_), 23.1 (CH_3_), 20.0 (CH_3_), 15.1 (CH_3_), 14.7 (CH_3_); HRMS (FAB) *m*/*z*: [M + H]^+^ calcd for C_26_H_34_NO_6_, 456.2381; found, 456.2372.

#### *N*-(4-Ethoxy-2-(3-(4-hydroxyphenyl)­propanoyl)-5-methoxyphenethyl)­acetamide (**20**)

5% Pd/C (77.1 mg) was added to a solution of enone **19** (400 mg, 0.906 mmol) in AcOEt (7.6 mL), and the mixture was stirred under hydrogen for 8 h. The reaction mixture was filtered through a Celite pad, and the filtrate was dried over anhydrous Na_2_SO_4_. Filtration and evaporation in vacuo furnished the crude product (438 mg) as colorless solids used for the next reaction.

PPTS (22.7 mg, 90.2 μmol) was added to a solution of crude ethyl vinyl ether (438 mg) in EtOH (7.2 mL). After 1 h of stirring at 50 °C, the reaction was quenched with sat. NaHCO_3_ (20 mL), and the mixture was extracted with AcOEt (3 × 30 mL). The combined organic extracts were washed with brine (100 mL) and dried over anhydrous Na_2_SO_4_. Filtration and evaporation in vacuo furnished the crude product, which was purified by flash column chromatography (50:1 → 20:1, CHCl_3_/MeOH) to give phenol **20** (242 mg, 69% in 2 steps) as a colorless solid. IR (ATR) 3365, 3209, 2933, 1676, 1615, 1570, 1515, 1444, 1370, 1265, 1227, 1204, 1129, 1063, 885, 795 cm^–1^; ^1^H NMR (399 MHz, CDCl_3_): δ 7.09–7.01 (m, 3H), 6.84 (br, 1H), 6.83–6.71 (m, 3H), 6.27 (br, 1H), 4.04 (q, *J* = 7.0 Hz, 2H), 3.88 (s, 3H), 3.46 (m, 2H), 3.16 (t, *J* = 7.4 Hz, 2H), 2.96 (t, *J* = 7.4 Hz, 2H), 2.85 (t, *J* = 6.7 Hz, 2H), 1.91 (s, 3H), 1.44 (t, *J* = 7.0 Hz, 3H); ^13^C NMR (100 MHz, CDCl_3_): δ 203.5 (C), 170.7 (C), 154.6 (C), 152.3 (C), 146.2 (C), 133.8 (C), 132.4 (C), 130.3 (C), 129.4 (CH), 115.4 (CH), 114.1 (CH), 113.5 (CH), 64.9 (CH_2_), 56.0 (CH_3_), 43.3 (CH_2_), 41.9 (CH_2_), 32.2 (CH_2_), 29.9 (CH_2_), 23.1 (CH_3_), 14.7 (CH_3_); HRMS (EI) *m*/*z*: [M]^+^ calcd for C_22_H_27_NO_5_, 385.1889; found, 385.1910.

#### *N*-(4-Ethoxy-5-methoxy-2-(3-(4-(prop-2-yn-1-yloxy)­phenyl)­propanoyl)­phenethyl)­acetamide (**6**)

Potassium carbonate (32.6 mg, 236 μmol) and propargyl bromide (20.0 μL, 231 μmol) were added to a solution of phenol **20** (75.8 mg, 197 μmol) in DMF (400 μL). After 8 h of stirring at room temperature, the reaction was quenched with H_2_O (10 mL), and the mixture was extracted with *n*-hexane/AcOEt (1:4, 3 × 10 mL). The combined organic extracts were washed with brine (10 mL) and dried over anhydrous Na_2_SO_4_. Filtration and evaporation in vacuo furnished the crude product, which was purified by flash column chromatography (50:1 → 20:1, CHCl_3_/MeOH) to give propargyl ether **6** (63.8 mg, 77%) as colorless solids. IR (ATR) 3310, 3249, 2972, 2938, 2923, 2112, 1678, 1633, 1565, 1509, 1361, 1349, 1270, 1213, 1128, 1033, 821 cm^–1^; ^1^H NMR (399 MHz, CDCl_3_): δ 7.19–7.09 (m, 2H), 7.06 (s, 1H), 6.96–6.86 (m, 2H), 6.74 (s, 1H), 6.67 (br, 1H), 4.67 (d, *J* = 2.4 Hz, 2H), 4.04 (q, *J* = 7.0 Hz, 2H), 3.90 (s, 3H), 3.48 (m, 2H), 3.17 (t, *J* = 7.5 Hz, 2H), 2.98 (t, *J* = 7.5 Hz, 2H), 2.88 (t, *J* = 6.7 Hz, 2H), 2.51 (t, *J* = 2.4 Hz, 1H), 1.91 (s, 3H), 1.45 (t, *J* = 7.0 Hz, 3H); ^13^C NMR (100 MHz, CDCl_3_): δ 203.0 (C), 170.3 (C), 156.1 (C), 152.3 (C), 146.2 (C), 134.0 (C), 133.9 (C), 130.1 (C), 129.4 (CH), 115.0 (CH), 114.1 (CH), 113.6 (CH), 78.6 (C), 75.4 (CH), 64.9 (CH_2_), 56.0 (CH_3_), 55.8 (CH_2_), 43.2 (CH_2_), 41.8 (CH_2_), 32.4 (CH_2_), 29.9 (CH_2_), 23.2 (CH_3_), 14.7 (CH_3_); HRMS (EI) *m*/*z*: [M]^+^ calcd for C_25_H_29_NO_5_, 423.2046; found, 423.2049.

#### *N*-(4-Ethoxy-5-methoxy-2-((1R*,2R*)-2-(4-(prop-2-yn-1-yloxy)­phenyl)­cyclopropane-1-carbonyl)­phenethyl) Acetamide (**7**)

Enone **5** (40.2 mg, 95.4 μmol) in DMSO (500 μL) was added to the mixture of NaH (60% wt, 7.63 mg, 191 μmol) and trimethylsulfoxonium iodide (42.0 mg, 191 μmol). After 30 min of stirring at room temperature, the mixture was stirred for an additional 30 min at 50 °C. The reaction was quenched with brine (10 mL) and extracted with CHCl_3_ (2 × 10 mL). The combined organic extracts were washed with brine (3 × 20 mL) and dried over anhydrous Na_2_SO_4_. Filtration and evaporation in vacuo furnished the crude product, which was purified by flash column chromatography (100:0 → 50:1, CHCl_3_/MeOH) to give cyclopropane **7** (33.1 mg, 76.0 μmol, 80%) as colorless solids. IR (neat) 3288, 3084, 2978, 2934, 2868, 2118, 1655, 1602, 1560, 1513, 1444, 1391, 1349, 1263, 1239, 1201, 1132, 1026, 970, 923, 824, 751 cm^–1^; ^1^H NMR (399 MHz, CDCl_3_): δ 7.19 (s, 1H), 7.12 (d, *J* = 8.8 Hz, 2H), 6.93 (d, *J* = 8.8 Hz, 1H), 6.88 (s, 1H), 6.75 (s, 1H), 4.67 (d, *J* = 2.4 Hz, 2H), 3.97–4.04 (m, 2H), 3.89 (s, 3H), 3.52–3.47 (m, 2H), 2.88 (t, *J* = 6.6 Hz, 2H), 2.62 (ddd, *J* = 9.8, 6.5, 5.8 Hz, 1H), 2.57 (ddd, *J* = 8.8, 6.5, 4.6 Hz, 1H), 2.51 (t, *J* = 2.4 Hz, 2H), 1.89 (m, 1H), 1.89 (s, 3H), 1.53 (m, 1H), 1.39 (t, *J* = 7.0 Hz, 3H); ^13^C NMR (100 MHz, CDCl_3_): δ 202.1 (C), 170.5 (C), 156.6 (C), 152.3 (C), 146.5 (C), 133.2 (C), 133.1 (C), 131.6 (C), 127.4 (CH), 115.3 (CH), 113.9 (CH), 113.8 (CH), 78.6 (C), 75.7 (CH), 64.9 (CH_2_), 56.1 (CH_3_), 56.0 (CH_2_), 42.1 (CH_2_), 32.9 (CH), 32.2 (CH_2_), 30.3 (CH), 23.3 (CH_3_), 19.2 (CH_2_), 14.8 (CH_3_); HRMS (EI) *m*/*z*: [M]^+^ calcd for C_26_H_29_NO_5_, 435.2046; found, 435.2024.

### Synthesis of Azide-Modified Pomalidomides

#### General Procedure B: Preparation of Azide-Modified Pomalidomides **27–32**

Acid chloride (1.5 equiv) was added to a solution of pomalidomide (1.0 equiv, for **27**–**31**) or *N*-Me pomalidomide (1.0 equiv, for **32**) in THF at 0 °C. After 24 h of stirring at 40 °C, the reaction mixture was concentrated in vacuo. The resulting mixture was diluted with Et_2_O. The solid was filtered and washed with Et_2_O to give the amide as a cream solid. It was used without further purification.

Sodium azide (3.0 equiv) was added to a solution of crude alkyl chloride (for **27**, **28**) or alkyl bromide (for **29**–**32**) in solvent (acetone for **27**, DMF for **28**–**32**) at 0 °C. After 24 h of stirring at 50 °C, the reaction mixture was cooled to room temperature, and then, H_2_O was added. The solid was filtered and washed with H_2_O to give azides **27**–**32**.

##### 2-Azido-*N*-(2-(2,6-dioxopiperidin-3-yl)-1,3-dioxoisoindolin-4-yl)­acetamide (**27**)

Following general procedure B, compound **27** was prepared in 98% yield as a cream solid using pomalidomide. IR (neat) 3455, 3255, 3110, 2914, 2856, 2116, 1768, 1703, 1617, 1534, 1479, 1396, 1350, 1289, 1259, 1196, 1118, 1026, 775, 747 cm^–1^; ^1^H NMR (594 MHz, DMSO-*d*_6_): δ 11.16 (s, 1H), 10.16 (s, 1H), 8.48 (d, *J* = 7.9 Hz, 1H), 7.87 (t, *J* = 7.9 Hz, 1H), 7.66 (d, *J* = 7.9 Hz, 1H), 5.15 (m, 1H), 4.32 (s, 2H), 2.89 (m, 1H), 2.61 (m, 1H), 2.55 (m, 1H), 2.07 (m, 1H); ^13^C NMR (149 MHz, DMSO-*d*_6_): δ 172.8 (C), 169.8 (C), 167.6 (C), 167.1 (C), 166.6 (C), 136.3 (CH), 135.6 (C), 131.6 (C), 126.1 (CH), 119.0 (CH), 117.5 (C), 51.9 (CH_2_), 49.0 (CH), 30.9 (CH_2_), 22.0 (CH_2_); HRMS (FAB) *m*/*z*: [M + H]^+^ calcd for C_15_H_13_N_6_O_5_, 357.0947; found, 357.0946.

##### 4-Azido-*N*-(2-(2,6-dioxopiperidin-3-yl)-1,3-dioxoisoindolin-4-yl)­butanamide (**28**)

Following general procedure B, compound **28** was prepared in 91% yield as a cream solid using pomalidomide. IR (ATR) 3855, 3748, 3651, 2102, 1780, 1722, 1625, 1522, 1483, 1354, 1260, 1206, 896 cm^–1^; ^1^H NMR (399 MHz, DMSO-*d*_6_): δ 11.15 (s, 1H), 9.78 (s, 1H), 8.42 (d, *J* = 7.9 Hz, 1H), 7.83 (t, *J* = 7.9 Hz, 1H), 7.62 (d, *J* = 7.9 Hz, 1H), 5.14 (dd, *J* = 12.7, 5.4 Hz, 1H), 3.42 (t, *J* = 7.0 Hz, 2H), 2.90 (m, 1H), 2.63–2.52 (m, 4H), 2.07 (m, 1H), 1.88 (quint, *J* = 7.0 Hz, 2H); ^13^C NMR (100 MHz, DMSO-*d*_6_): δ 172.8 (C), 171.3 (C), 169.8 (C), 167.5 (C), 166.7 (C), 136.3 (CH), 136.1 (C), 131.5 (C), 126.7 (CH), 118.5 (CH), 117.4 (C), 50.1 (CH_2_), 48.9 (CH), 33.4 (CH_2_), 30.9 (CH_2_), 24.1 (CH_2_), 22.0 (CH_2_); HRMS (FAB) *m*/*z*: [M + H]^+^ calcd for C_17_H_17_N_6_O_5_, 385.1255; found, 385.1270.

##### 6-Azido-*N*-(2-(2,6-dioxopiperidin-3-yl)-1,3-dioxoisoindolin-4-yl)­hexanamide (**29**)

Following general procedure B, compound **29** was prepared in 85% yield as a cream solid using pomalidomide. IR (neat) 3353, 3226, 3107, 2953, 2097, 1778, 1712, 1621, 1537, 1479, 1426, 1398, 1350, 1322, 1259, 1197, 741 cm^–1^; ^1^H NMR (399 MHz, CDCl_3_): δ 9.42 (s, 1H), 8.82 (d, *J* = 7.9 Hz, 1H), 8.19 (s, 1H), 7.72 (t, *J* = 7.9 Hz, 1H), 7.55 (d, *J* = 7.9 Hz, 1H), 4.96 (dd, *J* = 12.1, 5.4 Hz, 1H), 3.30 (t, *J* = 7.2 Hz, 2H), 2.97–2.69 (m, 3H), 2.48 (t, *J* = 7.2 Hz, 2H), 2.17 (m, 1H), 1.79 (quint, *J* = 7.2 Hz, 2H), 1.66 (quint, *J* = 7.2 Hz, 2H), 1.49 (m, 2H); ^13^C NMR (100 MHz, CDCl_3_): δ 172.1 (C), 170.8 (C), 169.3 (C), 167.9 (C), 166.8 (C), 138.0 (C), 136.6 (CH), 131.2 (C), 125.4 (CH), 118.7 (CH), 115.5 (C), 51.3 (CH_2_), 49.4 (CH), 37.8 (CH_2_), 31.5 (CH_2_), 28.7 (CH_2_), 26.4 (CH_2_), 24.8 (CH_2_), 22.8 (CH_2_); HRMS (FAB) *m*/*z*: [M + H]^+^ calcd for C_19_H_21_N_6_O_5_, 413.1568; found, 413.1578.

##### 8-Azido-*N*-(2-(2,6-dioxopiperidin-3-yl)-1,3-dioxoisoindolin-4-yl)­octanamide (**30**)

Following general procedure B, compound **30** was prepared in 77% yield as a cream solid using pomalidomide. IR (neat) 3357, 3228, 2928, 2857, 2097, 1777, 1712, 1620, 1534, 1480, 1397, 1349, 1322, 1259, 1197, 741 cm^–1^; ^1^H NMR (399 MHz, CDCl_3_): δ 9.41 (s, 1H), 8.83 (d, *J* = 8.0 Hz, 1H), 8.0 (s, 1H), 7.71 (t, *J* = 8.0 Hz, 1H), 7.55 (d, *J* = 8.0 Hz, 1H), 4.95 (m, 1H), 3.26 (t, *J* = 7.1 Hz, 2H), 2.97–2.69 (m, 3H), 2.46 (t, *J* = 7.4 Hz, 2H), 2.17 (m, 1H), 1.76 (quint, *J* = 7.1 Hz, 2H), 1.60 (quint, 7.4 Hz, 2H), 1.40 (m, 6H); ^13^C NMR (100 MHz, CDCl_3_): δ 172.4 (C), 170.8 (C), 169.3 (C), 168.0 (C), 166.8 (C), 138.0 (C), 136.6 (CH), 131.2 (C), 125.4 (CH), 118.6 (CH), 115.4 (C), 51.6 (CH_2_), 49.4 (CH), 38.0 (CH_2_), 31.5 (CH_2_), 29.1 (CH_2_), 28.93 (CH_2_), 28.89 (CH_2_), 26.7 (CH_2_), 25.2 (CH_2_), 22.8 (CH_2_); HRMS (FAB) *m*/*z*: [M + H]^+^ calcd for C_21_H_25_N_6_O_5_, 441.1881; found, 441.1894.

##### 11-Azido-*N*-(2-(2,6-dioxopiperidin-3-yl)-1,3-dioxoisoindolin-4-yl)­undecanamide (**31**)

Following general procedure B, compound **31** was prepared in 94% yield as a cream solid using pomalidomide. IR (neat) 3355, 3227, 2927, 2857, 2096, 1778, 1703, 1617, 1527, 1479, 1398, 1349, 1260, 1197, 747 cm^–1^; ^1^H NMR (399 MHz, CDCl_3_): δ 9.40 (s, 1H), 8.83 (d, *J* = 8.6 Hz, 1H), 8.23 (s, 1H), 7.71 (dd, *J* = 8.6, 7.3 Hz, 1H), 7.54 (d, *J* = 7.3 Hz, 1H), 4.95 (m, 1H), 3.25 (t, *J* = 7.3 Hz, 2H), 2.97–2.68 (m, 3H), 2.45 (t, *J* = 7.3 Hz, 2H), 2.17 (m, 1H), 1.74 (quint, *J* = 7.3 Hz, 2H), 1.59 (quint, *J* = 7.3 Hz, 2H), 1.43–1.26 (m, 12H); ^13^C NMR (100 MHz, CDCl_3_): δ 172.6 (C), 170.9 (C), 169.3 (C), 168.0 (C), 166.9 (C), 138.1 (C), 136.6 (CH), 131.2 (C), 125.5 (CH), 118.6 (CH), 115.4 (C), 51.6 (CH_2_), 49.4 (CH), 38.1 (CH_2_), 31.5 (CH_2_), 29.5 (CH_2_), 29.4 (CH_2_), 29.3 (CH_2_), 29.23 (CH_2_), 29.22 (CH_2_), 29.0 (CH_2_), 26.8 (CH_2_), 25.4 (CH_2_), 22.8 (CH_2_); HRMS (FAB) *m*/*z*: [M + H]^+^ calcd for C_24_H_31_N_6_O_5_, 483.2350; found, 483.2359.

##### 6-Azido-*N*-(2-(1-methyl-2,6-dioxopiperidin-3-yl)-1,3-dioxoisoindolin-4-yl)­hexanamide (**32**)

Following general procedure B, compound **32** was prepared in 57% yield as a cream solid using *N*-methyl pomalidomide. IR (neat) 3357, 2939, 2097, 1769, 1705, 1682, 1617, 1526, 1479, 1397, 1350, 1326, 1289, 1120, 747 cm^–1^; ^1^H NMR (399 MHz, CDCl_3_): δ 9.43 (s, 1H), 8.82 (d, *J* = 8.1 Hz, 1H), 7.71 (d, *J* = 8.1 Hz, 1H), 7.54 (t, *J* = 8.1 Hz, 1H), 4.95 (m, 1H), 3.29 (t, *J* = 6.8 Hz, 2H), 3.22 (s, 3H), 3.00 (m, 1H), 2.80 (m, 1H), 2.70 (m, 1H), 2.48 (t, *J* = 7.5 Hz, 2H), 2.12 (m, 1H), 1.79–1.74 (m, 2H), 1.71–1.58 (m, 2H), 1.54–1.40 (m, 2H); ^13^C NMR (100 MHz, CDCl_3_): δ 171.9 (C), 170.9 (C), 169.3 (C), 168.5 (C), 166.8 (C), 137.7 (C), 136.4 (CH), 131.1 (C), 125.2 (CH), 118.4 (CH), 115.4 (C), 51.2 (CH_2_), 50.0 (CH), 37.7 (CH_2_), 31.9 (CH_2_), 28.6 (CH_2_), 27.3 (CH_3_), 26.2 (CH_2_), 24.7 (CH_2_), 22.0 (CH_2_); HRMS (FAB) *m*/*z*: [M + H]^+^ calcd for C_20_H_23_N_6_O_5_, 427.1724; found, 427.1712.

### Synthesis of TKP-Compounds by CuAAC Reaction

#### General Procedure C: Click Reaction

CuTC (50 mol %) and azide (1.1 equiv) were added to an alkyne (1.0 equiv) in THF. After 5 h of stirring at room temperature, the reaction was quenched with aq. NH_4_Cl and aq. NH_3_ (1:1), and the mixture was extracted with CHCl_3_ twice. The combined organic extracts were dried over anhydrous Na_2_SO_4_. Filtration and evaporation in vacuo furnished the crude product, which was purified by PTLC (9:1 CHCl_3_/MeOH) to give TKPs.

##### **TKP-1**: (*E*)-14-(4-(5-((2-(3-(2-(2-Acetamidoethyl)-5-ethoxy-4-methoxyphenyl)-3-oxoprop-1-en-1-yl)­phenoxy)­methyl)­pyridin-2-yl)-1*H*-1,2,3-triazol-1-yl)-*N*-(2-(2,6-dioxopiperidin-3-yl)-1,3-dioxoisoindolin-4-yl)-3,6,9,12-tetraoxatetradecanamide

Following general procedure C, **TKP-1** was prepared as a yellow solid using **TK-351** and azide **33**. *R*_f_ = 0.60 (8:1 CHCl_3_/MeOH); IR (neat): 3849, 3741, 3730, 3583, 3315, 3076, 3010, 2917, 1769, 1707, 1651, 1618, 1566, 1529, 1481, 1455, 1429, 1397, 1350, 1324, 1294, 1262, 1198, 1131, 1042, 985, 917, 871, 823, 749, 666 cm^–1^; ^1^H NMR (594 MHz, CDCl_3_): δ 10.47 (s, 1H), 9.06 (s, 1H), 8.83 (dd, *J* = 8.5, 0.8 Hz, 1H), 8.64 (dd, *J* = 2.3, 0.8 Hz, 1H), 8.37 (s, 1H), 8.16 (dd, *J* = 7.7, 0.8 Hz, 1H), 7.97 (d, *J* = 16.1 Hz, 1H), 7.78 (dd, *J* = 7.7, 2.3 Hz, 1H), 7.70 (dd, *J* = 8.5, 7.7 Hz, 1H), 7.62 (dd, *J* = 7.8, 1.7 Hz, 1H), 7.56 (dd, *J* = 7.7, 0.8 Hz, 1H), 7.37 (ddd, *J* = 8.3, 7.4, 1.7 Hz, 1H), 7.23 (d, *J* = 16.1 Hz, 1H), 7.17 (br, 1H), 7.06–6.96 (m, 3H), 6.80 (s, 1H), 5.19 (s, 2H), 4.95 (m, 1H), 4.64–4.60 (m, 2H), 4.17 (m, 2H), 4.02 (q, *J* = 7.0 Hz, 2H), 3.98–3.90 (m, 2H), 3.91 (s, 3H), 3.77 (m, 4H), 3.69–3.58 (m, 8H), 3.51 (m, 2H), 2.87 (m, 3H), 2.83–2.70 (m, 2H), 2.15 (m, 1H), 1.91 (s, 3H), 1.39 (t, *J* = 7.0 Hz, 3H); ^13^C NMR (149 MHz, CDCl_3_): δ 195.7 (C), 171.3 (C), 170.7 (C), 169.5 (C), 168.6 (C), 168.3 (C), 166.9 (C), 157.4 (C), 152.0 (C), 150.6 (C), 148.5 (CH), 147.9 (C), 146.2 (C), 141.4 (CH), 136.9 (C), 136.4 (CH), 136.2 (CH), 133.4 (C), 132.3 (CH), 131.6 (C), 131.4 (C), 131.1 (C), 129.1 (CH), 127.1 (CH), 125.3 (CH), 124.2 (C), 123.7 (CH), 121.8 (CH), 120.3 (CH), 118.9 (CH), 116.3 (C), 113.8 (CH), 113.6 (CH), 113.0 (CH), 71.7 (CH_2_), 71.1 (CH_2_), 70.9 (CH_2_), 70.8 (CH_2_), 70.7 (CH_2_), 70.6 (CH_2_), 70.5 (CH_2_), 69.5 (CH_2_), 68.2 (CH_2_), 64.9 (CH_2_), 56.2 (CH_3_), 50.6 (CH_2_), 49.4 (CH), 42.1 (CH_2_), 31.9 (CH_2_), 31.6 (CH_2_), 23.4 (CH_3_), 22.9 (CH_2_), 14.9 (CH_3_); HRMS (FAB) *m*/*z*: [M + H]^+^ calcd for C_53_H_59_N_8_O_14_, 1031.4145; found, 1031.4153.

##### **TKP-3**: (*E*)-6-(4-(5-((2-(3-(2-(2-Acetamidoethyl)-5-ethoxy-4-methoxyphenyl)-3-oxoprop-1-en-1-yl)­phenoxy)­methyl)­pyridin-2-yl)-1*H*-1,2,3-triazol-1-yl)-*N*-(2-(2,6-dioxopiperidin-3-yl)-1,3-dioxoisoindolin-4-yl)­hexanamide

Following general procedure C, **TKP-3** was prepared as a yellow solid using **TK-351** and azide **29**. *R*_f_ = 0.53 (8:1 CHCl_3_/MeOH); IR (neat): 3357, 3073, 3013, 2981, 2935, 2858, 1768, 1704, 1657, 1618, 1600, 1567, 1525, 1480, 1455, 1428, 1398, 1350, 1324, 1294, 1262, 1197, 1160, 1131, 1041, 1026, 985, 920, 865, 822, 749, 666 cm^–1^; ^1^H NMR (594 MHz, CDCl_3_): δ 9.48 (s, 1H), 9.24 (s, 1H), 8.79 (dd, *J* = 8.5, 0.8 Hz, 1H), 8.65 (dd, *J* = 2.3, 0.8 Hz, 1H), 8.29 (s, 1H), 8.17 (dd, *J* = 7.7, 0.8 Hz, 1H), 7.97 (d, *J* = 16.1 Hz, 1H), 7.79 (dd, *J* = 7.8, 2.3 Hz, 1H), 7.71 (dd, *J* = 8.5, 7.7 Hz, 1H), 7.63 (dd, *J* = 7.8, 1.7 Hz, 1H), 7.55 (dd, *J* = 7.8, 0.8 Hz, 1H), 7.39 (ddd, *J* = 8.3, 7.4, 1.7 Hz, 1H), 7.24 (d, *J* = 16.1 Hz, 1H), 7.15 (br, 1H), 7.07–6.99 (m, 3H), 6.79 (s, 1H), 5.20 (s, 2H), 4.96 (m, 1H), 4.43 (m, 2H), 4.01 (q, *J* = 7.0 Hz, 2H), 3.91 (s, 3H), 3.54–3.47 (m, 2H), 2.97–2.84 (m, 3H), 2.83–2.72 (m, 2H), 2.56–2.44 (m, 2H), 2.17 (m, 1H), 2.05 (quint, *J* = 7.6 Hz, 2H), 1.90 (s, 3H), 1.84 (m, 2H), 1.49 (m, 2H), 1.39 (t, *J* = 7.0 Hz, 3H); ^13^C NMR (149 MHz, CDCl_3_): δ 195.8 (C), 171.8 (C), 171.1 (C), 170.7 (C), 169.4 (C), 168.4 (C), 166.8 (C), 157.4 (C), 152.0 (C), 150.5 (C), 148.5 (CH), 148.0 (C), 146.2 (C), 141.5 (CH), 137.8 (C), 136.6 (CH), 136.4 (CH), 133.3 (C), 132.3 (CH), 131.5 (C), 131.3 (C), 131.2 (C), 129.2 (CH), 127.2 (CH), 125.5 (CH), 124.2 (C), 122.5 (CH), 121.8 (CH), 120.4 (CH), 118.7 (CH), 115.6 (C), 113.8 (CH), 113.6 (CH), 113.0 (CH), 68.2 (CH_2_), 64.9 (CH_2_), 56.2 (CH_3_), 50.4 (CH_2_), 49.5 (CH), 42.1 (CH_2_), 37.5 (CH_2_), 31.9 (CH_2_), 31.6 (CH_2_), 30.3 (CH_2_), 25.9 (CH_2_), 24.8 (CH_2_), 23.4 (CH_3_), 23.0 (CH_2_), 14.9 (CH_3_); HRMS (FAB) *m*/*z*: [M + H]^+^ calcd for C_49_H_51_N_8_O_10_, 911.3723; found, 911.3710.

##### **TKP-4**: (*E*)-2-(4-(5-((2-(3-(2-(2-Acetamidoethyl)-5-ethoxy-4-methoxyphenyl)-3-oxoprop-1-en-1-yl)­phenoxy)­methyl)­pyridin-2-yl)-1*H*-1,2,3-triazol-1-yl)-*N*-(2-(2,6-dioxopiperidin-3-yl)-1,3-dioxoisoindolin-4-yl)­acetamide

Following general procedure C, **TKP-4** was prepared as a yellow solid using **TK-351** and azide **27**. *R*_f_ = 0.40 (8:1 CHCl_3_/MeOH); IR (neat): 3314, 1771, 1706, 1653, 1619, 1540, 1480, 1456, 1397, 1349, 1261, 1196, 1130, 1041, 748 cm^–1^; ^1^H NMR (594 MHz, CDCl_3_): δ 9.68 (s, 1H), 8.76 (br, 1H), 8.71 (d, *J* = 8.2 Hz, 1H), 8.63 (d, *J* = 2.2 Hz, 1H), 8.41 (s, 1H), 8.19 (d, *J* = 8.2 Hz, 1H), 7.96 (d, *J* = 16.1 Hz, 1H), 7.79 (dd, *J* = 7.8, 2.2 Hz, 1H), 7.70 (t, *J* = 8.2 Hz, 1H), 7.63 (dd, *J* = 7.7, 1.7 Hz, 1H), 7.56 (d, *J* = 7.8 Hz, 1H), 7.39 (dd, *J* = 7.9, 1.7 Hz, 1H), 7.21 (d, *J* = 16.1 Hz, 1H), 7.10–6.99 (m, 4H), 6.78 (s, 1H), 5.41 (m, 2H), 5.18 (s, 2H), 4.89 (m, 1H), 4.01 (q, *J* = 7.0 Hz, 2H), 3.89 (s, 3H), 3.52–3.46 (m, 2H), 2.87–2.77 (m, 3H), 2.72–2.64 (m, 2H), 2.08 (m, 1H), 1.88 (s, 3H), 1.39 (t, *J* = 7.0 Hz, 3H); ^13^C NMR (149 MHz, CDCl_3_): δ 195.8 (C), 171.0 (C), 170.7 (C), 168.7 (C), 168.1 (C), 166.6 (C), 164.3 (C), 157.4 (C), 151.9 (C), 150.0 (C), 149.1 (C), 148.7 (CH), 146.2 (C), 141.4 (CH), 136.6 (CH), 136.4 (C), 136.2 (CH), 133.3 (C), 132.3 (CH), 131.5 (C), 131.40 (C), 131.38 (C), 129.0 (CH), 127.1 (CH), 125.4 (CH), 124.23 (CH), 124.19 (C), 121.9 (CH), 120.5 (CH), 119.6 (CH), 116.6 (C), 113.8 (CH), 113.6 (CH), 113.0 (CH), 68.2 (CH_2_), 64.9 (CH_2_), 56.2 (CH_3_), 53.8 (CH_2_), 49.5 (CH), 42.0 (CH_2_), 32.0 (CH_2_), 31.5 (CH_2_), 23.3 (CH_3_), 22.6 (CH_2_), 14.9 (CH_3_); HRMS (FAB) *m*/*z*: [M + H]^+^ calcd for C_45_H_43_N_8_O_10_, 855.3097; found, 855.3083.

##### **TKP-5**: (*E*)-6-(4-((4-(3-(2-(2-Acetamidoethyl)-5-ethoxy-4-methoxyphenyl)-3-oxoprop-1-en-1-yl)­phenoxy)­methyl)-1*H*-1,2,3-triazol-1-yl)-*N*-(2-(2,6-dioxopiperidin-3-yl)-1,3-dioxoisoindolin-4-yl)­hexanamide

Following general procedure C, **TKP-5** was prepared as a yellow solid using alkyne **5** and azide **29**. *R*_f_ = 0.45 (8:1 CHCl_3_/MeOH); IR (neat): 3356, 2932, 2862, 1769, 1704, 1655, 1619, 1598, 1571, 1509, 1478, 1424, 1397, 1350, 1291, 1260, 1199, 1175, 1130, 1029, 990, 823, 748, 666 cm^–1^; ^1^H NMR (594 MHz, CDCl_3_): δ 9.39 (s, 1H), 8.79 (dd, *J* = 8.6, 0.8 Hz, 1H), 8.42 (s, 1H), 7.70 (dd, *J* = 8.6, 7.4 Hz, 1H), 7.63 (s, 1H), 7.56–7.50 (m, 4H), 7.21 (s, 1H), 7.06–6.99 (m, 4H), 6.81 (s, 1H), 5.24 (s, 2H), 4.95 (dd, *J* = 12.4, 5.4 Hz, 1H), 4.38 (t, *J* = 7.3 Hz, 2H), 4.09 (q, *J* = 7.0 Hz, 2H), 3.92 (s, 3H), 3.54–3.48 (m, 2H), 2.91 (m, 1H), 2.87–2.84 (m, 2H), 2.84–2.71 (m, 2H), 2.45 (t, *J* = 7.4 Hz, 2H), 2.17 (m, 1H), 1.98 (quint, *J* = 7.3 Hz, 2H), 1.91 (s, 3H), 1.79 (quint, *J* = 7.4 Hz, 2H), 1.46 (t, *J* = 7.0 Hz, 3H),1.44–1.38 (m, 2H); ^13^C NMR (149 MHz, CDCl_3_): δ 195.6 (C), 171.8 (C), 170.8 (C), 170.7 (C), 169.4 (C), 168.0 (C), 166.7 (C), 160.7 (C), 152.0 (C), 146.22 (CH), 146.18 (C), 143.7 (C), 137.8 (C), 136.6 (CH), 133.3 (C), 131.5 (C), 131.3 (C), 130.5 (CH), 127.8 (C), 125.4 (CH), 124.4 (CH), 122.9 (CH), 118.7 (CH), 115.52 (CH), 115.51 (C), 113.8 (CH), 113.6 (CH), 65.0 (CH_2_), 62.3 (CH_2_), 56.2 (CH_3_), 50.3 (CH_2_), 49.5 (CH), 42.1 (CH_2_), 37.5 (CH_2_), 31.8 (CH_2_), 31.5 (CH_2_), 30.1 (CH_2_), 26.0 (CH_2_), 24.5 (CH_2_), 23.3 (CH_3_), 22.8 (CH_2_), 14.9 (CH_3_); HRMS (FAB) *m*/*z*: [M + H]^+^ calcd for C_44_H_48_N_7_O_10_, 834.3457; found, 834.3460.

##### **TKP-6**: (*E*)-6-(4-((2-(3-(2-(2-Acetamidoethyl)-5-ethoxy-4-methoxyphenyl)-3-oxoprop-1-en-1-yl)­phenoxy)­methyl)-1*H*-1,2,3-triazol-1-yl)-*N*-(2-(2,6-dioxopiperidin-3-yl)-1-oxoisoindolin-4-yl) Hexanamide

Following general procedure C, **TKP-6** was prepared as a yellow solid using alkyne **3** and azide **29**. *R*_f_ = 0.48 (8:1 CHCl_3_/MeOH); IR (neat): 3345, 3097, 2929, 2849, 1767, 1704, 1658, 1619, 1600, 1523, 1479, 1397, 1350, 1261, 1197, 1130, 994, 748 cm^–1^; ^1^H NMR (594 MHz, CDCl_3_): δ 9.39 (s, 1H), 8.78 (dd, *J* = 8.5, 0.8 Hz, 1H), 8.61 (s, 1H), 7.89 (d, *J* = 16.1 Hz, 1H), 7.69 (dd, *J* = 8.5, 7.3 Hz, 1H), 7.59 (dd, *J* = 7.7, 1.7 Hz, 1H), 7.54 (s, 1H), 7.53 (dd, *J* = 7.3, 0.8 Hz, 2H), 7.39 (ddd, *J* = 8.4, 7.4, 1.7 Hz, 1H), 7.20 (d, *J* = 16.1 Hz, 1H), 7.16 (m, 1H), 7.10 (dd, *J* = 8.4, 1.0 Hz, 1H), 7.04 (s, 1H), 7.02 (ddd, *J* = 7.7, 7.4, 1.0 Hz, 1H), 6.82 (s, 1H), 5.27 (s, 2H), 4.95 (dd, *J* = 12.4, 5.4 Hz, 1H), 4.38 (t, *J* = 7.2 Hz, 2H), 4.02 (q, *J* = 7.0 Hz, 2H), 3.91 (s, 3H), 3.54–3.49 (m, 2H), 2.92 (m, 1H), 2.89–2.84 (m, 2H), 2.84–2.71 (m, 2H), 2.45 (t, *J* = 7.4 Hz, 2H), 2.17 (m, 1H), 1.95 (quint, *J* = 7.2 Hz, 2H), 1.91 (s, 3H), 1.78 (quint, *J* = 7.4 Hz, 2H), 1.44–1.36 (m, 2H), 1.40 (t, *J* = 7.0 Hz, 3H); ^13^C NMR (149 MHz, CDCl_3_): δ 196.1 (C), 171.8 (C), 170.9 (C), 170.7 (C), 169.3 (C), 168.1 (C), 166.8 (C), 157.4 (C), 151.9 (C), 146.1 (C), 143.8 (C), 141.9 (CH), 137.8 (C), 136.6 (CH), 133.5 (C), 132.4 (CH), 131.5 (C), 131.3 (C), 129.2 (CH), 127.1 (CH), 125.4 (CH), 123.9 (C), 122.7 (CH), 121.7 (CH), 118.7 (CH), 115.5 (C), 114.0 (CH), 113.9 (CH), 113.0 (CH), 65.0 (CH_2_), 62.9 (CH_2_), 56.2 (CH_3_), 50.2 (CH_2_), 49.5 (CH), 42.0 (CH_2_), 37.6 (CH_2_), 31.9 (CH_2_), 31.5 (CH_2_), 30.1 (CH_2_), 26.1 (CH_2_), 24.6 (CH_2_), 23.3 (CH_3_), 22.8 (CH_2_), 14.9 (CH_3_); HRMS (FAB) *m*/*z*: [M + H]^+^ calcd for C_40_H_40_N_7_O_10_, 778.2831; found, 778.2816.

##### **TKP-7**: (*E*)-2-(4-((4-(3-(2-(2-Acetamidoethyl)-5-ethoxy-4-methoxyphenyl)-3-oxoprop-1-en-1-yl)­phenoxy)­methyl)-1*H*-1,2,3-triazol-1-yl)-*N*-(2-(2,6-dioxopiperidin-3-yl)-1-oxoisoindolin-4-yl) Acetamide

Following general procedure C, **TKP-7** was prepared as a yellow solid using alkyne **5** and azide **27**. *R*_f_ = 0.43 (8:1 CHCl_3_/MeOH); IR (neat): 3311, 2930, 2856, 1771, 1707, 1653, 1622, 1599, 1540, 1508, 1479, 1396, 1349, 1260, 1199, 1175, 1130, 1029, 823, 747 cm^–1^; ^1^H NMR (594 MHz, CDCl_3_): δ 9.63 (s, 1H), 8.86 (s, 1H), 8.67 (dd, *J* = 8.5, 0.7 Hz, 1H), 7.89 (s, 1H), 7.67 (dd, *J* = 8.5, 7.4 Hz, 1H), 7.54 (dd, *J* = 7.4, 0.7 Hz, 1H), 7.53–7.47 (m, 3H), 7.19 (m, 1H), 7.05–6.97 (m, 4H), 6.80 (s, 1H), 5.40–5.30 (m, 2H), 5.27 (s, 2H), 4.91 (m, 1H), 4.08 (q, *J* = 7.0 Hz, 2H), 3.90 (s, 3H), 3.53–3.47 (m, 2H), 2.88–2.82 (m, 2H), 2.82 (m, 1H), 2.76–2.67 (m, 2H), 2.10 (m, 1H), 1.90 (s, 3H), 1.44 (t, *J* = 7.0 Hz, 3H); ^13^C NMR (149 MHz, CDCl_3_): δ 195.4 (C), 171.2 (C), 170.7 (C), 168.7 (C), 168.3 (C), 166.5 (C), 164.4 (C), 160.6 (C), 151.9 (C), 146.2 (CH), 146.0 (C), 144.6 (C), 136.6 (C), 136.3 (CH), 133.3 (C), 131.5 (C), 131.3 (C), 130.5 (CH), 127.9 (C), 125.4 (CH), 124.8 (CH), 124.4 (CH), 119.6 (CH), 116.5 (CH), 115.5 (C), 113.8 (CH), 113.7 (CH), 65.0 (CH_2_), 62.1 (CH_2_), 56.1 (CH_3_), 53.5 (CH_2_), 49.5 (CH), 42.0 (CH_2_), 31.9 (CH_2_), 31.5 (CH_2_), 23.3 (CH_3_), 22.6 (CH_2_), 14.9 (CH_3_); HRMS (FAB) *m*/*z*: [M + H]^+^ calcd for C_40_H_40_N_7_O_10_, 778.2831; found, 778.2849.

##### **TKP-8**: (*E*)-2-(4-((2-(3-(2-(2-Acetamidoethyl)-5-ethoxy-4-methoxyphenyl)-3-oxoprop-1-en-1-yl)­phenoxy)­methyl)-1*H*-1,2,3-triazol-1-yl)-*N*-(2-(2,6-dioxopiperidin-3-yl)-1,3-dioxoisoindolin-4-yl)­acetamide

Following general procedure C, **TKP-8** was prepared as a yellow solid using alkyne **3** and azide **27**. *R*_f_ = 0.43 (8:1 CHCl_3_/MeOH); IR (neat): 3357, 2935, 2848, 1768, 1705, 1661, 1618, 1598, 1529, 1477, 1394, 1349, 1261, 1200, 1127, 1028, 994, 771, 748 cm^–1^; ^1^H NMR (594 MHz, CDCl_3_): δ 9.57 (s, 1H), 8.72 (d, *J* = 8.5 Hz, 1H), 8.67 (s, 1H), 7.88 (d, *J* = 16.3 Hz, 1H), 7.74 (s, 1H), 7.71 (dd, *J* = 8.5, 7.3 Hz, 1H), 7.62 (dd, *J* = 7.8, 1.7 Hz, 1H), 7.57 (d, *J* = 7.3 Hz, 1H), 7.42 (m, 1H), 7.14 (d, *J* = 16.3 Hz, 1H), 7.12–7.08 (m, 2H), 7.08–7.01 (m, 2H), 6.79 (s, 1H), 5.46–5.40 (m, 2H), 5.33 (s, 2H), 4.89 (dd, *J* = 12.4, 5.4 Hz, 1H), 3.99 (q, *J* = 7.0 Hz, 2H), 3.90 (s, 3H), 3.49–3.46 (m, 2H), 2.86 (m, 1H), 2.81 (t, *J* = 6.6 Hz, 2H), 2.77–2.66 (m, 2H), 2.11 (m, 1H), 1.89 (s, 3H), 1.36 (t, *J* = 7.0 Hz, 3H); ^13^C NMR (149 MHz, CDCl_3_): δ 196.6 (C), 171.0 (C), 170.7 (C), 168.7 (C), 168.1 (C), 166.6 (C), 164.6 (C), 157.2 (C), 151.8 (C), 145.8 (C), 144.9 (C), 142.2 (CH), 136.6 (CH), 136.5 (C), 133.6 (C), 132.6 (CH), 131.4 (C), 131.3 (C), 128.6 (CH), 127.4 (CH), 125.3 (CH), 124.3 (CH), 123.7 (C), 121.8 (CH), 119.6 (CH), 116.5 (C), 114.7 (CH), 114.0 (CH), 112.7 (CH), 65.4 (CH_2_), 63.0 (CH_2_), 56.2 (CH_3_), 53.4 (CH_2_), 49.5 (CH), 41.9 (CH_2_), 31.9 (CH_2_), 31.5 (CH_2_), 23.3 (CH_3_), 22.7 (CH_2_), 14.9 (CH_3_); HRMS (FAB) *m*/*z*: [M + H]^+^ calcd for C_40_H_40_N_7_O_10_, 778.2831; found, 778.2849.

##### **TKP-9**: (*E*)-4-(4-((4-(3-(2-(2-Acetamidoethyl)-5-ethoxy-4-methoxyphenyl)-3-oxoprop-1-en-1-yl)­phenoxy)­methyl)-1*H*-1,2,3-triazol-1-yl)-*N*-(2-(2,6-dioxopiperidin-3-yl)-1,3-dioxoisoindolin-4-yl)­butanamide

Following general procedure C, **TKP-9** was prepared as a yellow solid using alkyne **5** and azide **28**. *R*_f_ = 0.45 (8:1 CHCl_3_/MeOH); IR (neat) 3352, 3085, 2931, 2849, 1766, 1704, 1655, 1619, 1598, 1509, 1479, 1397, 1350, 1260, 1198, 1130, 988, 823, 748 cm^–1^; ^1^H NMR (594 MHz, CDCl_3_): δ 9.39 (s, 1H), 8.76 (dd, *J* = 8.5, 0.7 Hz, 1H), 8.27 (s, 1H), 7.72 (dd, *J* = 8.5, 6.7 Hz, 1H), 7.69 (s, 1H), 7.60–7.50 (m, 4H), 7.21 (m, 1H), 7.07–7.00 (m, 4H), 6.81 (s, 1H), 5.25 (s, 2H), 4.95 (dd, *J* = 12.4, 5.4 Hz, 1H), 4.52 (t, *J* = 6.8 Hz, 2H), 4.10 (q, *J* = 7.0 Hz, 2H), 3.92 (s, 3H), 3.55–3.49 (m, 2H), 2.91 (dd, *J* = 15.9, 3.7 Hz, 1H), 2.86 (t, *J* = 6.4 Hz, 2H), 2.83–2.71 (m, 2H), 2.52 (t, *J* = 6.9 Hz, 2H), 2.40–2.33 (m, 2H), 2.17 (m, 1H), 1.92 (s, 3H), 1.46 (t, *J* = 7.0 Hz, 3H); ^13^C NMR (149 MHz, CDCl_3_): δ 195.6 (C), 170.72 (C), 170.69 (C), 170.62 (C), 169.2 (C), 167.9 (C), 166.7 (C), 160.7 (C), 152.0 (C), 146.3 (CH), 146.2 (C), 143.9 (C), 137.6 (C), 136.6 (CH), 133.3 (C), 131.5 (C), 131.3 (C), 130.5 (CH), 127.9 (C), 125.4 (CH), 124.4 (CH), 123.2 (CH), 118.9 (CH), 115.7 (CH), 115.5 (C), 113.8 (CH), 113.6 (CH), 65.1 (CH_2_), 62.2 (CH_2_), 56.2 (CH_3_), 49.5 (CH_2_), 49.4 (CH), 42.1 (CH_2_), 33.8 (CH_2_), 31.8 (CH_2_), 31.5 (CH_2_), 25.5 (CH_2_), 23.3 (CH_3_), 22.8 (CH_2_), 14.9 (CH_3_); HRMS (FAB) *m*/*z*: [M + H]^+^ calcd for C_42_H_44_N_7_O_10_, 806.3144; found, 806.3159.

##### **TKP-10**: (*E*)-4-(4-((2-(3-(2-(2-Acetamidoethyl)-5-ethoxy-4-methoxyphenyl)-3-oxoprop-1-en-1-yl)­phenoxy)­methyl)-1*H*-1,2,3-triazol-1-yl)-*N*-(2-(2,6-dioxopiperidin-3-yl)-1,3-dioxoisoindolin-4-yl)­butanamide

Following general procedure C, **TKP-10** was prepared as a yellow solid using alkyne **3** and azide **28**. *R*_f_ = 0.49 (8:1 CHCl_3_/MeOH); IR (neat) 3316, 3087, 2980, 2945, 2856, 1771, 1708, 1656, 1621, 1598, 1538, 1480, 1455, 1397, 1349, 1325, 1295, 1261, 1198, 1162, 1130, 1029, 992, 822, 748, 666 cm^–1^; ^1^H NMR (594 MHz, CDCl_3_): δ 9.37 (s, 1H), 8.74 (dd, *J* = 8.5, 0.8 Hz, 1H), 8.52 (s, 1H), 7.89 (d, *J* = 16.2 Hz, 1H), 7.70 (dd, *J* = 8.5, 7.3 Hz, 1H), 7.60 (dd, *J* = 7.7, 1.7 Hz, 1H), 7.58 (s, 1H), 7.53 (dd, *J* = 7.3, 0.8 Hz, 1H), 7.39 (ddd, *J* = 8.4, 7.6, 1.7 Hz, 1H), 7.20 (d, *J* = 16.2 Hz, 1H), 7.14 (br, 1H), 7.10 (dd, *J* = 8.4, 1.0 Hz, 1H), 7.04 (s, 1H), 7.03 (ddd, *J* = 7.7, 7.6, 1.0 Hz, 1H), 6.82 (s, 1H), 5.27 (s, 2H), 4.93 (m, 1H), 4.51 (t, *J* = 6.9 Hz, 2H), 4.02 (q, *J* = 7.0 Hz, 2H), 3.92 (s, 3H), 3.53–3.47 (m, 2H), 2.89 (m, 1H), 2.85 (t, *J* = 6.5 Hz, 2H), 2.82–2.70 (m, 2H), 2.48 (t, *J* = 6.9 Hz, 2H), 2.33 (quint, *J* = 6.9 Hz, 2H), 2.16 (m, 1H), 1.90 (s, 3H), 1.40 (t, *J* = 7.0 Hz, 3H); ^13^C NMR (149 MHz, CDCl_3_): δ 196.2 (C), 170.9 (C), 170.7 (C), 170.7 (C), 169.2 (C), 168.0 (C), 166.7 (C), 157.4 (C), 151.8 (C), 146.0 (C), 144.0 (C), 141.9 (CH), 137.6 (C), 136.6 (CH), 133.5 (C), 132.4 (CH), 131.5 (C), 131.3 (C), 129.1 (CH), 127.2 (CH), 125.4 (CH), 123.9 (C), 123.0 (CH), 121.7 (CH), 118.9 (CH), 115.7 (C), 114.0 (CH), 114.0 (CH), 112.9 (CH), 65.1 (CH_2_), 62.9 (CH_2_), 56.2 (CH_3_), 49.5 (CH_2_), 49.4 (CH), 42.0 (CH_2_), 33.9 (CH_2_), 31.9 (CH_2_), 31.5 (CH_2_), 25.6 (CH_2_), 23.3 (CH_3_), 22.8 (CH_2_), 14.9 (CH_3_); HRMS (FAB) *m*/*z*: [M + H]^+^ calcd for C_42_H_44_N_7_O_10_, 806.3144; found, 806.3154.

##### **TKP-11**: (*E*)-4-(4-(5-((2-(3-(2-(2-Acetamidoethyl)-5-ethoxy-4-methoxyphenyl)-3-oxoprop-1-en-1-yl)­phenoxy)­methyl)­pyridin-2-yl)-1*H*-1,2,3-triazol-1-yl)-*N*-(2-(2,6-dioxopiperidin-3-yl)-1,3-dioxoisoindolin-4-yl)­butanamide

Following general procedure C, **TKP-11** was prepared as a yellow solid using **TK-351** and azide **28**. *R*_f_ = 0.46 (8:1 CHCl_3_/MeOH); IR (neat) 3345, 2926, 2849, 1768, 1705, 1657, 1617, 1524, 1479, 1397, 1350, 1261, 1197, 1130, 1032, 989, 822, 748 cm^–1^; ^1^H NMR (594 MHz, CDCl_3_): δ 9.41 (s, 1H), 8.82 (br, 1H), 8.75 (dd, *J* = 8.5, 0.8 Hz, 1H), 8.61 (dd, *J* = 2.3, 0.9 Hz, 1H), 8.23 (s, 1H), 8.15 (dd, *J* = 8.2, 0.9 Hz, 1H), 7.96 (d, *J* = 16.1 Hz, 1H), 7.78 (dd, *J* = 8.2, 2.3 Hz, 1H), 7.69 (dd, *J* = 8.5, 7.4 Hz, 1H), 7.63 (dd, *J* = 7.7, 1.7 Hz, 1H), 7.54 (dd, *J* = 7.4, 0.8 Hz, 1H), 7.39 (ddd, *J* = 8.9, 7.4, 1.7 Hz, 1H), 7.23 (d, *J* = 16.1 Hz, 1H), 7.16 (br, 1H), 7.05 (ddd, *J* = 8.9, 7.7, 1.0 Hz, 1H), 7.02 (dd, *J* = 7.4, 1.0 Hz, 1H), 7.01 (s, 1H), 6.79 (s, 1H), 5.19 (s, 2H), 4.93 (m, 1H), 4.59 (t, *J* = 6.9 Hz, 2H), 4.00 (q, *J* = 7.0 Hz, 2H), 3.90 (s, 3H), 3.51 (m, 2H), 2.89 (m, 1H), 2.85 (t, *J* = 6.4 Hz, 2H), 2.82–2.69 (m, 2H), 2.64–2.52 (m, 2H), 2.43 (quint, *J* = 6.9 Hz, 2H), 2.17 (m, 1H), 1.90 (s, 3H), 1.39 (t, *J* = 7.0 Hz, 3H); ^13^C NMR (149 MHz, CDCl_3_): δ 195.9 (C), 171.0 (C), 170.8 (C), 170.6 (C), 169.2 (C), 168.1 (C), 166.8 (C), 157.5 (C), 151.9 (C), 150.4 (C), 148.6 (CH), 148.3 (C), 146.2 (C), 141.4 (CH), 137.6 (C), 136.6 (CH), 136.3 (CH), 133.3 (C), 132.3 (CH), 131.5 (C), 131.3 (C), 131.2 (C), 129.2 (CH), 127.2 (CH), 125.5 (CH), 124.2 (C), 122.7 (CH), 121.8 (CH), 120.3 (CH), 118.8 (CH), 115.7 (C), 113.8 (CH), 113.5 (CH), 112.9 (CH), 68.2 (CH_2_), 64.9 (CH_2_), 56.2 (CH_3_), 49.54 (CH_2_), 49.50 (CH), 42.1 (CH_2_), 33.9 (CH_2_), 31.9 (CH_2_), 31.5 (CH_2_), 25.6 (CH_2_), 23.3 (CH_3_), 22.9 (CH_2_), 14.9 (CH_3_); HRMS (FAB) *m*/*z*: [M + H]^+^ calcd for C_47_H_47_N_8_O_10_, 883.3410; found, 883.3443.

##### **TKP-13**: (*E*)-6-(4-((4-(3-(2-(2-Acetamidoethyl)-5-ethoxy-4-methoxyphenyl)-3-oxoprop-1-en-1-yl)­phenoxy)­methyl)-1*H*-1,2,3-triazol-1-yl)-*N*-(2-(1-methyl-2,6-dioxopiperidin-3-yl)-1,3-dioxoisoindolin-4-yl)­hexanamide

Following general procedure C, **TKP-13** was prepared as a yellow solid using alkyne **5** and azide **32**. *R*_f_ = 0.57 (8:1 CHCl_3_/MeOH); IR (neat) 3358, 2935, 1768, 1705, 1681, 1618, 1598, 1570, 1509, 1479, 1424, 1397, 1349, 1326, 1289, 1259, 1175, 1157, 1129, 1050, 1029, 987, 823, 748, 666 cm^–1^; ^1^H NMR (399 MHz, CDCl_3_): δ 9.40 (s, 1H), 8.78 (dd, *J* = 8.5, 0.7 Hz, 1H), 7.69 (dd, *J* = 8.5, 7.3 Hz, 1H), 7.63 (s, 1H), 7.61–7.48 (m, 4H), 7.18 (br, 1H), 7.09–6.96 (m, 4H), 6.81 (s, 1H), 5.23 (s, 2H), 4.95 (dd, *J* = 12.3, 5.6 Hz, 1H), 4.39 (t, *J* = 7.1 Hz, 2H), 4.09 (q, *J* = 7.0 Hz, 2H), 3.92 (s, 3H), 3.56–3.47 (m, 2H), 3.21 (s, 3H), 2.98 (m, 1H), 2.87–2.84 (m, 2H), 2.79–2.74 (m, 2H), 2.45 (t, *J* = 7.4 Hz, 2H), 2.13 (m, 1H), 1.99 (quint, *J* = 7.1 Hz, 2H), 1.91 (s, 3H), 1.79 (quint, *J* = 7.4 Hz, 2H), 1.46 (t, *J* = 7.0 Hz, 3H), 1.46–1.39 (m, 2H); ^13^C NMR (100 MHz, CDCl_3_): δ 195.5 (C), 171.8 (C), 171.0 (C), 170.6 (C), 169.5 (C), 168.7 (C), 166.9 (C), 160.7 (C), 152.0 (C), 146.3 (C), 146.1 (CH), 143.7 (C), 137.8 (C), 136.5 (CH), 133.3 (C), 131.6 (C), 131.3 (C), 130.5 (CH), 127.8 (C), 125.3 (CH), 124.4 (CH), 122.8 (CH), 118.6 (CH), 115.6 (C), 115.5 (CH), 113.8 (CH), 113.7 (CH), 65.1 (CH_2_), 62.3 (CH_2_), 56.2 (CH_3_), 50.3 (CH_2_), 50.2 (CH), 42.1 (CH_2_), 37.5 (CH_2_), 32.0 (CH_2_), 31.8 (CH_2_), 30.1 (CH_2_), 27.4 (CH_3_), 26.1 (CH_2_), 24.5 (CH_2_), 23.3 (CH_3_), 22.2 (CH_2_), 14.9 (CH_3_); HRMS (FAB) *m*/*z*: [M + H]^+^ calcd for C_45_H_50_N_7_O_10_, 848.3614; found, 848.3624.

##### **TKP-14**: (*E*)-6-(4-((3-(3-(2-(2-Acetamidoethyl)-5-ethoxy-4-methoxyphenyl)-3-oxoprop-1-en-1-yl)­phenoxy)­methyl)-1*H*-1,2,3-triazol-1-yl)-*N*-(2-(2,6-dioxopiperidin-3-yl)-1,3-dioxoisoindolin-4-yl)­hexanamide

Following general procedure C, **TKP-14** was prepared as a yellow solid using alkyne **4** and azide **29**. *R*_f_ = 0.57 (8:1 CHCl_3_/MeOH); IR (neat) 3357, 2937, 2866, 1769, 1704, 1658, 1618, 1601, 1576, 1524, 1479, 1444, 1397, 1349, 1324, 1291, 1262, 1198, 1159, 1130, 1037, 986, 822, 748, 681, 666 cm^–1^; ^1^H NMR (399 MHz, CDCl_3_): δ 9.39 (s, 1H), 8.78 (dd, *J* = 8.5, 0.9 Hz, 1H), 8.54 (s, 1H), 7.69 (dd, *J* = 8.5, 7.3 Hz, 1H), 7.63 (s, 1H), 7.59–7.49 (m, 2H), 7.32 (t, *J* = 7.9 Hz, 1H), 7.24–7.13 (m, 3H), 7.08–7.03 (m, 3H), 6.82 (s, 1H), 5.22 (s, 2H), 4.95 (dd, *J* = 12.2, 5.4 Hz, 1H), 4.38 (t, *J* = 7.2 Hz, 2H), 4.10 (q, *J* = 7.9 Hz, 2H), 3.92 (s, 3H), 3.54–3.50 (m, 2H), 2.92 (m, 1H), 2.89 (t, *J* = 6.5 Hz, 2H), 2.87–2.68 (m, 2H), 2.45 (t, *J* = 7.3 Hz, 2H), 2.17 (m, 1H), 1.98 (quint, *J* = 7.2 Hz, 2H), 1.91 (s, 3H), 1.85–1.76 (m, 2H), 1.45 (t, *J* = 7.9 Hz, 3H), 1.47–1.36 (m, 2H); ^13^C NMR (100 MHz, CDCl_3_): δ 195.1 (C), 171.8 (C), 170.9 (C), 170.6 (C), 169.4 (C), 168.1 (C), 166.8 (C), 158.8 (C), 152.2 (C), 146.3 (C), 145.9 (CH), 143.9 (C), 137.8 (C), 136.6 (CH), 136.2 (C), 133.7 (C), 131.3 (C), 131.2 (C), 130.3 (CH), 126.7 (CH), 125.4 (CH), 122.8 (CH), 122.0 (CH), 118.7 (CH), 117.5 (CH), 115.5 (C), 114.6 (CH), 114.0 (CH), 113.9 (CH), 65.1 (CH_2_), 62.3 (CH_2_), 56.2 (CH_3_), 50.3 (CH_2_), 49.5 (CH), 42.0 (CH_2_), 37.5 (CH_2_), 32.1 (CH_2_), 31.5 (CH_2_), 30.1 (CH_2_), 26.0 (CH_2_), 24.5 (CH_2_), 23.3 (CH_3_), 22.8 (CH_2_), 14.9 (CH_3_); HRMS (FAB) *m*/*z*: [M + H]^+^ calcd for C_44_H_48_N_7_O_10_, 834.3457; found, 834.3446.

##### **TKP-16**: (*E*)-6-(4-((3-(3-(2-(2-Acetamidoethyl)-5-ethoxy-4-methoxyphenyl)-3-oxoprop-1-en-1-yl)­phenoxy)­methyl)-1*H*-1,2,3-triazol-1-yl)-*N*-(2-(2,6-dioxopiperidin-3-yl)-1,3-dioxoisoindolin-4-yl)­hexanamide

Following general procedure C, **TKP-16** was prepared as a yellow solid using alkyne **5** and azide **30**. *R*_f_ = 0.57 (8:1 CHCl_3_/MeOH); IR (neat) 3357, 2929, 2854, 1768, 1704, 1658, 1618, 1598, 1570, 1509, 1479, 1424, 1397, 1349, 1291, 1260, 1197, 1175, 1130, 1031, 989, 823, 748, 667 cm^–1^; ^1^H NMR (594 MHz, CDCl_3_): δ 9.40 (s, 1H), 8.80 (dd, *J* = 8.5, 0.8 Hz, 1H), 8.53 (s, 1H), 7.70 (dd, *J* = 8.5, 7.3 Hz, 1H), 7.61 (s, 1H), 7.56–7.50 (m, 4H), 7.24 (s, 1H), 7.06–6.99 (m, 4H), 6.81 (s, 1H), 5.24 (s, 2H), 4.96 (m, 1H), 4.39–4.30 (m, 2H), 4.09 (q, *J* = 7.0 Hz, 2H), 3.91 (s, 3H), 3.52–3.48 (m, 2H), 2.92 (m, 1H), 2.89–2.81 (m, 2H), 2.80 (m, 1H), 2.76 (m, 1H), 2.44 (t, *J* = 7.4 Hz, 2H), 2.17 (m, 1H), 1.94–1.89 (m, 2H), 1.91 (s, 3H), 1.75–1.70 (m, 2H), 1.45 (t, *J* = 7.0 Hz, 3H), 1.41–1.33 (m, 6H); ^13^C NMR (149 MHz, CDCl_3_): δ 195.6 (C), 172.3 (C), 170.9 (C), 170.7 (C), 169.3 (C), 168.1 (C), 166.8 (C), 160.7 (C), 151.9 (C), 146.2 (2 × CH), 143.6 (C), 137.9 (C), 136.6 (CH), 133.3 (C), 131.5 (C), 131.2 (C), 130.5 (CH), 127.8 (C), 125.4 (CH), 124.4 (CH), 122.8 (CH), 118.6 (CH), 115.50 (CH), 115.45 (C), 113.8 (CH), 113.5 (CH), 65.0 (CH_2_), 62.3 (CH_2_), 56.1 (CH_3_), 50.6 (CH_2_), 49.4 (CH), 42.1 (CH_2_), 37.9 (CH_2_), 31.8 (CH_2_), 31.5 (CH_2_), 30.3 (CH_2_), 28.9 (CH_2_), 28.7 (CH_2_), 26.4 (CH_2_), 25.1 (CH_2_), 23.3 (CH_3_), 22.8 (CH_2_), 14.9 (CH_3_); HRMS (FAB) *m*/*z*: [M + H]^+^ calcd for C_46_H_52_N_7_O_10_, 862.3770; found, 862.3768.

##### **TKP-17**: (*E*)-11-(4-((4-(3-(2-(2-Acetamidoethyl)-5-ethoxy-4-methoxyphenyl)-3-oxoprop-1-en-1-yl)­phenoxy)­methyl)-1*H*-1,2,3-triazol-1-yl)-*N*-(2-(2,6-dioxopiperidin-3-yl)-1,3-dioxoisoindolin-4-yl)­undecanamide

Following general procedure C, **TKP-17** was prepared as a yellow solid using alkyne **5** and azide **31**. *R*_f_ = 0.51 (8:1 CHCl_3_/MeOH); IR (neat) 3355, 2928, 2854, 1768, 1704, 1656, 1618, 1598, 1570, 1509, 1478, 1424, 1397, 1349, 1291, 1260, 1198, 1175, 1130, 1030, 988, 823, 748, 666 cm^–1^; ^1^H NMR (594 MHz, CDCl_3_): δ 9.40 (s, 1H), 8.81 (dd, *J* = 8.6, 0.7 Hz, 1H), 8.57 (s, 1H), 7.69 (dd, *J* = 8.6, 7.3 Hz, 1H), 7.60 (s, 1H), 7.55–7.49 (m, 4H), 7.25 (br, 1H), 7.07–6.99 (m, 4H), 6.81 (s, 1H), 5.24 (s, 2H), 4.95 (m, 1H), 4.34 (t, *J* = 7.2 Hz, 2H), 4.09 (q, *J* = 7.0 Hz, 2H), 3.91 (s, 3H), 3.54–3.48 (m, 2H), 2.92 (m, 1H), 2.88–2.82 (m, 2H), 2.84–2.71 (m, 2H), 2.43 (t, *J* = 7.6 Hz, 2H), 2.16 (m, 1H), 1.93–1.88 (m, 2H), 1.91 (s, 3H), 1.72 (quint, *J* = 7.6 Hz, 2H), 1.45 (t, *J* = 7.0 Hz, 3H), 1.38–1.23 (m, 12H); ^13^C NMR (149 MHz, CDCl_3_): δ 195.5 (C), 172.5 (C), 170.9 (C), 170.7 (C), 169.3 (C), 168.1 (C), 166.8 (C), 160.7 (C), 151.9 (C), 146.23 (C), 146.21 (CH), 143.5 (C), 138.0 (C), 136.5 (CH), 133.3 (C), 131.5 (C), 131.2 (C), 130.5 (CH), 127.7 (C), 125.3 (CH), 124.3 (CH), 122.7 (CH), 118.5 (CH), 115.5 (C), 115.4 (CH), 113.7 (CH), 113.5 (CH), 65.0 (CH_2_), 62.2 (CH_2_), 56.1 (CH_3_), 50.6 (CH_2_), 49.4 (CH), 42.1 (CH_2_), 38.0 (CH_2_), 31.7 (CH_2_), 31.5 (CH_2_), 30.3 (CH_2_), 29.3 (CH_2_), 29.3 (CH_2_), 29.2 (CH_2_), 29.1 (CH_2_), 28.9 (CH_2_), 26.5 (CH_2_), 25.2 (CH_2_), 23.3 (CH_3_), 22.8 (CH_2_), 14.9 (CH_3_); HRMS (FAB) *m*/*z*: [M + H]^+^ calcd for C_49_H_58_N_7_O_10_, 904.4240; found, 904.4244.

##### **TKP-18**: 6-(4-((4-(3-(2-(2-Acetamidoethyl)-5-ethoxy-4-methoxyphenyl)-3-oxopropyl)­phenoxy) methyl)-1*H*-1,2,3-triazol-1-yl)-*N*-(2-(2,6-dioxopiperidin-3-yl)-1,3-dioxoisoindolin-4-yl) Hexanamide

Following general procedure C, **TKP-18** was prepared as a white solid using alkyne **6** and azide **29**. *R*_f_ = 0.49 (8:1 CHCl_3_/MeOH); IR (neat) 3355, 2928, 2854, 1768, 1704, 1656, 1618, 1598, 1570, 1509, 1478, 1424, 1397, 1349, 1291, 1260, 1198, 1175, 1130, 1030, 988, 823, 748, 666 cm^–1^; ^1^H NMR (594 MHz, CDCl_3_): δ 9.39 (s, 1H), 8.78 (dd, *J* = 8.6, 0.8 Hz, 1H), 8.61 (s, 1H), 7.69 (dd, *J* = 8.6, 7.3 Hz, 1H), 7.61 (s, 1H), 7.53 (dd, *J* = 7.3, 0.8 Hz, 1H), 7.11 (d, *J* = 8.6 Hz, 2H), 7.06 (s, 1H), 6.90 (d, *J* = 8.6 Hz, 2H), 6.74 (s, 1H), 6.73 (m, 1H), 5.15 (s, 2H), 4.95 (dd, *J* = 12.4, 5.4 Hz, 1H), 4.37 (t, *J* = 7.3 Hz, 2H), 4.03 (q, *J* = 7.2 Hz, 2H), 3.88 (s, 3H), 3.45 (td, *J* = 6.7, 4.9 Hz, 2H), 3.16 (t, *J* = 7.5 Hz, 2H), 2.96 (t, *J* = 7.5 Hz, 2H), 2.85 (t, *J* = 6.7 Hz, 2H), 2.92–2.70 (m, 3H), 2.45 (t, *J* = 7.3 Hz, 2H), 2.16 (m, 1H), 1.97 (quint, *J* = 7.1 Hz, 2H), 1.90 (s, 3H), 1.78 (quint, *J* = 7.3 Hz, 2H), 1.44 (t, *J* = 7.2 Hz, 3H), 1.47–1.40 (m, 2H); ^13^C NMR (149 MHz, CDCl_3_): δ 203.2 (C), 171.8 (C), 171.0 (C), 170.6 (C), 169.3 (C), 168.1 (C), 166.8 (C), 156.9 (C), 152.5 (C), 146.3 (C), 144.4 (C), 137.8 (C), 136.6 (CH), 134.2 (C), 133.7 (C), 131.2 (C), 130.2 (C), 129.6 (CH), 125.4 (CH), 122.7 (CH), 118.7 (CH), 115.5 (C), 115.0 (CH), 114.3 (CH), 113.9 (CH), 65.1 (CH_2_), 62.3 (CH_2_), 56.1 (CH_3_), 50.2 (CH_2_), 49.4 (CH), 43.2 (CH_2_), 41.9 (CH_2_), 37.5 (CH_2_), 32.6 (CH_2_), 31.5 (CH_2_), 30.1 (CH_2_), 30.0 (CH_2_), 26.0 (CH_2_), 24.5 (CH_2_), 23.3 (CH_3_), 22.8 (CH_2_), 14.9 (CH_3_); HRMS (FAB) *m*/*z*: [M + H]^+^ calcd for C_44_H_50_N_7_O_10_, 836.3614; found, 836.3607.

##### **TKP-20**: 6-(4-((4-((1R*,2R*)-2-(2-(2-acetamidoethyl)-5-ethoxy-4-methoxyphenyl)-3-oxopropyl) phenoxy)­methyl)-1*H*-1,2,3-triazol-1-yl)-*N*-(2-(2,6-dioxopiperidin-3-yl)-1,3-dioxoisoindolin-4-yl) Hexanamide

Following general procedure C, **TKP-20** was prepared as a white solid using alkyne **7** and azide **29**. *R*_f_ = 0.40 (8:1 CHCl_3_/MeOH); IR (neat) 3358, 2931, 2856, 1768, 1704, 1659, 1617, 1514, 1479, 1397, 1349, 1262, 1199, 1131, 1028, 822, 748 cm^–1^; ^1^H NMR (399 MHz, CDCl_3_): δ 9.40 (s, 1H), 8.79 (d, *J* = 8.5 Hz, 1H), 8.30 (m, 1H), 7.70 (dd, *J* = 8.5, 7.3 Hz, 1H), 7.61 (s, 1H), 7.54 (d, *J* = 7.3 Hz, 1H), 7.20 (s, 1H), 7.10 (d, *J* = 8.7 Hz, 2H), 6.94 (d, *J* = 8.7 Hz, 2H), 6.87 (s, 1H), 6.75 (s, 1H), 5.18 (s, 2H), 4.95 (m, 1H), 4.37 (t, *J* = 7.3 Hz, 2H), 4.05–3.98 (m, 2H), 3.89 (s, 3H), 3.49 (s, 2H), 2.90 (m, 1H), 2.88 (t, *J* = 7.1 Hz, 2H), 2.84–2.68 (m, 2H), 2.63 (m, 1H), 2.56 (m, 1H), 2.46 (t, *J* = 7.5 Hz, 2H), 2.17 (m, 1H), 1.98 (quint, *J* = 7.3 Hz, 2H), 1.90 (m, 1H), 1.89 (s, 3H), 1.80 (quint, *J* = 7.5 Hz, 2H), 1.52 (m, 1H), 1.47–1.43 (m, 2H), 1.40 (t, *J* = 7.2 Hz, 3H); ^13^C NMR (100 MHz, CDCl_3_): δ 202.2 (C), 171.8 (C), 170.7 (C), 170.5 (C), 169.3 (C), 167.9 (C), 166.7 (C), 157.3 (C), 152.3 (C), 146.4 (C), 144.2 (C), 137.9 (C), 136.6 (CH), 133.1 (C), 132.9­(C), 131.6 (C), 131.3 (C), 127.5 (CH), 125.4 (CH), 122.7 (CH), 118.7 (CH), 115.5 (C), 115.2 (CH), 113.8 (CH), 113.7 (CH), 65.0 (CH_2_), 62.4 (CH_2_), 56.2 (CH_3_), 50.2 (CH_2_), 49.5 (CH), 42.1 (CH_2_), 37.5 (CH_2_), 32.9 (CH), 32.2 (CH_2_), 31.5 (CH_2_), 30.3 (CH), 30.1 (CH_2_), 26.1 (CH_2_), 24.5 (CH_2_), 23.4 (CH_3_), 22.8 (CH_2_), 19.3 (CH_2_), 14.9 (CH_3_); HRMS (FAB) *m*/*z*: [M + H]^+^ calcd for C_45_H_50_N_7_O_10_, 848.3614; found, 848.3642.

#### Synthesis of 1,3-Butadiyne-Typed PROTACs

##### *N*-(2-(2,6-Dioxopiperidin-3-yl)-1,3-dioxoisoindolin-4-yl)­oct-7-ynamide (**34**)

Pomalidomide (4.00 g, 14.7 mmol) was added to a solution of acid chloride (3.25 g) in DMF (30 mL) at 0 °C. After 24 h of stirring at 40 °C, the reaction mixture was concentrated in vacuo. The resulting mixture was diluted with Et_2_O (20 mL). The solid was filtered and washed with Et_2_O to give amide **34** (5.26 g, 13.3 mmol, 91%) as a cream solid. IR (neat) 3358, 3280, 3113, 2935, 2861, 2114, 1769, 1702, 1617, 1526, 1479, 1426, 1398, 1349, 1324, 1293, 1260, 1197, 1118, 1024, 822, 747 cm^–1^; ^1^H NMR (399 MHz, CDCl_3_): δ 9.41 (s, 1H), 8.83 (d, *J* = 8.0 Hz, 1H), 8.08 (s, 1H), 7.72 (t, *J* = 8.0 Hz, 1H), 7.55 (d, *J* = 8.0 Hz, 1H), 4.96 (m, 1H), 2.95–2.72 (m, 3H), 2.48 (t, *J* = 7.7 Hz, 2H), 2.25–2.18 (m, 2H), 2.16 (m, 1H), 1.94 (m, 1H), 1.78 (quint, *J* = 7.7 Hz, 2H), 1.60–1.50 (m, 4H); ^13^C NMR (100 MHz, CDCl_3_): δ 172.3 (C), 171.1 (C), 169.3 (C), 168.2 (C), 166.8 (C), 138.0 (C), 136.6 (CH), 131.2 (C), 125.4 (CH), 118.6 (CH), 115.4 (C), 84.4 (C), 68.6 (CH), 49.4 (CH), 37.9 (CH_2_), 31.5 (CH_2_), 28.3 (CH_2_), 28.2 (CH_2_), 24.8 (CH_2_), 22.8 (CH_2_), 18.3 (CH_2_); HRMS (FAB) *m*/*z*: [M + H]^+^ calcd for C_21_H_22_N_3_O_5_, 396.1554; found, 396.1563.

##### 8-Bromo-*N*-(2-(2,6-dioxopiperidin-3-yl)-1,3-dioxoisoindolin-4-yl)­oct-7-ynamide (**35**)

NBS (1.62 g, 9.10 mmol) and AgNO_3_ (129 mg, 759 mmol) were added to a solution of alkyne **34** (3.00 g, 7.59 mmol) in acetone (30 mL). After 3 h of stirring at room temperature, the reaction mixture was filtrated through a Celite pad, and the filtrate was evaporated in vacuo. The mixture was added to water (20 mL) and extracted with AcOEt (3 × 20 mL). The combined organic extracts were washed with brine (50 mL) and dried over anhydrous Na_2_SO_4_. Filtration and evaporation in vacuo furnished the crude product, which was purified by flash column chromatography (CHCl_3_) to give bromo alkyne **35** (2.85 g, 6.01 mmol, 79%) as a cream solid. IR (neat) 3360, 3226, 3114, 2934, 2858, 2214, 1769, 1702, 1617, 1526, 1478, 1426, 1398, 1349, 1323, 1292, 1260, 1196, 1118, 822, 747 cm^–1^; ^1^H NMR (399 MHz, CDCl_3_): δ 9.41 (s, 1H), 8.83 (dd, *J* = 8.5, 0.8 Hz, 1H), 8.14 (s, 1H), 7.72 (dd, *J* = 8.5, 7.3 Hz, 1H), 7.55 (dd, *J* = 7.3, 0.8 Hz, 1H), 4.96 (dd, *J* = 12.2, 5.4 Hz, 1H), 2.91 (m, 1H), 2.86–2.69 (m, 2H), 2.48 (t, *J* = 7.5 Hz, 2H), 2.24 (t, *J* = 6.9 Hz, 2H), 2.18 (m, 1H), 1.77 (quint, *J* = 9.6, 7.5 Hz, 2H), 1.58–1.47 (m, 4H); ^13^C NMR (100 MHz, CDCl_3_): δ 172.3 (C), 170.7 (C), 169.3 (C), 167.9 (C), 166.8 (C), 138.0 (C), 136.6 (CH), 131.2 (C), 125.5 (CH), 118.6 (CH), 115.4 (C), 80.1 (C), 49.4 (CH), 38.1 (CH_2_), 37.9 (CH_2_), 31.5 (CH_2_), 28.3 (CH_2_), 28.0 (CH_2_), 24.8 (CH_2_), 22.8 (CH_2_), 19.7 (CH_2_); HRMS (EI) *m*/*z*: [M]^+^ calcd for C_21_H_20_BrN_3_O_5_, 473.0586; found, 473.0568.

##### *N*-(2-(2,6-Dioxopiperidin-3-yl)-1,3-dioxoisoindolin-4-yl)­dec-9-ynamide (**36**)

Pomalidomide (2.00 g, 7.35 mmol) was added to a solution of acid chloride (2.24 g) in DMF (15 mL) at 0 °C. After 24 h of stirring at 40 °C, the reaction mixture was concentrated in vacuo. The resulting mixture was diluted with Et_2_O (10 mL). The solid was filtered and washed with Et_2_O to give amide **42** (2.05 g, 4.85 mmol, 66%) as a cream solid. IR (neat) 3357, 3284, 3113, 2931, 2856, 2117, 1770, 1702, 1618, 1529, 1479, 1404, 1358, 1294, 1259, 1198, 1119, 1026, 823, 746, 658 cm^–1^; ^1^H NMR (600 MHz, DMSO-*d*_6_): δ 11.15 (s, 1H), 9.68 (s, 1H), 8.47 (d, *J* = 8.4 Hz, 1H), 7.82 (dd, *J* = 8.4, 7.2 Hz, 1H), 7.60 (d, *J* = 7.2 Hz, 1H), 5.15 (dd, *J* = 12.9, 5.4 Hz, 1H), 2.90 (m, 1H), 2.71 (t, *J* = 2.6 Hz, 1H), 2.66–2.51 (m, 2H), 2.46 (t, *J* = 7.6 Hz, 2H), 2.14 (td, *J* = 7.0, 2.6 Hz, 2H), 2.07 (m, 1H), 1.62 (quint, *J* = 7.6 Hz, 2H), 1.44 (quint, *J* = 7.0 Hz, 2H), 1.39–1.25 (m, 6H).; ^13^C NMR (151 MHz, DMSO-*d*_6_): δ 172.8 (C), 172.0 (C), 169.8 (C), 167.7 (C), 166.7 (C), 136.6 (C), 136.1 (CH), 131.5 (C), 126.3 (CH), 118.3 (CH), 116.9 (C), 84.5 (C), 71.0 (CH), 48.8 (CH), 36.5 (CH_2_), 30.9 (CH_2_), 28.4 (CH_2_), 28.2 (CH_2_), 28.0 (CH_2_), 27.9 (CH_2_), 24.7 (CH_2_), 22.0 (CH_2_), 17.6 (CH_2_); HRMS (FAB) *m*/*z*: [M + H]^+^ calcd for C_23_H_26_N_3_O_5_^+^, 424.1867; found, 424.1875.

##### 10-Bromo-*N*-(2-(2,6-dioxopiperidin-3-yl)-1,3-dioxoisoindolin-4-yl)­dec-9-ynamide (**37**)

NBS (403 mg, 2.27 mmol) and AgNO_3_ (32.1 mg, 189 μmol) were added to a solution of alkyne **42** (800 mg, 1.89 mmol) in acetone (7.6 mL). After 3 h of stirring at room temperature, the reaction mixture was filtrated through a Celite pad, and the filtrate was evaporated in vacuo. The mixture was added to water (10 mL) and extracted with AcOEt (3 × 10 mL). The combined organic extracts were washed with brine (25 mL) and dried over anhydrous Na_2_SO_4_. Filtration and evaporation in vacuo furnished the crude product, which was purified by flash column chromatography (CHCl_3_) to give bromo alkyne **44** (776 mg, 1.54 mmol, 82%) as a cream solid. IR (neat) 3354, 3234, 3124, 2933, 2858, 2160, 1768, 1702, 1618, 1479, 140, 1352, 1259, 1196, 1119, 823, 746 cm^–1^; ^1^H NMR (600 MHz, DMSO-*d*_6_): δ 11.15 (s, 1H), 9.67 (s, 1H), 8.48 (d, *J* = 8.4 Hz, 1H), 7.81 (dd, *J* = 8.4, 7.2 Hz, 1H), 7.60 (d, *J* = 7.2 Hz, 1H), 5.15 (dd, *J* = 12.9, 5.4 Hz, 1H), 2.90 (m, 1H), 2.67–2.51 (m, 2H), 2.46 (t, *J* = 7.7 Hz, 2H), 2.21 (t, *J* = 7.3 Hz, 2H), 2.06 (m, 1H), 1.62 (quint, *J* = 7.97 Hz, 2H), 1.44 (quint, *J* = 7.3 Hz, 2H), 1.38–1.24 (m, 6H).; ^13^C NMR (151 MHz, DMSO-*d*_6_): δ 173.3 (C), 172.5 (C), 170.3 (C), 168.3 (C), 167.2 (C), 137.1 (C), 136.6 (CH), 132.0 (C), 126.7 (CH), 118.8 (CH), 117.4 (C), 81.0 (C), 49.4 (CH), 45.9 (C), 37.0 (CH_2_), 31.5 (CH_2_), 28.9 (CH_2_), 28.7 (CH_2_), 28.6 (CH_2_), 28.2 (CH_2_), 25.2 (CH_2_), 22.5 (CH_2_), 19.4 (CH_2_); HRMS (FAB) *m*/*z*: [M + H]^+^ calcd for C_23_H_25_BrN_3_O_5_^+^, 502.0972; found, 502.0983.

##### **TKP-21**: 11-(4-(3-(2-(2-Acetamidoethyl)-5-ethoxy-4-methoxyphenyl)-3-oxopropyl)­phenoxy)-*N*-(2-(2,6-dioxopiperidin-3-yl)-1,3-dioxoisoindolin-4-yl)­undeca-7,9-diynamide

A mixture of Pd_2_(dba)_3_ (81.8 mg, 79.1 μmol), phosphine ligand (62.1 mg, 158 μmol), and CuI (15.1 mg, 79.1 μmol) in DMF (8.0 mL) was degassed. After 10 min of stirring, alkyne **5** (733 mg, 1.74 mmol) and Et_3_N (440 μL, 3.17 mmol) were added. After another 5 min of stirring, bromoalkyne **35** (750 mg, 1.58 mol) was added. After 10 h of stirring at room temperature, the reaction was quenched with saturated aq. NH_4_Cl (20 mL), and the mixture was extracted with CHCl_3_ (2 × 20 mL). The combined organic extracts were washed with brine (3 × 20 mL) and dried over anhydrous Na_2_SO_4_. Filtration and evaporation in vacuo furnished the crude product, which was purified by flash column chromatography (100:0 → 50:1, CHCl_3_/MeOH) to give **TKP-21** (1.10 g, 1.35 mmol, 85%) as a yellow solid. *R*_f_ = 0.53 (8:1 CHCl_3_/MeOH); IR (neat) 3357, 3101, 2928, 2853, 2255, 1771, 1703, 1655, 1595, 1509, 1476, 1397, 1351, 1261, 1177, 1130, 1009, 821, 747 cm^–1^; ^1^H NMR (399 MHz, CDCl_3_): δ 9.40 (s, 1H), 8.81 (d, *J* = 8.5 Hz, 1H), 8.50 (s, 1H), 7.70 (dd, *J* = 8.5, 7.3 Hz, 1H), 7.57–7.49 (m, 4H), 7.21 (br, 1H), 7.09–7.01 (m, 2H), 7.01–6.94 (m, 2H), 6.81 (s, 1H), 4.95 (m, 1H), 4.77 (s, 2H), 4.09 (q, *J* = 7.0 Hz, 2H), 3.92 (s, 3H), 3.52 (m, 2H), 2.95–2.68 (m, 5H), 2.46 (t, *J* = 7.5 Hz, 2H), 2.30 (t, *J* = 6.9 Hz, 2H), 2.16 (m, 1H), 1.91 (s, 3H), 1.81–1.69 (m, 2H), 1.64–1.52 (m, 2H), 1.52–1.41 (m, 2H), 1.46 (t, *J* = 7.0 Hz, 3H); ^13^C NMR (100 MHz, CDCl_3_): δ 195.6 (C), 172.1 (C), 170.9 (C), 170.7 (C), 169.3 (C), 168.0 (C), 166.8 (C), 159.9 (C), 151.9 (C), 146.2 (CH), 146.1 (C), 137.9 (C), 136.6 (CH), 133.3 (C), 131.4 (C), 131.2 (C), 130.4 (CH), 128.1 (C), 125.4 (CH), 124.5 (CH), 118.6 (CH), 115.5 (C), 115.4 (CH), 113.7 (CH), 113.5 (CH), 82.1 (C), 72.8 (C), 69.7 (C), 65.0 (CH_2_), 64.6 (C), 56.5 (CH_2_), 56.1 (CH_3_), 49.4 (CH), 42.1 (CH_2_), 37.8 (CH_2_), 31.8 (CH_2_), 31.5 (CH_2_), 28.3 (CH_2_), 27.8 (CH_2_), 24.7 (CH_2_), 23.3 (CH_3_), 22.8 (CH_2_), 19.2 (CH_2_), 14.9 (CH_3_); HRMS (FAB) *m*/*z*: [M + H]^+^ calcd for C_46_H_49_N_4_O_10_, 815.3287; found, 815.3286.

##### (*E*)-*N*-(4-Ethoxy-2-(3-(4-ethynylphenyl)­acryloyl)-5-methoxyphenethyl)­acetamide (**39**)

Following general procedure A, compound **39** was prepared in 93% yield as a yellow solid using aldehyde **38** (1.0 equiv) and methyl ketone **14** (1.0 equiv). IR (neat) 3288, 2933, 2106, 1657, 1599, 1556, 1444, 1350, 1263, 1200, 1130, 1038, 984, 827, 771 cm^–1^; ^1^H NMR (600 MHz, CDCl_3_): δ 7.62–7.47 (m, 5H), 7.17 (d, *J* = 15.9 Hz, 1H), 7.08 (br, 1H), 7.06 (s, 1H), 6.82 (s, 1H), 4.10 (q, *J* = 6.6 Hz, 2H), 3.93 (s, 3H), 3.53 (q, *J* = 5.7 Hz, 2H), 3.22 (s, 1H), 2.88 (t, *J* = 5.7 Hz, 2H), 1.92 (s, 3H), 1.46 (t, *J* = 6.6 Hz, 3H); ^13^C NMR (151 MHz, CDCl_3_): δ 194.9 (C), 170.6 (C), 152.3 (C), 146.3 (C), 145.0 (CH), 134.9 (C), 133.8 (C), 132.9 (CH), 131.1 (C), 128.5 (CH), 127.0 (CH), 124.7 (C), 113.9 (CH), 113.6 (CH), 83.2 (CH), 79.8 (C), 65.1 (CH_2_), 56.2 (CH_3_), 42.1 (CH_2_), 31.9 (CH_2_), 23.4 (CH_3_), 14.9 (CH_3_); HRMS (EI) *m*/*z*: [M]^+^ calcd for C_24_H_25_NO_4_, 391.1784; found, 391.1786.

##### **TKP30**: (*E*)-12-(4-(3-(2-(2-Acetamidoethyl)-5-ethoxy-4-methoxyphenyl)-3-oxoprop-1-en-1-yl) phenyl)-*N*-(2-(2,6-dioxopiperidin-3-yl)-1,3-dioxoisoindolin-4-yl)­dodeca-9,11-diynamide

After freeze–pump–thaw cycling for the mixture of alkyne **39** (281 mg, 717 μmol), bromoalkyne **37** (300 mg, 597 μmol), and Et_3_N (166 mL, 1.19 mmol) in DMF (6.0 mL), it was added to a mixture of Pd_2_(dba)_3_ (30.9 mg, 29.9 μmol), phosphine ligand (23.4 mg, 59.7 μmol), and CuI (11.4 mg, 59.7 μmol). After 8 h of stirring at room temperature, the reaction was quenched with saturated aq. NH_4_Cl (20 mL), and the mixture was extracted with CHCl_3_ (2 × 20 mL). The combined organic extracts were washed with brine (3 × 20 mL) and dried over anhydrous Na_2_SO_4_. Filtration and evaporation in vacuo furnished the crude product, which was purified by flash column chromatography (100:0 → 50:1, CHCl_3_/MeOH) to give **TKP-30** (390 mg, 480 μmol, 80%) as a yellow solid. ^1^H NMR (600 MHz, CDCl_3_): δ 9.40 (br, 1H), 8.81 (d, *J* = 8.2 Hz, 1H), 8.69 (br, 1H), 7.69 (t, *J* = 8.2 Hz, 1H), 7.58–7.42 (m, 6H), 7.14 (d, *J* = 15.9 Hz, 1H), 7.09 (t, *J* = 5.9 Hz, 1H), 7.04 (s, 1H), 6.81 (s, 1H), 4.94 (m, 1H), 4.08 (q, *J* = 7.0 Hz, 2H), 3.91 (s, 3H), 3.51 (q, *J* = 5.9 Hz, 2H), 2.99–2.67 (m, 5H), 2.45 (t, *J* = 7.4 Hz, 2H), 2.36 (t, *J* = 7.0 Hz, 2H), 2.15 (m, 1H), 1.90 (s, 3H), 1.74 (quint, *J* = 7.4 Hz, 2H), 1.57 (quint, *J* = 7.0 Hz, 2H), 1.45 (t, *J* = 7.0 Hz, 3H), 1.43–1.31 (m, 6H); ^13^C NMR (151 MHz, CDCl_3_): δ 194.8 (C), 172.4 (C), 171.0 (C), 170.6 (C), 169.3 (C), 168.1 (C), 166.8 (C), 152.2 (C), 146.2 (C), 144.9 (CH), 138.0 (C), 136.5 (CH), 134.8 (C), 133.7 (C), 133.1 (CH), 131.2 (C), 131.0 (C), 128.5 (CH), 126.9 (CH), 125.4 (CH), 124.7 (C), 118.5 (CH), 115.3 (C), 113.9 (CH), 113.6 (CH), 86.5 (C), 77.1 (C), 74.3 (C), 65.2 (C), 65.0 (CH_2_), 56.1 (CH_3_), 49.4 (CH), 42.0 (CH_2_), 38.0 (CH_2_), 31.9 (CH_2_), 31.5 (CH_2_), 29.0 (CH_2_), 28.8 (CH_2_), 28.7 (CH_2_), 28.1 (CH_2_), 25.2 (CH_2_), 23.3 (CH_3_), 22.8 (CH_2_), 19.7 (CH_2_), 14.9 (CH_3_); HRMS (FAB) *m*/*z*: [M + H]^+^ calcd for C_47_H_49_N_4_O_9_, 813.3494; found, 813.3494.

#### Synthesis of 1,3-Butadiyne-Derived PROTACs

##### **TKP-22**: (*E*)-6-(5-((4-(3-(2-(2-Acetamidoethyl)-5-ethoxy-4-methoxyphenyl)-3-oxoprop-1-en-1-yl)­phenoxy)­methyl)­thiophen-2-yl)-*N*-(2-(2,6-dioxopiperidin-3-yl)-1,3-dioxoisoindolin-4-yl) Hexanmide

Na_2_S·9H_2_O (88.4 mg, 368 μmol) was added to a solution of **TKP-21** (50.0 mg, 61.4 μmol) in DMSO (12 mL). After 6 h of stirring at rt, the reaction mixture was diluted with CHCl_3_ (20 mL) and the reaction was quenched with half-saturated aqueous NH_4_Cl (20 mL), and the mixture was extracted with CHCl_3_ (3 × 30 mL). The combined organic extracts were washed with brine (3 × 50 mL) and dried over anhydrous Na_2_SO_4_. Filtration and evaporation in vacuo furnished the crude product, which was purified by flash column chromatography (100:0 → 50:1 CHCl_3_/MeOH) to give **TKP-22** (40.2 mg, 47.3 μmol, 77%) as a yellow solid. *R*_f_ = 0.53 (8:1 CHCl_3_/MeOH); IR (neat) 3351, 2930, 2856, 1768, 1705, 1657, 1619, 1599, 1570, 1509, 1479, 1424, 1397, 1350, 1261, 1199, 1174, 1130, 1029, 988, 822, 748, 666 cm^–1^; ^1^H NMR (399 MHz, CDCl_3_): δ 9.41 (s, 1H), 8.82 (dd, *J* = 8.2, 3.7 Hz, 1H), 8.10 (s, 1H), 7.76–7.67 (t, *J* = 8.2 Hz, 1H), 7.58–7.50 (m, 4H), 7.24 (br, 1H), 7.09–6.96 (m, 4H), 6.92 (d, *J* = 3.4 Hz, 1H), 6.81 (s, 1H), 6.67 (d, *J* = 3.4 Hz, 1H), 5.18 (s, 2H), 4.95 (dd, *J* = 12.2, 5.4 Hz, 1H), 4.10 (q, *J* = 7.0 Hz, 2H), 3.93 (s, 3H), 3.57–3.48 (m, 2H), 3.00–2.69 (m, 5H), 2.47 (t, *J* = 7.5 Hz, 2H), 2.18 (m, 1H), 1.92 (s, 3H), 1.83–1.70 (m, 4H), 1.54–1.38 (m, 5H); ^13^C NMR (100 MHz, CDCl_3_): δ 195.6 (C), 172.3 (C), 170.7 (C), 170.6 (C), 169.3 (C), 167.9 (C), 166.8 (C), 160.9 (C), 151.9 (C), 147.2 (C), 146.4 (CH), 146.2 (C), 138.0 (C), 136.6 (CH), 135.9 (C), 133.3 (C), 131.6 (C), 131.2 (C), 130.5 (CH), 127.7 (C), 127.4 (CH), 125.4 (CH), 124.3 (CH), 124.1 (CH), 118.6 (CH), 115.6 (CH), 115.4 (C), 113.7 (CH), 113.5 (CH), 65.5 (CH_2_), 65.0 (CH_2_), 56.2 (CH_3_), 49.4 (CH), 42.2 (CH_2_), 41.2 (CH_2_), 37.9 (CH_2_), 31.5 (CH_2_), 31.4 (CH_2_), 30.1 (CH_2_), 28.7 (CH_2_), 25.0 (CH_2_), 23.4 (CH_3_), 22.8 (CH_2_), 14.9 (CH_3_); HRMS (FAB) *m*/*z*: [M + H]^+^ calcd for C_46_H_51_N_4_O_10_S, 849.3164; found, 849.3173. HPLC purity ≥ 99%.

##### **TKP-44**: (*E*)-8-(5-(4-(3-(2-(2-Acetamidoethyl)-5-ethoxy-4-methoxyphenyl)-3-oxoprop-1-en-1-yl)­phenyl)­thiophen-2-yl)-*N*-(2-(2,6-dioxopiperidin-3-yl)-1,3-dioxoisoindolin-4-yl)­octanamide

Na_2_S·9H_2_O (22.9 mg, 95.2 μmol) was added to a solution of **TKP-30** (25.8 mg, 31.7 μmol) in DMSO (600 μL). After 1 h of stirring at rt, the reaction mixture was diluted with CHCl_3_ (10 mL) and the reaction was quenched with half saturated aqueous NH_4_Cl (10 mL), and the mixture was extracted with CHCl_3_ (2 × 20 mL). The combined organic extracts were washed with brine (3 × 30 mL) and dried over anhydrous Na_2_SO_4_. Filtration and evaporation in vacuo furnished the crude product, which was purified by flash column chromatography (100:0 → 50:1 CHCl_3_/MeOH) to give **TKP-44** (2.8 mg, 3.3 μmol, 10%) as a yellow solid. ^1^H NMR (597 MHz, CDCl_3_): δ 9.41 (s, 1H), 8.84 (d, *J* = 8.4 Hz, 1H), 8.02 (s, 1H), 7.72 (t, *J* = 8.4 Hz, 1H), 7.61–7.50 (m, 6H), 7.21 (d, *J* = 3.6 Hz, 1H), 7.19 (br, 1H), 7.15 (d, *J* = 15.9 Hz, 1H), 7.07 (s, 1H), 6.82 (s, 1H), 6.77 (d, *J* = 3.6 Hz, 1H), 4.95 (m, 1H), 4.11 (q, *J* = 6.9 Hz, 2H), 3.93 (s, 3H), 3.54 (q, *J* = 5.4 Hz, 2H), 2.99–2.70 (m, 7H), 2.46 (t, *J* = 7.5 Hz, 2H), 2.17 (m, 1H), 1.93 (s, 3H), 1.80–1.68 (m, 4H), 1.47 (t, *J* = 6.9 Hz, 3H), 1.44–1.40 (m, 6H); ^13^C NMR (150 MHz, CDCl_3_): δ 195.4 (C), 172.5 (C), 170.7 (C), 170.6 (C), 169.3 (C), 167.8 (C), 166.8 (C), 152.1 (C), 147.2 (C), 146.3 (C), 146.0 (CH), 140.7 (C), 138.1 (C), 137.4 (C), 136.6 (CH), 133.5 (C), 133.0 (C), 131.4 (C), 131.2 (C), 129.3 (CH), 125.8 (CH), 125.65 (CH), 125.63 (CH), 125.4 (CH), 124.0 (CH), 118.6 (CH), 115.4 (C), 113.8 (CH), 113.5 (CH), 65.0 (CH_2_), 56.2 (CH_3_), 49.4 (CH), 42.1 (CH_2_), 38.1 (CH_2_), 31.8 (CH_2_), 31.6 (CH_2_), 31.5 (CH_2_), 30.4 (CH_2_), 29.8 (CH_2_), 29.1 (CH_2_), 28.9 (CH_2_), 25.3 (CH_2_), 23.4 (CH_3_), 22.8 (CH_2_), 14.9 (CH_3_); HRMS (FAB) *m*/*z*: [M + H]^+^ calcd for C_47_H_51_N_4_O_9_S, 847.3371; found, 847.3376.

##### **TKP-45**: (*E*)-8-(5-(4-(3-(2-(2-Acetamidoethyl)-5-ethoxy-4-methoxyphenyl)-3-oxoprop-1-en-1-yl)­phenyl)­furan-2-yl)-*N*-(2-(2,6-dioxopiperidin-3-yl)-1,3-dioxoisoindolin-4-yl)­octanamide

SPhosAuNTf_2_ (7.37 mg, 8.30 μmol) and H_2_O (5.0 μL, 280 μmol) were added to a solution of **TKP-30** (22.5 mg, 27.7 μmol) in THF (550 μL), and the mixture was stirred at 60 °C for 24 h. The reaction mixture was filtered through a Celite pad, and the filtrate was dried over anhydrous Na_2_SO_4_. Filtration and evaporation in vacuo furnished the crude product, which was purified by flash column chromatography (100:0 → 50:1 CHCl3/MeOH) to give **TKP-45** (10.4 mg, 27.7 μmol, 45%) as a yellow solid. ^1^H NMR (597 MHz, CDCl_3_): δ 9.40 (s, 1H), 8.83 (d, *J* = 8.4 Hz, 1H), 8.22 (s, 1H), 7.71 (t, *J* = 8.4 Hz, 1H), 7.65 (d, *J* = 8.4 Hz, 2H), 7.62–7.47 (m, 4H), 7.22 (t, *J* = 5.4 Hz, 1H), 7.14 (d, *J* = 15.9 Hz, 1H), 7.07 (s, 1H), 6.82 (s, 1H), 6.66 (d, *J* = 3.3 Hz, 1H), 6.10 (d, *J* = 3.3 Hz, 1H), 4.95 (m, 1H), 4.11 (q, *J* = 7.0 Hz, 2H), 3.93 (s, 3H), 3.53 (q, *J* = 5.4 Hz, 2H), 2.97–2.74 (m, 5H), 2.69 (t, *J* = 7.5 Hz, 2H), 2.45 (t, *J* = 7.5 Hz, 2H), 2.16 (m, 1H), 1.93 (s, 3H), 1.75 (quint, *J* = 7.5 Hz, 2H), 1.70 (quint, *J* = 7.5 Hz, 2H), 1.47 (t, *J* = 7.0 Hz, 3H), 1.48–1.45 (m, 4H), 1.25–1.20 (m, 2H); ^13^C NMR (150 MHz, CDCl_3_): δ 195.5 (C), 172.5 (C), 170.73 (C), 170.71 (C), 169.3 (C), 167.9 (C), 166.8 (C), 157.7 (C), 152.0 (C), 151.4 (C), 146.24 (C), 146.21 (CH), 138.0 (C), 136.6 (CH), 133.5 (C), 133.4 (C), 132.7 (C), 131.4 (C), 131.2 (C), 129.2 (CH), 125.5 (CH), 125.4 (CH), 123.8 (CH), 118.6 (CH), 115.4 (C), 113.8 (CH), 113.6 (CH), 107.9 (CH), 107.7 (CH), 65.1 (CH_2_), 56.2 (CH_3_), 49.4 (CH), 42.1 (CH_2_), 38.1 (CH_2_), 31.8 (CH_2_), 31.5 (CH_2_), 29.8 (CH_2_), 29.11 (CH_2_), 29.06 (CH_2_), 28.3 (CH_2_), 28.1 (CH_2_), 25.3 (CH_2_), 23.4 (CH_3_), 22.8 (CH_2_), 14.9 (CH_3_); HRMS (FAB) *m*/*z*: [M + H]^+^ calcd for C_47_H_51_N_4_O_10_, 831.3600; found, 831.3600.

##### **TKP-46**: (*E*)-8-(5-(4-(3-(2-(2-Acetamidoethyl)-5-ethoxy-4-methoxyphenyl)-3-oxoprop-1-en-1-yl)­phenyl)-1-phenyl-1*H*-pyrrol-2-yl)-*N*-(2-(2,6-dioxopiperidin-3-yl)-1,3-dioxoisoindolin-4-yl) Octanamide

CuCl (7.31 mg, 73.8 μmol) and aniline (68.0 μL, 69.5 μmol) were added to a solution of **TKP-30** (30.0 mg, 36.9 μmol) in mesitylene (740 μL). After 24 h of stirring at rt, the reaction mixture was diluted with CHCl_3_ (5 mL) and the reaction was quenched with half saturated aqueous NH_4_Cl (5 mL), and the mixture was extracted with CHCl_3_ (2 × 10 mL). The combined organic extracts were washed with brine (20 mL) and dried over anhydrous Na_2_SO_4_. Filtration and evaporation in vacuo furnished the crude product, which was purified by flash column chromatography (100:0 → 50:1 CHCl_3_/MeOH) to give **TKP-46** (9.5 mg, 10 μmol, 28%) as a yellow solid. ^1^H NMR (597 MHz, CDCl_3_): δ 9.39 (s, 1H), 8.83 (d, *J* = 7.8 Hz, 1H), 8.02 (s, 1H), 7.72 (t, *J* = 7.8 Hz, 1H), 7.55 (d, *J* = 7.8 Hz, 1H), 7.46 (d, *J* = 15.8 Hz, 1H), 7.43–7.37 (m, 3H), 7.33 (d, *J* = 8.5 Hz, 2H), 7.21–7.15 (m, 3H), 7.06 (d, *J* = 8.5 Hz, 2H), 7.03 (d, *J* = 15.8 Hz, 1H), 7.00 (s, 1H), 6.80 (s, 1H), 6.49 (d, *J* = 3.6 Hz, 1H), 6.13 (d, *J* = 3.6 Hz, 1H), 4.95 (m, 1H), 4.08 (q, *J* = 7.0 Hz, 2H), 3.92 (s, 3H), 2.95–2.73 (m, 5H), 2.51–2.33 (m, 4H), 2.16 (m, 1H), 1.91 (s, 3H), 1.70 (quint, *J* = 7.5 Hz, 2H), 1.53–1.47 (m, 2H), 1.45 (t, *J* = 7.0 Hz, 3H), 1.38–1.21 (m, 6H); ^13^C NMR (150 MHz, CDCl_3_): δ 195.5 (C), 172.5 (C), 170.7 (C), 170.6 (C), 169.3 (C), 167.8 (C), 166.8 (C), 151.9 (C), 146.4 (CH), 146.2 (C), 139.3 (C), 138.4 (C), 138.1 (C), 136.6 (CH), 136.2 (C), 133.3 (C), 133.2 (C), 131.5 (C), 131.4 (C), 131.2 (C), 129.4 (CH), 128.7 (CH), 128.6 (CH), 128.0 (CH), 127.7 (CH), 125.4 (CH), 125.1 (CH), 118.6 (CH), 115.4 (C), 113.7 (CH), 113.4 (CH), 110.4 (CH), 107.2 (CH), 65.0 (CH_2_), 56.2 (CH_3_), 49.4 (CH), 42.1 (CH_2_), 38.1 (CH_2_), 31.7 (CH_2_), 31.5 (CH_2_), 29.9 (CH_2_), 29.2 (CH_2_), 29.1 (CH_2_), 28.9 (CH_2_), 27.2 (CH_2_), 25.3 (CH_2_), 23.4 (CH_3_), 22.8 (CH_2_), 14.9 (CH_3_); HRMS (FAB) *m*/*z*: [M + H]^+^ calcd for C_53_H_56_N_5_O_9_, 906.4073; found, 906.4074.

**TKP-34**: (*E*)-8-(4-((4-(3-(2-(2-Acetamidoethyl)-5-ethoxy-4-methoxyphenyl)-3-oxoprop-1-en-1-yl)­phenoxy)­methyl)-1-oxo-1,2-dihydroisoquinolin-3-yl)-*N*-(2-(2,6-dioxopiperidin-3-yl)-1,3-dioxoisoindolin-4-yl)­oct-7-ynamide.

##### **TKP-35**: (*E*)-6-(3-(3-(4-(3-(2-(2-Acetamidoethyl)-5-ethoxy-4-methoxyphenyl)-3-oxo prop-1-en-1-yl)­phenoxy)­prop-1-yn-1-yl)-1-oxo-1,2-dihydroisoquinolin-4-yl)-*N*-(2-(2,6-dioxopiperidin-3-yl)-1,3-dioxoisoindolin-4-yl)­hexanamide

*N*-Chloro amide (20.5 mg, 132 μmol), NaOAc (19.6 mg, 239 μmol), and Ru catalyst (7.32 mg, 12.0 μmol) were added to a solution of **TKP-21** (97.7 mg, 120 μmol) in TFE (1.2 mL). After 1 h of stirring at rt, the reaction mixture was filtered through a Celite pad and the filtrate was dried over anhydrous Na_2_SO_4_. Filtration and evaporation in vacuo furnished the crude product, which was purified by flash column chromatography (100:0 → 50:1 CHCl_3_/MeOH) to give **TKP-34** and **TKP-35** (1:1, 90.0 mg, 96.4 μmol, 80%) as a yellow solid. These isomers were separated using GPC.

**TKP-34**: *R*_f_ = 0.53 (8:1 CHCl_3_/MeOH); IR (neat) 3350, 3203, 2933, 2858, 2227, 1768, 1655, 1618, 1512, 1398, 1350, 1259, 1198, 1130, 989, 823, 750 cm^–1^; ^1^H NMR (600 MHz, CDCl_3_): δ 9.94 (br, 1H), 9.40 (br, 1H), 9.20 (br, 1H), 8.81 (d, *J* = 8.5 Hz, 1H), 8.40 (d, *J* = 8.0 Hz, 1H), 7.74–7.63 (m, 3H), 7.58–7.46 (m, 5H), 7.21 (br, 1H), 7.10–7.01 (m, 4H), 6.81 (s, 1H), 5.34 (s, 2H), 5.01 (m, 1H), 4.09 (q, *J* = 7.0 Hz, 2H), 3.91 (s, 3H), 3.51 (q, *J* = 6.0 Hz, 2H), 2.91–2.76 (m, 5H), 2.51 (t, *J* = 7.2 Hz, 2H), 2.42 (t, *J* = 7.2 Hz, 2H), 2.17 (m, 1H), 1.92 (s, 3H), 1.77 (quint, *J* = 7.2 Hz, 2H), 1.67 (quint, *J* = 7.2 Hz, 2H), 1.51–1.60 (quint, *J* = 7.2 Hz, 2H), 1.45 (t, *J* = 7.0 Hz, 3H); ^13^C NMR (151 MHz, CDCl_3_): δ 195.4 (C), 172.1 (C), 171.3 (C), 170.6 (C), 170.2 (C), 169.4 (C), 168.6 (C), 166.7 (C), 162.8 (C), 161.0 (C), 151.9 (C), 146.15 (CH), 146.05 (C), 137.7 (C), 136.7 (C), 136.6 (CH), 133.37 (C), 133.29 (CH), 131.4 (C), 131.2 (C), 130.5 (CH), 128.0 (CH), 127.8 (C), 127.6 (CH), 126.2 (C), 125.4 (CH), 124.3 (CH), 124.0 (CH), 118.6 (CH), 115.6 (CH), 115.4 (C), 114.5 (C), 113.7 (CH), 113.6 (CH), 99.5 (C), 73.6 (C), 65.3 (CH_2_), 65.0 (CH_2_), 56.1 (CH_3_), 49.4 (CH), 42.0 (CH_2_), 37.5 (CH_2_), 31.8 (CH_2_), 31.5 (CH_2_), 28.0 (CH_2_), 27.4 (CH_2_), 24.5 (CH_2_), 23.3 (CH_3_), 22.8 (CH_2_), 19.4 (CH_2_), 14.9 (CH_3_); HRMS (FAB) *m*/*z*: [M + H]^+^ calcd for C_53_H_52_N_5_O_11_, 934.3658; found, 934.3657.

**TKP-35**: *R*_f_ = 0.53 (8:1 CHCl_3_/MeOH); IR (neat); 3357, 3298, 3192, 2927, 2856, 2231, 1768, 1705, 1655, 1601, 1570, 1512, 1477, 1425, 1298, 1352, 1292, 1263, 1200, 1178, 1130, 1026, 823, 750, 665 cm^–1^; ^1^H NMR (597 MHz, DMSO-*d*_6_): δ 11.55 (s, 1H), 11.14 (s, 1H), 9.58 (s, 1H), 8.40 (d, *J* = 8.4 Hz, 1H), 8.20 (d, *J* = 7.5 Hz, 1H), 7.83 (t, *J* = 6.5 Hz, 1H), 7.80–7.69 (m, 5H), 7.54 (d, *J* = 7.5 Hz, 1H), 7.51 (t, *J* = 7.5 Hz, 1H), 7.47 (d, *J* = 15.8 Hz, 1H), 7.27 (d, *J* = 15.8 Hz, 1H), 7.15 (s, 1H), 7.11 (d, *J* = 8.6 Hz, 2H), 6.84 (s, 1H), 5.18 (s, 2H), 5.12 (m, 1H), 4.02 (q, *J* = 6.9 Hz, 2H), 3.80 (s, 3H), 3.22 (q, *J* = 6.5 Hz, 2H), 2.87 (m, 1H), 2.82–2.72 (m, 2H), 2.63–2.51 (m, 2H), 2.37 (t, *J* = 7.6 Hz, 2H), 2.05 (m, 1H), 1.57 (quint, *J* = 7.6 Hz, 2H), 1.46 (quint, *J* = 7.6 Hz, 2H), 1.33–1.27 (m, 5H); ^13^C NMR (150 MHz, DMSO-*d*_6_): δ 193.2 (C), 172.8 (C), 171.9 (C), 169.8 (C), 168.9 (C), 167.8 (C), 166.7 (C), 160.9 (C), 159.1 (C), 150.7 (C), 145.5 (C), 143.9 (CH), 136.5 (C), 136.0 (CH), 132.7 (CH), 132.6 (C), 131.3 (C), 130.9 (C), 130.5 (CH), 128.0 (C), 127.3 (CH), 127.2 (CH), 126.7 (C), 126.0 (CH), 124.3 (CH), 124.0 (CH), 121.2 (C), 119.4 (C), 118.2 (CH), 116.7 (C), 115.4 (CH), 114.2 (CH), 113.5 (CH), 91.1 (C), 80.1 (C), 64.0 (CH_2_), 56.0 (CH_2_), 55.5 (CH_3_), 48.9 (CH), 40.4 (CH_2_), 36.5 (CH_2_), 32.5 (CH_2_), 30.9 (CH_2_), 29.3 (CH_2_), 28.2 (CH_2_), 27.6 (CH_2_), 24.6 (CH_2_), 22.6 (CH_3_), 22.0 (CH_2_), 14.7 (CH_3_); HRMS (FAB) *m*/*z*: [M + H]^+^ calcd for C_53_H_52_N_5_O_11_, 934.3658; found, 934.3660.

##### 11-(4-(3-(2-(2-Acetamidoethyl)-5-ethoxy-4-methoxyphenyl)-3-oxopropyl)­phenoxy)-*N*-(2-(2,6-dioxopiperidin-3-yl)-1,3-dioxoisoindolin-4-yl)­undecanamide (**40**)

5% Pd/C (8.0 mg) was added to a solution of **TKP-21** (80.0 mg, 98.2 μmol) in MeOH/AcOEt (1:1, 2.0 mL), and the mixture was stirred under hydrogen for 12 h. The reaction mixture was filtered through a Celite pad, and the filtrate was dried over anhydrous Na_2_SO_4_. Filtration and evaporation in vacuo furnished the crude product, which was purified by flash column chromatography (100:0 → 50:1 CHCl_3_/MeOH) to give ketone **40** (52.0 mg, 63.2 μmol, 64%) as a yellow solid. *R*_f_ = 0.53 (8:1 CHCl_3_/MeOH); IR (neat) 2920, 2846, 1705, 1657, 1618, 1514, 1396, 1363, 1261, 1194, 1126, 1026, 916, 795, 744 cm^–1^ ; ^1^H NMR (600 MHz, CDCl_3_): δ 9.40 (s, 1H), 8.82 (dd, *J* = 8.5, 0.7 Hz, 1H), 8.59 (s, 1H), 7.69 (dd, *J* = 8.5, 7.4 Hz, 1H), 7.53 (dd, *J* = 7.4, 0.7 Hz, 1H), 7.10 (d, *J* = 8.7 Hz, 2H), 7.03 (s, 1H), 6.81 (d, *J* = 8.7 Hz, 2H), 6.77 (br, 1H), 6.74 (s, 1H), 4.95 (dd, *J* = 12.6, 5.4 Hz, 1H), 4.02 (q, *J* = 7.0 Hz, 2H), 3.90 (t, *J* = 6.6 Hz, 2H), 3.88 (s, 3H), 3.46 (q, *J* = 6.6 Hz, 2H), 3.15 (t, *J* = 7.5 Hz, 2H), 2.96 (t, *J* = 7.5 Hz, 2H), 2.93–2.66 (m, 5H), 2.44 (t, *J* = 7.6 Hz, 2H), 2.15 (m, 1H), 1.90 (s, 3H), 1.78–1.70 (m, 4H), 1.44 (t, *J* = 7.0 Hz, 3H), 1.45–1.29 (m, 12H); ^13^C NMR (151 MHz, CDCl_3_): δ 203.4 (C), 172.6 (C), 171.0 (C), 170.5 (C), 169.3 (C), 168.1 (C), 166.8 (C), 157.8 (C), 152.4 (C), 146.3 (C), 138.0 (C), 136.5 (CH), 134.1 (C), 132.8 (C), 131.2 (C), 130.3 (C), 129.4 (CH), 125.4 (CH), 118.5 (CH), 115.4 (C), 114.7 (CH), 114.3 (CH), 113.7 (CH), 68.1 (CH_2_), 65.0 (CH_2_), 56.1 (CH_3_), 49.4 (CH), 43.4 (CH_2_), 41.9 (CH_2_), 38.1 (CH_2_), 32.6 (CH_2_), 31.5 (CH_2_), 30.1 (CH_2_), 29.5 (CH_2_), 29.44 (CH_2_), 29.42 (CH_2_), 29.38 (CH_2_), 29.3 (CH_2_), 29.2 (CH_2_), 26.1 (CH_2_), 25.3 (CH_2_), 23.3 (CH_3_), 22.8 (CH_2_), 14.9 (CH_3_); HRMS (FAB) *m*/*z*: [M + H]^+^ calcd for C_46_H_57_N_4_O_10_, 825.4069; found, 825.4071.

##### **TKP-26**: (*E*)-11-(4-(3-(2-(2-Acetamidoethyl)-5-ethoxy-4-methoxyphenyl)-3-oxoprop-1-en-1-yl) phenoxy)-*N*-(2-(2,6-dioxopiperidin-3-yl)-1,3-dioxoisoindolin-4-yl)­undecanamide

Pd­(TFA)_2_ (14.2 mg, 42.7 μmol) and 4,5-diazafluoren-9-one (**41**) (7.77 mg, 42.7 μmol) were added to a solution of ketone **40** (32.0 mg, 38.8 μmol) in DMSO (1.0 mL), and the reaction mixture was purged with O_2_ for 3 times. After 24 h of stirring at 100 °C under O_2_, the reaction was quenched with saturated aqueous NaHCO_3_ (3 mL) at 20 °C, and the mixture was extracted with CHCl_3_ (3 × 10 mL). The combined organic extracts were washed with brine (30 mL) and dried over anhydrous Na_2_SO_4_. Filtration and evaporation in vacuo furnished the crude product, which was purified by flash column chromatography (50:1 → 20:1, CHCl_3_/MeOH) to give **TKP-26** (11.0 mg, 13.4 μmol, 35%) as a yellow solid. *R*_f_ = 0.53 (8:1 CHCl_3_/MeOH); IR (neat) 2929, 2850, 1768, 1705, 1597, 1512, 1390, 1344, 1259, 1194, 789, 748 cm^–1^; ^1^H NMR (600 MHz, CDCl_3_): δ 9.41 (s, 1H), 8.83 (d, *J* = 8.3 Hz, 1H), 8.21 (s, 1H), 7.71 (t, *J* = 8.3 Hz, 1H), 7.60–7.48 (m, 4H), 7.29 (br, 1H), 7.06–6.99 (m, 2H), 6.91 (d, *J* = 8.7 Hz, 2H), 6.81 (s, 1H), 4.95 (dd, *J* = 12.5, 5.3 Hz, 1H), 4.10 (q, *J* = 7.0 Hz, 2H), 3.99 (t, *J* = 6.5 Hz, 2H), 3.92 (s, 3H), 3.53 (t, *J* = 6.3 Hz, 2H), 2.95–2.76 (m, 5H), 2.45 (t, *J* = 7.5 Hz, 2H), 2.17 (m, 1H), 1.92 (s, 3H), 1.84–1.69 (m, 4H), 1.46 (t, *J* = 7.0 Hz, 3H), 1.48–1.32 (m, 12H); ^13^C NMR (151 MHz, CDCl_3_): δ 195.8 (C), 172.6 (C), 170.75 (C), 170.74 (C), 169.3 (C), 167.9 (C), 166.8 (C), 161.8 (C), 151.9 (C), 146.7 (CH), 146.2 (C), 138.1 (C), 136.6 (CH), 133.2 (C), 131.6 (C), 131.2 (C), 130.5 (CH), 127.0 (C), 125.4 (CH), 123.9 (CH), 118.6 (CH), 115.4 (C), 115.2 (CH), 113.7 (CH), 113.5 (CH), 68.4 (CH_2_), 65.0 (CH_2_), 56.2 (CH_3_), 49.4 (CH), 42.2 (CH_2_), 38.1 (CH_2_), 31.7 (CH_2_), 31.5 (CH_2_), 29.6 (CH_2_), 29.5 (CH_2_), 29.42 (CH_2_), 29.38 (CH_2_), 29.33 (CH_2_), 29.2 (CH_2_), 26.1 (CH_2_), 25.4 (CH_2_), 23.3 (CH_3_), 22.8 (CH_2_), 14.9 (CH_3_); HRMS (FAB) *m*/*z*: [M + H]^+^ calcd for C_46_H_55_N_4_O_10_, 823.3913; found, 823.3945.

##### **TKP-23** Isomer Mixture (7:3)

Major isomer: (*E*)-6-(2-(3-(4-(3-(2-(2-acetamidoethyl)-5-ethoxy-4-methoxyphenyl)-3-oxoprop-1-en-1-yl)­phenoxy)­prop-1-yn-1-yl)-5-methylphenyl)-*N*-(2-(2,6-dioxopiperidin-3-yl)-1,3-dioxoisoindolin-4-yl)­hexanamide.

Minor isomer: (*E*)-8-(2-((4-(3-(2-(2-acetamidoethyl)-5-ethoxy-4-methoxyphenyl)-3-oxoprop-1-en-1-yl)­phenoxy)­methyl)-4-methylphenyl)-*N*-(2-(2,6-dioxopiperidin-3-yl)-1,3-dioxoisoindolin-4-yl)­oct-7-ynamide.

Pd­(PPh_3_)_4_ (14.2 mg, 12.3 μmol) and 2-methylbut-1-en-3-yne (24.0 mg, 369 μmol) were added to a solution of **TKP-21** (100 mg, 123 μmol) in THF (1.6 mL). After 12 h of stirring at 65̊C, the reaction mixture was evaporated in vacuo. The crude product was directly purified by flash column chromatography (100:0 → 50:1 CHCl_3_/MeOH) to give the **TKP-23** isomer mixture (7:3, 88.2 mg, 100 μmol, 82%) as a yellow solid. *R*_f_ = 0.51 (8:1 CHCl_3_/MeOH); IR (neat) 3357, 2925, 2868, 2231, 1785, 1705, 1649, 1597, 1512, 1477, 1398, 1350, 1261, 1198, 1128, 825 cm^–1^; ^1^H NMR (600 MHz, CDCl_3_): δ 9.36 (s, 1H), 8.82 (s, 1H), 8.80–8.74 (m, 1H), 7.69–7.62 (m, 1H), 7.59–7.46 (m, 4H), 7.33–7.26 (m, 4H), 7.24 (s, 0.3H), 7.32–7.26 (m, 1.0H), 7.21 (s, 0.3H), 7.21 (br, 1H), 7.07–6.98 (m, 4.3H), 6.96 (s, 0.7H), 6.92 (d, *J* = 7.8 Hz, 0.7H), 6.79 (s, 1.0H), 5.19 (s, 0.6H), 4.97 (s, 1.4H), 4.94 (dd, *J* = 12.4, 5.4 Hz, 1H), 4.12–4.04 (m, 2H), 3.90 (s, 0.9H), 3.89 (s, 2.1H), 3.49 (q, *J* = 6.0 Hz, 2H), 2.90–2.72 (m, 5H), 2.43 (t, *J* = 6.9 Hz, 1.4H), 2.43 (t, *J* = 6.9 Hz, 0.6H), 2.41–2.35 (m, 2H), 2.31 (s, 0.9H), 2.29 (s, 2.1H), 2.14 (m, 1H), 1.902 (s, 0.9H), 1.898 (s, 2.1H), 1.76–1.66 (m, 2H), 1.63–1.55 (m, 2H), 1.55–1.49 (m, 0.6H), 1.42–1.36 (m, 3.0H), 1.35–1.29 (m, 1.4H); ^13^C NMR (151 MHz, CDCl_3_): δ 195.45 (C), 195.40 (C), 172.3 (C), 172.1 (C), 171.11 (C), 171.09 (C), 170.64 (C), 170.63 (C), 169.30 (C), 169.29 (C), 168.19 (C), 168.17 (C), 166.78 (C), 166.77 (C), 161.4 (C), 160.2 (C), 151.9 (C), 151.8 (C), 146.3 (CH), 146.12 (C), 146.11 (C), 146.09 (CH), 144.78 (C), 144.77 (C), 139.2 (C), 138.2 (C), 137.9 (C), 137.8 (C), 137.5 (C), 136.47 (CH), 136.45 (CH), 133.3 (C), 133.2 (C), 132.4 (CH), 132.2 (CH), 131.5 (C), 131.4 (C), 131.18 (C), 131.17 (C), 130.5 (CH), 130.3 (CH), 129.7 (CH), 128.8 (CH), 128.3 (CH), 127.8 (C), 127.4 (C), 126.7 (CH), 125.27 (CH), 125.25 (CH), 124.3 (CH), 124.0 (CH), 119.7 (C), 118.44 (CH), 118.38 (CH), 115.6 (CH), 115.5 (CH), 115.36 (C), 115.35 (C), 113.74 (CH), 113.73 (CH), 113.61 (CH), 113.57 (CH), 94.7 (C), 86.9 (C), 86.0 (C), 78.3 (C), 68.7 (CH_2_), 64.97 (CH_2_), 64.96 (CH_2_), 56.9 (CH_2_), 56.09 (CH_3_), 56.08 (CH_3_), 49.40 (CH), 49.39 (CH), 41.98 (CH_2_), 41.97 (CH_2_), 37.9 (CH_2_), 37.7 (CH_2_), 34.2 (CH_2_), 31.86 (CH_2_), 31.85 (CH_2_), 31.47 (CH_2_), 31.46 (CH_2_), 30.4 (CH_2_), 28.8 (CH_2_), 28.4 (CH_2_), 28.3 (CH_2_), 25.0 (CH_2_), 24.7 (CH_2_), 23.26 (CH_3_), 23.25 (CH_3_), 22.73 (CH_2_), 22.72 (CH_2_), 21.55 (CH_3_), 21.54 (CH_3_), 19.4 (CH_2_), 14.87 (CH_3_), 14.86 (CH_3_); HRMS (FAB) *m*/*z*: [M + H]^+^ calcd for C_51_H_53_N_4_O_10_, 881.3756; found, 881.3739.

##### **TKP-24** Isomer Mixture (7:3)

Major isomer: 6-(2-((*Z*)-3-(4-((*E*)-3-(2-(2-acetamidoethyl)-5-ethoxy-4-methoxyphenyl)-3-oxoprop-1-en-1-yl)­phenoxy)­prop-1-en-1-yl)-5-methylphenyl)-*N*-(2-(2,6-dioxopiperidin-3-yl)-1,3-dioxoisoindolin-4-yl)­hexanamide.

Minor isomer: (*Z*)-8-(2-((4-((*E*)-3-(2-(2-acetamidoethyl)-5-ethoxy-4-methoxyphenyl)-3-oxoprop-1-en-1-yl)­phenoxy)­methyl)-4-methylphenyl)-*N*-(2-(2,6-dioxopiperidin-3-yl)-1,3-dioxoisoindolin-4-yl)­oct-7-enamide.

The Lindlar catalyst (TCI, P1703, Lot LYEMO-QL, 32.0 mg) and quinoline (1 drop) were added to a solution of **TKP-23** (80.0 mg, 90.6 μmol) in AcOEt/MeOH (2:1, 1.5 mL), and the mixture was stirred under hydrogen for 40 h. The reaction mixture was filtered through a Celite pad, and the filtrate was dried over anhydrous Na_2_SO_4_. Filtration and evaporation in vacuo furnished the crude product, which was purified by flash column chromatography (100:0 → 50:1 CHCl_3_/MeOH) to give the **TKP-24** isomer mixture (7:3, 68.0 mg, 76.8 μmol, 85%) as a yellow solid. *R*_f_ = 0.51 (8:1 CHCl_3_/MeOH); IR (neat) 3354, 3309, 2922, 2850, 1768, 1705, 1660, 1597, 1512, 1398, 1350, 1261, 1198, 1130, 1026, 1003, 825, 750 cm^–1^; ^1^H NMR (597 MHz, CDCl_3_): δ 9.37 (s, 1H), 8.80 (m, 1H), 8.70 (br, 1H), 7.68 (t, *J* = 7.8 Hz, 1H), 7.60–7.41 (m, 4H), 7.23 (br, 1H), 7.16–6.73 (m, 8.7H), 6.47 (d, *J* = 11.4 Hz, 0.3H), 5.96 (dt, *J* = 11.9, 6.4 Hz, 0.7H), 5.72 (dt, *J* = 11.4, 7.4 Hz, 0.3H), 5.00 (s, 0.6H), 4.94 (m, 1H), 4.68 (d, *J* = 6.3 Hz, 1.4H), 4.15–4.05 (m, 2H), 3.911 (s, 0.9H), 3.906 (s, 2.1H), 3.55–3.48 (m, 2H), 2.95–2.67 (m, 5H), 2.59 (t, *J* = 7.7 Hz, 1.4H), 2.48–2.36 (m, 2H), 2.33 (s, 0.9H), 2.32 (s, 2.1H), 2.18–2.11 (m, 1.6H), 1.95–1.87 (m, 3H), 1.76 (quint, *J* = 7.7 Hz, 1.4H), 1.69 (quint, *J* = 7.6 Hz, 0.6H), 1.60 (quint, *J* = 7.7 Hz, 1.4H), 1.50–1.40 (m, 5H), 1.39–1.33 (m, 0.6H); ^13^C NMR (150 MHz, CDCl_3_): δ 195.62 (C), 195.60 (C), 172.30 (C), 172.29 (C), 171.02 (C), 170.99 (C), 170.67 (C), 170.66 (C), 169.30 (C), 169.29 (C), 168.13 (C), 168.10 (C), 166.78 (C), 166.77 (C), 161.42 (C), 161.03 (C), 151.83 (C), 151.82 (C), 146.42 (CH), 146.40 (CH), 146.13 (C), 146.12 (C), 140.71 (C), 137.93 (C), 137.89 (C), 136.99 (C), 136.53 (CH), 136.52 (CH), 136.51 (C), 133.98 (CH), 133.79 (C), 133.74 (C), 133.25 (C), 133.22 (C), 132.48 (CH), 131.76 (C), 131.49 (C), 131.19 (C), 131.18 (C), 130.48 (CH), 130.40 (CH), 130.20 (CH), 129.61 (CH), 129.35 (CH), 129.34 (C), 129.18 (CH), 128.81 (CH), 127.37 (C), 127.23 (C), 126.57 (CH), 126.54 (CH), 126.39 (CH), 125.33 (CH), 125.32 (CH), 124.06 (CH), 123.99 (CH), 118.51 (CH), 118.49 (CH), 115.47 (C), 115.41 (CH), 115.35 (C), 115.34 (CH), 113.70 (CH), 113.68 (CH), 113.49 (CH), 113.48 (CH), 68.47 (CH_2_), 65.10 (CH_2_), 64.96 (CH_2_), 64.95 (CH_2_), 56.11 (CH_3_), 56.10 (CH_3_), 49.39 (CH), 49.38 (CH), 42.05 (CH_2_), 42.04 (CH_2_), 37.90 (CH_2_), 37.87 (CH_2_), 33.27 (CH_2_), 31.78 (CH_2_), 31.76 (CH_2_), 31.48 (CH_2_), 31.47 (CH_2_), 30.53 (CH_2_), 29.44 (CH_2_), 29.06 (CH_2_), 28.79 (CH_2_), 28.30 (CH_2_), 25.10 (CH_2_), 25.09 (CH_2_), 23.28 (CH_3_), 23.27 (CH_3_), 22.75 (CH_2_), 22.74 (CH_2_), 21.29 (CH_3_), 21.27 (CH_3_), 14.87 (CH_3_), 14.86 (CH_3_); HRMS (FAB) *m*/*z*: [M + H]^+^ calcd for C_51_H_55_N_4_O_10_, 883.3913; found, 883.3924.

##### **TKP-43** Isomer Mixture (3:2)

Major isomer: (*E*)-8-(2-((4-(3-(2-(2-acetamidoethyl)-5-ethoxy-4-methoxyphenyl)-3-oxo prop-1-en-1-yl)­phenoxy)­methyl)-4-methylphenyl)-*N*-(2-(2,6-dioxopiperidin-3-yl)-1,3-dioxoisoindolin-4-yl)-8-oxooctanamide.

Minor isomer: (*E*)-8-(2-((4-(3-(2-(2-acetamidoethyl)-5-ethoxy-4-methoxyphenyl)-3-oxo prop-1-en-1-yl)­phenoxy)­methyl)-4-methylphenyl)-*N*-(2-(2,6-dioxopiperidin-3-yl)-1,3-dioxoisoindolin-4-yl)-7-oxooctanamide.

AgSbF_6_ (3.89 mg, 11.3 μmol) and IPrAuCl (7.03 mg, 11.3 μmol) were added to a solution of **TKP-23** (100 mg, 113 μmol) in 1,4-dioxane/H_2_O (2:1, 12 mL), and the mixture was stirred at 120 °C for 24 h. The reaction mixture was filtered through a Celite pad, and the filtrate was extracted with CHCl_3_ (2 × 10 mL). The combined organic extracts were washed with brine (20 mL) and dried over anhydrous Na_2_SO_4_. Filtration and evaporation in vacuo furnished the crude product, which was purified by flash column chromatography (100:0 → 50:1, CHCl_3_/MeOH) to give the **TKP-43** isomer mixture (3:2, 28.7 mg, 31.9 μmol, 28%) as a white solid. *R*_f_ = 0.50 (8:1 CHCl_3_/MeOH); IR (neat) 2929, 2862, 1768, 1705, 1597, 1522, 1390, 1344, 1254, 1213, 1134, 771 cm^–1^; ^1^H NMR (600 MHz, CDCl_3_): δ 9.40 (s, 0.6H), 9.38 (s, 0.4H), 8.81 (d, *J* = 8.5 Hz, 0.4H), 8.79 (d, *J* = 8.5 Hz, 0.6H), 8.42 (s, 0.4H), 8.36 (s, 0.6H), 7.76 (d, *J* = 7.9 Hz, 0.4H), 7.70 (dd, *J* = 8.5, 8.0 Hz, 0.6H), 7.68 (dd, *J* = 8.5, 8.0 Hz, 0.4H), 7.56–7.48 (m, 4.6H), 7.26–7.09 (m, 3H), 7.07–7.00 (m, 3.2H), 6.96 (d, *J* = 8.8 Hz, 0.8H), 6.81 (s, 1H), 5.43 (s, 1.2H), 4.98 (s, 0.8H), 4.94 (m, 1H), 4.10 (q, *J* = 7.0 Hz, 2H), 3.92 (s, 3H), 3.75 (s, 0.8H), 3.55–3.49 (m, 2H), 2.96 (t, *J* = 7.3 Hz, 1.2H), 2.92–2.70 (m, 5H), 2.45 (t, *J* = 7.4 Hz, 2H), 2.42 (s, 1.8H), 2.39 (t, *J* = 7.6 Hz, 1H), 2.34 (s, 1.2H), 2.15 (m, 1H), 1.92 (s, 3H), 1.75–1.69 (m, 2.8H), 1.62–1.55 (m, 1.2H), 1.49–1.44 (m, 3H), 1.45–1.40 (m, 2H), 1.36–1.29 (m, 1.2H); ^13^C NMR (150 MHz, CDCl_3_): δ 208.1 (C), 203.1 (C), 195.7 (C), 195.6 (C), 172.4 (C), 172.2 (C), 170.9 (C), 170.8 (C), 170.69 (C), 170.68 (C), 169.33 (C), 169.31 (C), 168.01 (C), 167.98 (C), 166.80 (C), 166.79 (C), 161.3 (C), 161.1 (C), 151.89 (C), 151.86 (C), 146.5 (C), 146.3 (C), 146.18 (CH), 146.17 (CH), 143.2 (C), 138.01 (C), 137.99 (C), 137.93 (C), 137.5 (C), 136.58 (CH), 136.57 (CH), 134.5 (C), 133.3 (C), 133.2 (C), 132.9 (C), 131.6 (C), 131.5 (C), 131.4 (C), 131.2 (CH), 130.71 (CH), 130.67 (CH), 130.57 (CH), 130.56 (CH), 130.55 (CH), 129.83 (CH), 129.81 (CH), 128.5 (C), 128.2 (C), 127.7 (C), 127.4 (C), 125.4 (CH), 125.3 (CH), 124.2 (CH), 124.1 (CH), 118.58 (CH), 118.57 (CH), 115.60 (CH), 115.59 (CH), 115.4 (C), 115.3 (C), 113.73 (CH), 113.69 (CH), 113.51 (CH), 113.5 (CH), 69.1 (CH_2_), 68.7 (CH_2_), 65.00 (CH_2_), 64.99 (CH_2_), 56.15 (CH_3_), 56.14 (CH_3_), 49.42 (CH), 49.40 (CH), 47.3 (CH_2_), 42.11 (CH_2_), 42.08 (CH_2_), 41.9 (CH_2_), 40.5 (CH_2_), 38.0 (CH_2_), 37.7 (CH_2_), 31.8 (CH_2_), 31.7 (CH_2_), 31.51 (CH_2_), 31.49 (CH_2_), 29.08 (CH_2_), 29.07 (CH_2_), 28.6 (CH_2_), 25.2 (CH_2_), 25.0 (CH_2_), 24.3 (CH_2_), 23.4 (CH_2_), 23.34 (CH_3_), 23.33 (CH_3_), 22.80 (CH_2_), 22.78 (CH_2_), 21.9 (CH_3_), 21.2 (CH_3_), 14.91 (CH_3_), 14.90 (CH_3_); HRMS (FAB) *m*/*z*: [M + H]^+^ calcd for C_51_H_55_N_4_O_11_, 899.3862; found, 899.3878.

##### **TKP-25** Isomer Mixture (3:7)

Major isomer: (*E*)-6-(2-(3-(4-(3-(2-(2-acetamidoethyl)-5-ethoxy-4-methoxyphenyl)-3-oxoprop-1-en-1-yl)­phenoxy)­propyl)-5-methylphenyl)-*N*-(2-(2,6-dioxopiperidin-3-yl)-1,3-dioxoisoindolin-4-yl)­hexanamide.

Minor isomer: (*E*)-8-(2-((4-(3-(2-(2-acetamidoethyl)-5-ethoxy-4-methoxyphenyl)-3-oxoprop-1-en-1-yl)­phenoxy)­methyl)­phenyl)-*N*-(2-(2,6-dioxopiperidin-3-yl)-1,3-dioxoisoindolin-4-yl) octanamide.

5% Pd/C (8.0 mg) was added to a solution of **TKP-23** (77.6 mg, 87.9 μmol) in MeOH/AcOEt (1:1, 8.8 mL), and the mixture was stirred under hydrogen for 20 h. The reaction mixture was filtered through a Celite pad, and the filtrate was dried over anhydrous Na_2_SO_4_. Filtration and evaporation in vacuo furnished the crude product (55.9 mg), which was used without further purification.

Pd­(TFA)_2_ (25.1 mg, 75.6 μmol) and 4,5-diazafluoren-9-one (**41**) (13.7 mg, 75.6 μmol) were added to a solution of crude ketone (55.9 mg) in DMSO (1.3 mL), and the reaction mixture was purged with O_2_ for 3 times. After 24 h of stirring at 100 °C under O_2_, the reaction was quenched with saturated aqueous NaHCO_3_ (3 mL) at 20 °C, and the mixture was extracted with CHCl_3_ (3 × 10 mL). The combined organic extracts were washed with brine (30 mL) and dried over anhydrous Na_2_SO_4_. Filtration and evaporation in vacuo furnished the crude product, which was purified by flash column chromatography (50:1 → 20:1, CHCl_3_/MeOH) to give the **TKP-25** isomer mixture (7:3, 29.6 mg, 33.4 μmol, 38% in 2 steps) as a yellow solid. *R*_f_ = 0.51 (8:1 CHCl_3_/MeOH); IR (neat) 3342, 3080, 2920, 1768, 1705, 1660, 1618, 1593, 1514, 1396, 1348, 1259, 1201, 1126, 1030, 825, 677 cm^–1^; ^1^H NMR (597 MHz, CDCl_3_): δ 9.40 (s, 0.7H), 9.39 (s, 0.3H), 8.85–8.78 (m, 1H), 8.51 (s, 1H), 7.74–7.66 (m, 1H), 7.59–7.47 (m, 4H), 7.23–6.88 (m, 8H), 6.81 (s, 1H), 5.05 (s, 0.6H), 4.95 (m, 1H), 4.10 (q, *J* = 7.0 Hz, 2H), 4.01 (t, *J* = 6.1 Hz, 1.4H), 3.92 (s, 3H), 3.52 (q, *J* = 5.8 Hz, 2H), 2.92–2.74 (m, 6.4H), 2.63–2.58 (m, 2H), 2.45 (t, *J* = 7.6 Hz, 1.4H), 2.41 (t, *J* = 7.6 Hz, 0.6H), 2.32 (s, 0.9H), 2.29 (s, 2.1H), 2.15 (m, 1H), 2.06 (quint, *J* = 6.1 Hz, 1H), 1.92 (s, 3H), 1.79 (quint, *J* = 7.6 Hz, 1.4H), 1.71 (quint, *J* = 7.6 Hz, 0.6H), 1.66–1.56 (m, 2H), 1.52–1.43 (m, 4.4H), 1.38–1.33 (m, 1.8H); ^13^C NMR (150 MHz, CDCl_3_): δ 195.68 (C), 195.64 (C), 172.44 (C), 172.33 (C), 170.90 (C), 170.89 (C), 170.67 (C), 170.66 (C), 169.33 (C), 169.32 (C), 168.06 (C), 168.04 (C), 166.79 (C), 166.78 (C), 161.59 (C), 161.41 (C), 151.87 (C), 151.85 (C), 146.54 (CH), 146.42 (CH), 146.17 (C), 146.16 (C), 140.29 (C), 138.57 (C), 137.99 (C), 137.96 (C), 136.57 (CH), 136.56 (CH), 135.86 (C), 135.79 (C), 133.39 (C), 133.26 (C), 133.24 (C), 131.55 (C), 131.52 (C), 131.21 (C), 131.20 (C), 130.55 (CH), 130.52 (CH), 130.20 (CH), 130.16 (CH), 129.72 (CH), 129.54 (CH), 129.48, 129.31 (CH), 127.44 (C), 127.13 (C), 126.92 (CH), 125.36 (CH), 125.35 (CH), 124.13 (CH), 123.94 (CH), 118.56 (CH), 118.53 (CH), 115.40 (CH), 115.38 (C), 115.36 (C), 115.15 (CH), 113.72 (CH), 113.69 (CH), 113.51 (CH), 113.50 (CH), 68.66 (CH_2_), 67.37 (CH_2_), 65.00 (CH_2_), 64.98 (CH_2_), 56.14 (CH_3_), 56.13 (CH_3_), 49.41 (CH), 49.39 (CH), 42.09 (CH_2_), 42.08 (CH_2_), 38.02 (CH_2_), 38.01 (CH_2_), 32.50 (CH_2_), 32.21 (CH_2_), 31.80 (CH_2_), 31.50 (CH_2_), 31.49 (CH_2_), 31.33 (CH_2_), 31.20 (CH_2_), 31.19 (CH_2_), 30.69 (CH_2_), 29.50 (CH_2_), 29.39 (CH_2_), 29.22 (CH_2_), 29.09 (CH_2_), 28.38 (CH_2_), 25.27 (CH_2_), 25.20 (CH_2_), 23.32 (CH_3_), 23.31 (CH_3_), 22.79 (CH_2_), 22.78 (CH_2_), 21.11 (CH_3_), 21.06 (CH_3_), 14.90 (CH_3_), 14.89 (CH_3_); HRMS (FAB) *m*/*z*: [M + H]^+^ calcd for C_51_H_57_N_4_O_10_, 885.4069; found, 885.4073.

#### General Procedure D: RuAAC Reaction

After freeze–pump–thaw cycling, a mixture of **TKP-21** (1.0 equiv) and the corresponding azide (1.1 equiv) in THF (0.05 M) was added to a flask containing Cp*Ru­(cod)Cl (30 mol %), and the resulting mixture was stirred for 6 h. The reaction mixture was filtered through a Celite pad, and the filtrate was dried over anhydrous Na_2_SO_4_. Filtration and evaporation in vacuo furnished the crude product, which was purified by flash column chromatography (100:0 → 50:1 CHCl_3_/MeOH) to give **TKP-27**, **28**, **29**, **36**, and **37** (41–90%) as yellow solids.

##### **TKP-36**: (*E*)-8-(5-((4-(3-(2-(2-Acetamidoethyl)-5-ethoxy-4-methoxyphenyl)-3-oxoprop-1-en-1-yl)­phenoxy)­methyl)-1-benzyl-1*H*-1,2,3-triazol-4-yl)-*N*-(2-(2,6-dioxopiperidin-3-yl)-1,3-dioxoisoindolin-4-yl)­oct-7-ynamide

Following general procedure D, **TKP-36** was prepared in 90% yield as a yellow solid using **TKP-21** and benzylazide. *R*_f_ = 0.52 (8:1 CHCl_3_/MeOH); IR (neat) 3381, 2941, 2858, 2239, 1776, 1705, 1601, 1512, 1350, 1261, 1190, 1130, 1018, 748 cm^–1^; ^1^H NMR (600 MHz, CDCl_3_): δ 9.37 (s, 1H), 8.75 (d, *J* = 8.4 Hz, 1H), 8.46 (s, 1H), 7.65 (t, *J* = 8.4 Hz, 1H), 7.53–7.48 (m, 4H), 7.33–7.26 (m, 4H), 7.15 (m, 1H), 7.12 (br, 1H), 7.03 (d, *J* = 15.8 Hz, 1H), 7.03 (s, 1H), 6.87 (d, *J* = 8.8 Hz, 2H), 6.80 (s, 1H), 5.59 (s, 2H), 4.95 (s, 2H), 4.93 (m, 1H), 4.08 (q, *J* = 7.0 Hz, 2H), 3.90 (s, 3H), 3.55–3.48 (m, 2H), 2.92–2.73 (m, 5H), 2.48 (t, *J* = 7.2 Hz, 2H), 2.43 (t, *J* = 7.3 Hz, 2H), 2.15 (m, 1H), 1.90 (s, 3H), 1.76 (quint, *J* = 7.2 Hz, 2H), 1.67 (quint, *J* = 7.3 Hz, 2H), 1.60–1.50 (m, 2H), 1.44 (t, *J* = 7.0 Hz, 3H); ^13^C NMR (151 MHz, CDCl_3_): δ 195.4 (C), 172.1 (C), 170.9 (C), 170.6 (C), 169.3 (C), 168.1 (C), 166.8 (C), 159.5 (C), 152.0 (C), 146.2 (C), 145.7 (CH), 137.9 (C), 136.5 (CH), 134.0 (C), 133.5 (C), 133.1 (C), 132.5 (C), 131.4 (C), 131.2 (C), 130.5 (CH), 129.1 (CH), 128.8 (CH), 128.5 (C), 127.8 (CH), 125.3 (CH), 124.8 (CH), 118.5 (CH), 115.5 (C), 115.2 (CH), 113.8 (CH), 113.7 (CH), 96.6 (C), 69.5 (C), 65.1 (CH_2_), 57.8 (CH_2_), 56.2 (CH_3_), 53.3 (CH_2_), 49.4 (CH), 42.0 (CH_2_), 37.7 (CH_2_), 31.9 (CH_2_), 31.5 (CH_2_), 28.4 (CH_2_), 28.1 (CH_2_), 24.7 (CH_2_), 23.3 (CH_3_), 22.8 (CH_2_), 19.4 (CH_2_), 14.9 (CH_3_); HRMS (FAB) *m*/*z*: [M + H]^+^ calcd for C_53_H_54_N_7_O_10_, 948.3927; found, 948.3923. HPLC purity = 95%.

##### **TKP-29** Isomer Mixture (3:2)

Major isomer: *tert*-butyl (*E*)-(3-(5-((4-(3-(2-(2-acetamidoethyl)-5-ethoxy-4-methoxyphenyl)-3-oxoprop-1-en-1-yl)­phenoxy)­methyl)-4-(8-((2-(2,6-dioxopiperidin-3-yl)-1,3-dioxoisoindolin-4-yl)­amino)-8-oxooct-1-yn-1-yl)-1*H*-1,2,3-triazol-1-yl)­propyl)­carbamate.

Minor isomer: *tert*-butyl (*E*)-(3-(4-((4-(3-(2-(2-acetamidoethyl)-5-ethoxy-4-methoxyphenyl)-3-oxoprop-1-en-1-yl)­phenoxy)­methyl)-5-(8-((2-(2,6-dioxopiperidin-3-yl)-1,3-dioxoisoindolin-4-yl)­amino)-8-oxooct-1-yn-1-yl)-1*H*-1,2,3-triazol-1-yl)­propyl)­carbamate.

Following general procedure D, the **TKP-29** isomer mixture (3:2) was prepared in 60% yield as a yellow solid using **TKP-21** and *tert*-butyl (3-azidopropyl)­carbamate. *R*_f_ = 0.49 (8:1 CHCl_3_/MeOH); IR (neat) 3357, 2974, 2933, 2870, 2235, 1768, 1705, 1657, 1618, 1599, 1512, 1479, 1396, 1350, 1261, 1176, 1130, 989, 825, 752, 667 cm^–1^; ^1^H NMR (600 MHz, CDCl_3_): δ 9.38 (s, 0.6H), 9.38 (s, 0.4H), 8.76 (s, 0.6H), 8.75 (s, 0.4H), 8.74 (br, 1.0H), 7.72–7.62 (m, 1H), 7.55–7.45 (m, 4H), 7.20 (br, 0.6H), 7.1 (br, 0.4H), 7.08–6.95 (m, 4H), 6.80 (s, 0.6H), 6.80 (s, 0.4H), 5.18 (s, 1.2H), 5.17 (s, 0.8H), 4.95 (m, 1H), 4.93 (br, 0.4H), 4.84 (s, 0.6H), 4.40 (t, *J* = 6.7 Hz, 2H), 4.08 (q, *J* = 7.0 Hz, 2H), 3.90 (s, 3H), 3.53–3.44 (m, 2H), 3.18–3.07 (m, 2H), 2.90–2.72 (m, 5H), 2.53 (t, *J* = 6.9 Hz, 0.8H), 2.50 (t, *J* = 6.9 Hz, 1.2H), 2.46–2.36 (m, 2H), 2.15 (m, 1H), 2.14–2.04 (m, 2H), 1.90 (s, 3H), 1.83–1.71 (m, 2H), 1.73–1.64 (m, 2H), 1.59–1.50 (m, 2H), 1.44 (t, *J* = 7.0 Hz, 3H), 1.42 (s, 3.6H), 1.40 (s, 5.4H); ^13^C NMR (151 MHz, CDCl_3_): δ 195.5 (C), 195.3 (C), 172.1 (C), 172.0 (C), 171.1 (C), 171.0 (C), 170.64 (C), 170.61 (C), 169.4 (C), 169.3 (C), 168.20 (C), 168.19 (C), 166.8 (C), 166.7 (C), 160.9 (C), 159.5 (C), 156.2 (C), 156.1 (C), 152.0 (C), 151.9 (C), 146.18 (C), 146.16 (C), 145.58 (CH), 145.56 (CH), 144.6 (C), 137.84 (C), 137.81 (C), 136.6 (CH), 136.5 (CH), 133.4 (C), 133.3 (C), 133.2 (C), 132.1 (C), 131.4 (C), 131.3 (C), 131.24 (C), 131.22 (C), 130.5 (CH), 130.4 (CH), 128.6 (C), 127.7 (C), 125.3 (CH), 125.2 (CH), 124.9 (CH), 124.2 (CH), 121.7 (C), 118.6 (CH), 118.5 (CH), 115.53 (C), 115.52 (C), 115.4 (CH), 115.3 (CH), 113.8 (CH), 113.8 (CH), 113.7 (CH), 113.6 (CH), 104.5 (C), 96.5 (C), 79.6 (C), 69.6 (C), 65.37 (C), 65.36 (C), 65.04 (CH_2_), 65.00 (CH_2_), 61.2 (CH_2_), 57.8 (CH_2_), 56.13 (CH_3_), 56.12 (CH_3_), 49.44 (CH), 49.42 (CH), 46.8 (CH_2_), 46.6 (CH_2_), 42.01 (CH_2_), 42.00 (CH_2_), 37.7 (CH_2_), 37.6 (CH_2_), 37.54 (CH_2_), 37.48 (CH_2_), 31.91 (CH_2_), 31.88 (CH_2_), 31.50 (CH_2_), 31.47 (CH_2_), 30.11 (CH_2_), 30.09 (CH_2_), 28.49 (CH_3_), 28.46 (CH_3_), 28.4 (CH_2_), 28.3 (CH_2_), 28.0 (CH_2_), 27.8 (CH_2_), 24.7 (CH_2_), 24.6 (CH_2_), 23.30 (CH_3_), 23.29 (CH_3_), 22.8 (CH_2_), 22.7 (CH_2_), 19.6 (CH_2_), 19.4 (CH_2_), 14.89 (CH_3_), 14.88 (CH_3_); HRMS (FAB) *m*/*z*: [M + H]^+^ calcd for C_54_H_63_N_8_O_12_, 1015.4560; found, 1015.4534.

##### (*E*)-*N*-(2-(3-(4-(3-Azidopropoxy)­phenyl)­acryloyl)-4-ethoxy-5-methoxyphenethyl) Acetamide (**42**)

K_2_CO_3_ (360 mg, 2.61 mmol) and 3-azidopropyl methanesulfonate (280 mg, 1.56 mmol) were added to a solution of phenol **S1** (500 mg, 1.30 mmol) in DMF (6.5 mL). After 8 h of stirring at rt, the reaction was quenched with saturated aqueous NH_4_Cl (20 mL), and the mixture was extracted with AcOEt (3 × 20 mL). The combined organic extracts were washed with brine (3 × 50 mL) and dried over anhydrous Na_2_SO_4_. Filtration and evaporation in vacuo furnished the crude product, which was purified by flash column chromatography (50:1 → 20:1, CHCl_3_/MeOH) to give azide **42** (430 mg, 71%) as a yellow solid. IR (neat) 3298, 2981, 2935, 2877, 2098, 1655, 1597, 1566, 1512, 1469, 1350, 1259, 1174, 1130, 1039, 985, 827, 754 cm^–1^; ^1^H NMR (399 MHz, CDCl_3_): δ 7.59–7.48 (m, 3H), 7.27 (s, 1H), 7.09–7.00 (m, 2H), 6.93 (d, *J* = 8.7 Hz, 2H), 6.81 (s, 1H), 4.15–4.07 (m, 4H), 3.93 (s, 3H), 3.58–3,50 (t, *J* = 6.4 Hz, 4H), 2.86 (t, *J* = 6.4 Hz, 2H), 2.08 (quint, *J* = 6.3 Hz, 2H), 1.92 (s, 3H), 1.47 (t, *J* = 7.0 Hz, 3H); ^13^C NMR (101 MHz, CDCl_3_): δ 195.6 (C), 170.6 (C), 161.1 (C), 151.8 (C), 146.3 (CH), 146.2 (C), 133.2 (C), 131.5 (C), 130.5 (CH), 127.4 (C), 124.1 (CH), 115.1 (CH), 113.6 (CH), 113.4 (CH), 64.9 (CH_2_), 64.8 (CH_2_), 56.1 (CH_3_), 48.2 (CH_2_), 42.1 (CH_2_), 31.6 (CH_2_), 28.7 (CH_2_), 23.3 (CH_3_), 14.9 (CH_3_); HRMS (FAB) *m*/*z*: [M + H]^+^ calcd for C_25_H_31_N_4_O_5_, 467.2289; found, 467.2304.

##### **TKP-27** Isomer Mixture (4:1)

8-(5-((4-((*E*)-3-(2-(2-Acetamidoethyl)-5-ethoxy-4-methoxyphenyl)-3-oxoprop-1-en-1-yl) phenoxy)­methyl)-1-(3-(4-((*E*)-3-(2-(2-acetamidoethyl)-5-ethoxy-4-methoxyphenyl)-3-oxoprop-1-en-1-yl)­phenoxy)­propyl)-1*H*-1,2,3-triazol-4-yl)-*N*-(2-(2,6-dioxopiperidin-3-yl)-1,3-dioxoisoindolin-4-yl)­oct-7-ynamide.

8-(4-((4-((*E*)-3-(2-(2-Acetamidoethyl)-5-ethoxy-4-methoxyphenyl)-3-oxoprop-1-en-1-yl) phenoxy)­methyl)-1-(3-(4-((*E*)-3-(2-(2-acetamidoethyl)-5-ethoxy-4-methoxyphenyl)-3-oxoprop-1-en-1-yl)­phenoxy)­propyl)-1*H*-1,2,3-triazol-5-yl)-*N*-(2-(2,6-dioxopiperidin-3-yl)-1,3-dioxoisoindolin-4-yl)­oct-7-ynamide.

Following general procedure D, the **TKP-27** isomer mixture (4:1) was prepared in 50% yield as a yellow solid using **TKP-21** and azide **42**. *R*_f_ = 0.50 (8:1 CHCl_3_/MeOH); IR (neat) 3357, 3323, 2935, 2866, 2243, 1772, 1705, 1657, 1597, 1512, 1477, 1396, 1350, 1259, 1176, 1130, 985, 825, 750 cm^–1^; ^1^H NMR (597 MHz, CDCl_3_): δ 9.37 (s, 0.8H), 9.33 (s, 0.2H), 8.78–8.72 (m, 1H), 8.72–8.60 (m, 1H), 7.71–7.63 (m, 1H), 7.54–7.47 (m, 7H), 7.20 (brs, 1H), 7.11 (brs, 1H), 7.06–7.01 (m, 4H), 6.96 (d, *J* = 8.8 Hz, 2H), 6.90–6.78 (m, 4H), 5.18 (s, 0.4H), 5.16 (s, 1.6H), 4.95 (m, 1H), 4.59 (t, *J* = 6.7 Hz, 1.6H), 4.59 (t, *J* = 6.7 Hz, 0.4H), 4.12–4.05 (m, 4H), 4.02 (t, *J* = 5.8 Hz, 2H), 3.914 (s, 3H), 3.910 (s, 3H), 3.57–3.45 (m, 4H), 2.98–2.71 (m, 7H), 2.50 (t, *J* = 7.0 Hz, 1.6H), 2.46–2.38 (m, 4H), 2.33 (t, 0.4H), 2.16 (m, 1H), 1.91 (s, 6H), 1.78 (quint, *J* = 7.1 Hz, 2H), 1.69 (quint, *J* = 7.1 Hz, 2H), 1.56 (quint, 7.1 Hz, 2H), 1.48–1.40 (m, 6H); ^13^C NMR (150 MHz, CDCl_3_, Only the major isomer peaks are reported.): δ 195.5 (C), 195.3 (C), 172.1 (C), 171.0 (C), 170.6 (C), 170.6 (C), 169.3 (C), 168.1 (C), 166.8 (C), 160.7 (C), 159.5 (C), 152.0 (C), 151.9 (C), 146.2 (C), 146.1 (C), 146.1 (CH), 145.5 (CH), 137.9 (C), 136.5 (CH), 133.5 (C), 133.4 (C), 133.3 (C), 132.1 (C), 131.4 (C), 131.3 (C), 131.2 (C), 130.5 (CH), 130.4 (CH), 128.7 (C), 127.7 (C), 125.3 (CH), 124.9 (CH), 124.4 (CH), 124.2 (C), 118.5 (CH), 115.4 (CH), 115.3 (CH), 115.1 (CH), 113.8 (CH), 113.8 (CH), 113.6 (CH), 96.6 (C), 69.5 (C), 65.1 (CH_2_), 65.0 (CH_2_), 64.4 (CH_2_), 57.8 (CH_2_), 56.1 (CH_3_), 56.1 (CH_3_), 49.4 (CH), 45.9 (CH_2_), 42.1 (CH_2_), 42.0 (CH_2_), 37.7 (CH_2_), 32.0 (CH_2_), 31.8 (CH_2_), 31.5 (CH_2_), 29.5 (CH_2_), 28.4 (CH_2_), 28.1 (CH_2_), 24.7 (CH_2_), 23.3 (CH_3_), 23.3 (CH_3_), 22.8 (CH_2_), 19.4 (CH_2_), 14.9 (CH_3_), 14.9 (CH_3_); HRMS (FAB) *m*/*z*: [M + H]^+^ calcd for C_71_H_77_N_8_O_5_, 1281.5503; found, 1281.5492. HPLC purity = > 99%.

##### **TKP-28**: (*E*)-8-(5-((4-(3-(2-(2-Acetamidoethyl)-5-ethoxy-4-methoxyphenyl)-3-oxoprop-1-en-1-yl)­phenoxy)­methyl)-1-(6-((2-(2,6-dioxopiperidin-3-yl)-1,3-dioxoisoindolin-4-yl)­amino)-6-oxohexyl)-1*H*-1,2,3-triazol-4-yl)-*N*-(2-(2,6-dioxopiperidin-3-yl)-1,3-dioxoisoindolin-4-yl)­oct-7-ynamide

Following general procedure D, **TKP-28** was prepared in 41% yield as a yellow solid using **TKP-21** and azide **29**. *R*_f_ = 0.50 (8:1 CHCl_3_/MeOH); IR (neat) 3356, 3113, 2916, 2856, 2268, 1707, 1614, 1525, 1479, 1394, 1348, 1287, 1203, 1124, 1028, 993, 771 cm^–1^; ^1^H NMR (597 MHz, CDCl_3_): δ 9.47–9.25 (m, 2H), 8.98–8.56 (m, 4H), 7.71–7.64 (m, 2H), 7.59–7.44 (m, 5H), 7.10 (br, 1H), 7.07–7.01 (m, 2H), 6.99 (d, *J* = 8.6 Hz, 2H), 6.80 (s, 1H), 5.17 (s, 2H), 4.96 (m, 2H), 4.36 (t, *J* = 7.0 Hz, 2H), 4.08 (q, *J* = 6.8 Hz, 2H), 3.91 (s, 3H), 3.50 (q, *J* = 5.8 Hz, 2H), 2.99–2.64 (m, 8H), 2.50 (t, *J* = 7.1 Hz, 2H), 2.46–2.35 (m, 4H), 2.22–2.10 (m, 2H), 1.98 (quint, 7.0 Hz, 2H), 1.91 (s, 3H), 1.82–1.71 (m, 4H), 1.69 (quint, *J* = 7.1 Hz, 2H), 1.57 (quint, *J* = 7.1 Hz, 2H), 1.43 (t, *J* = 6.8 Hz, 3H), 1.44–1.38 (m, 2H); ^13^C NMR (150 MHz, CDCl_3_): δ 195.3 (C), 172.2 (C), 171.9 (C), 171.3 (C), 171.2 (C), 170.7 (C), 169.3 (C), 169.33 (C), 168.32 (C), 168.3 (C), 166.80 (C), 166.78 (C), 159.4 (C), 152.0 (C), 146.2 (C), 145.5 (CH), 137.85 (C), 137.78 (C), 136.6 (CH), 136.5 (CH), 133.5 (C), 133.0 (C), 132.0 (C), 131.3 (C), 131.2 (C), 130.6 (CH), 128.6 (C), 127.6 (C), 125.4 (CH), 125.3 (CH), 124.8 (CH), 118.7 (CH), 118.6 (CH), 115.52 (C), 115.46 (C), 115.2 (CH), 113.9 (CH), 113.8 (CH), 96.5 (C), 69.7 (C), 65.1 (CH_2_), 57.8 (CH_2_), 56.2 (CH_3_), 49.44 (CH), 49.41 (CH), 49.1 (CH_2_), 42.0 (CH_2_), 37.8 (CH_2_), 37.5 (CH_2_), 32.1 (CH_2_), 31.51 (CH_2_), 31.50 (CH_2_), 29.6 (CH_2_), 28.4 (CH_2_), 28.0 (CH_2_), 26.1 (CH_2_), 24.7 (CH_2_), 24.5 (CH_2_), 23.3 (CH_3_), 22.80 (CH_2_), 22.78 (CH_2_), 19.4 (CH_2_), 14.9 (CH_3_); HRMS (FAB) *m*/*z*: [M + H]^+^ calcd for C_65_H_67_N_10_O_15_, 1227.4782; found, 1227.4809.

##### **TKP-37**: (*E*)-8-(5-((4-(3-(2-(2-Acetamidoethyl)-5-ethoxy-4-methoxyphenyl)-3-oxoprop–1-en-1-yl)­phenoxy)­methyl)-1-(7-hydroxy-2-oxo-2*H*-chromen-3-yl)-1*H*-1,2,3-triazol-4-yl)-*N*-(2-(2,6-dioxopiperidin-3-yl)-1,3-dioxoisoindolin-4-yl)­oct-7-ynamide

Following general procedure D, **TKP-37** was prepared in 73% yield as a yellow solid using **TKP-21** and 3-azido-7-hydroxycoumarin. *R*_f_ = 0.47 (8:1 CHCl_3_/MeOH); IR (neat) 3369, 3109, 2933, 2854, 2239, 1705, 1604, 1512, 1477, 1396, 1352, 1261, 1196, 1128, 1026, 995, 823, 750 cm^–1^; ^1^H NMR (600 MHz, CDCl_3_): δ 10.42 (br, 1H), 9.41 (s, 1H), 8.77 (d, *J* = 8.4 Hz, 1H), 8.49 (s, 1H), 7.86 (s, 1H), 7.66 (dd, *J* = 8.4, 7.4 Hz, 1H), 7.49 (d, *J* = 7.4 Hz, 1H), 7.39 (d, *J* = 15.8 Hz, 1H), 7.36 (d, *J* = 8.6 Hz, 1H), 7.34 (d, *J* = 8.8 Hz, 2H), 7.25 (br, 1H), 6.99 (s, 1H), 6.92 (d, *J* = 15.8 Hz, 1H), 6.90 (dd, *J* = 8.6, 2.2 Hz, 1H), 6.85 (d, *J* = 2.2 Hz, 1H), 6.77 (s, 1H), 6.72 (d, *J* = 8.8 Hz, 2H), 5.40 (s, 2H), 4.96 (m, 1H), 4.06 (q, *J* = 7.0 Hz, 2H), 3.89 (s, 3H), 3.48 (q, *J* = 6.3 Hz, 2H), 3.01–2.71 (m, 5H), 2.51 (t, *J* = 6.9 Hz, 2H), 2.46 (t, *J* = 7.4 Hz, 2H), 2.15 (m, 1H), 1.96 (s, 3H), 1.79 (quint, *J* = 6.9 Hz, 2H), 1.70 (quint, *J* = 7.4 Hz, 2H), 1.63–1.53 (m, 2H), 1.42 (t, *J* = 7.0 Hz, 3H); ^13^C NMR (151 MHz, CDCl_3_): δ 195.8 (C), 172.3 (C), 171.9 (C), 171.1 (C), 169.3 (C), 168.2 (C), 166.9 (C), 163.6 (C), 159.9 (C), 157.1 (C), 155.9 (C), 151.9 (C), 146.5 (C), 145.9 (CH), 140.6 (CH), 137.9 (C), 136.6 (CH), 136.1 (C), 132.5 (C), 131.8 (C), 131.7 (C), 131.2 (C), 130.5 (CH), 130.4 (CH), 128.4 (C), 125.4 (CH), 124.7 (CH), 119.5 (C), 118.6 (CH), 115.7 (CH), 115.5 (C), 115.3 (CH), 113.7 (CH), 113.6 (CH), 110.4 (C), 103.3 (CH), 97.1 (C), 69.1 (C), 65.0 (CH_2_), 59.8 (CH_2_), 56.2 (CH_3_), 49.4 (CH), 42.3 (CH_2_), 37.8 (CH_2_), 32.0 (CH_2_), 31.5 (CH_2_), 28.4 (CH_2_), 28.0 (CH_2_), 24.8 (CH_2_), 23.2 (CH_3_), 22.8 (CH_2_), 19.5 (CH_2_), 14.9 (CH_3_); HRMS (FAB) *m*/*z*: [M + H]^+^ calcd for C_55_H_52_N_7_O_13_, 1018.3618; found, 1018.3615.

##### **TKP-47**: (*E*)-6-(5′-((4-(3-(2-(2-Acetamidoethyl)-5-ethoxy-4-methoxyphenyl)-3-oxoprop–1-en-1-yl)­phenoxy)­methyl)-1,1′-dibenzyl-1*H*,1′*H*-[4,4′-bi­(1,2,3-triazol)]-5 yl)-*N*-(2-(2,6-dioxopiperidin-3-yl)-1,3-dioxoisoindolin-4-yl)­hexanamide

After freeze–pump–thaw cycling for the mixture of **TKP-36** (30.0 mg, 31.6 μmol) and benzyl azide (6.31 mg, 47.4 μmol) in THF (630 μL), it was added to a solution of Cp*Ru­(cod)Cl (3.59 mg, 9.47 μmol), and the mixture was stirred for 10 h. The reaction mixture was filtered through a Celite pad, and the filtrate was dried over anhydrous Na_2_SO_4_. Filtration and evaporation in vacuo furnished the crude product, which was purified by flash column chromatography (100:0 → 50:1 CHCl_3_/MeOH) to give **TKP-44** (24.0 mg, 22,1 μmol, 70%) as a yellow solid. *R*_f_ = 0.48 (8:1 CHCl_3_/MeOH); IR (neat) 3350, 3008, 2935, 2858, 1768, 1705, 1657, 1597, 1512, 1477, 1398, 1350, 1261, 1198, 1130, 1005, 825, 750 cm^–1^; ^1^H NMR (597 MHz, CDCl_3_): δ 9.36 (s, 1H), 8.80 (d, *J* = 8.4 Hz, 1H), 8.43 (s, 1H), 7.69 (dd, *J* = 8.4, 7.1 Hz, 1H), 7.53 (d, *J* = 7.1 Hz, 1H), 7.50 (d, *J* = 15.9 Hz, 1H), 7.46 (d, *J* = 8.8 Hz, 2H), 7.37–7.27 (m, 6H), 7.25 (s, 1H), 7.23–7.18 (m, 4H), 7.03 (s, 1H), 7.01 (d, *J* = 15.9 Hz, 1H), 6.95 (d, *J* = 8.8 Hz, 2H), 6.81 (s, 1H), 5.69 (s, 2H), 5.58 (s, 2H), 5.56 (s, 2H), 4.93 (m, 1H), 4.09 (q, *J* = 7.0 Hz, 2H), 3.92 (s, 3H), 3.52 (q, *J* = 5.5 Hz, 2H), 3.03 (t, *J* = 7.8 Hz, 2H), 2.94–2.67 (m, 5H), 2.35 (t, *J* = 7.6 Hz, 2H), 2.15 (m, 1H), 1.91 (s, 3H), 1.65 (quint, *J* = 7.6 Hz, 2H), 1.53–1.42 (m, 5H), 1.37 (quint, *J* = 7.6 Hz, 2H); ^13^C NMR (150 MHz, CDCl_3_): δ 195.5 (C), 172.2 (C), 170.9 (C), 170.7 (C), 169.2 (C), 168.0 (C), 166.8 (C), 159.9 (C), 151.9 (C), 146.2 (C), 146.0 (CH), 139.5 (C), 137.9 (C), 137.3 (C), 136.5 (CH), 136.0 (C), 135.1 (C), 134.4 (C), 133.3 (C), 131.4 (C), 131.2 (C), 130.5 (CH), 129.2 (CH), 129.2, 129.1 (CH), 128.7 (CH), 128.7 (CH), 128.1 (C), 127.8 (CH), 127.4 (CH), 125.4 (CH), 124.5 (CH), 118.5 (CH), 115.5 (CH), 115.4 (C), 113.7 (CH), 113.4 (CH), 65.0 (CH_2_), 58.4 (CH_2_), 56.1 (CH_3_), 53.2 (CH_2_), 52.2 (CH_2_), 49.4 (CH), 42.1 (CH_2_), 37.6 (CH_2_), 31.8 (CH_2_), 31.5 (CH_2_), 28.8 (CH_2_), 28.0 (CH_2_), 24.6 (CH_2_), 23.3 (CH_3_), 23.1 (CH_2_), 22.8 (CH_2_), 14.9 (CH_3_); HRMS (FAB) *m*/*z*: [M + H]^+^ calcd for C_60_H_61_N_10_O_10_, 1081.4567; found, 1081.4564. HPLC purity = 93%.

#### Synthesis of TKP Analogs with Different Triazole Positions

##### **TKP-31**: (*E*)-8-(4-(4-(3-(2-(2-Acetamidoethyl)-5-ethoxy-4-methoxyphenyl)-3-oxoprop-1-en-1-yl)­phenyl)-1*H*-1,2,3-triazol-1-yl)-*N*-(2-(2,6-dioxopiperidin-3-yl)-1,3-dioxoisoindolin-4-yl) Octanamide

Following general procedure C, **TKP-31** was prepared in 82% yield as a yellow solid using alkyne **39** and azide **30**. *R*_f_ = 0.53 (8:1 CHCl_3_/MeOH); IR (neat) 3357, 2931, 2856, 1768, 1705, 1657, 1618, 1523, 1477, 1398, 1350, 1263, 1198, 1130, 1041, 974, 823, 750, 665 cm^–1^; ^1^H NMR (597 MHz, CDCl_3_): δ 9.38 (s, 1H), 8.82 (s, 1H), 8.78 (d, *J* = 7.6 Hz, 1H), 7.86 (d, *J* = 8.3 Hz, 2H), 7.82 (s, 1H), 7.67 (t, *J* = 7.6 Hz, 1H), 7.61 (d, *J* = 8.3 Hz, 2H), 7.57 (d, *J* = 15.9 Hz, 1H), 7.50 (d, *J* = 7.6 Hz, 1H), 7.17 (br, 1H), 7.17 (d, *J* = 15.9 Hz, 1H), 7.06 (s, 1H), 6.81 (s, 1H), 4.94 (m, 1H), 4.38 (t, *J* = 7.3 Hz, 2H), 4.09 (q, *J* = 7.0 Hz, 2H), 3.90 (s, 3H), 3.51 (q, *J* = 6.0 Hz, 2H), 2.92–2.71 (m, 5H), 2.42 (t, *J* = 7.6 Hz, 2H), 2.14 (m, 1H), 1.94 (quint, *J* = 7.3 Hz, 2H), 1.91 (s, 3H), 1.72 (quint, *J* = 7.6 Hz, 2H), 1.44 (t, *J* = 7.0 Hz, 3H), 1.43–1.31 (m, 6H); ^13^C NMR (150 MHz, CDCl_3_): δ 195.2 (C), 172.3 (C), 171.1 (C), 170.6 (C), 169.3 (C), 168.2 (C), 166.8 (C), 152.0 (C), 146.9 (C), 146.2 (C), 145.7 (CH), 137.8 (C), 136.5 (CH), 134.1 (C), 133.5 (C), 133.2 (C), 131.17 (C), 131.18 (C), 129.2 (CH), 126.2 (CH), 126.1 (CH), 125.3 (CH), 120.2 (CH), 118.5 (CH), 115.4 (C), 113.8 (CH), 113.6 (CH), 65.0 (CH_2_), 56.1 (CH_3_), 50.5 (CH_2_), 49.4 (CH), 42.0 (CH_2_), 37.8 (CH_2_), 31.9 (CH_2_), 31.5 (CH_2_), 30.3 (CH_2_), 28.8 (CH_2_), 28.7 (CH_2_), 26.3 (CH_2_), 25.1 (CH_2_), 23.3 (CH_3_), 22.7 (CH_2_), 14.9 (CH_3_). HRMS (FAB) *m*/*z*: [M + H]^+^ calcd for C_45_H_50_N_7_O_9_, 832.3665; found, 832.3675.

##### (*E*)-*N*-(4-Ethoxy-2-(3-(4-hydroxyphenyl)­acryloyl)-5-methoxyphenethyl)­acetamide (**S1**)

PPTS (110 mg, 439 μmol) was added to a solution of ethyl vinyl ether **19** (2.00 g, 4.39 mmol) in EtOH (29 mL). After 3 h of stirring at 50 °C, the reaction was quenched with sat. NaHCO_3_ aqueous (20 mL), and the mixture was extracted with AcOEt (3 × 30 mL). The combined organic extracts were washed with brine (100 mL) and dried over anhydrous Na_2_SO_4_. Filtration and evaporation in vacuo furnished the crude product, which was purified by flash column chromatography (50:1 → 20:1, CHCl_3_/MeOH) to give phenol **S1** (1.65 g, 98%) as a yellow solid. IR (neat) 3278, 3101, 2981, 1649, 1593, 1576, 1512, 1442, 1356, 1261, 1200, 1167, 1132, 1047, 985, 831, 754 cm^–1^; ^1^H NMR (597 MHz, DMSO-*d*_6_): δ 10.09 (br, 1H), 7.88 (br, 1H), 7.63 (d, *J* = 6.3 Hz, 2H), 7.44 (d, *J* = 15.5 Hz, 1H), 7.18 (d, *J* = 15.5 Hz, 1H), 7.15 (s, 1H), 6.89 (s, 1H), 6.82 (d, *J* = 6.3 Hz, 2H), 4.16–3.97 (m, 2H), 3.83 (s, 3H), 3.32–3.12 (m, 2H), 2.94–2.71 (s, 2H), 1.75 (s, 3H), 1.42–1.20 (m, 3H); ^13^C NMR (150 MHz, DMSO-*d*_6_): δ 193.6 (C), 169.0 (C), 160.1 (C), 150.6 (C), 145.6 (C), 144.9 (CH), 132.3 (C), 131.3 (CH), 130.9 (C), 125.6 (C), 123.0 (CH), 115.9 (CH), 114.2 (CH), 113.4 (CH), 64.0 (CH_2_), 55.5 (CH_3_), 40.5 (CH_2_), 32.5 (CH_2_), 22.6 (CH_3_), 14.7 (CH_3_); HRMS (E I) *m*/*z*: [M]^+^ calcd for C_22_H_25_NO_5_, 383.4440; found, 383.1734.

##### (*E*)-*N*-(4-Ethoxy-5-methoxy-2-(3-(4-(pent-4-yn-1-yloxy)­phenyl)­acryloyl)­phenethyl)­acetamide (**S2**)

K_2_CO_3_ (72.1 mg, 522 μmol) and pent-4-yn-1-yl methanesulfonate (63.5 mg, 391 μmol) were added to a solution of phenol **S1** (100 mg, 261 μmol) in DMF (2.6 mL). After 12 h of stirring at 80 °C, the reaction was quenched with half-saturated aqueous NH_4_Cl (5 mL), and the mixture was extracted with AcOEt (3 × 10 mL). The combined organic extracts were washed with brine (3 × 30 mL) and dried over anhydrous Na_2_SO_4_. Filtration and evaporation in vacuo furnished the crude product, which was purified by flash column chromatography (50:1 → 20:1, CHCl_3_/MeOH) to give alkyne **S2** (94.2 mg, 80%) as a yellow solid. IR (neat) 3294, 2935, 2121, 1655, 1597, 1568, 1512, 1469, 1352, 1257, 1200, 1174, 1130, 1043, 985, 827, 746 cm^–1^; ^1^H NMR (597 MHz, CDCl_3_): δ 7.57–7.47 (m, 3H), 7.28 (br, 1H), 7.05–6.98 (m, 2H), 6.92 (d, *J* = 8.7 Hz, 2H), 6.80 (s, 1H), 4.18–4.01 (m, 4H), 3.91 (s, 3H), 3.51 (q, *J* = 6.0 Hz, 2H), 2.85 (t, *J* = 6.0 Hz, 2H), 2.40 (td, *J* = 6.9, 2.5 Hz, 2H), 2.04–1.98 (m, 2H), 1.97 (t, *J* = 2.5 Hz, 1H), 1.90 (s, 3H), 1.45 (t, *J* = 7.0 Hz, 3H); ^13^C NMR (150 MHz, CDCl_3_): δ 195.6 (C), 170.6 (C), 161.4 (C), 151.8 (C), 146.5 (CH), 146.2 (C), 133.2 (C), 131.6 (C), 130.5 (CH), 127.2 (C), 124.0 (CH), 115.1 (CH), 113.6 (CH), 113.4 (CH), 83.2 (CH), 69.2 (C), 66.4 (CH_2_), 64.9 (CH_2_), 56.1 (CH_3_), 42.1 (CH_2_), 31.6 (CH_2_), 28.1 (CH_2_), 23.3 (CH_3_), 15.2 (CH_2_), 14.9 (CH_3_); HRMS (EI) *m*/*z*: [M]^+^ calcd for C_27_H_31_NO_5_, 449.2202; found, 449.2196.

##### **TKP-32**: (*E*)-4-(4-(3-(4-(3-(2-(2-Acetamidoethyl)-5-ethoxy-4-methoxyphenyl)-3-oxoprop-1-en-1-yl)­phenoxy)­propyl)-1*H*-1,2,3-triazol-1-yl)-*N*-(2-(2,6-dioxopiperidin-3-yl)-1,3-dioxoisoindolin-4-yl)­butanamide

Following general procedure C, **TKP-32** was prepared in 89% yield as a yellow solid using alkyne **S2** and azide **28**. *R*_f_ = 0.53 (8:1 CHCl_3_/MeOH); IR (neat) 3342, 2937, 2359, 2335, 1765, 1705, 1655, 1597, 1512, 1477, 1398, 1350, 1259, 1198, 1130, 1038, 750 cm^–1^; ^1^H NMR (597 MHz, CDCl_3_): δ 9.36 (s, 1H), 8.83 (s, 1H), 8.72 (d, *J* = 8.2 Hz, 1H), 7.67 (t, *J* = 8.2 Hz, 1H), 7.56–7.44 (m, 4H), 7.36 (s, 1H), 7.02 (s, 1H), 7.00 (d, *J* = 15.9 Hz, 1H), 6.89 (d, *J* = 8.7 Hz, 2H), 6.80 (s, 1H), 4.94 (dd, *J* = 12.3, 5.3 Hz, 1H), 4.44 (t, *J* = 7.0 Hz, 2H), 4.08 (q, *J* = 7.0 Hz, 2H), 4.04 (t, *J* = 6.3 Hz, 2H), 3.90 (s, 3H), 3.50 (q, *J* = 5.7 Hz, 2H), 2.94–2.69 (m, 7H), 2.48 (t, *J* = 7.0 Hz, 2H), 2.30 (quint, *J* = 7.0 Hz, 2H), 2.17 (quint, *J* = 6.3 Hz, 2H), 2.13 (m, 1H), 1.90 (s, 3H), 1.44 (t, *J* = 7.0 Hz, 3H); ^13^C NMR (150 MHz, CDCl_3_): δ 195.6 (C), 171.1 (C), 170.75 (C), 170.65 (C), 169.1 (C), 168.2 (C), 166.7 (C), 161.4 (C), 151.8 (C), 147.3 (C), 146.4 (CH), 146.1 (C), 137.5 (C), 136.5 (CH), 133.2 (C), 131.5 (C), 131.2 (C), 130.5 (CH), 127.2 (C), 125.3 (CH), 124.0 (CH), 121.3 (CH), 118.8 (CH), 115.6 (C), 115.1 (CH), 113.7 (CH), 113.5 (CH), 67.2 (CH_2_), 64.9 (CH_2_), 56.1 (CH_3_), 49.4 (CH), 49.1 (CH_2_), 42.0 (CH_2_), 33.9 (CH_2_), 31.7 (CH_2_), 31.5 (CH_2_), 28.8 (CH_2_), 25.5 (CH_2_), 23.3 (CH_3_), 22.7 (CH_2_), 22.1 (CH_2_), 14.9 (CH_3_); HRMS (FAB) *m*/*z*: [M + H]^+^ calcd for C_44_H_48_N_7_O_10_, 834.3457; found, 834.3454.

##### 4-(2-((Tetrahydro-2*H*-pyran-2-yl)­oxy)­ethyl)­benzaldehyde (**S3**)

*n*-BuLi (2.10 mL, 3.36 mmol) was added to a solution of 2-(3-(4-bromophenyl)­propoxy) tetrahydro-2*H*-pyran^47^ (950 mg, 3.18 mmol) in THF (21 mL) at −78 °C for dropwise. After 30 min of stirring at −78 °C, DMF (740 μL, 9.56 mmol) was added to the reaction mixture for dropwise. After another 3 h of stirring at room temperature, the reaction was quenched with saturated aqueous NH_4_Cl (20 mL), and the mixture was extracted with AcOEt (3 × 30 mL). The combined organic extracts were washed with brine (100 mL) and dried over anhydrous Na_2_SO_4_. Filtration and evaporation in vacuo furnished the crude product, which was purified by flash column chromatography (4:1, *n*-hexane/AcOEt) to give aldehyde **S3** (645 mg, 2.60 mmol, 82%) as a colorless oil. IR (neat) 2943, 2870, 1699, 1606, 1576, 1419, 1389, 1275, 1213, 1173, 1119, 1034, 989, 852, 827 cm^–1^; ^1^H NMR (597 MHz, CDCl_3_): δ 9.97 (d, *J* = 2.5 Hz, 1H), 7.80 (dd, *J* = 2.5, 6.4 Hz, 2H), 7.36 (d, *J* = 6.4 Hz, 2H), 4.57 (s, 1H), 3.86 (m, 1H), 3.78 (m, 1H), 3.50 (m, 1H), 3.41 (m, 1H), 2.84–2.76 (m, 2H), 1.97–1.93 (m, 2H), 1.84 (m, 1H), 1.72 (m, 1H), 1.61–1.50 (m, 4H); ^13^C NMR (150 MHz, CDCl_3_): δ 192.2 (CH), 149.8 (CH), 134.7 (C), 130.1 (CH), 129.2 (C), 99.3 (CH), 66.7 (CH_2_), 62.6 (CH_2_), 32.9 (CH_2_), 31.1 (CH_2_), 30.9 (CH_2_), 25.6 (CH_2_), 19.8 (CH_2_); HRMS (EI) *m*/*z*: [M]^+^ calcd for C_15_H_20_O_3_, 248.1412; found, 248.1404.

##### (*E*)-*N*-(4-Ethoxy-2-(3-(4-(3-hydroxypropyl)­phenyl)­acryloyl)-5-methoxyphenethyl) Acetamide (**S4**)

10% aqueous NaOH (7.5 mL) was added to a solution of aldehyde **S3** (489 mg, 1.97 mmol) and methyl ketone **14** (500 mg, 1.79 mmol) in EtOH (7.5 mL) at 20 °C. After 1 h of stirring at room temperature, the reaction mixture was diluted with H_2_O (20 mL) at 0 °C, and the mixture was extracted with CHCl_3_ (2 × 20 mL). The combined organic extracts were dried over anhydrous Na_2_SO_4_. Filtration and concentrated in vacuo furnished the crude product (812 mg), which was used without further purification.

PPTS (30.3 mg, 159 μmol) was added to a solution of crude ether (812 mg) in MeOH (13 mL). After 1 h of stirring at rt, the reaction was quenched with saturated aqueous NaHCO_3_ (20 mL), and the mixture was extracted with AcOEt (3 × 30 mL). The combined organic extracts were washed with brine (100 mL) and dried over anhydrous Na_2_SO_4_. Filtration and evaporation in vacuo furnished the crude product, which was purified by flash column chromatography (50:1 → 20:1, CHCl_3_/MeOH) to give alcohol **S4** (560 mg, 83% in 2 steps) as a yellow solid. IR (neat) 3300, 2935, 1655, 1599, 1584, 1514, 1444, 1350, 1263, 1200, 1130, 1045, 985, 754 cm^–1^; ^1^H NMR (600 MHz, CDCl_3_): δ 7.55 (d, *J* = 15.9 Hz, 1H), 7.50 (d, *J* = 8.1 Hz, 2H), 7.25 (br, 1H), 7.25 (d, *J* = 8.1 Hz, 2H), 7.11 (d, *J* = 15.9 Hz, 1H), 7.04 (s, 1H), 6.81 (s, 1H), 4.09 (q, *J* = 7.0 Hz, 2H), 3.92 (s, 3H), 3.67 (t, *J* = 6.4 Hz, 2H), 3.55–3.49 (m, 2H), 2.86 (t, *J* = 6.4 Hz, 2H), 2.75 (t, *J* = 7.4 Hz, 2H), 1.91 (s, 3H), 1.92–1.88 (m, 3H), 1.45 (t, *J* = 7.0 Hz, 3H); ^13^C NMR (151 MHz, CDCl_3_): δ 195.6 (C), 170.7 (C), 152.0 (C), 146.5 (CH), 146.2 (C), 145.7 (C), 133.4 (C), 132.3 (C), 131.3 (C), 129.3 (CH), 128.8 (CH), 125.6 (CH), 113.8 (CH), 113.5 (CH), 65.0 (CH_2_), 62.0 (CH_2_), 56.1 (CH_3_), 42.1 (CH_2_), 34.0 (CH_2_), 32.2 (CH_2_), 31.7 (CH_2_), 23.3 (CH_3_), 14.9 (CH_3_); HRMS (EI) *m*/*z*: [M]^+^ calcd for C_25_H_31_NO_5_, 425.2202; found, 425.2207.

##### *N*-(4-Ethoxy-5-methoxy-2-(3-(4-(3-oxopropyl)­phenyl)­propanoyl)­phenethyl)­acetamide (**S5**)

5% Pd/C (10.0 mg) was added to a solution of enone **S4** (186 mg, 437 μmol) in MeOH/AcOEt (1:1, 4.4 mL), and the mixture was stirred under hydrogen for 2 h. The reaction mixture was filtered through a Celite pad, and the filtrate was dried over anhydrous Na_2_SO_4_. Filtration and evaporation in vacuo furnished the crude product (202 mg), which was used without further purification.

Dess–Martin periodinane (220 mg, 520 μmol) was added to a solution of crude alcohol (202 mg) in CH_2_Cl_2_ (4.7 mL). After 2 h of stirring at rt, the reaction mixture was diluted with CH_2_Cl_2_ (10 mL), and the reaction was quenched with saturated aqueous NaHCO_3_/10% aqueous Na_2_S_2_O_3_ (1:1, 10 mL). After another 30 min of stirring at room temperature, the mixture was extracted with CH_2_Cl_2_ (2 × 20 mL). The combined organic extracts were washed with brine (30 mL) and dried over anhydrous Na_2_SO_4_. Filtration and evaporation in vacuo furnished the crude product, which was purified by flash column chromatography (50:1 → 20:1, CHCl_3_/MeOH) to give aldehyde **S5** (190 mg, 100% in 2 steps) as a colorless solid. IR (neat) 3298, 2933, 1720, 1670, 1603, 1564, 1516, 1444, 1362, 1265, 1128, 1043 cm^–1^; ^1^H NMR (600 MHz, CDCl_3_): δ 9.81 (t, *J* = 1.3 Hz, 1H), 7.17–7.10 (m, 4H), 7.07 (s, 1H), 6.75 (s, 1H), 6.69 (br, 1H), 4.04 (q, *J* = 7.0 Hz, 2H), 3.90 (s, 3H), 3.48 (q, *J* = 6.7 Hz, 2H), 3.18 (t, *J* = 7.6 Hz, 2H), 3.00 (t, *J* = 7.6 Hz, 2H), 2.93 (t, *J* = 7.5 Hz, 2H), 2.88 (t, *J* = 6.7 Hz, 2H), 2.76 (td, *J* = 7.5, 1.3 Hz, 2H), 1.91 (s, 3H), 1.45 (t, *J* = 7.0 Hz, 3H); ^13^C NMR (151 MHz, CDCl_3_): δ 203.0 (C), 201.7 (CH), 170.5 (C), 152.5 (C), 146.4 (C), 139.1 (C), 138.4 (C), 134.3 (C), 130.2 (C), 128.8 (CH), 128.7 (CH), 114.3 (CH), 113.8 (CH), 65.1 (CH_2_), 56.1 (CH_3_), 45.4 (CH_2_), 43.1 (CH_2_), 42.0 (CH_2_), 32.6 (CH_2_), 30.4 (CH_2_), 27.8 (CH_2_), 23.4 (CH_3_), 14.9 (CH_3_); HRMS (EI) *m*/*z*: [M]^+^ calcd for C_25_H_31_NO_5_, 425.2202; found, 425.2215.

##### *N*-(2-(3-(4-(But-3-yn-1-yl)­phenyl)­propanoyl)-4-ethoxy-5-methoxyphenethyl)­acetamide (**S6**)

Ohira–Bestmann reagent (63.0 μL, 341 μmol) and K_2_CO_3_ (78.6 mg, 569 μmol) were added to a solution of aldehyde **S5** (121 mg, 284 μmol) in MeOH (5.0 mL) at room temperature. After 5 h of stirring at room temperature, the reaction was quenched with saturated aqueous NH_4_Cl (5 mL) at 0 °C, and the mixture was extracted with AcOEt (3 × 10 mL). The combined organic extracts were washed with brine (30 mL) and dried over anhydrous Na_2_SO_4_. Filtration and evaporation in vacuo furnished the crude product, which was purified by flash column chromatography (50:1 → 20:1, CHCl_3_/MeOH) to give alkyne **S6** (90.2 mg, 214 μmol, 75%) as a colorless solid. IR (neat) 3284, 2931, 2100, 1668, 1641, 1564, 1516, 1444, 1362, 1265, 1198, 1128, cm^–1^; ^1^H NMR (597 MHz, CDCl_3_): δ 7.15 (s, 4H), 7.07 (s, 1H), 6.74 (s, 1H), 6.72 (br, 1H), 4.03 (q, *J* = 6.9 Hz, 2H), 3.89 (s, 3H), 3.50–3.45 (m, 2H), 3.18 (t, *J* = 7.5 Hz, 2H), 3.00 (t, *J* = 7.5 Hz, 2H), 2.88 (t, *J* = 6.5 Hz, 2H), 2.81 (t, *J* = 7.4 Hz, 2H), 2.49–2.43 (m, 2H), 1.97 (m, 1H), 1.90 (s, 3H), 1.44 (t, *J* = 6.9 Hz, 3H); ^13^C NMR (150 MHz, CDCl_3_): δ 203.0 (C), 170.4 (C), 152.4 (C), 146.3 (C), 139.0 (C), 138.5 (C), 134.2 (C), 130.2 (C), 128.7 (CH), 128.6 (CH), 114.3 (CH), 113.7 (CH), 83.8 (CH), 69.1 (C), 65.0 (CH_2_), 56.1 (CH_3_), 43.1 (CH_2_), 41.9 (CH_2_), 34.4 (CH_2_), 32.6 (CH_2_), 30.4 (CH_2_), 23.3 (CH_3_), 20.6 (CH_2_), 14.9 (CH_3_); HRMS (EI) *m*/*z*: [M]^+^ calcd for C_26_H_31_NO_4_, 421.2253; found, 421.2258.

##### 6-(4-(4-(3-(2-(2-Acetamidoethyl)-5-ethoxy-4-methoxyphenyl)-3-oxopropyl)­phenethyl)-1*H*-1,2,3-triazol-1-yl)-*N*-(2-(2,6-dioxopiperidin-3-yl)-1,3-dioxoisoindolin-4-yl) Hexanamide (**S7**)

Following general procedure C, compound **S7** was prepared in 86% yield as a yellow solid using alkyne **S6** and azide **29**. *R*_f_ = 0.53 (8:1 CHCl_3_/MeOH); IR (neat) 3348, 2927, 2862, 1772, 1705, 1618, 1523, 1477, 1396, 1352, 1263, 1198, 1130, 1043, 816, 748 cm^–1^; ^1^H NMR (600 MHz, CDCl_3_): δ 9.38 (s, 1H), 8.77 (d, *J* = 7.9 Hz, 1H), 8.55 (s, 1H), 7.67 (t, *J* = 7.9 Hz, 1H), 7.52 (d, *J* = 7.9 Hz, 1H), 7.15 (s, 1H), 7.13–7.08 (m, 4H), 7.05 (s, 1H), 6.73 (s, 1H), 6.71 (br, 1H), 4.93 (dd, *J* = 12.1, 5.0 Hz, 1H), 4.29 (t, *J* = 6.9 Hz, 2H), 4.01 (q, *J* = 6.9 Hz, 2H), 3.87 (s, 3H), 3.48–3.43 (m, 2H), 3.17 (t, *J* = 7.4 Hz, 2H), 3.05–2.68 (m, 11H), 2.43 (t, *J* = 7.2 Hz, 2H), 2.15 (m, 1H), 1.95–1.88 (m, 2H), 1.88 (s, 3H), 1.79–1.72 (m, 2H), 1.42 (t, *J* = 6.9 Hz, 3H), 1.40–1.29 (m, 2H); ^13^C NMR (151 MHz, CDCl_3_): δ 203.1 (C), 171.8 (C), 170.9 (C), 170.5 (C), 169.3 (C), 168.0 (C), 166.7 (C), 152.4 (C), 147.4 (C), 146.3 (C), 139.3 (C), 138.7 (C), 137.8 (C), 136.5 (CH), 134.2 (C), 131.2 (C), 130.1 (C), 128.7 (CH), 128.5 (CH), 125.3 (CH), 120.9 (CH), 118.6 (CH), 115.5 (C), 114.3 (CH), 113.8 (CH), 65.0 (CH_2_), 56.1 (CH_3_), 49.9 (CH_2_), 49.4 (CH), 43.1 (CH_2_), 41.8 (CH_2_), 37.5 (CH_2_), 35.3 (CH_2_), 32.6 (CH_2_), 31.5 (CH_2_), 30.4 (CH_2_), 30.1 (CH_2_), 27.6 (CH_2_), 26.0 (CH_2_), 24.5 (CH_2_), 23.3 (CH_3_), 22.8 (CH_2_), 14.9 (CH_3_); HRMS (FAB) *m*/*z*: [M + H]^+^ calcd for C_45_H_52_N_7_O_9_, 834.3821; found, 834.3823.

##### **TKP-33**: (*E*)-6-(4-(4-(3-(2-(2-Acetamidoethyl)-5-ethoxy-4-methoxyphenyl)-3-oxoprop-1-en-1-yl)­phenethyl)-1*H*-1,2,3-triazol-1-yl)-*N*-(2-(2,6-dioxopiperidin-3-yl)-1,3-dioxoisoindolin-4-yl)­hexanamide

Pd­(TFA)_2_ (68.6 mg, 206 μmol) and 4,5-diazafluoren-9-one (**47**) (37.6 mg, 206 μmol) were added to a solution of ketone **S7** (141 mg, 172 μmol) in DMSO (3.4 mL), and the reaction mixture was purged with O_2_ for 3 times. After 24 h of stirring at 100 °C under O_2_, the reaction was quenched with saturated aqueous NaHCO_3_ (5 mL) at 20 °C, and the mixture was extracted with CHCl_3_ (3 × 10 mL). The combined organic extracts were washed with brine (30 mL) and dried over anhydrous Na_2_SO_4_. Filtration and evaporation in vacuo furnished the crude product, which was purified by flash column chromatography (50:1 → 20:1, CHCl_3_/MeOH) to give **TKP-33** (21.0 mg, 25.7 μmol, 15%) as a yellow solid. *R*_f_ = 0.53 (8:1 CHCl_3_/MeOH); IR (neat) 3354, 2929, 2858, 2366, 2339, 1768, 1705, 1664, 1618, 1522, 1477, 1398, 1352, 1263, 1198, 1126, 1049, 750 cm^–1^; ^1^H NMR (597 MHz, CDCl_3_): δ 9.39 (s, 1H), 8.78 (d, *J* = 8.4 Hz, 1H), 8.42 (s, 1H), 7.70 (t, *J* = 8.4 Hz, 1H), 7.55 (d, *J* = 15.9 Hz, 1H), 7.54 (d, *J* = 8.4 Hz, 1H), 7.50 (d, *J* = 8.3 Hz, 2H), 7.22 (d, *J* = 8.3 Hz, 2H), 7.16 (s, 1H), 7.15 (m, 1H), 7.13 (d, *J* = 15.9 Hz, 1H), 7.05 (s, 1H), 6.81 (s, 1H), 4.94 (m, 1H), 4.31 (t, *J* = 7.1 Hz, 2H), 4.09 (q, *J* = 7.0 Hz, 2H), 3.92 (s, 3H), 3.52 (q, *J* = 5.7 Hz, 2H), 3.02–2.72 (m, 7H), 2.44 (t, *J* = 7.3 Hz, 2H), 2.17 (m, 1H), 1.98–1.88 (m, 2H), 1.91 (s, 3H), 1.83–1.73 (m, 4H), 1.45 (t, *J* = 7.0 Hz, 3H), 1.42–1.36 (m, 2H); ^13^C NMR (150 MHz, CDCl_3_): δ 195.5 (C), 171.8 (C), 170.8 (C), 170.7 (C), 169.3 (C), 168.0 (C), 166.8 (C), 152.0 (C), 146.9 (CH), 146.2 (C), 144.8 (C), 137.8 (C), 136.6 (CH), 133.5 (C), 132.5 (C), 131.9 (C), 131.2 (C), 129.4 (CH), 128.8 (CH), 125.3 (CH), 125.3 (CH), 120.5 (CH), 118.7 (CH), 115.5 (C), 114.0 (C), 113.8 (CH), 113.4 (CH), 65.0 (CH_2_), 56.2 (CH_3_), 49.9 (CH), 49.2 (CH_2_), 42.1 (CH_2_), 37.6 (CH_2_), 35.6 (CH_2_), 31.8 (CH_2_), 30.1 (CH_2_), 29.8 (CH_2_), 27.3 (CH_2_), 26.0 (CH_2_), 24.5 (CH_2_), 23.3­(CH_3_), 22.8 (CH_2_), 14.9­(CH_3_); HRMS (FAB) *m*/*z*: [M + H]^+^ calcd for C_45_H_50_N_7_O_9_, 832.3665; found, 832.3675.

## Supplementary Material







## Notes

Supplementary tables, schemes, and figures and copies of the NMR spectra (PDF)
Molecular strings formula (PDB)
Ternary complex modeling of (BRD4-BD1)-(TKP-5)-(DDB1B-CRBN)_Figure S5 (CSV)

## Abbreviations Used:

BET: bromodomain and extra-terminal domain
BRD4: bromodomain-containing protein 4
CuAAC: copper-catalyzed azide–alkyne cycloaddition
CRBN: cereblon
DMSO: dimethyl sulfoxide
E3: E3 ubiquitin ligase
FBS: fetal bovine serum
HRP: horseradish peroxidase
*K*_d_: dissociation constant
MD: molecular dynamics
MEM: minimum essential medium
PBS: phosphate-buffered saline
PCR: polymerase chain reaction
PEG: polyethylene glycol
POI: protein of interest
PROTACs: proteolysis-targeting chimeras
qPCR: quantitative polymerase chain reaction
RMSD: root-mean-square deviation
RuAAC: Ruthenium-catalyzed azide–alkyne cycloaddition
SDS–PAGE: sodium dodecyl-sulfate–polyacrylamide gel electrophoresis
TEWL: *trans*-epidermal water loss
TSLP: thymic stromal lymphopoietin

## References

[1] Lee S., Kim J., Jo J., Chang J. W., Sim J., Yun H. (2021). Recent Advances
in Development of Hetero-Bivalent Kinase Inhibitors. Eur. J. Med. Chem..

[2] Sakamoto K. M., Kim K. B., Kumagai A., Mercurio F., Crews C. M., Deshaies R. J. (2001). Protacs: Chimeric Molecules That Target Proteins to
the Skp1-Cullin-F Box Complex for Ubiquitination and Degradation. Proc. Natl. Acad. Sci. U. S. A..

[3] Toure M., Crews C. M. (2016). Small-Molecule PROTACS: New Approaches
to Protein Degradation. Angew. Chem. Int. Ed..

[4] Lai A. C., Crews C. M. (2017). Induced Protein
Degradation: An Emerging Drug Discovery
Paradigm. Nat. Rev. Drug Discovery.

[5] Békés M., Langley D. R., Crews C. M. (2022). PROTAC
Targeted Protein Degraders:
The Past Is Prologue. Nat. Rev. Drug Discovery.

[6] Zhong G., Chang X., Xie W., Zhou X. (2024). Targeted Protein Degradation:
Advances in Drug Discovery and Clinical Practice. Signal Transduct. Targeted Ther..

[7] Su Z., Xiao D., Xie F., Liu L., Wang Y., Fan S., Zhou X., Li S. (2021). Antibody-Drug Conjugates: Recent
Advances in Linker Chemistry. Acta Pharm. Sin.
B.

[8] Bashore F. M., Foley C. A., Ong H. W., Rectenwald J. M., Hanley R. P., Norris-Drouin J. L., Cholensky S. H., Mills C. A., Pearce K. H., Herring L. E., Kireev D., Frye S. V., James L. I. (2023). PROTAC Linkerology
Leads to an Optimized
Bivalent Chemical Degrader of Polycomb Repressive Complex 2 (PRC2)
Components. ACS Chem. Biol..

[9] Hendrick C. E., Jorgensen J. R., Chaudhry C., Strambeanu I. I., Brazeau J.-F., Schiffer J., Shi Z., Venable J. D., Wolkenberg S. E. (2022). Direct-to-Biology Accelerates PROTAC
Synthesis and
the Evaluation of Linker Effects on Permeability and Degradation. ACS Med. Chem. Lett..

[10] Liu J., Deng Y., Yin J., Ji J., Guan C., Chen X., Wu X., Zhu T., Liu S. (2024). A One-Pot
Photocatalytic Triazole-Based Linkerology for PROTACs. Cell Rep. Phys. Sci..

[11] Zografou-Barredo N. A., Hallatt A. J., Goujon-Ricci J., Cano C. (2023). A Beginner’s
Guide to Current Synthetic Linker Strategies towards VHL-Recruiting
PROTACs. Bioorg. Med. Chem..

[12] Pasieka A., Diamanti E., Uliassi E., Laura Bolognesi M. (2023). Click Chemistry
and Targeted Degradation: A Winning Combination for Medicinal Chemists?. ChemMedChem.

[13] Pravin N., Jóźwiak K. (2024). PROTAC Unleashed: Unveiling the Synthetic
Approaches and Potential Therapeutic Applications. Eur. J. Med. Chem..

[14] Sosič I., Bricelj A., Steinebach C. (2022). E3 Ligase Ligand Chemistries: From
Building Blocks to Protein Degraders. Chem.
Soc. Rev..

[15] Stevens R., Thompson J. D. F., Fournier J. C. L., Burley G. A., Battersby D. J., Miah A. H. I. (2024). Combinatorial
and High-Throughput Approaches to Degrader
Synthesis. Chem. Soc. Rev..

[16] Plesniak M. P., Taylor E. K., Eisele F., Kourra C. M. B. K., Michaelides I. N., Oram A., Wernevik J., Valencia Z. S., Rowbottom H., Mann N., Fredlund L., Pivnytska V., Novén A., Pirmoradian M., Lundbäck T., Storer R. I., Pettersson M., De Donatis G. M., Rehnström M. (2023). Rapid PROTAC Discovery Platform: Nanomole-Scale Array
Synthesis and Direct Screening of Reaction Mixtures. ACS Med. Chem. Lett..

[17] Wurz R. P., Dellamaggiore K., Dou H., Javier N., Lo M.-C., McCarter J. D., Mohl D., Sastri C., Lipford J. R., Cee V. J. A. (2018). “Click
Chemistry Platform” for the Rapid
Synthesis of Bispecific Molecules for Inducing Protein Degradation. J. Med. Chem..

[18] Brownsey D. K., Rowley B. C., Gorobets E., Gelfand B. S., Derksen D. J. (2021). Rapid Synthesis
of Pomalidomide-Conjugates for the Development of Protein Degrader
Libraries. Chem. Sci..

[19] Bhela I. P., Ranza A., Balestrero F. C., Serafini M., Aprile S., Di Martino R. M. C., Condorelli F., Pirali T. (2022). A Versatile and Sustainable
Multicomponent Platform for the Synthesis of Protein Degraders: Proof-of-Concept
Application to BRD4-Degrading PROTACs. J. Med.
Chem..

[20] Xu H., Kurohara T., Takano R., Yokoo H., Shibata N., Ohoka N., Inoue T., Naito M., Demizu Y. (2022). Development
of Rapid and Facile Solid-Phase Synthesis of PROTACs via a Variety
of Binding Styles. ChemistryOpen.

[21] Shi W., Luo Y., Luo X., Chao L., Zhang H., Wang J., Lei A. (2008). Investigation of an Efficient Palladium-Catalyzed
C­(Sp)–C­(Sp)
Cross-Coupling Reaction Using Phosphine–Olefin Ligand: Application
and Mechanistic Aspects. J. Am. Chem. Soc..

[22] Sindhu K. S., Thankachan A. P., Sajitha P. S., Anilkumar G. (2015). Recent Developments
and Applications of the Cadiot-Chodkiewicz Reaction. Org. Biomol. Chem..

[23] Segawa R., Takeda H., Yokoyama T., Ishida M., Miyata C., Saito T., Ishihara R., Nakagita T., Sasano Y., Kanoh N., Iwabuchi Y., Mizuguchi M., Hiratsuka M., Hirasawa N. (2021). A Chalcone Derivative Suppresses
TSLP Induction in Mice and Human Keratinocytes through Binding to
BET Family Proteins. Biochem. Pharmacol..

[24] Sarnik J., Popławski T., Tokarz P. (2021). BET Proteins as Attractive
Targets
for Cancer Therapeutics. Int. J. Mol. Sci..

[25] Tian B., Hosoki K., Liu Z., Yang J., Zhao Y., Sun H., Zhou J., Rytting E., Kaphalia L., Calhoun W. J., Sur S., Brasier A. R. (2019). Mucosal
Bromodomain-Containing Protein 4 Mediates Aeroallergen-Induced
Inflammation and Remodeling. J. Allergy Clin.
Immunol..

[26] Xiao X., Fan Y., Li J., Zhang X., Lou X., Dou Y., Shi X., Lan P., Xiao Y., Minze L., Li X. C. (2018). Guidance
of Super-Enhancers in Regulation of IL-9 Induction and Airway Inflammation. J. Exp. Med..

[27] Nadeem A., Al-Harbi N. O., Al-Harbi M. M., El-Sherbeeny A. M., Ahmad S. F., Siddiqui N., Ansari M. A., Zoheir K. M. A., Attia S. M., Al-Hosaini K. A., Al-Sharary S. D. (2015). Imiquimod-Induced
Psoriasis-like Skin Inflammation Is Suppressed by BET Bromodomain
Inhibitor in Mice through RORC/IL-17A Pathway Modulation. Pharmacol. Res..

[28] Tang J., Zhao X. (2012). Synthesis of 2,5-Disubstituted
Thiophenes via Metal-Free Sulfur Heterocyclization
of 1,3-Diynes with Sodium Hydrosulfide. RSC
Adv..

[29] Nun P., Dupuy S., Gaillard S., Poater A., Cavallo L., Nolan S. P. (2011). Gold­(I)-Catalyzed
Synthesis of Furans and Pyrroles
via Alkyne Hydration. Catal. Sci. Technol..

[30] Zheng Q., Hua R. (2010). CuCl-Catalyzed Cycloaddition
of 1,3-Butadiynes with Primary Amines:
An Atom-Economic Process for Synthesis of 1,2,5-Trisubsituted Pyrroles. Tetrahedron Lett..

[31] Ghosh A., Sapkal G. T., Pawar A. B. (2023). Ru­(II)-Catalyzed
Regioselective Redox-Neutral
[4 + 2] Annulation of N-Chlorobenzamides with 1,3-Diynes at Room Temperature
for the Synthesis of Isoquinolones. J. Org.
Chem..

[32] Hyster T. K., Rovis T. (2010). Rhodium-Catalyzed Oxidative Cycloaddition of Benzamides and Alkynes
via C-H/N-H Activation. J. Am. Chem. Soc..

[33] Yu D.-G., de Azambuja F., Gensch T., Daniliuc C. G., Glorius F. (2014). The C-H Activation/1,3-Diyne
Strategy: Highly Selective Direct Synthesis of Diverse Bisheterocycles
by Rh­(III) Catalysis. Angew. Chem. Int. Ed..

[34] Diao T., Wadzinski T. J., Stahl S. S. (2012). Direct Aerobic α, β-Dehydrogenation
of Aldehydes and Ketones with a Pd­(TFA)(2)/4,5-Diazafluorenone Catalyst­(). Chem. Sci..

[35] Gevorgyan V., Takeda A., Homma M., Sadayori N., Radhakrishnan U., Yamamoto Y. (1999). Palladium-Catalyzed [4 + 2] Cross-Benzannulation Reaction
of Conjugated Enynes with Diynes and Triynes. J. Am. Chem. Soc..

[36] Oger C., Balas L., Durand T., Galano J.-M. (2013). Are Alkyne Reductions
Chemo-, Regio-, and Stereoselective Enough to Provide Pure (Z)-Olefins
in Polyfunctionalized Bioactive Molecules?. Chem. Rev..

[37] Marion N., Ramón R. S., Nolan S. P. (2009). [(NHC)­Au­(I)]-Catalyzed Acid-Free
Alkyne Hydration at Part-per-Million Catalyst Loadings. J. Am. Chem. Soc..

[38] Zhang L., Chen X., Xue P., Sun H. H. Y., Williams I. D., Sharpless K. B., Fokin V. V., Jia G. (2005). Ruthenium-Catalyzed
Cycloaddition of Alkynes and Organic Azides. J. Am. Chem. Soc..

[39] Johansson J. R., Beke-Somfai T., Said Stålsmeden A., Kann N. (2016). Ruthenium-Catalyzed
Azide Alkyne Cycloaddition Reaction: Scope, Mechanism, and Applications. Chem. Rev..

[40] Knutson P. C., Fredericks H. E., Ferreira E. M. (2018). Synthesis of 1,3-Diynes
via Cadiot-Chodkiewicz
Coupling of Volatile, in Situ Generated Bromoalkynes. Org. Lett..

[41] Nowak R. P., DeAngelo S. L., Buckley D., He Z., Donovan K. A., An J., Safaee N., Jedrychowski M. P., Ponthier C. M., Ishoey M., Zhang T., Mancias J. D., Gray N. S., Bradner J. E., Fischer E. S. (2018). Plasticity in Binding
Confers Selectivity in Ligand-Induced
Protein Degradation. Nat. Chem. Biol..

[42] Wu Q., Heidenreich D., Zhou S., Ackloo S., Krämer A., Nakka K., Lima-Fernandes E., Deblois G., Duan S., Vellanki R. N., Li F., Vedadi M., Dilworth J., Lupien M., Brennan P. E., Arrowsmith C. H., Müller S., Fedorov O., Filippakopoulos P., Knapp S. (2019). A Chemical Toolbox for the Study of Bromodomains and Epigenetic Signaling. Nat. Commun..

[43] Gilan O., Rioja I., Knezevic K., Bell M. J., Yeung M. M., Harker N. R., Lam E. Y. N., Chung C.-W., Bamborough P., Petretich M., Urh M., Atkinson S. J., Bassil A. K., Roberts E. J., Vassiliadis D., Burr M. L., Preston A. G. S., Wellaway C., Werner T., Gray J. R., Michon A.-M., Gobbetti T., Kumar V., Soden P. E., Haynes A., Vappiani J., Tough D. F., Taylor S., Dawson S.-J., Bantscheff M., Lindon M., Drewes G., Demont E. H., Daniels D. L., Grandi P., Prinjha R. K., Dawson M. A. (2020). Selective
Targeting of BD1 and BD2 of the BET Proteins in Cancer and Immunoinflammation. Science.

[44] Segawa R., Yamashita S., Mizuno N., Shiraki M., Hatayama T., Satou N., Hiratsuka M., Hide M., Hirasawa N. (2014). Identification
of a Cell Line Producing High Levels of TSLP: Advantages for Screening
of Anti-Allergic Drugs. J. Immunol. Methods.

[45] Friesner R. A., Banks J. L., Murphy R. B., Halgren T. A., Klicic J. J., Mainz D. T., Repasky M. P., Knoll E. H., Shelley M., Perry J. K., Shaw D. E., Francis P., Shenkin P. S. (2004). Glide:
A New Approach for Rapid, Accurate Docking and Scoring. 1. Method
and Assessment of Docking Accuracy. J. Med.
Chem..

[46] Fuse S., Sugiyama H., Kobayashi D., Iijima Y., Matsumura K., Tanaka H., Takahashi T. R. (2015). One-pot,
Three-component Synthesis
of 1,3,4- and 1,3,5-triarylpyrazoles from 1- and 2-aryl-1-alkenyl
Sulfones: Synthesis of 1,3,4- and 1,3,5-Triarylpyrazoles. Eur. J. Org. Chem..

[47] Höhne A., Yu L., Mu L., Reiher M., Voigtmann U., Klar U., Graham K., Schubiger P. A., Ametamey S. M. (2009). Organofluorosilanes as Model Compounds for 18F-Labeled
Silicon-Based PET Tracers and Their Hydrolytic Stability: Experimental
Data and Theoretical Calculations (PET = Positron Emission Tomography). Chemistry.

