# Supplementary material for: Alkyne Two-Phase Strategy: Rapid Generation of TK-285-Derived PROTACs as BRD4 Degraders
Source: J Med Chem. 2026 Mar 19;69(7):8388–416. doi: 10.1021/acs.jmedchem.5c03771 (PMC13071873; doi:10.1021/acs.jmedchem.5c03771)
Supplement: Supplementary file 1 [file jm5c03771_si_001.pdf]

An Alkyne Two-Phase Strategy: Rapid Generation of TK-285-Derived PROTACs as BRD4 Degraders

Hiroyuki Yamakoshi[1*], Ryo Watanabe[1], Ryosuke Segawa[1,2], Ryosuke Ishihara[1], Ryo Tachibana[1], Genki Kudo[3], Shota Nagasawa[1], Satoshi Yamanaka[4], Ayano Ito[1], Hiroyuki Takeda[5], Tatsuya Sawasaki[6], Ryunosuke Yoshino[7,8], Takatsugu Hirokawa[7,8], Takayuki Doi[1], Noriyasu Hirasawa[1], and Yoshiharu Iwabuchi[1*]

[1] *Graduate School of Pharmaceutical Sciences, Tohoku University, 6-3 Aoba, Aramaki, Aoba-ku, Sendai 980-8578, Japan*

[2] *Department of Pharmaceutical Sciences, Tohoku University Hospital, Sendai 980-8574, Japan.*

[3] *Graduate School of Pure and Applied Sciences, University of Tsukuba, 1-1-1 Tennodai, Tsukuba, Ibaraki 305-8577, Japan.*

[4] *Division of Proteo-Interactome, Proteo-Science Center, Ehime University, 3 Bunkyo-cho, Matsuyama, Ehime 790-8577, Japan.*

[5] *Division of Proteo-Drug-Discovery Sciences, Proteo-Science Center, Ehime University, 3 Bunkyo-cho, Matsuyama, Ehime 790-8577, Japan.*

[6] *Division of Cell-Free Sciences, Proteo-Science Center, Ehime University, 3 Bunkyo-cho, Matsuyama, Ehime 790-8577, Japan.*

[7] *Institute of Medicine, University of Tsukuba, 2 Amakubo, Tsukuba, Ibaraki 305-0005, Japan.*

[8] *Transborder Medical Research Center, University of Tsukuba, 1-1-1 Tennodai, Tsukuba, Ibaraki 305-8577, Japan.*

[*] *hiroyuki.yamakoshi.e1@tohoku.ac.jp* (HY) and y-iwabuchi@tohoku.ac.jp (YI)

Table of Contents

1. Supplementary Tables and Figures S3–S17
2. Copies of ^{1}H and ^{13}C NMR Spectra S18–S85

1. Supplementary Tables and Figures

Table S1. BRD4 degradation after treatment with TKP compounds.

TKP compounds	BRD4 levels	TKP compounds	BRD4 levels
TKP-1	144.3 ± 22.1%	TKP-10	125.3 ± 35.3%
TKP-3	63.7 ± 4.4%	TKP-11	118.8 ± 18.8%
TKP-4	104.9 ±7.4%	TKP-13	86.5 ± 25.3%
TKP-5	5.2 ± 1.3%	TKP-14	33.4 ± 13.2%
TKP-6	53.7 ± 9.7%	TKP-16	19.1 ± 2.5%
TKP-7	93.3 ± 17.9%	TKP-17	46.0 ± 4.1%
TKP-8	98.1 ± 13.1%	TKP-18	69.4 ± 11.1%
TKP-9	67.2 ± 8.9%	TKP-20	117.9 ± 12.8%

Western blot analysis of BRD4 degradation induced by 10 μM TKP compounds in the mouse keratinocyte cell line KCMH-1 after 24 h treatment. BRD4 levels are reported as percentages of BRD4 band densitometry relative to the DMSO vehicle. Western blot results are representative of three (n = 3) independent experiments. BRD4, Bromodomain-containing protein 4; DMSO, Dimethyl sulfoxide.

Table S2. BRD4 degradation after treatment with TKP compounds.

TKP compounds	BRD4 levels	TKP compounds	BRD4 levels
TKP-5 (1 μM)	7.9 ± 2.6%	TKP-30 (1 μM)	77 ± 3.8%
TKP-5 (0.1 μM)	45 ± 6.5%	TKP-34 (1 μM)	43 ± 3.6%
TKP-21 (1 μM)	40 ± 5.0%	TKP-35 (1 μM)	38 ± 7.6%
TKP-22 (1 μM)	7.0 ± 3.4%	TKP-36 (1 μM)	4.1 ± 0.78%
TKP-22 (0.1 μM)	57 ± 9.6%	TKP-36 (0.1 μM)	88 ± 2.2%
TKP-23 (1 μM)	76 ± 5.7%	TKP-37 (1 μM)	29 ± 2.9%
TKP-24 (1 μM)	54 ± 6.3%	TKP-43 (1 μM)	2.9 ± 0.90%
TKP-25 (1 μM)	52 ± 4.8%	TKP-43 (0.1 μM)	71 ± 5.7%
TKP-26 (1 μM)	70 ± 2.7%	TKP-44 (1 μM)	70 ± 4.8%
TKP-27 (1 μM)	13 ± 3.5%	TKP-45 (1 μM)	26 ± 2.7%
TKP-27 (0.1 μM)	88 ± 1.9%	TKP-46 (1 μM)	36 ± 6.3%
TKP-28 (1 μM)	38 ± 4.9%	TKP-47 (1 μM)	0.91 ± 0.20%
TKP-29 (1 μM)	23 ± 5.1%	TKP-47 (0.1 μM)	62 ± 10%
TKP-29 (0.1 μM)	102 ± 6.2%		

Western blot analysis of BRD4 degradation induced by 1 or 0.1 μM TKP compounds in the mouse keratinocyte cell line KCMH-1 after 24 h treatment. BRD4 levels are reported as percentages of BRD4 band densitometry relative to the DMSO vehicle. Western blot results are representative of three (n = 3) independent experiments, except for **TKP-5** (n = 6). BRD4, Bromodomain-containing protein 4; DMSO, Dimethyl sulfoxide.

Table S3. BRD selectivity of **TKP-5**.

BD, gene symbol	Percentage control @1000 nM	BD, gene symbol	Percentage control @1000 nM	BD, gene symbol	Percentage control @1000 nM
ATAD2A	84	BRD7	100	PBRM1(2)	95
ATAD2B	95	BRD9	91	PBRM1(5)	100
BAZ2A	88	**BRDT(1)**	**16**	PCAF	95
BAZ2B	79	BRDT(2)	81	SMARCA2	49
BRD1	74	BRPF1	100	SMARCA4	78
**BRD2(1)**	**0.95**	BRPF3	100	TAF1(2)	58
BRD2(2)	79	CECR2	89	TAF1L(2)	78
**BRD3(1)**	**0.8**	CREBBP	87	TRIM24(PHD,Bromo.)	74
BRD3(2)	61	EP300	93	TRIM33(PHD,Bromo.)	93
**BRD4(1)**	**9.4**	FALZ	57	WDR9(2)	100
BRD4(2)	66	GCN5L2	73		

BROMOscan™ profiling (Eurofins Discovery, Fremont, CA, USA) was employed at a concentration of 1000 nM.

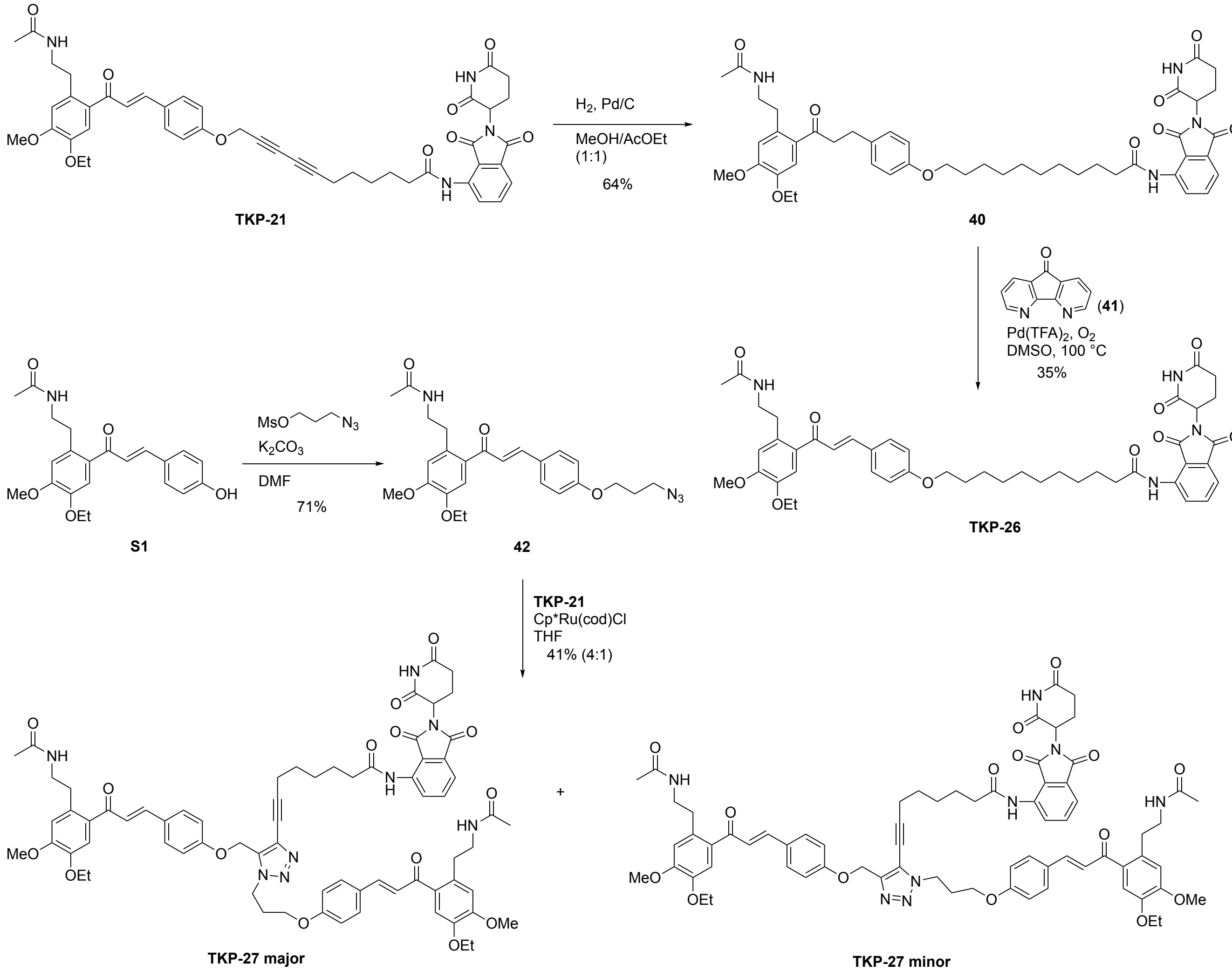

Scheme S1. Synthesis of **TKP-26** and **TKP-27**.

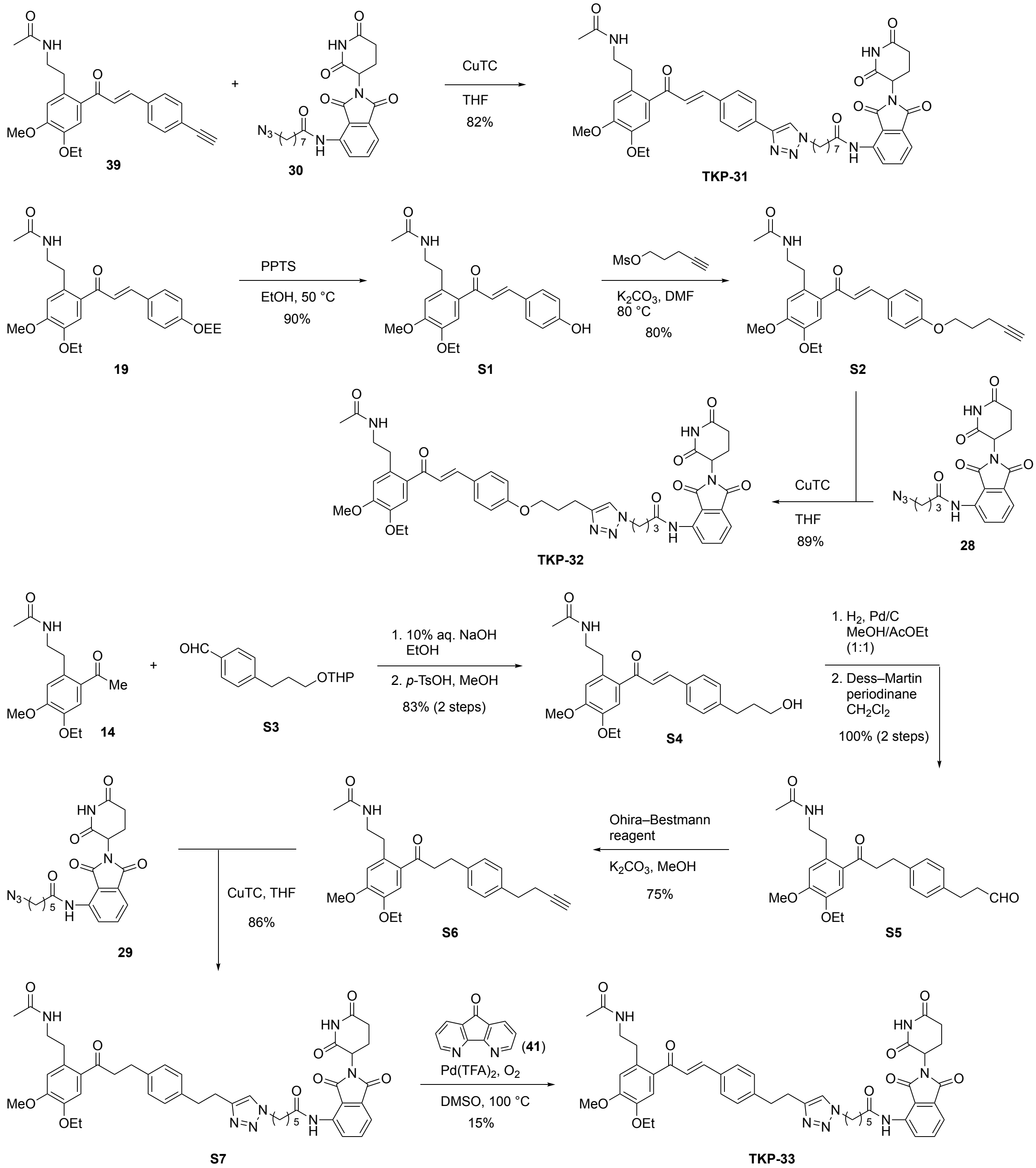

Scheme S2. Synthesis of **TKP-5** derivatives with shifted triazole ring positions

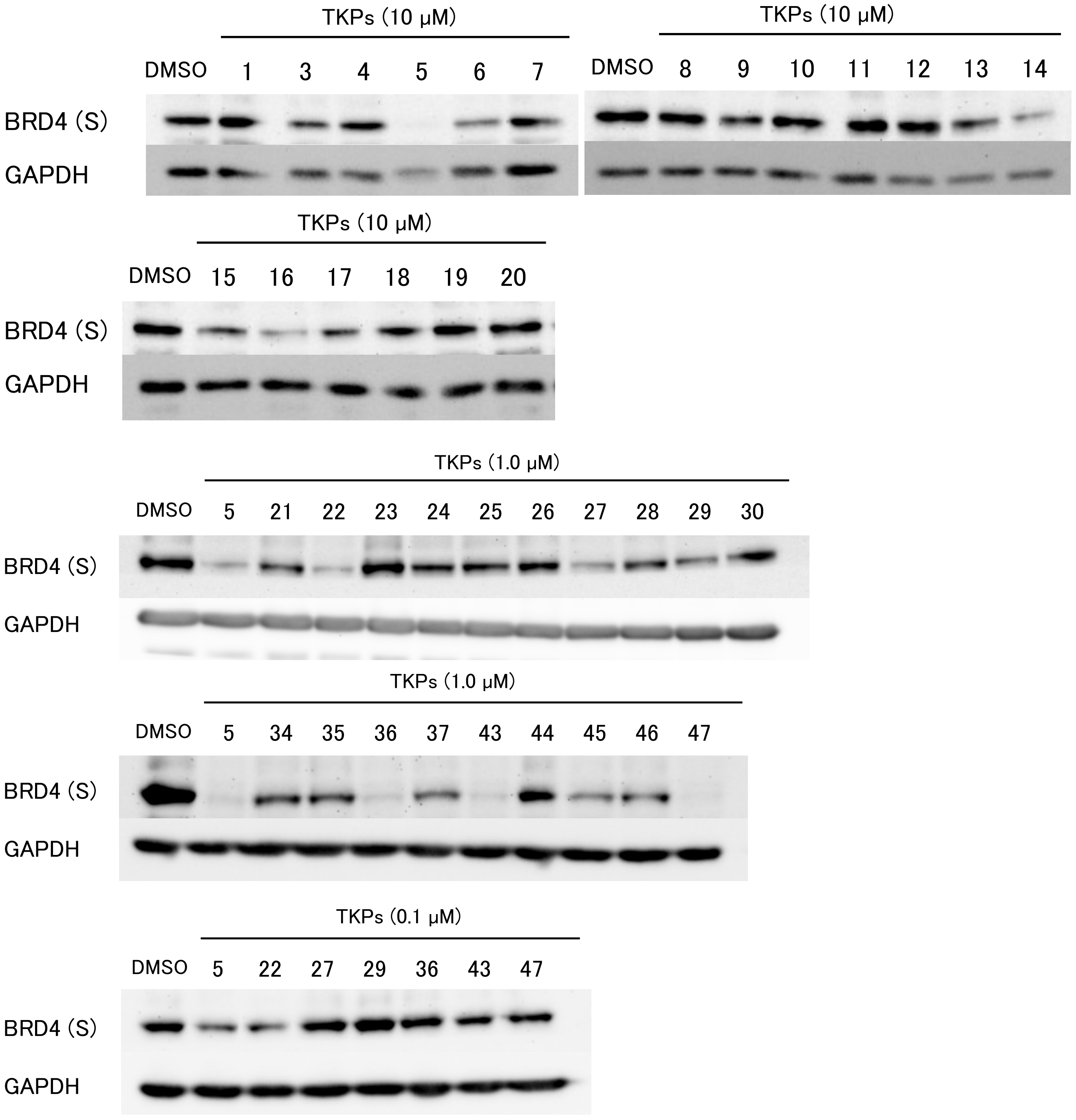

Figure S1. Western blot analysis of BRD4 degradation in KCMH-1 cells after 24-hour treatment with the compounds. Representative results are shown. BRD4, Bromodomain-containing protein 4; DMSO, Dimethyl sulfoxide.

(a) **TKP-5**

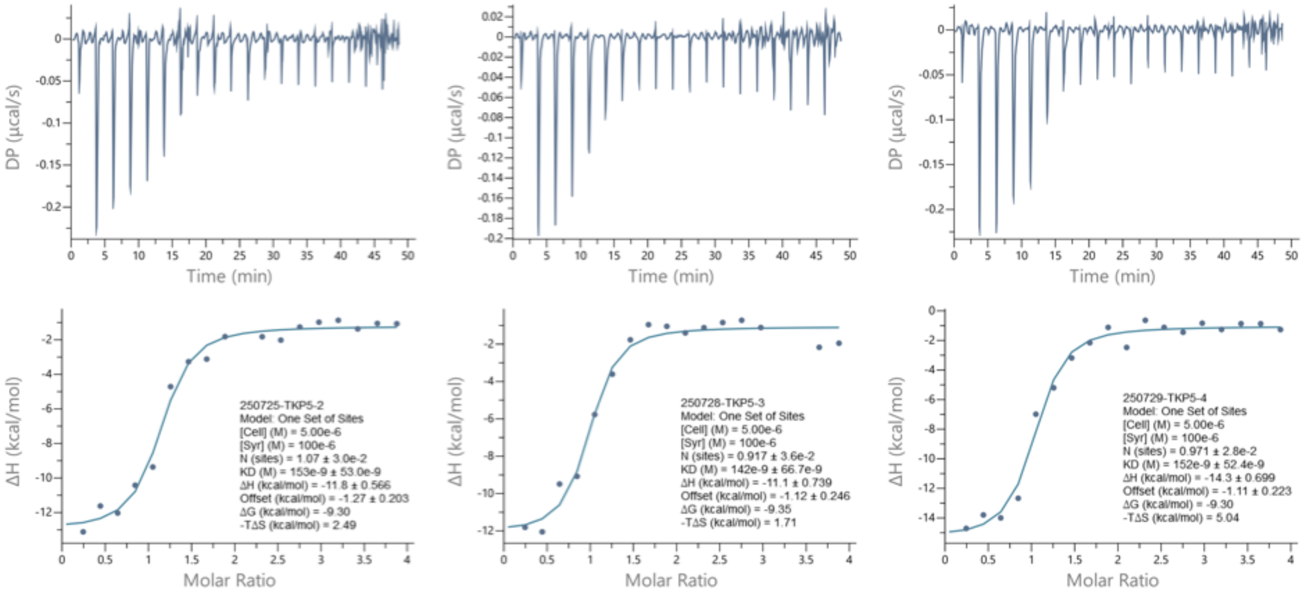
DP (µcal/s)
Time (min)
ΔH (kcal/mol)
Molar Ratio
250725-TKP5-2
Model: One Set of Sites
[Cell] (M) = 5.00e-6
[Syr] (M) = 100e-6
N (sites) = 1.07 ± 3.0e-2
KD (M) = 153e-9 ± 53.0e-9
ΔH (kcal/mol) = -11.8 ± 0.566
Offset (kcal/mol) = -1.27 ± 0.203
ΔG (kcal/mol) = -9.30
-TΔS (kcal/mol) = 2.49
250728-TKP5-3
Model: One Set of Sites
[Cell] (M) = 5.00e-6
[Syr] (M) = 100e-6
N (sites) = 0.917 ± 3.6e-2
KD (M) = 142e-9 ± 66.7e-9
ΔH (kcal/mol) = -11.1 ± 0.739
Offset (kcal/mol) = -1.12 ± 0.246
ΔG (kcal/mol) = -9.35
-TΔS (kcal/mol) = 1.71
250729-TKP5-4
Model: One Set of Sites
[Cell] (M) = 5.00e-6
[Syr] (M) = 100e-6
N (sites) = 0.971 ± 2.8e-2
KD (M) = 152e-9 ± 52.4e-9
ΔH (kcal/mol) = -14.3 ± 0.699
Offset (kcal/mol) = -1.11 ± 0.223
ΔG (kcal/mol) = -9.30
-TΔS (kcal/mol) = 5.04

(b) **TKP-6**

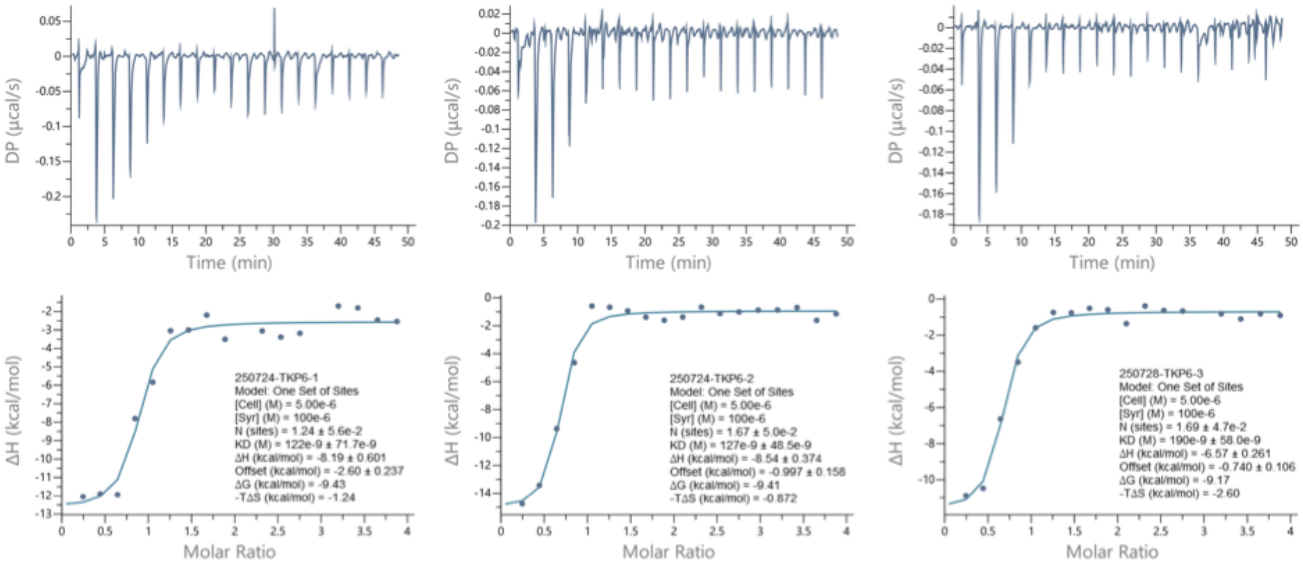
DP (µcal/s)
Time (min)
ΔH (kcal/mol)
Molar Ratio
250724-TKP6-1
Model: One Set of Sites
[Cell] (M) = 5.00e-6
[Syr] (M) = 100e-6
N (sites) = 1.24 ± 5.6e-2
KD (M) = 122e-9 ± 71.7e-9
ΔH (kcal/mol) = -8.19 ± 0.601
Offset (kcal/mol) = -2.60 ± 0.237
ΔG (kcal/mol) = -9.43
-TΔS (kcal/mol) = -1.24
250724-TKP6-2
Model: One Set of Sites
[Cell] (M) = 5.00e-6
[Syr] (M) = 100e-6
N (sites) = 1.67 ± 5.0e-2
KD (M) = 127e-9 ± 48.5e-9
ΔH (kcal/mol) = -8.54 ± 0.374
Offset (kcal/mol) = -0.997 ± 0.158
ΔG (kcal/mol) = -9.41
-TΔS (kcal/mol) = -0.872
250728-TKP6-3
Model: One Set of Sites
[Cell] (M) = 5.00e-6
[Syr] (M) = 100e-6
N (sites) = 1.69 ± 4.7e-2
KD (M) = 190e-9 ± 58.0e-9
ΔH (kcal/mol) = -6.57 ± 0.261
Offset (kcal/mol) = -0.740 ± 0.106
ΔG (kcal/mol) = -9.17
-TΔS (kcal/mol) = -2.60

(c) **TKP-14**

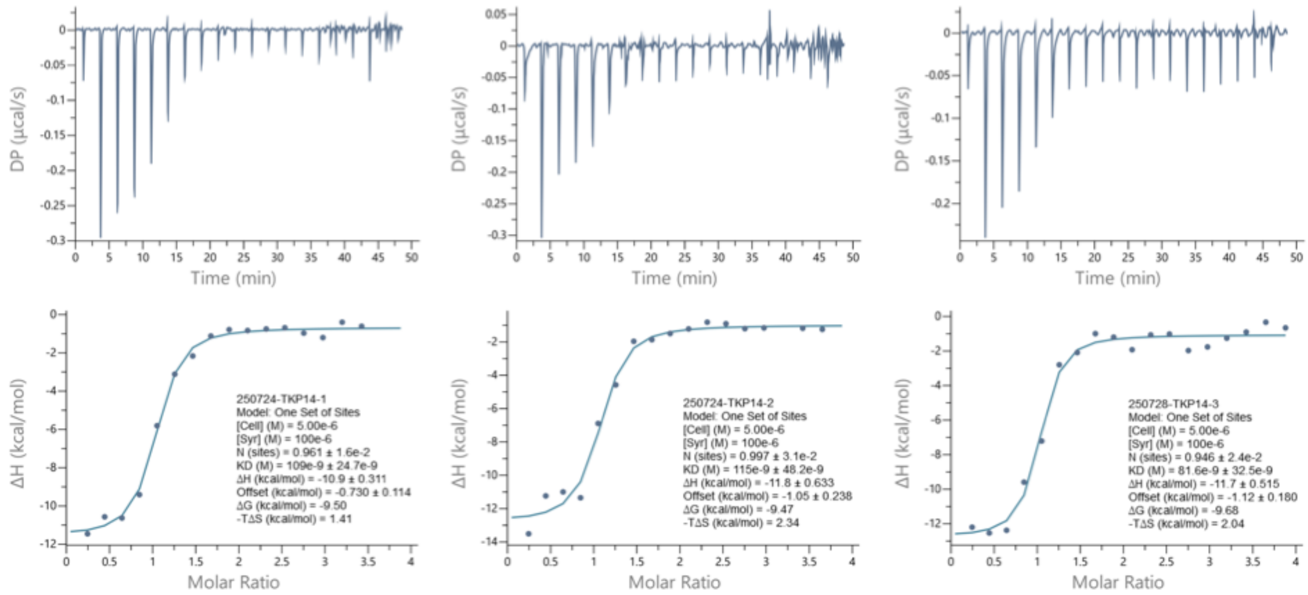

(d) **TKP-18**

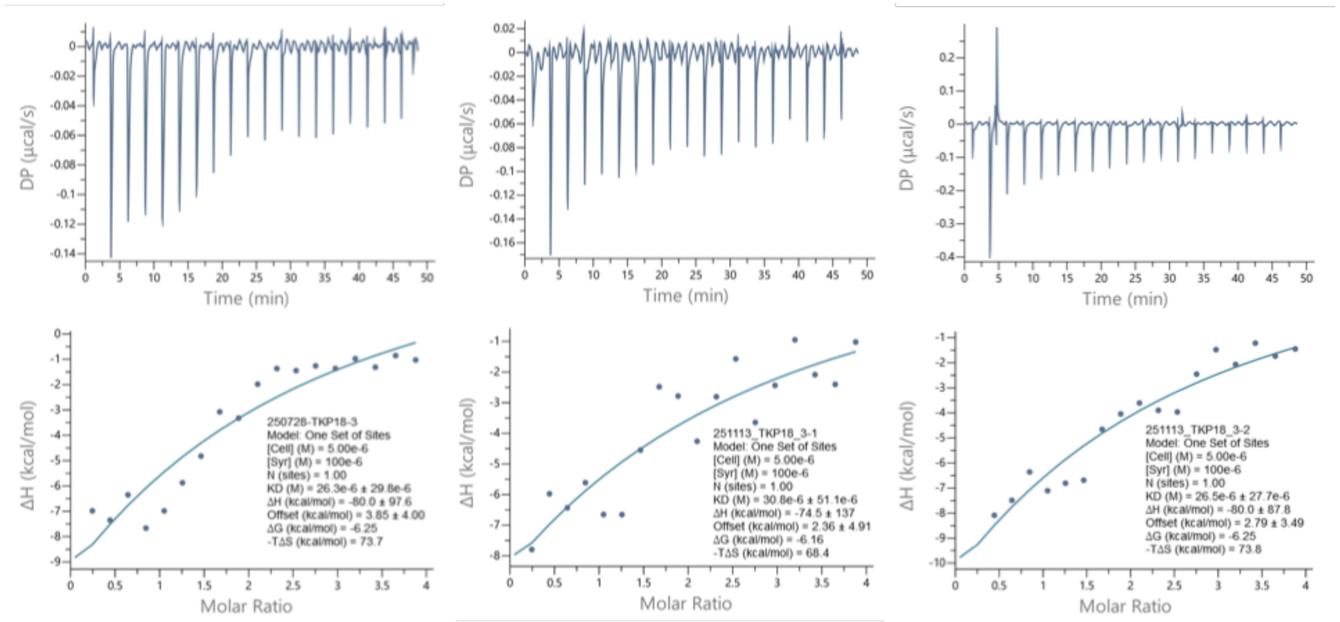

(e)

Compd.	*Kd* (μM)	Δ*G* (kcal/mol)	Δ*H* (kcal/mol)	-*T*Δ*S* (kcal/mol)	Number of measurements
TKP-5	0.15 ± 0.0035	-9.3 ± 0.017	-12 ± 0.97	3.1 ± 1.0	3
TKP-6	0.15 ± 0.021	-9.3 ± 0.084	-7.8 ± 0.61	-1.6 ± 0.53	3
TKP-14	0.10 ± 0.010	-9.6 ± 0.066	-12 ± 0.28	1.9 ± 0.27	3
TKP-18	28 ± 1.5	-6.2 ± 0.030	-78 ± 1.8	72 ± 1.8	3

Figure S2. Isothermal titration calorimetry (ITC) analysis of the interaction between BRD4-BD1 and TKP-compounds. (a) **TKP-5**. (b) **TKP-6**. (c) **TKP-14**. (d) **TKP-18**. (e) Summary table of the results shown in panels (a–d). Each measurement was performed in triplicate (n = 3).

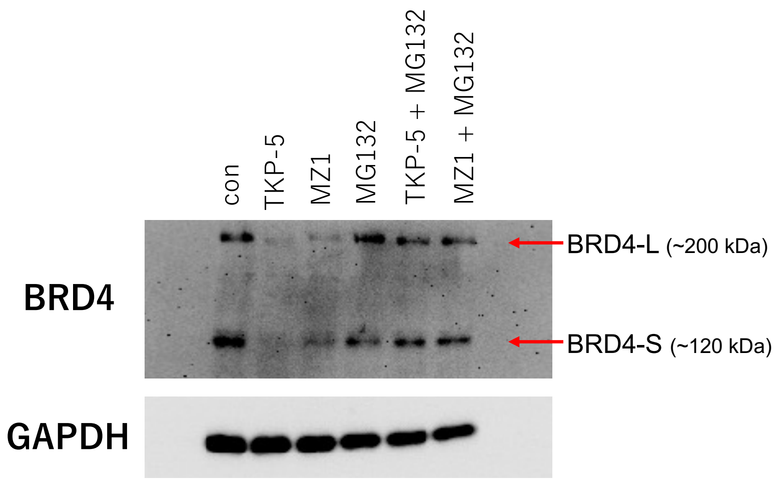

Figure S3. Effect of proteasome inhibitor on BRD4 degradation by **TKP-5**. BRD4 expression levels after 2-hour treatment with 1 μM PROTACs with or without 10 μM **MG132** were compared using DMSO vehicle as a control. BRD4, Bromodomain-containing protein 4; DMSO, Dimethyl sulfoxide.

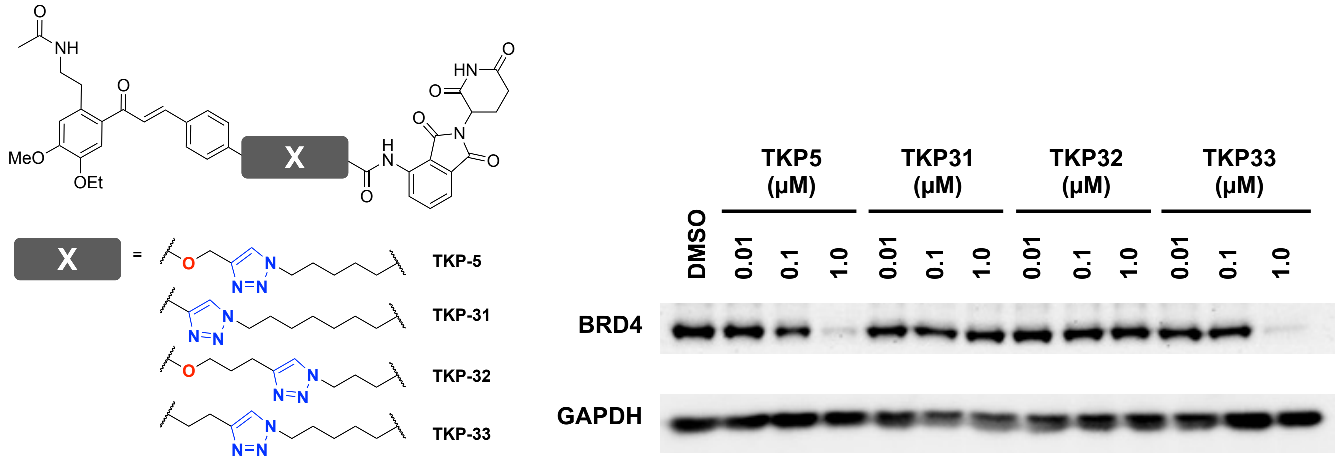

Figure S4. Western blot analysis of TKPs with triazole rings in different positions. KCMH-1 cells were treated with 1, 0.1, or 0.01 μM of each compound for 24 h.

a)

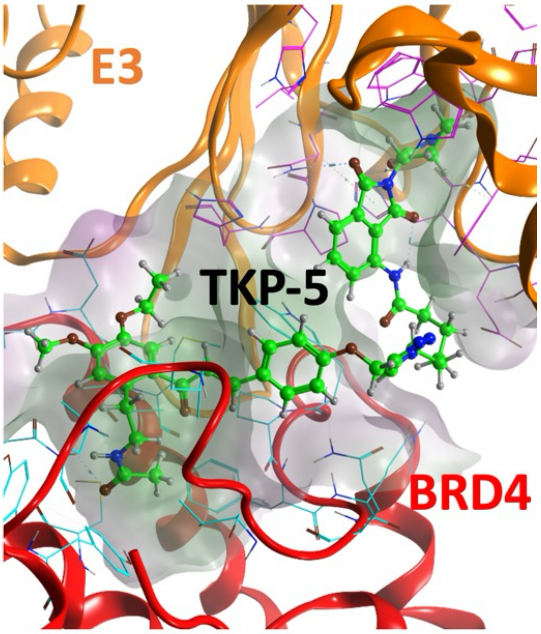

b)

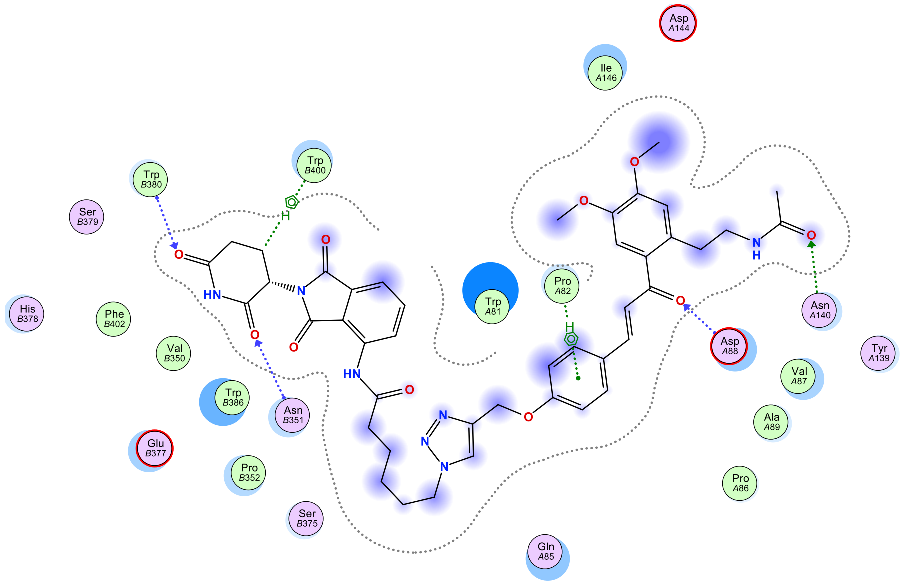

Figure S5. Docking model of **TKP-5** bound to CRBN–BRD4. The crystal structure of the DDB1B–CRBN–BRD4-BD1 complex co-crystallized with dBET23 was obtained from the Protein Data Bank (PDB ID: 6BN7), and monomers B and C were used for subsequent modeling. a) 3D view and b) 2D ligand interaction diagram using MOE ver.2024.0601. BRD4, Bromodomain-containing protein 4.

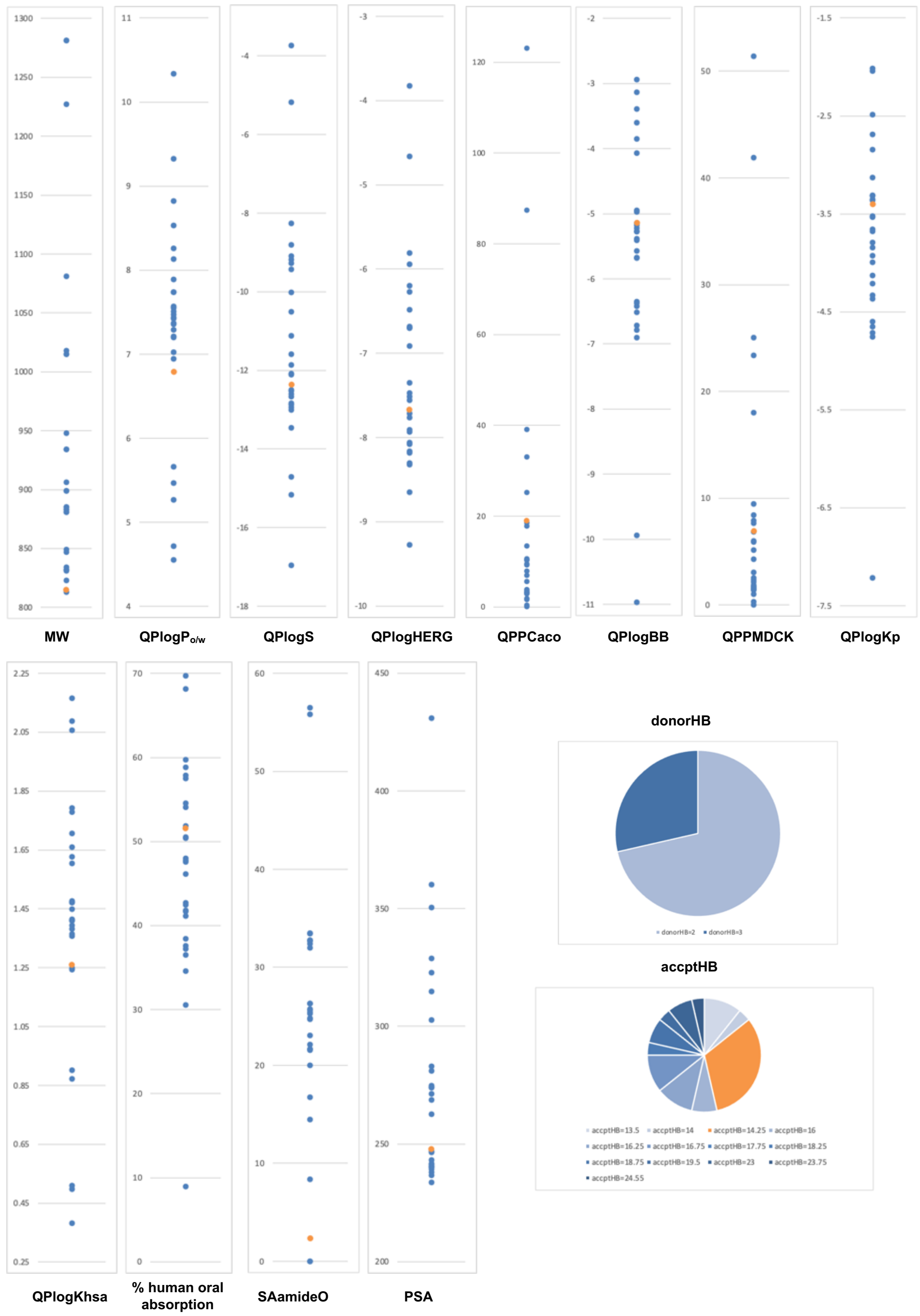

Figure S6. In silico prediction of physicochemical and ADMET-related properties using QikProp and Deep-PK. Predicted parameters include molecular weight (MW), lipophilicity (QPlogPo/w), aqueous solubility (QPlogS), Caco-2 cell permeability (QPPCaco), blood–brain barrier penetration (QPlogBB), MDCK cell permeability (QPPMDCK), skin permeability (QPlogKp), human serum albumin binding (QPlogKhsa), percent human oral absorption, amide oxygen surface area (SAamideO), polar surface area (PSA), and numbers of hydrogen bond donors (donorHB) and acceptors (acceptHB). The starting material used for diversity-generating transformations, TKP-21, is highlighted in orange, whereas the remaining compounds are shown in light blue.

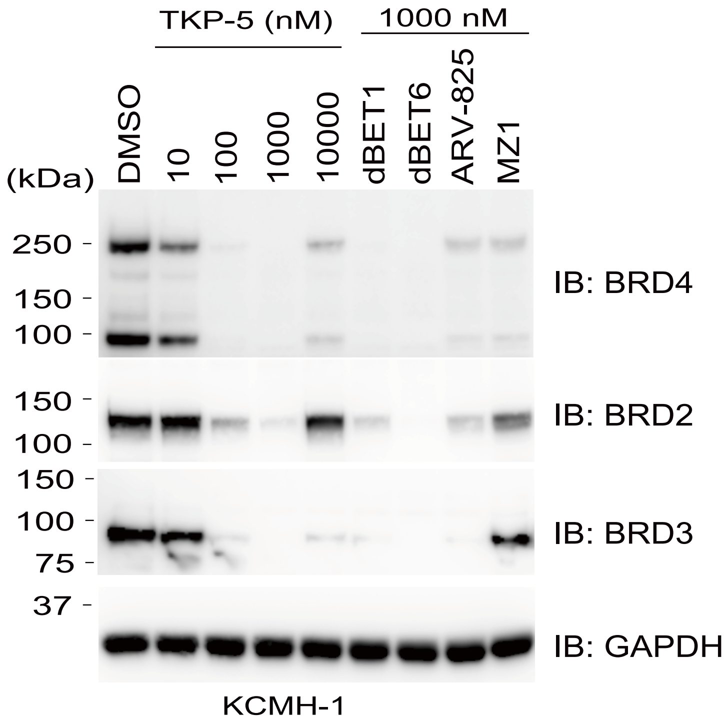

Figure S7. Western blot analysis of BRD2–4 degradation in KCMH-1 cells after 5-hour treatment with the compounds. Representative results are shown. BRD, Bromodomain-containing protein; DMSO, Dimethyl sulfoxide.

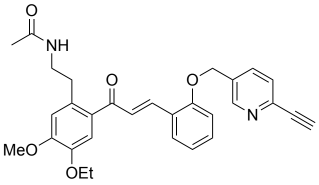

TK-351
[alkyne-modified **TK-285** (**1**)]

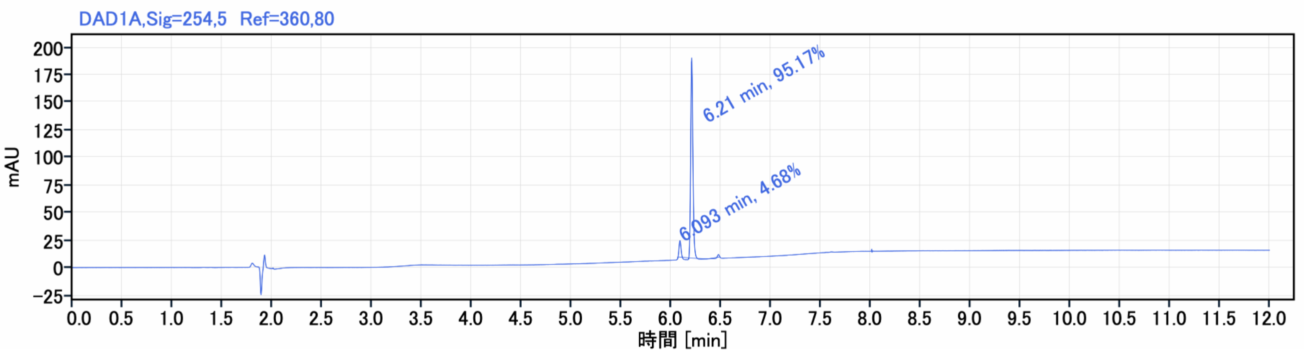

Analytical HPLC chromatogram of TK-351

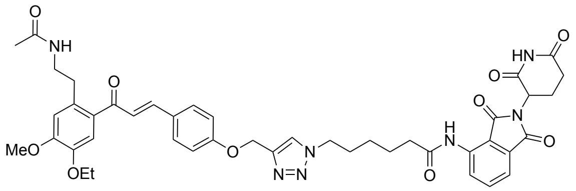

TKP-5

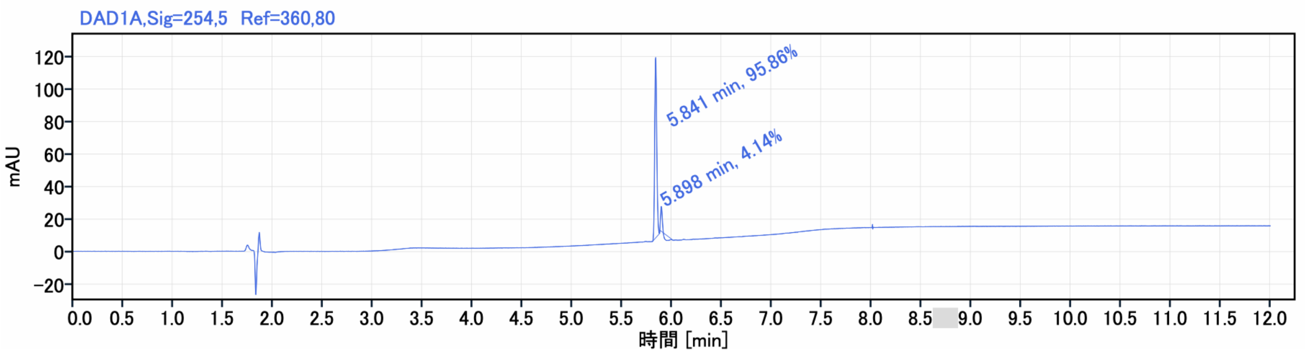

Analytical HPLC chromatogram of TKP-5

Figure S8. Analytical HPLC, performed on an Agilent LCMSD iQ system equipped with an Agilent InfinityLab Poroshell 120 EC-C18 column (2.7 µm, 4.6 × 100 mm) with diode array detection (DAD). Analyses were conducted under isocratic conditions using H_2O/MeCN (5:95, v/v) containing 0.1% formic acid at a flow rate of 0.80 mL min^{-1}.

2. Copies of ^{1}H and ^{13}C NMR spectra

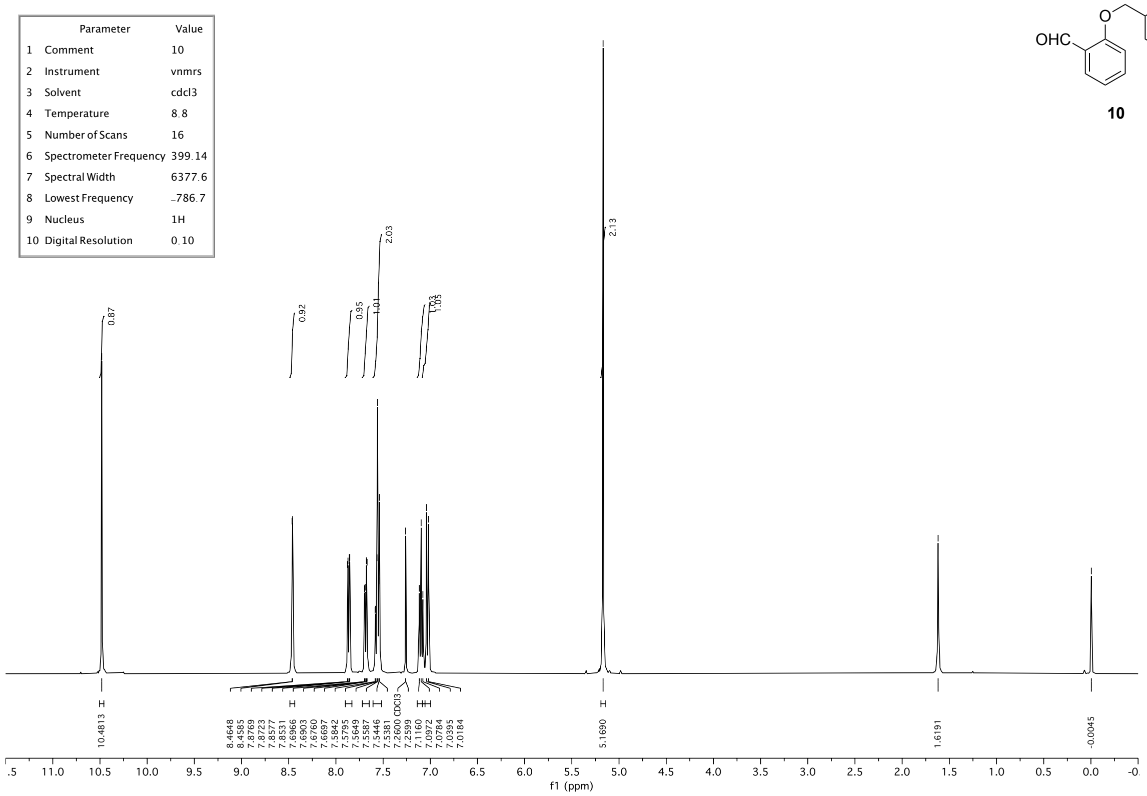

^{1}H NMR spectrum of compound **10**

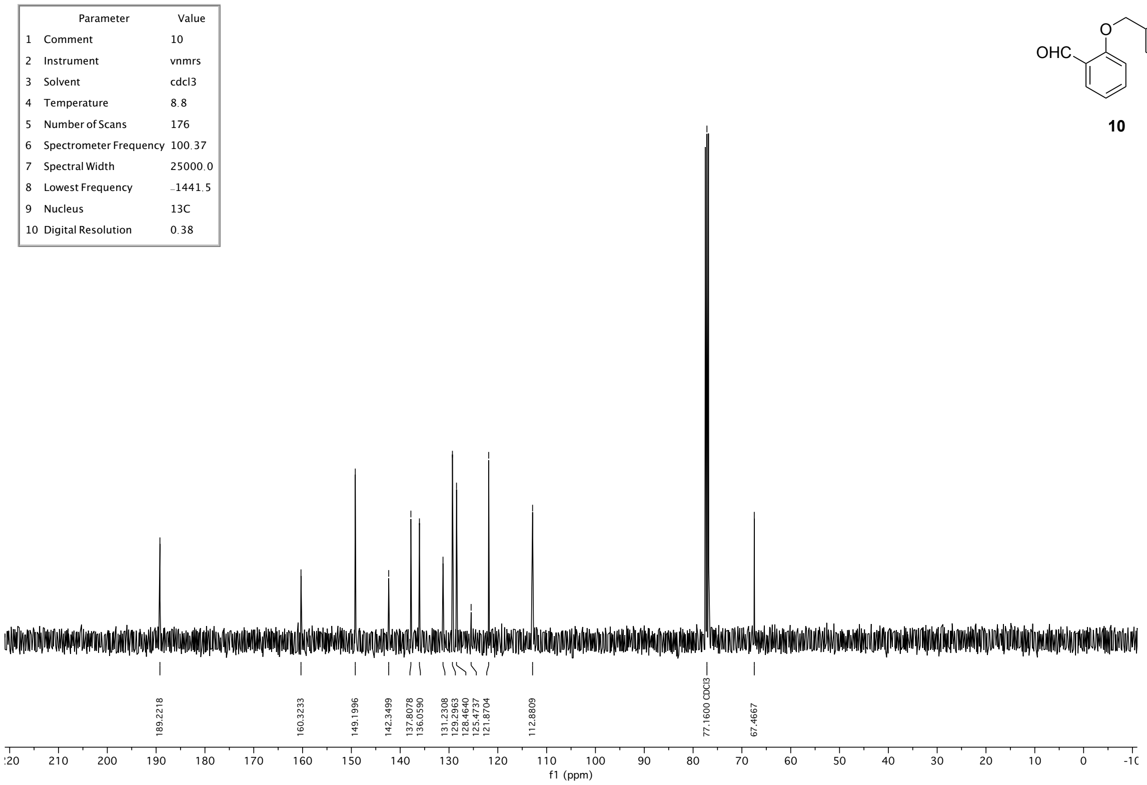

^{13}C NMR spectrum of compound **10**

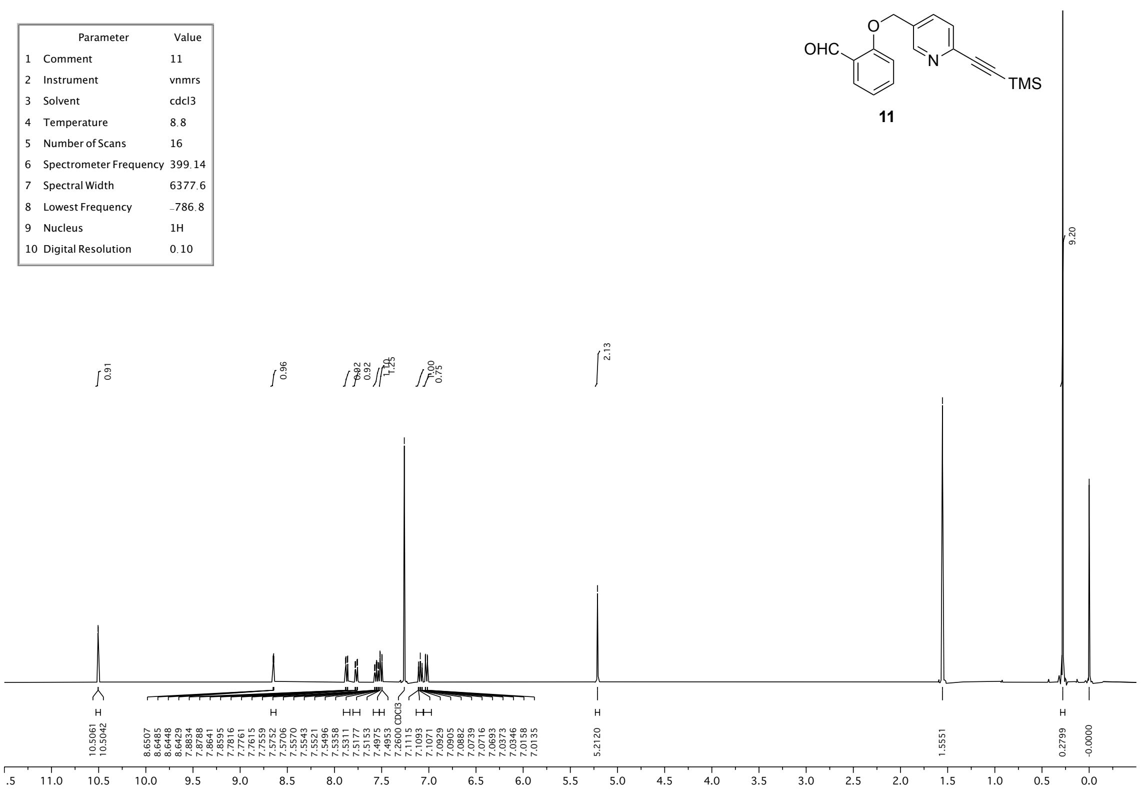

^{1}H NMR spectrum of compound **11**

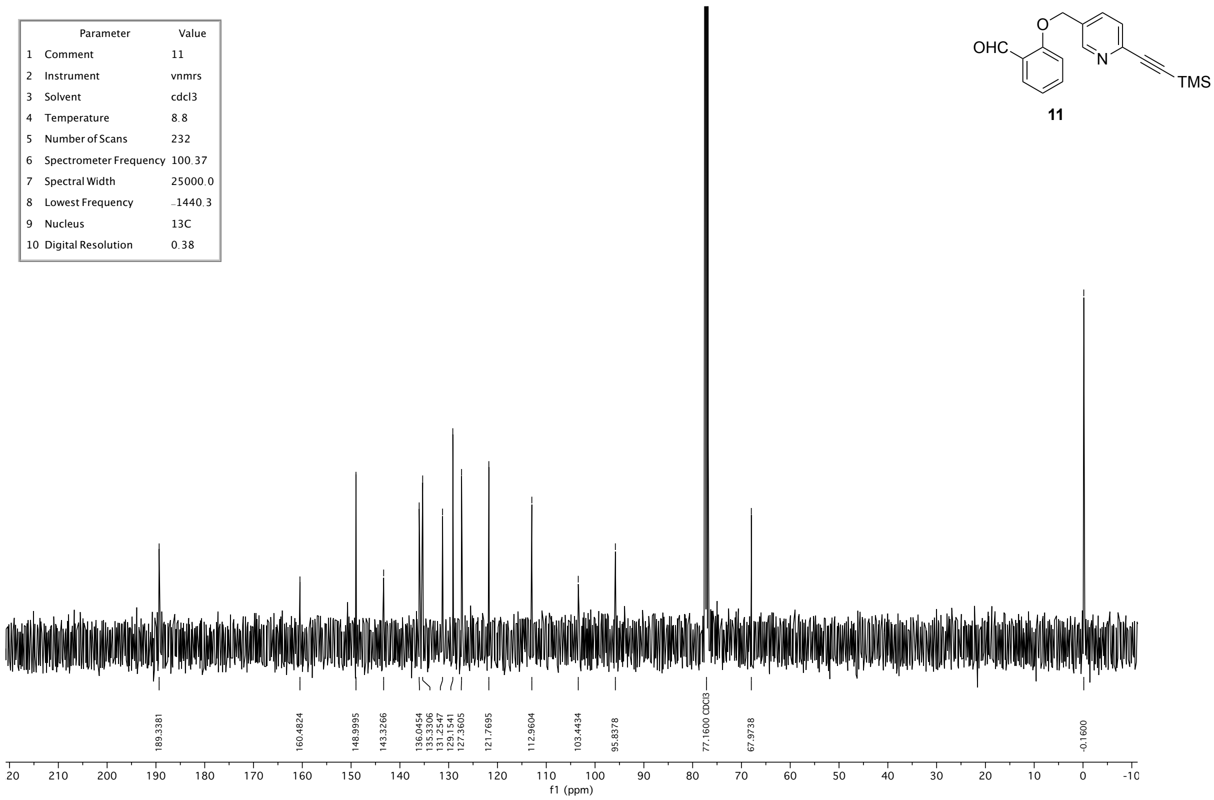

^{13}C NMR spectrum of compound **11**

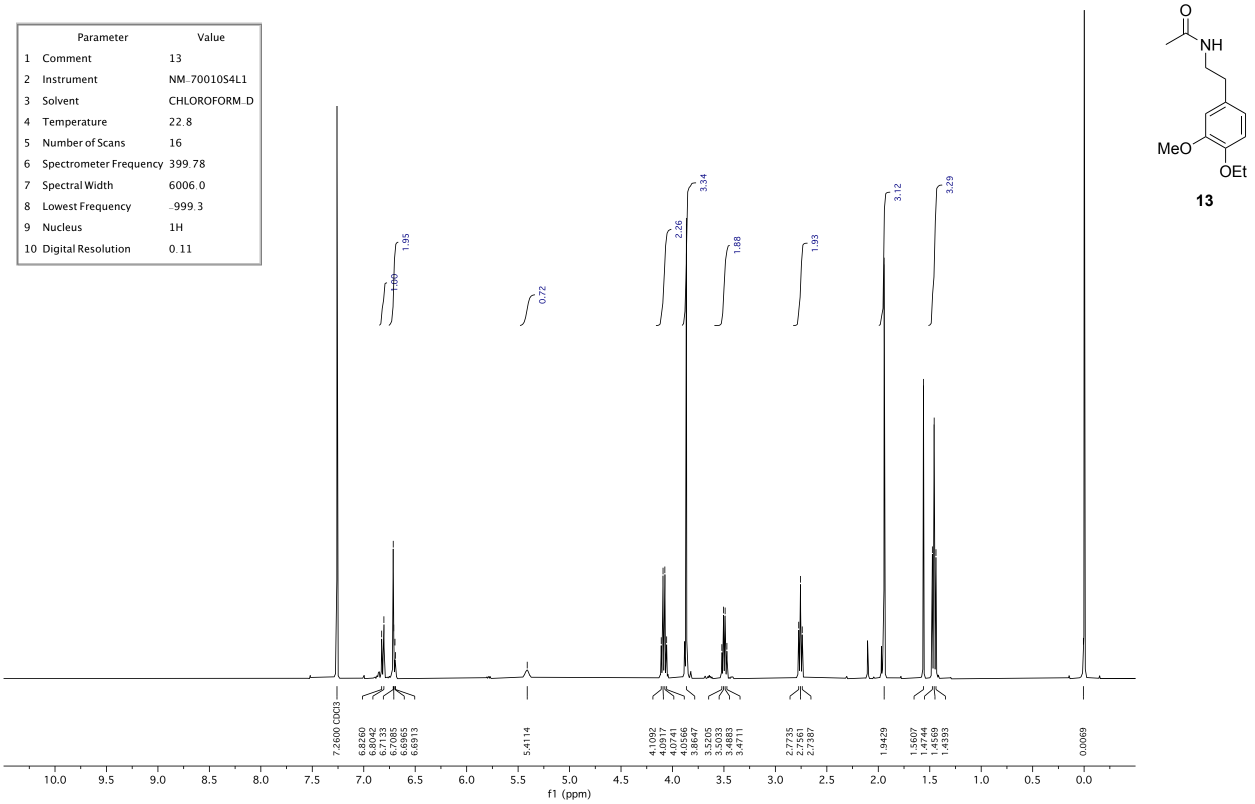

^{1}H NMR spectrum of compound **13**

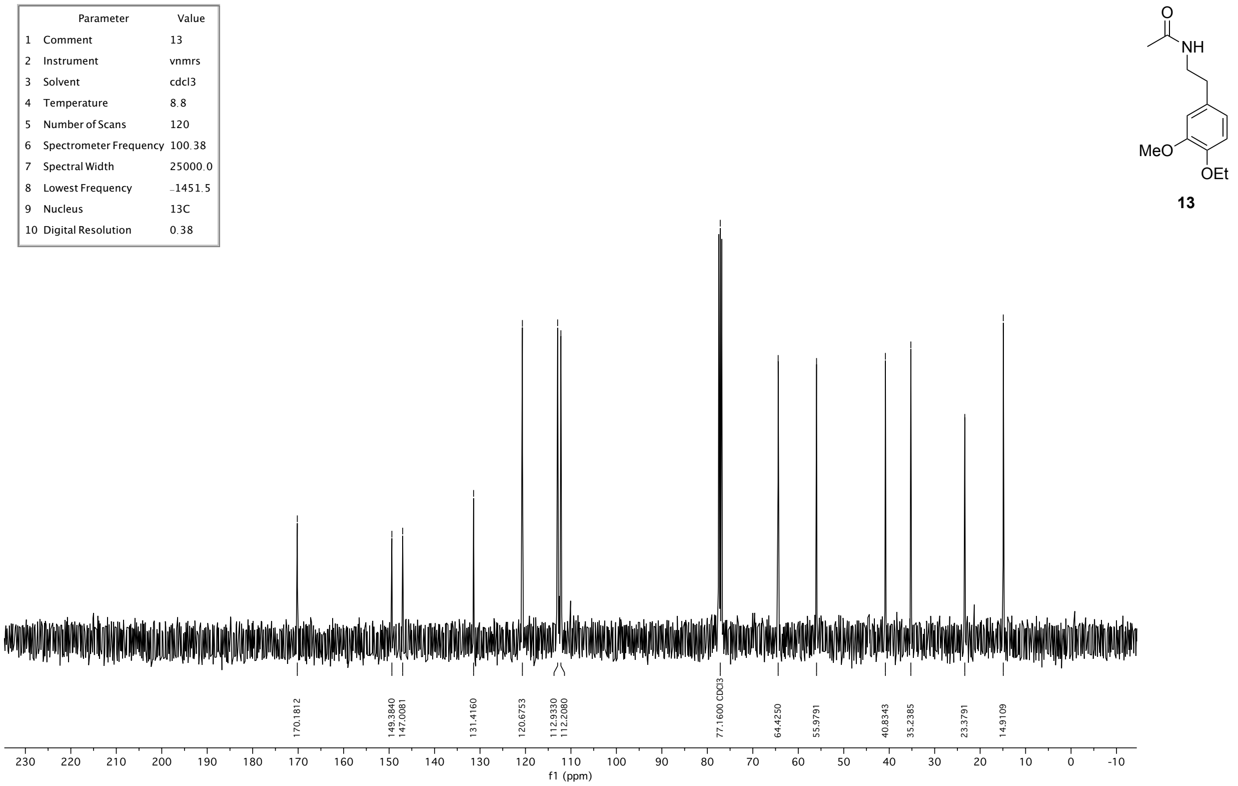

^{13}C NMR spectrum of compound **13**

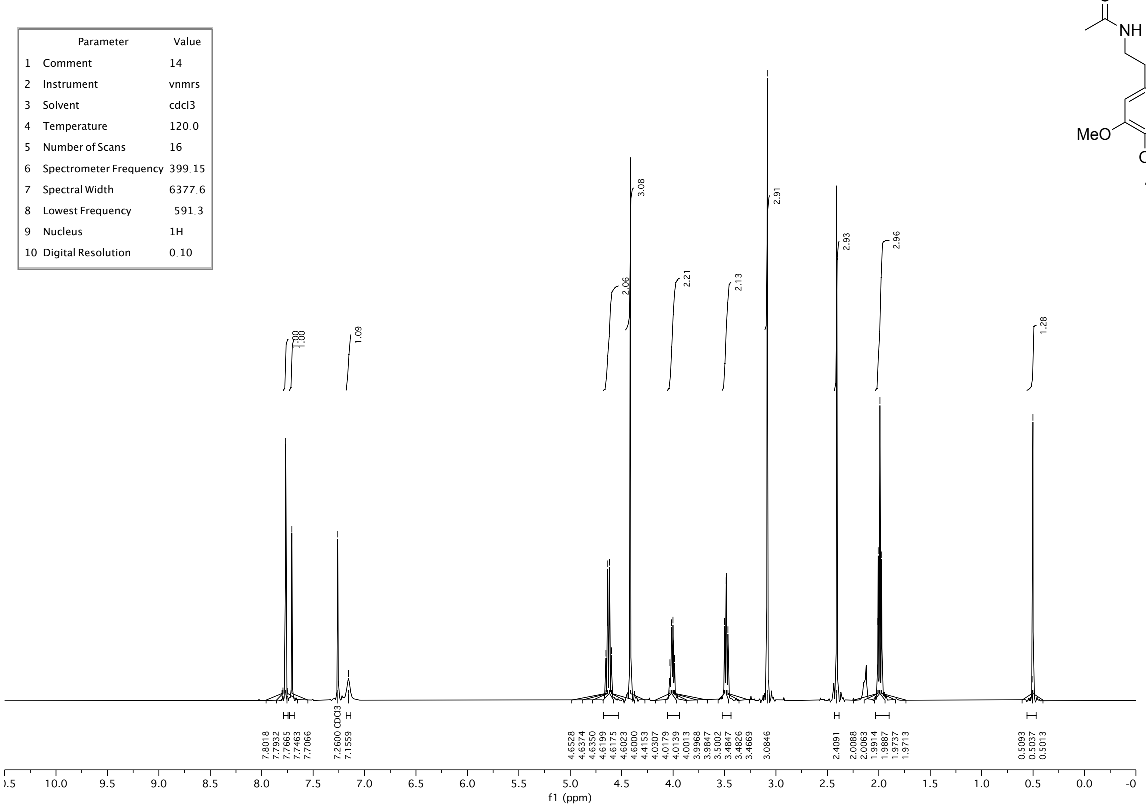

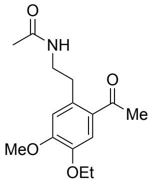

14

^{1}H NMR spectrum of compound **14**

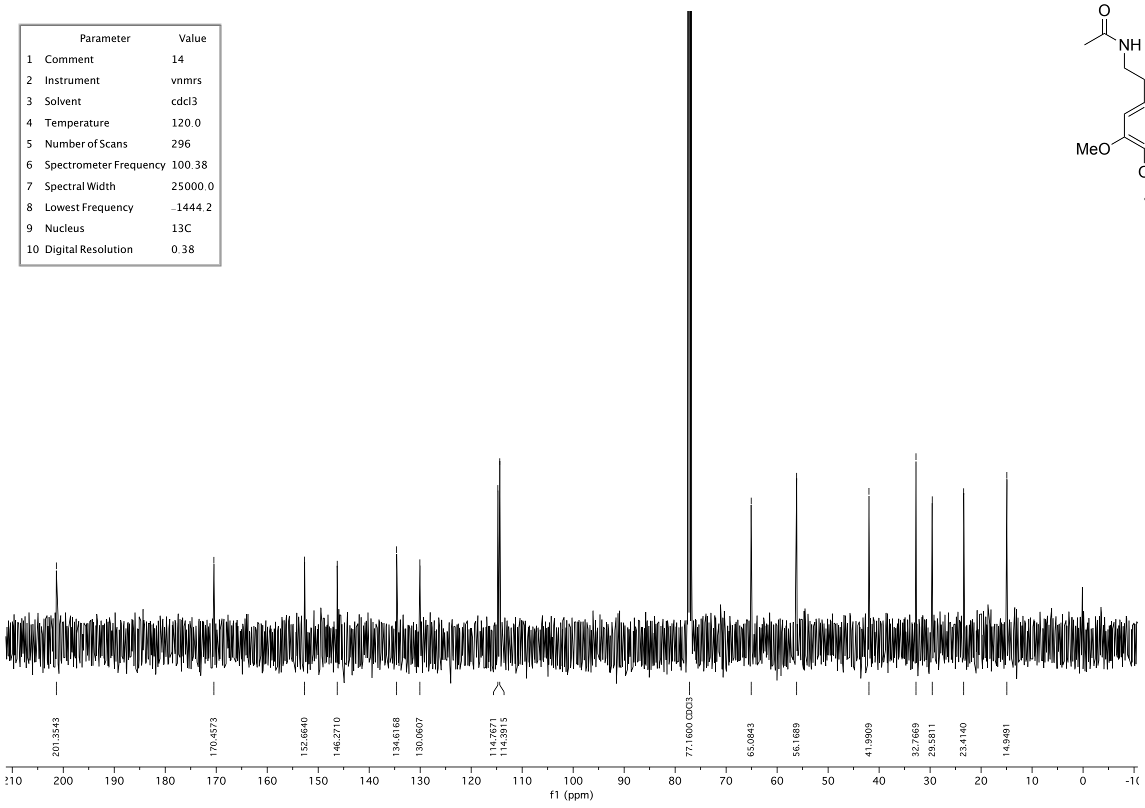

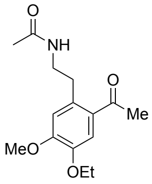

14

^{13}C NMR spectrum of compound **14**

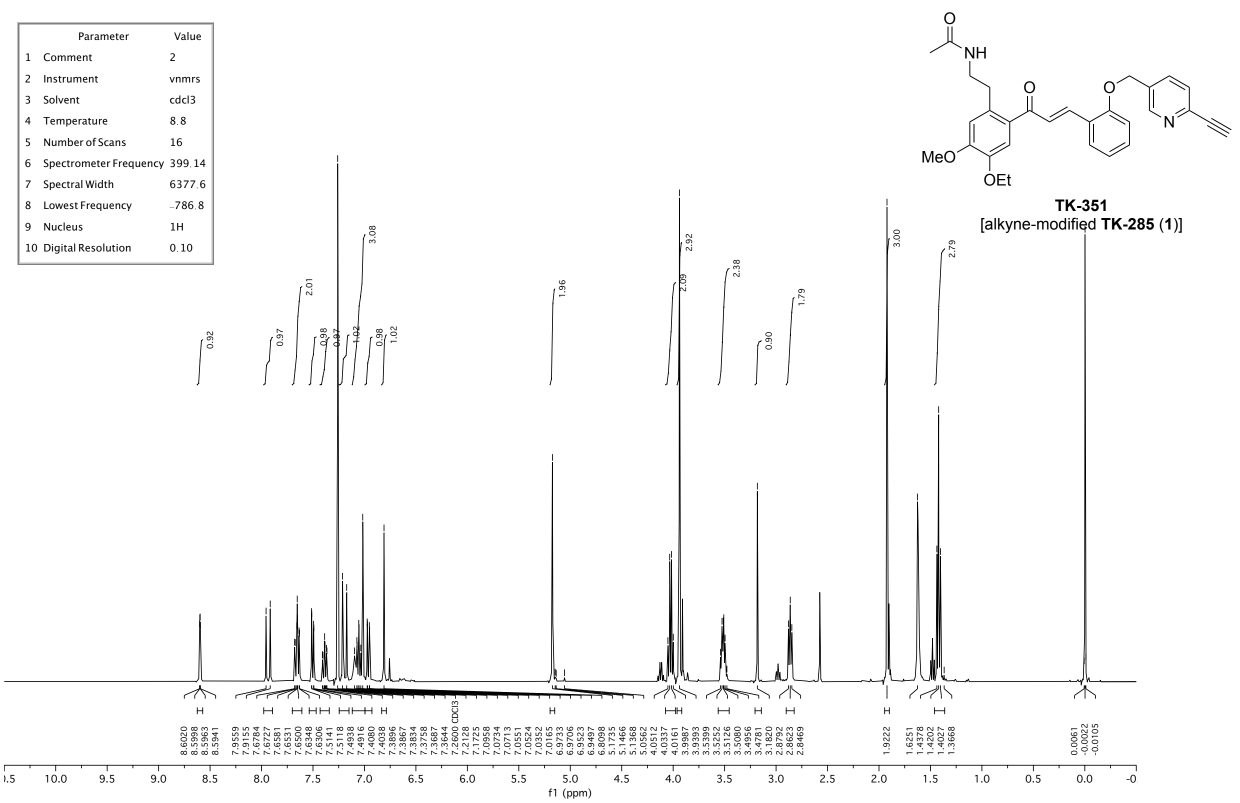

	Parameter	Value
1	Comment	2
2	Instrument	vnmrs
3	Solvent	cdcl3
4	Temperature	8.8
5	Number of Scans	16
6	Spectrometer Frequency	399.14
7	Spectral Width	6377.6
8	Lowest Frequency	-786.8
9	Nucleus	1H
10	Digital Resolution	0.10

^{1}H NMR spectrum of compound **1**

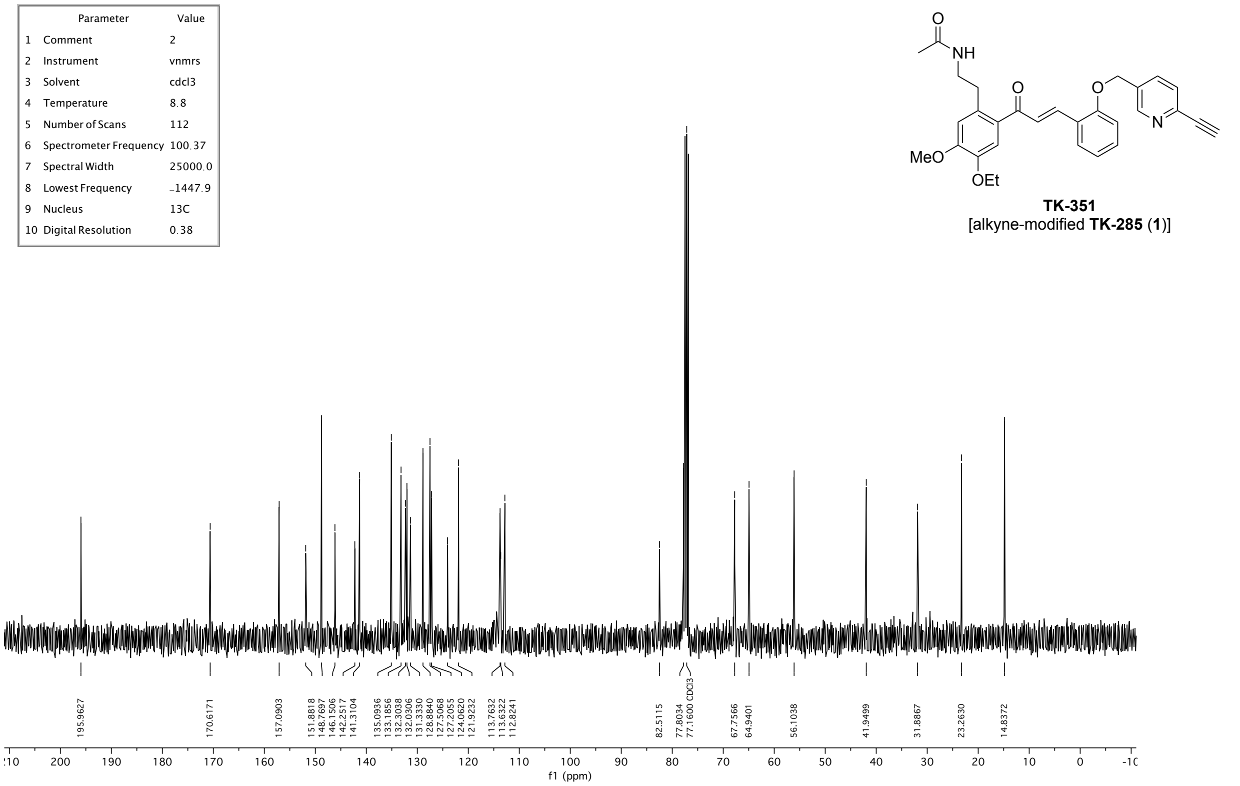

	Parameter	Value
1	Comment	2
2	Instrument	vnmrs
3	Solvent	cdcl3
4	Temperature	8.8
5	Number of Scans	112
6	Spectrometer Frequency	100.37
7	Spectral Width	25000.0
8	Lowest Frequency	-1447.9
9	Nucleus	13C
10	Digital Resolution	0.38

^{13}C NMR spectrum of compound **1**

	Parameter	Value
1	Comment	3
2	Instrument	vnmrs
3	Solvent	cdcl3
4	Temperature	120.0
5	Number of Scans	16
6	Spectrometer Frequency	399.15
7	Spectral Width	6377.6
8	Lowest Frequency	-793.5
9	Nucleus	1H
10	Digital Resolution	0.10

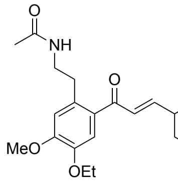

3

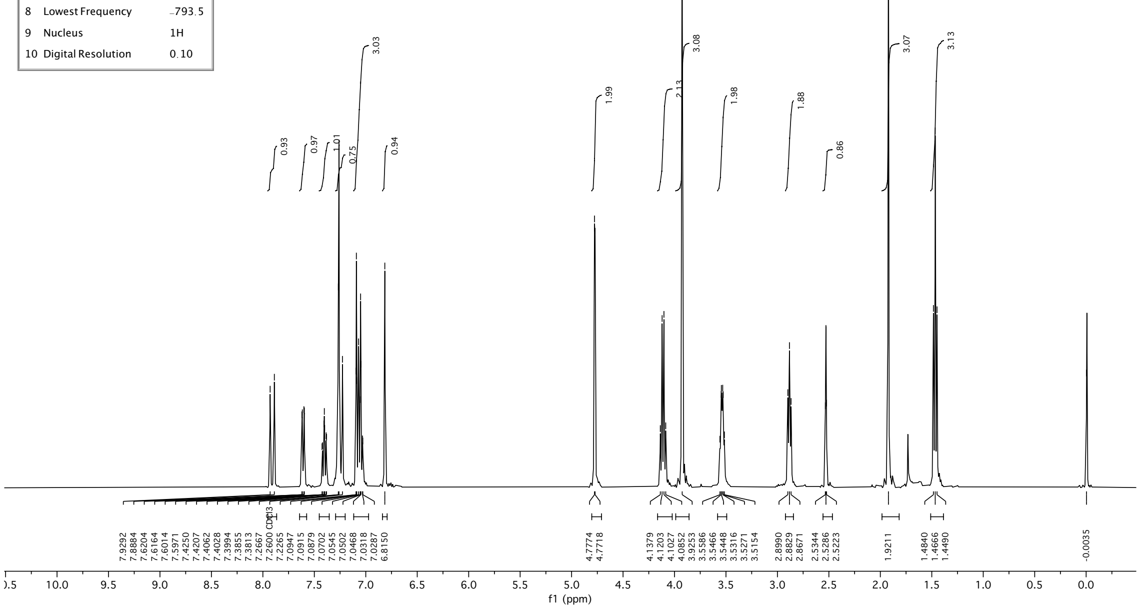

^{1}H NMR spectrum of compound **3**

	Parameter	Value
1	Comment	3
2	Instrument	vnmrs
3	Solvent	cdcl3
4	Temperature	120.0
5	Number of Scans	232
6	Spectrometer Frequency	100.38
7	Spectral Width	25000.0
8	Lowest Frequency	-1445.4
9	Nucleus	13C
10	Digital Resolution	0.38

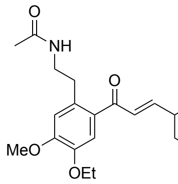

3

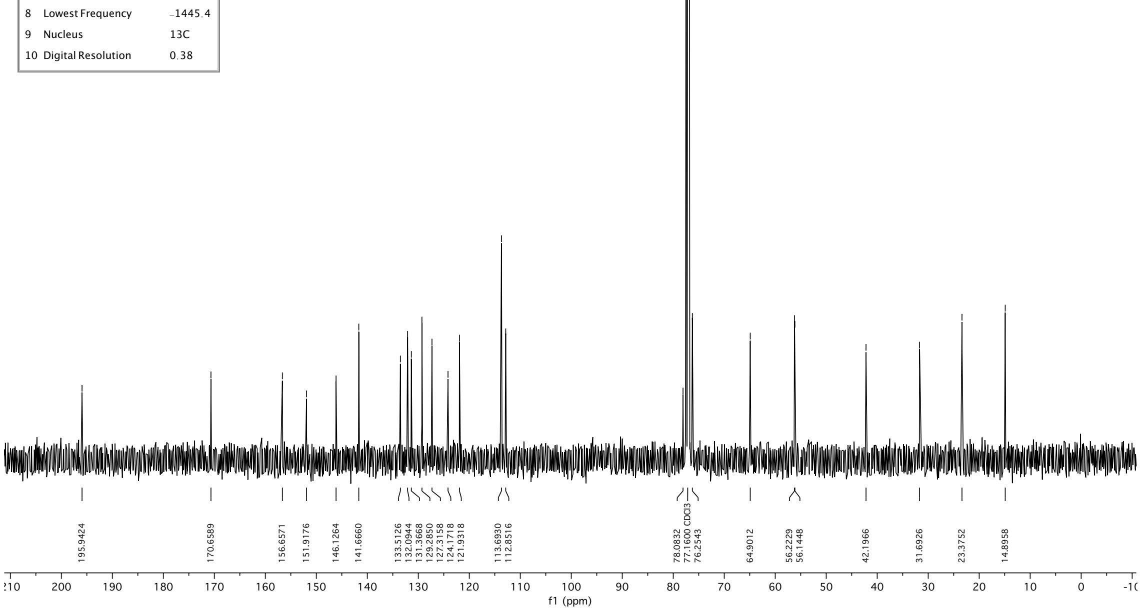

^{13}C NMR spectrum of compound **3**

	Parameter	Value
1	Comment	4
2	Instrument	vnmrs
3	Solvent	cdcl3
4	Temperature	8.8
5	Number of Scans	16
6	Spectrometer Frequency	399.14
7	Spectral Width	6377.6
8	Lowest Frequency	-794.0
9	Nucleus	1H
10	Digital Resolution	0.10

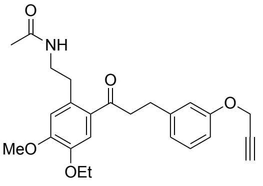

4

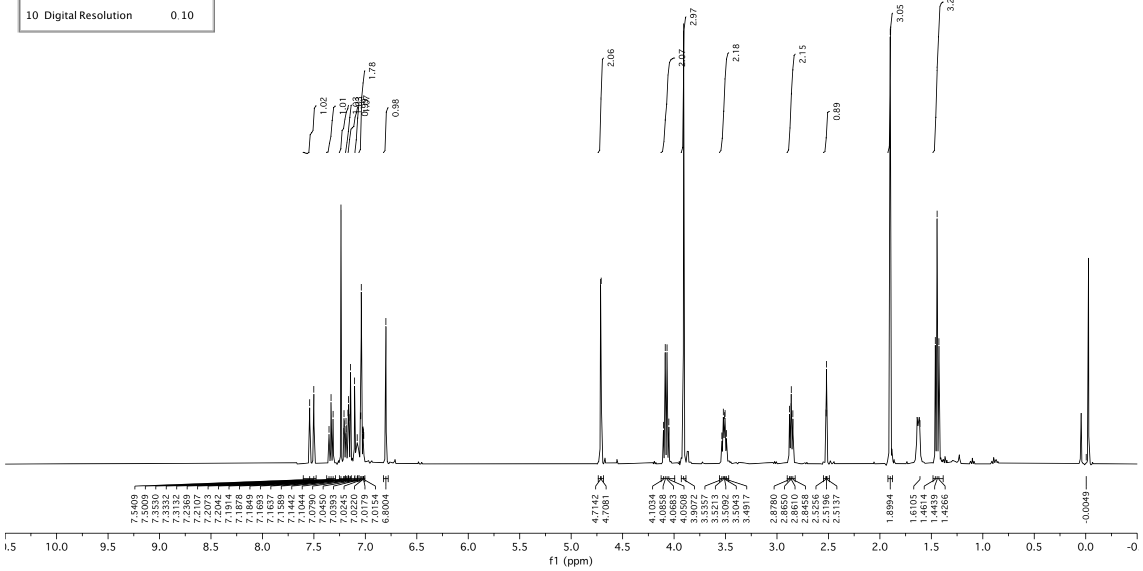

^{1}H NMR spectrum of compound **4**

	Parameter	Value
1	Comment	4
2	Instrument	vnmrs
3	Solvent	cdcl3
4	Temperature	8.8
5	Number of Scans	184
6	Spectrometer Frequency	100.37
7	Spectral Width	25000.0
8	Lowest Frequency	-1441.9
9	Nucleus	13C
10	Digital Resolution	0.38

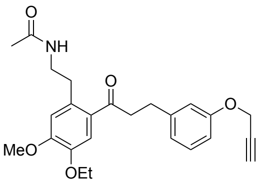

4

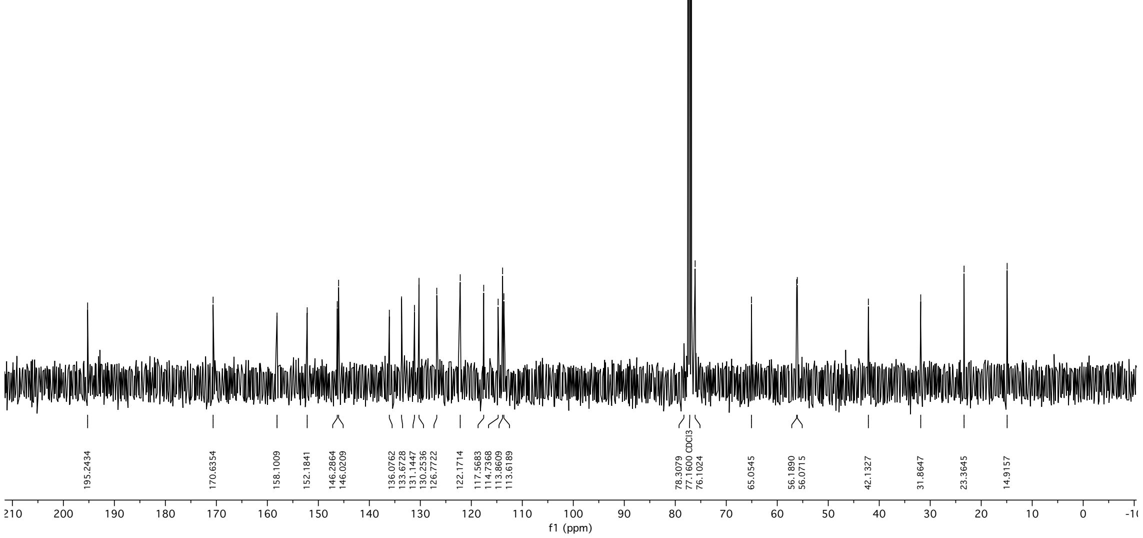

^{13}C NMR spectrum of compound **4**

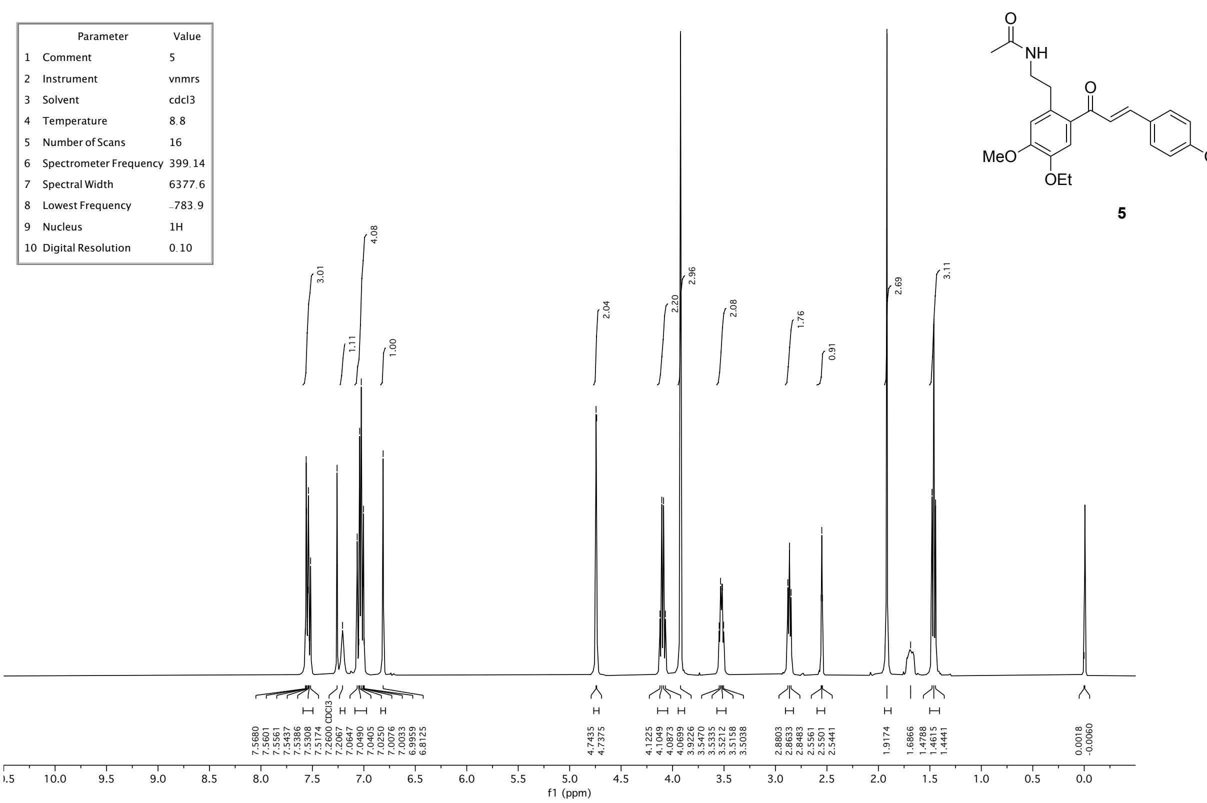

^{1}H NMR spectrum of compound **5**

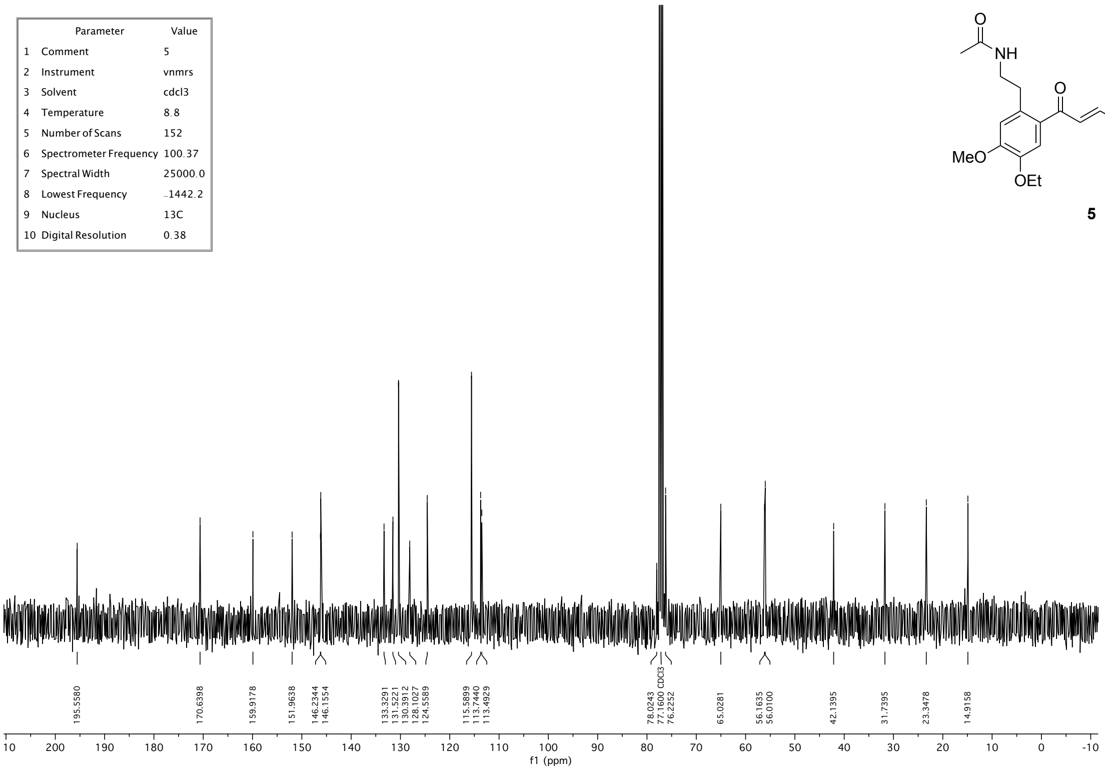

^{13}C NMR spectrum of compound **5**

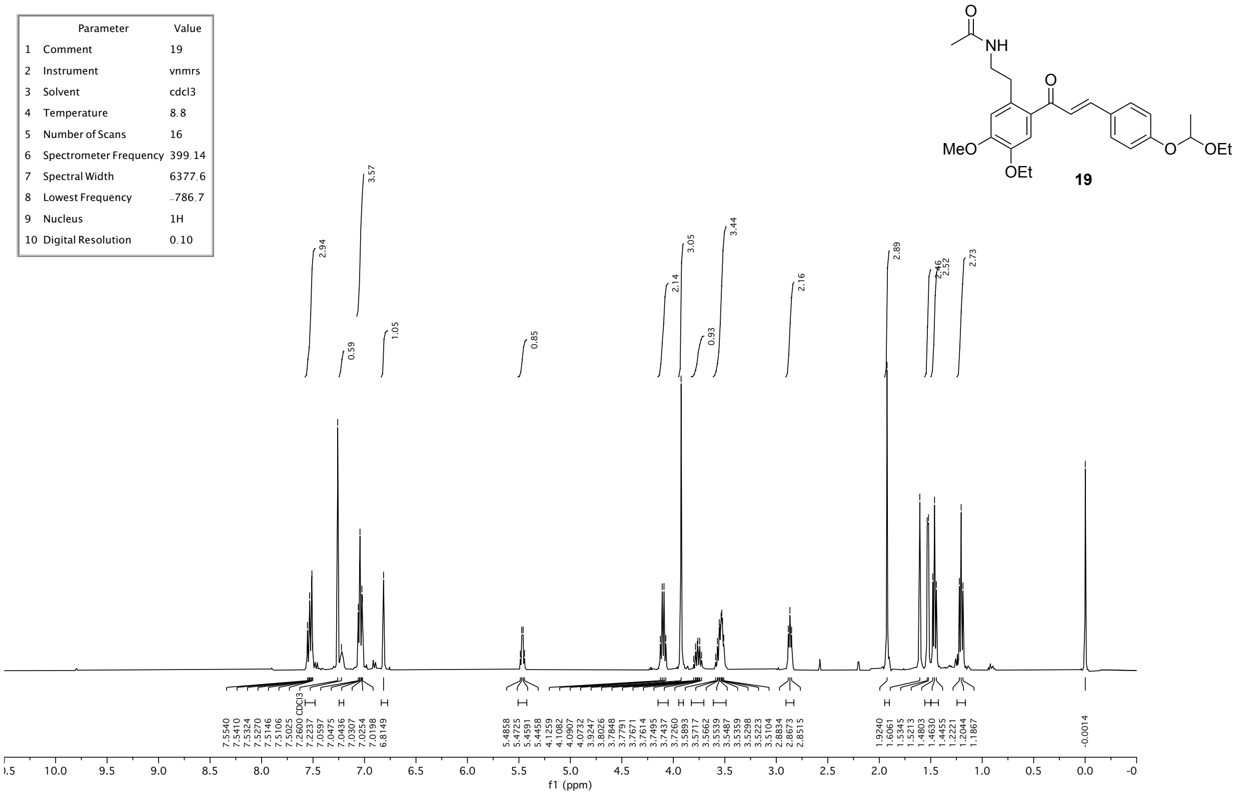

^{1}H NMR spectrum of compound **19**

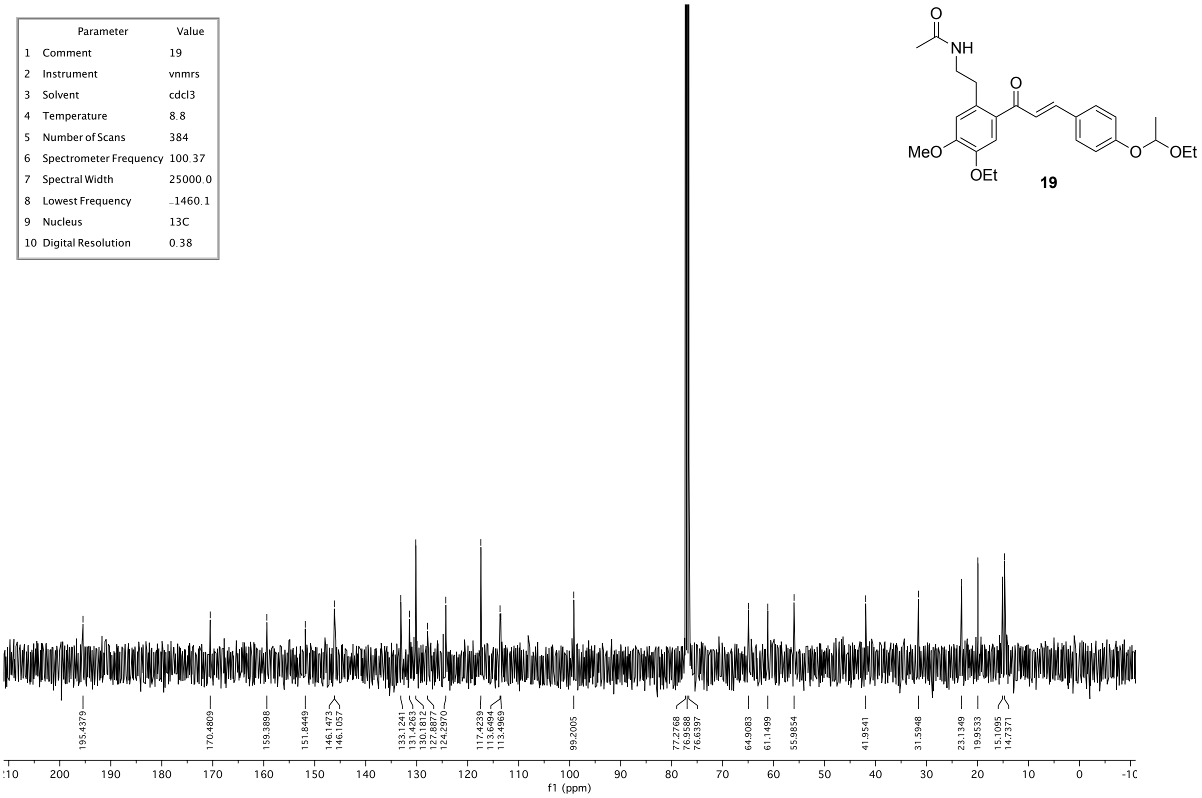

^{13}C NMR spectrum of compound **19**

	Parameter	Value
1	Comment	20
2	Instrument	vnmrs
3	Solvent	cdcl3
4	Temperature	8.8
5	Number of Scans	16
6	Spectrometer Frequency	399.14
7	Spectral Width	6377.6
8	Lowest Frequency	-786.8
9	Nucleus	1H
10	Digital Resolution	0.10

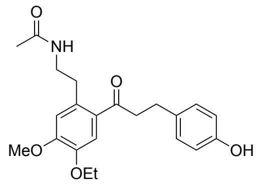

20

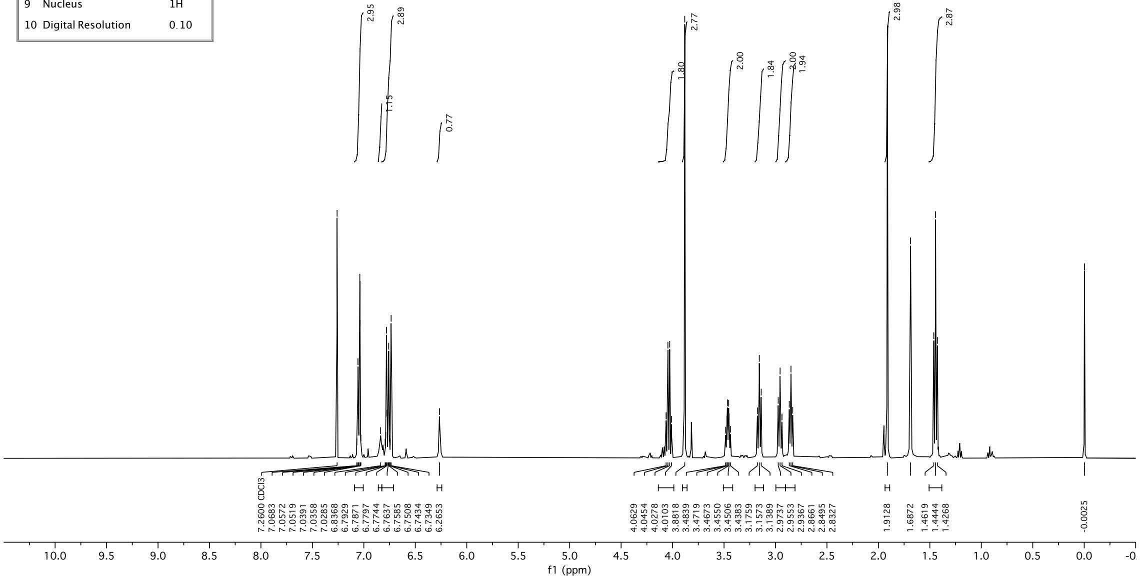

^{1}H NMR spectrum of compound **20**

	Parameter	Value
1	Comment	20
2	Instrument	vnmrs
3	Solvent	cdcl3
4	Temperature	8.8
5	Number of Scans	192
6	Spectrometer Frequency	100.37
7	Spectral Width	25000.0
8	Lowest Frequency	-1460.1
9	Nucleus	13C
10	Digital Resolution	0.38

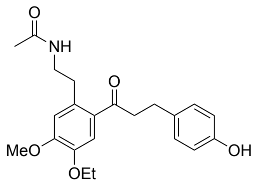

20

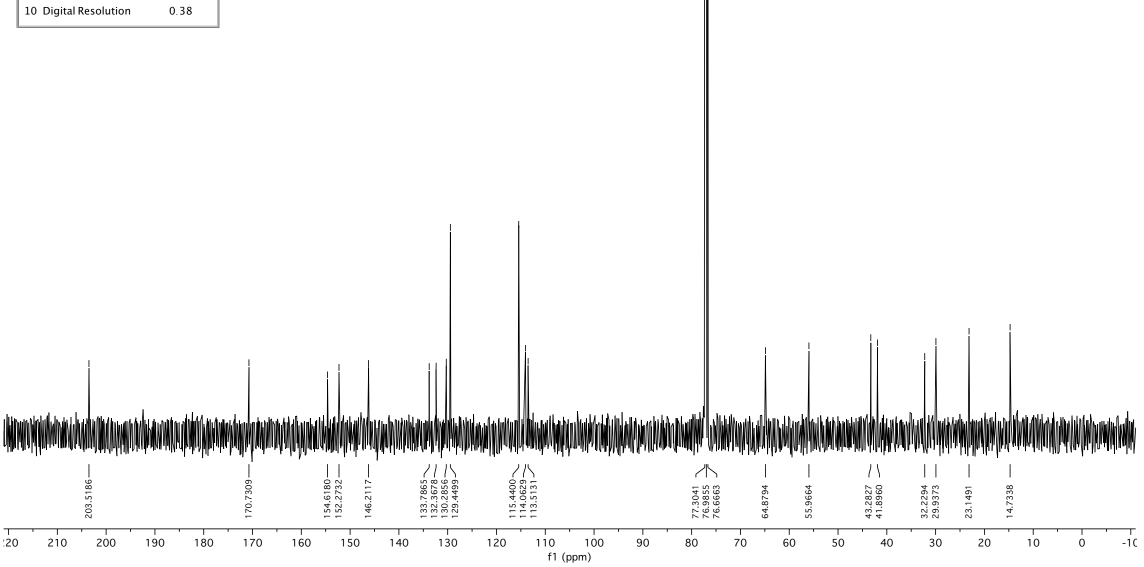

^{13}C NMR spectrum of compound **20**

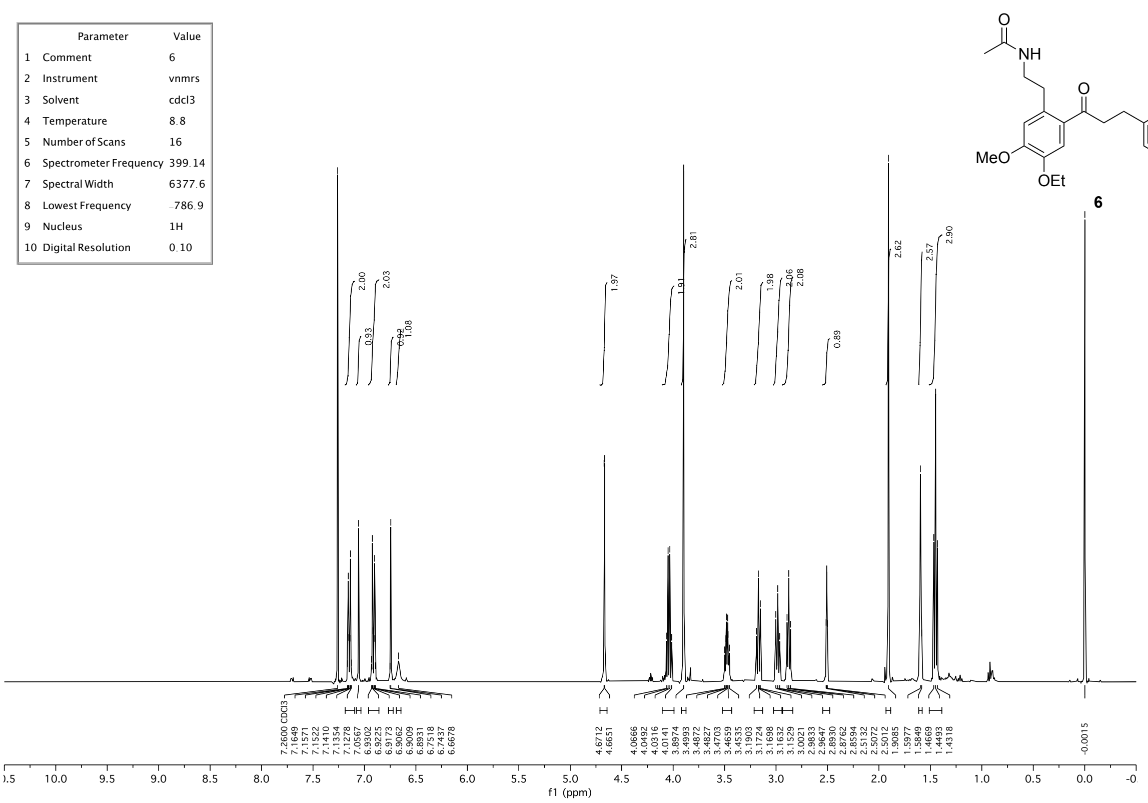

^{1}H NMR spectrum of compound **6**

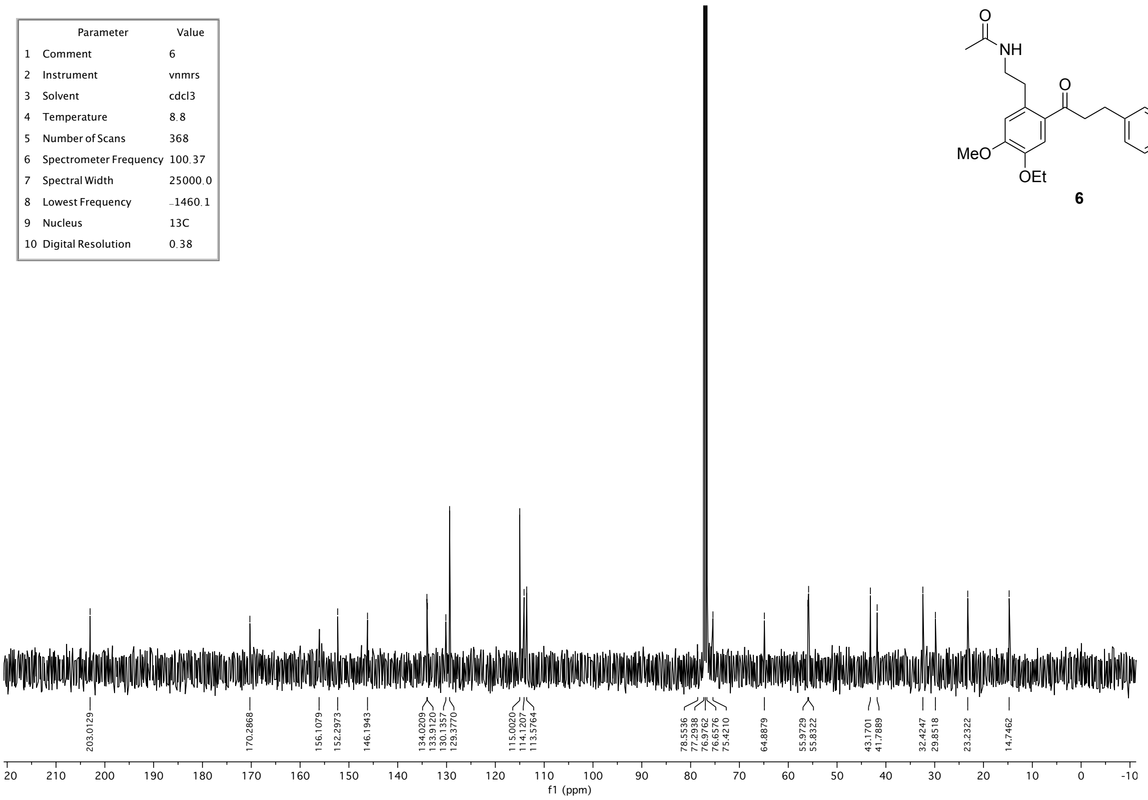

^{13}C NMR spectrum of compound **6**

	Parameter	Value
1	Comment	7
2	Instrument	vnmrs
3	Solvent	cdcl3
4	Temperature	8.8
5	Number of Scans	16
6	Spectrometer Frequency	399.14
7	Spectral Width	6377.6
8	Lowest Frequency	-786.3
9	Nucleus	1H
10	Digital Resolution	0.10

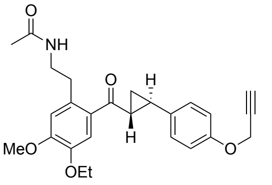

7

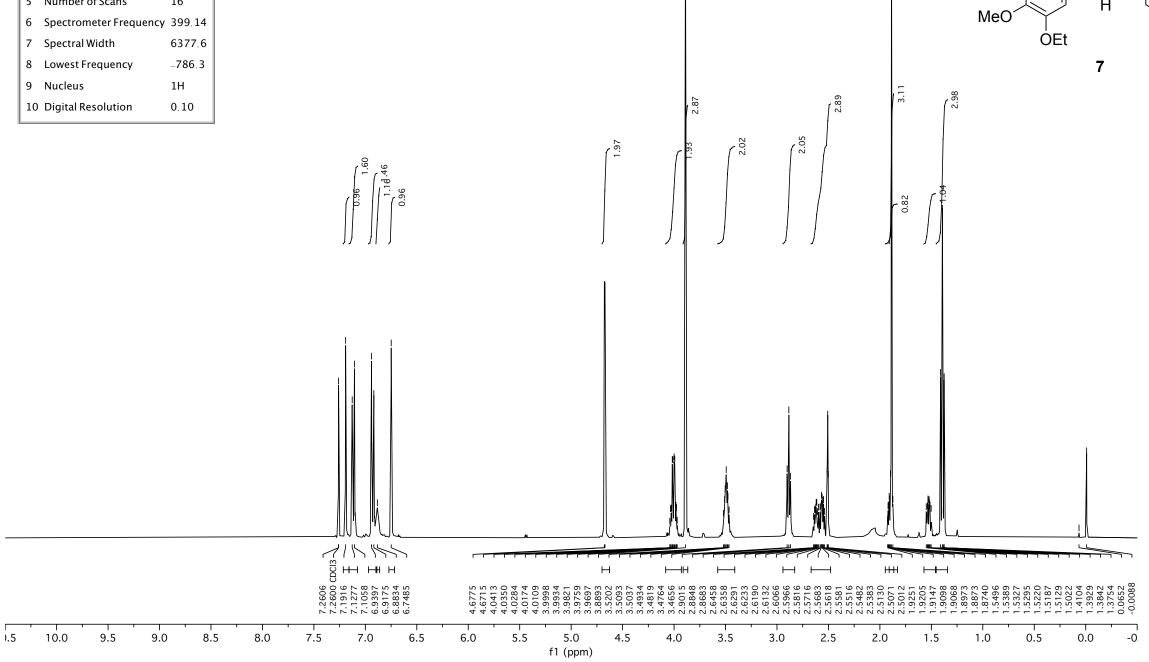

^{1}H NMR spectrum of compound **7**

	Parameter	Value
1	Comment	7
2	Instrument	vnmrs
3	Solvent	cdcl3
4	Temperature	8.8
5	Number of Scans	88
6	Spectrometer Frequency	100.37
7	Spectral Width	25000.0
8	Lowest Frequency	-1443.8
9	Nucleus	13C
10	Digital Resolution	0.38

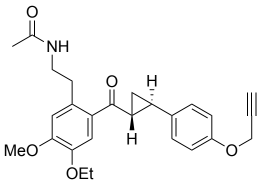

7

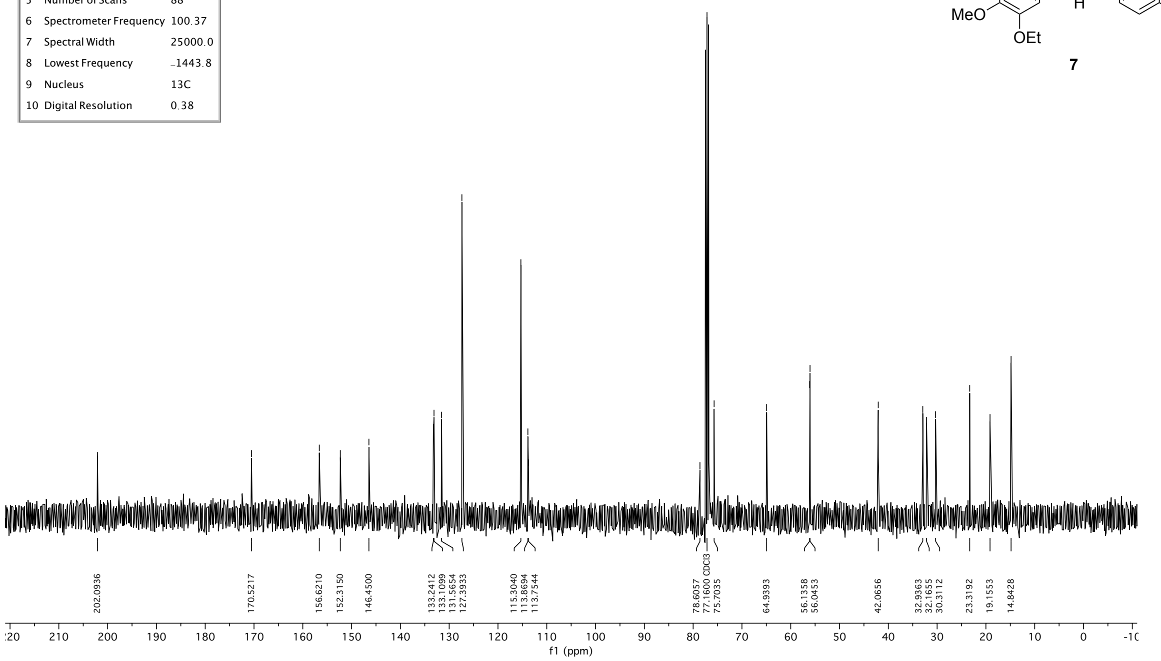

^{13}C NMR spectrum of compound **7**

	Parameter	Value
1	Comment	27
2	Instrument	ECA 600SL
3	Solvent	DMSO-D6
4	Temperature	25.1
5	Number of Scans	16
6	Spectrometer Frequency	594.17
7	Spectral Width	8912.7
8	Lowest Frequency	-1463.5
9	Nucleus	1H
10	Digital Resolution	0.08

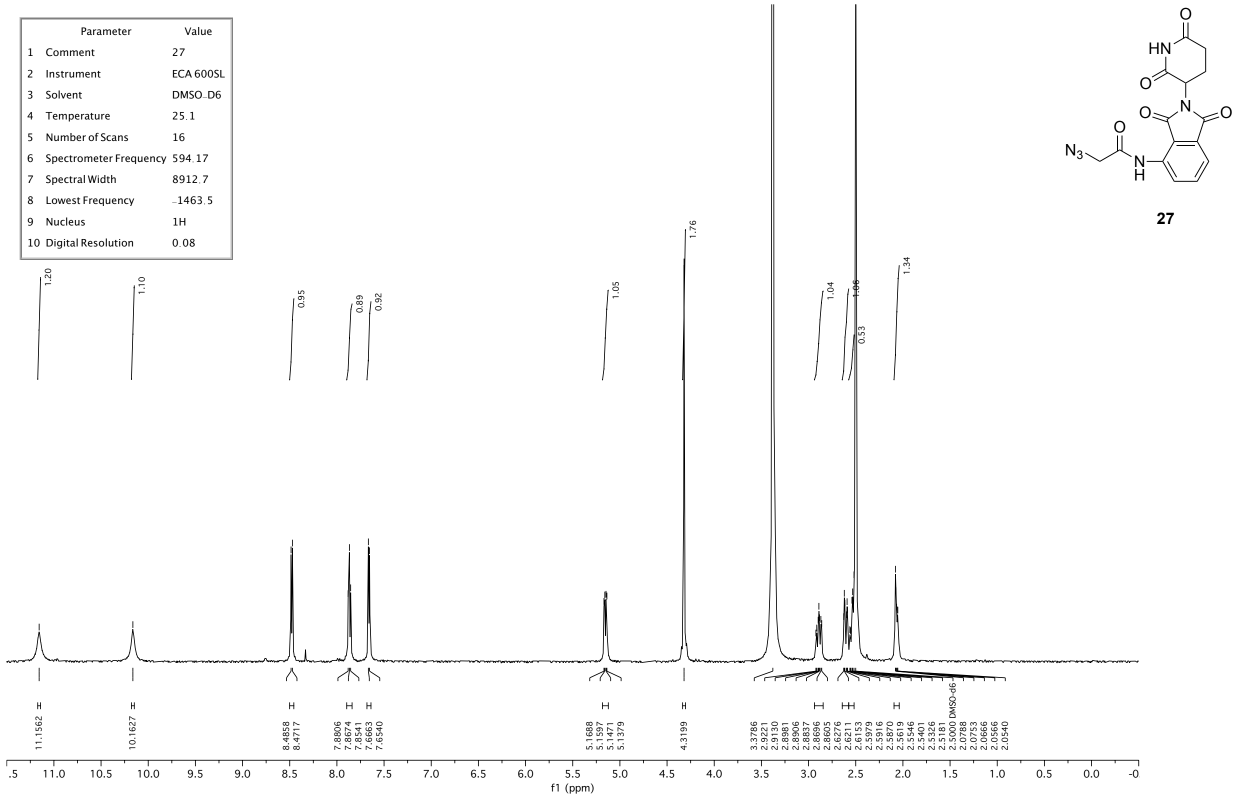

^{1}H NMR spectrum of compound **27**

	Parameter	Value
1	Comment	27
2	Instrument	ECA 600SL
3	Solvent	DMSO-D6
4	Temperature	26.2
5	Number of Scans	40000
6	Spectrometer Frequency	149.40
7	Spectral Width	37594.0
8	Lowest Frequency	-3934.5
9	Nucleus	13C
10	Digital Resolution	0.72

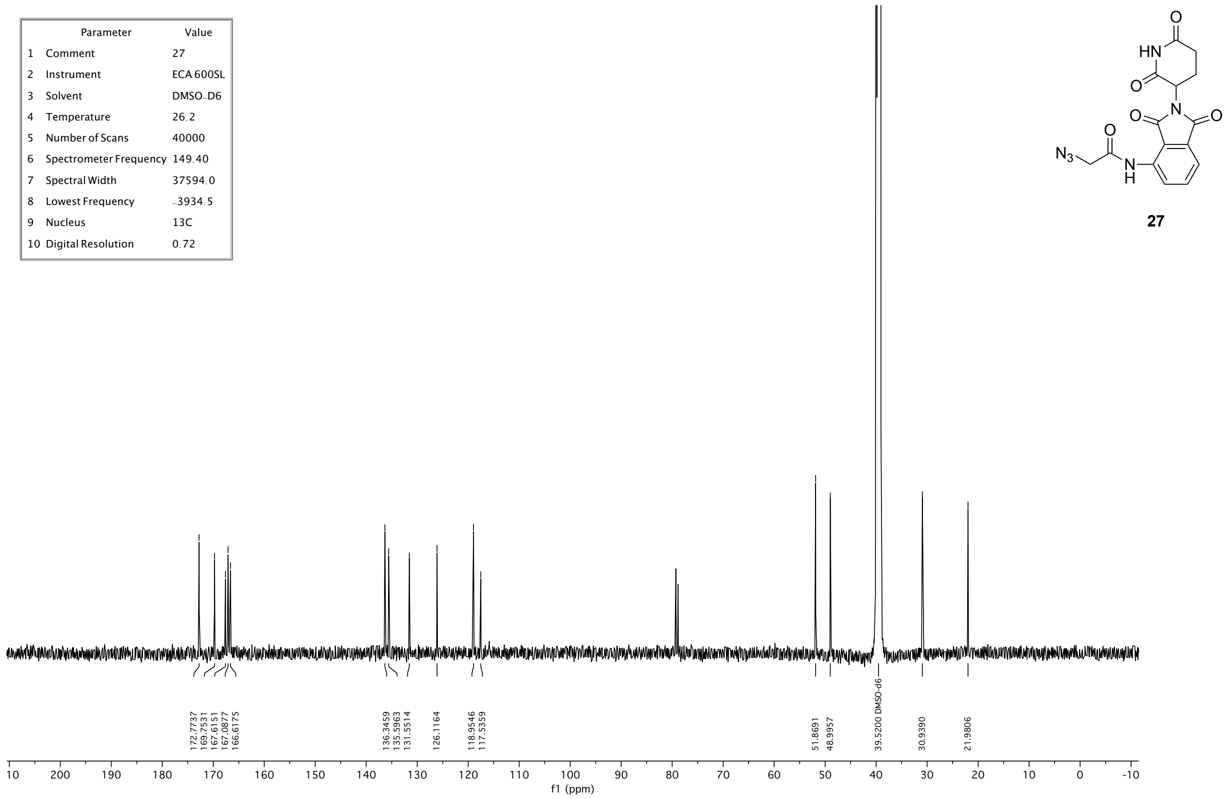

^{13}C NMR spectrum of compound **27**

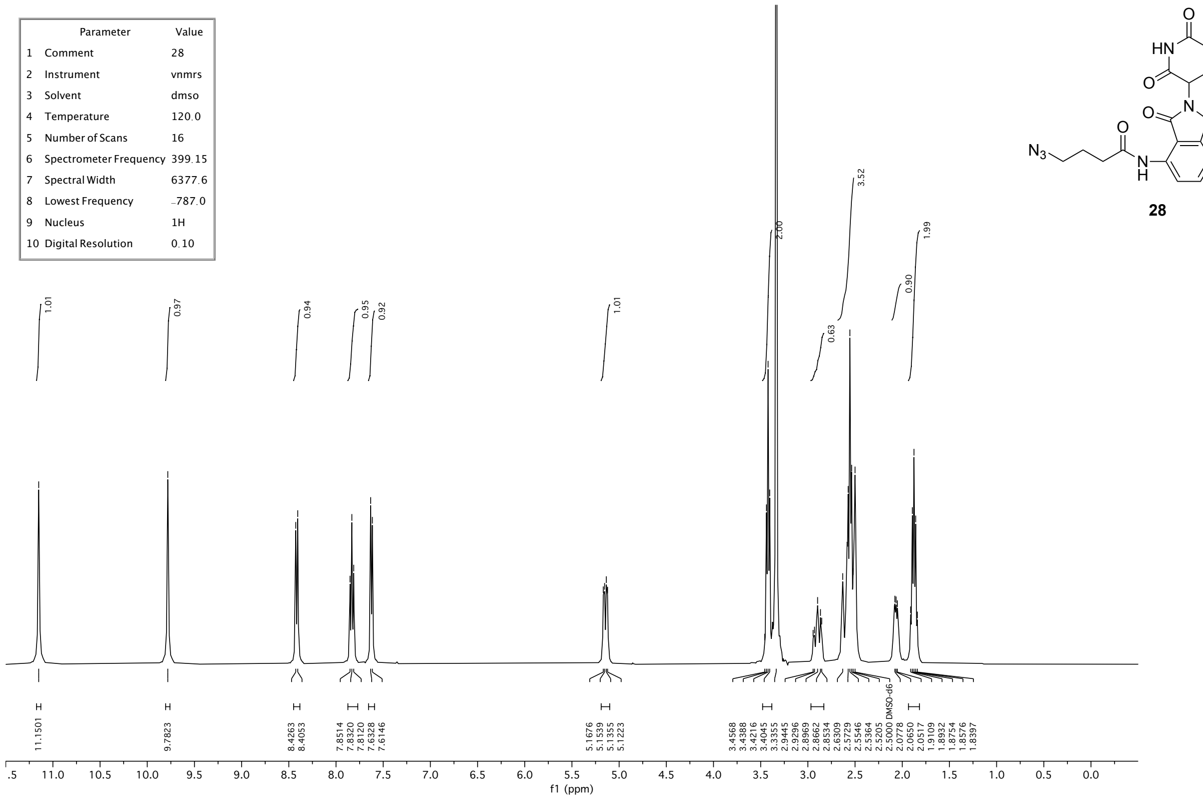

^{1}H NMR spectrum of compound **28**

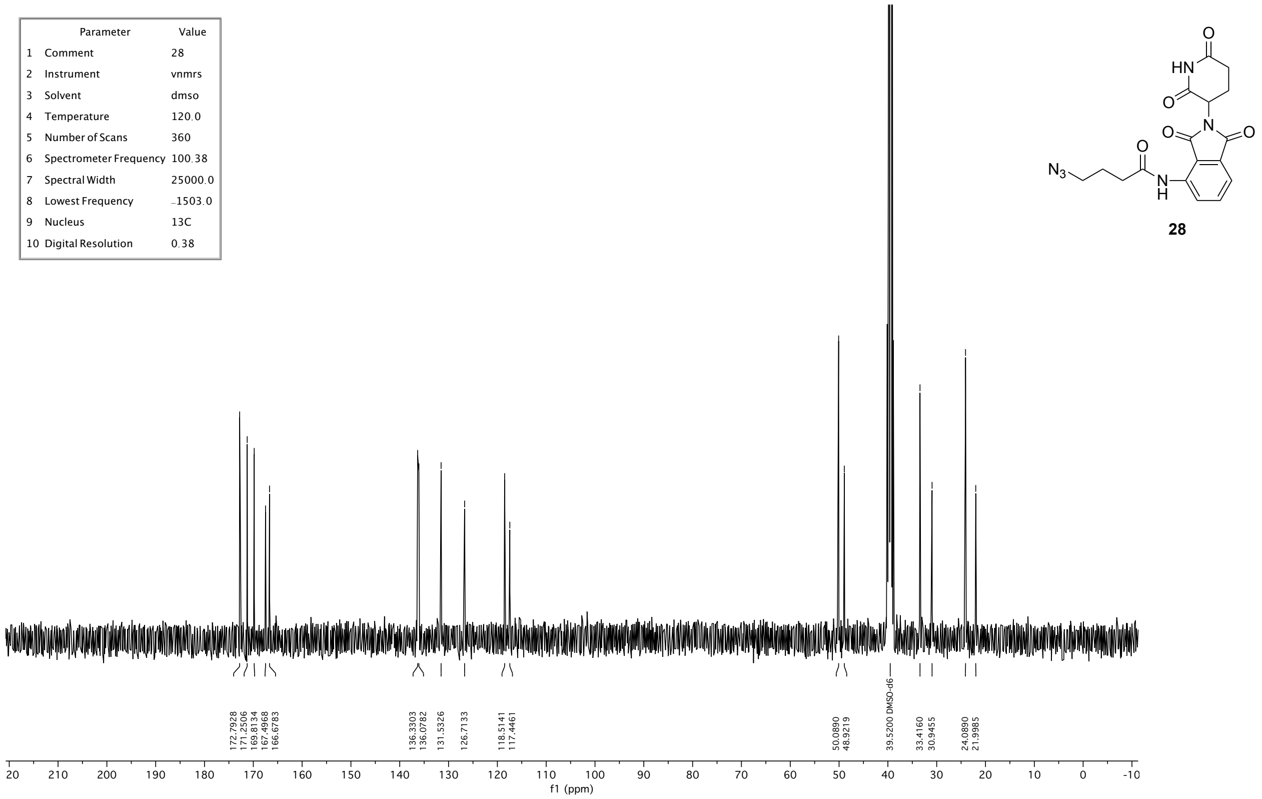

^{13}C NMR spectrum of compound **28**

	Parameter	Value
1	Comment	29
2	Instrument	vnmrs
3	Solvent	cdcl3
4	Temperature	8.8
5	Number of Scans	16
6	Spectrometer Frequency	399.14
7	Spectral Width	6377.6
8	Lowest Frequency	-781.9
9	Nucleus	1H
10	Digital Resolution	0.10

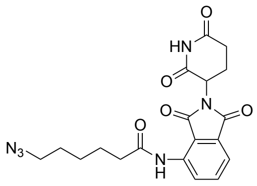

29

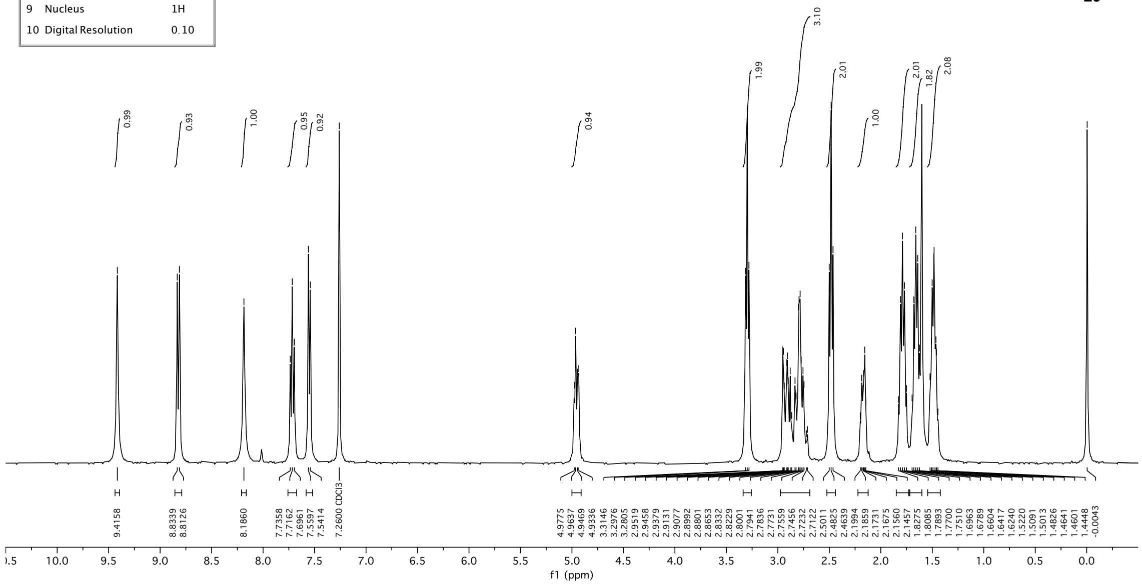

^{1}H NMR spectrum of compound **29**

	Parameter	Value
1	Comment	29
2	Instrument	vnmrs
3	Solvent	cdcl3
4	Temperature	8.8
5	Number of Scans	256
6	Spectrometer Frequency	100.37
7	Spectral Width	25000.0
8	Lowest Frequency	-1442.2
9	Nucleus	13C
10	Digital Resolution	0.38

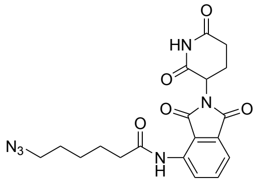

29

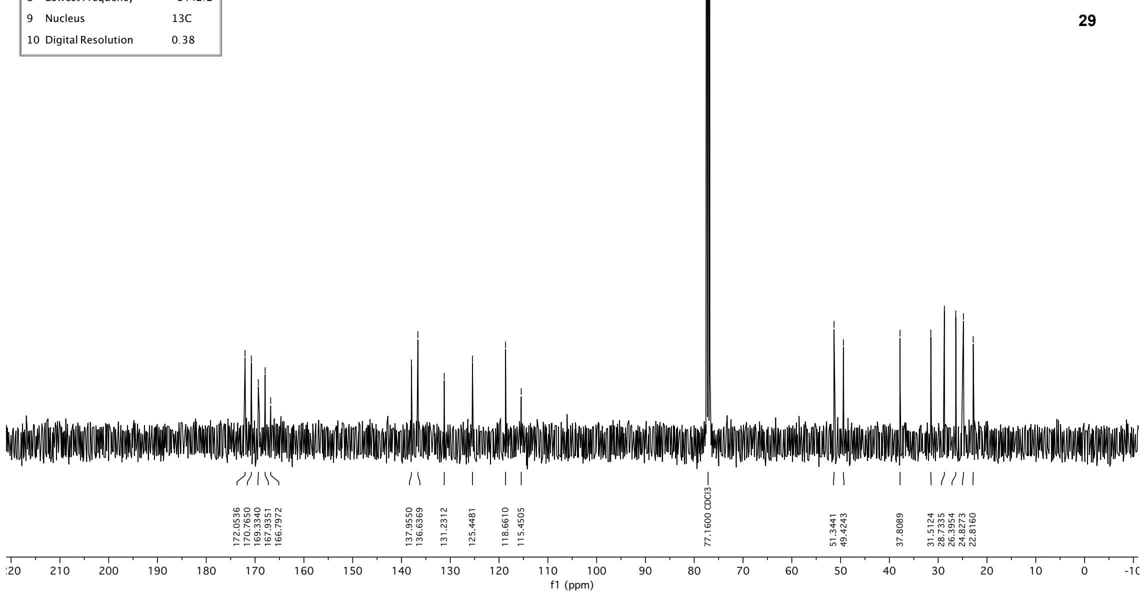

^{13}C NMR spectrum of compound **29**

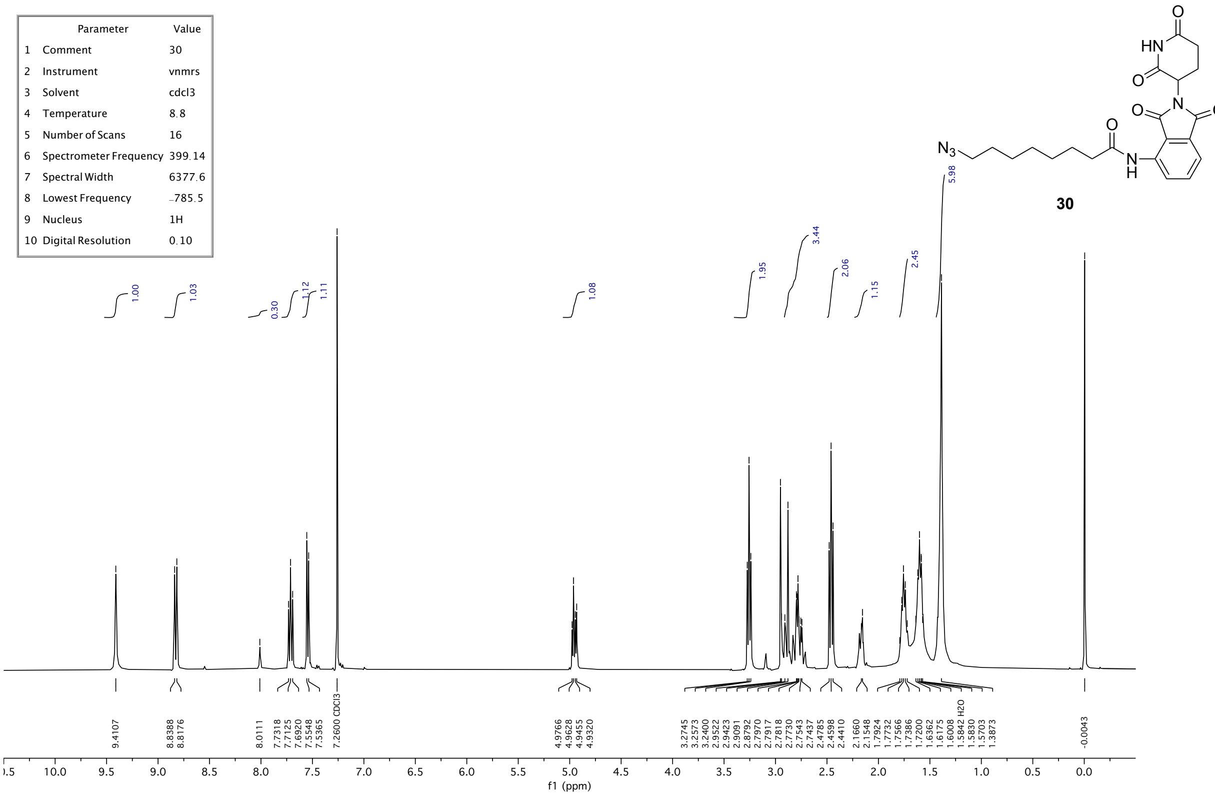

^{1}H NMR spectrum of compound **30**

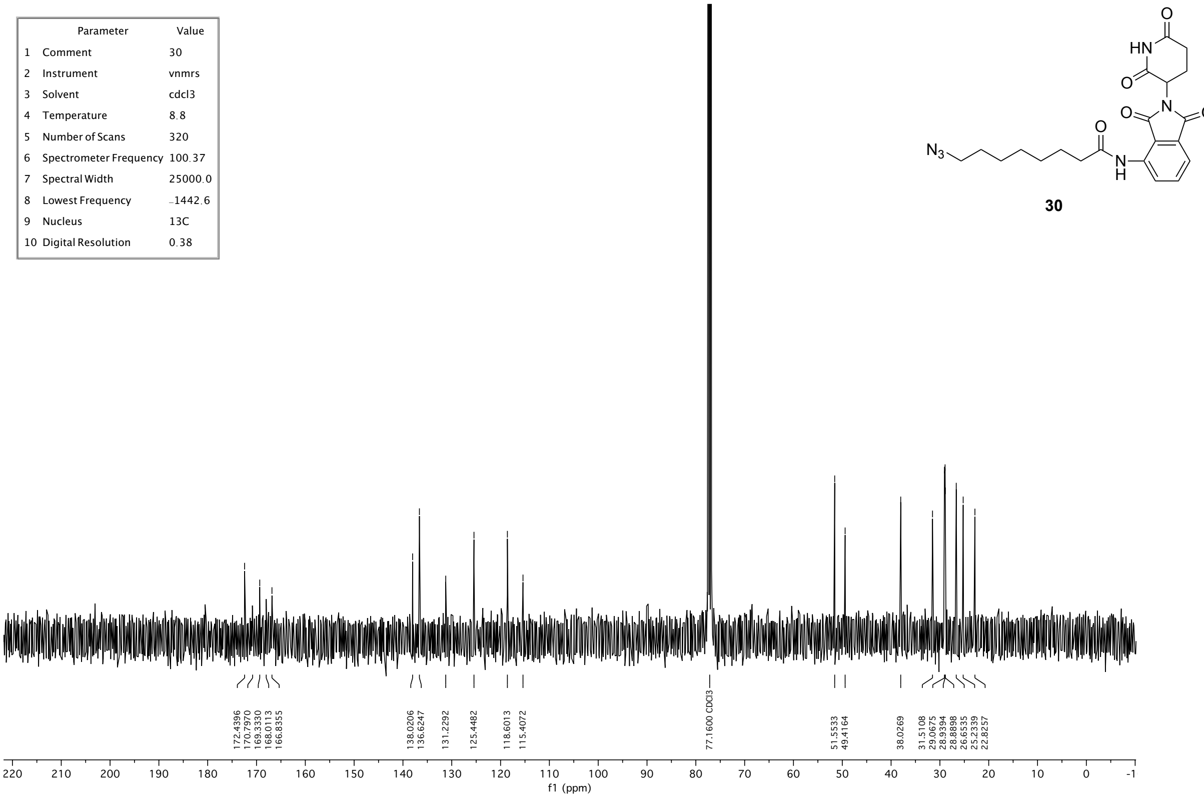

^{13}C NMR spectrum of compound **30**

	Parameter	Value
1	Comment	31
2	Instrument	vnmrs
3	Solvent	cdcl3
4	Temperature	8.8
5	Number of Scans	16
6	Spectrometer Frequency	399.14
7	Spectral Width	6377.6
8	Lowest Frequency	-785.6
9	Nucleus	1H
10	Digital Resolution	0.10

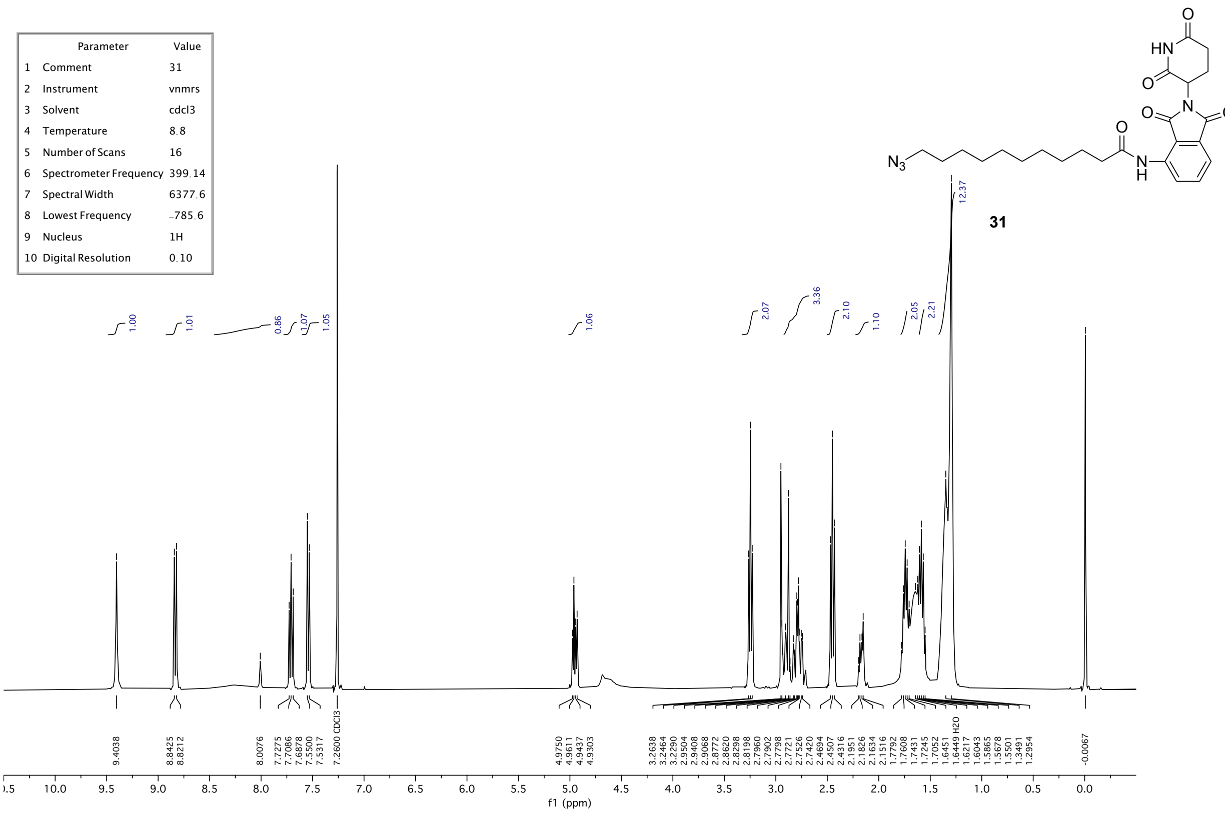

^{1}H NMR spectrum of compound **31**

	Parameter	Value
1	Comment	31
2	Instrument	vnmrs
3	Solvent	cdcl3
4	Temperature	8.8
5	Number of Scans	176
6	Spectrometer Frequency	100.37
7	Spectral Width	25000.0
8	Lowest Frequency	-1442.6
9	Nucleus	13C
10	Digital Resolution	0.38

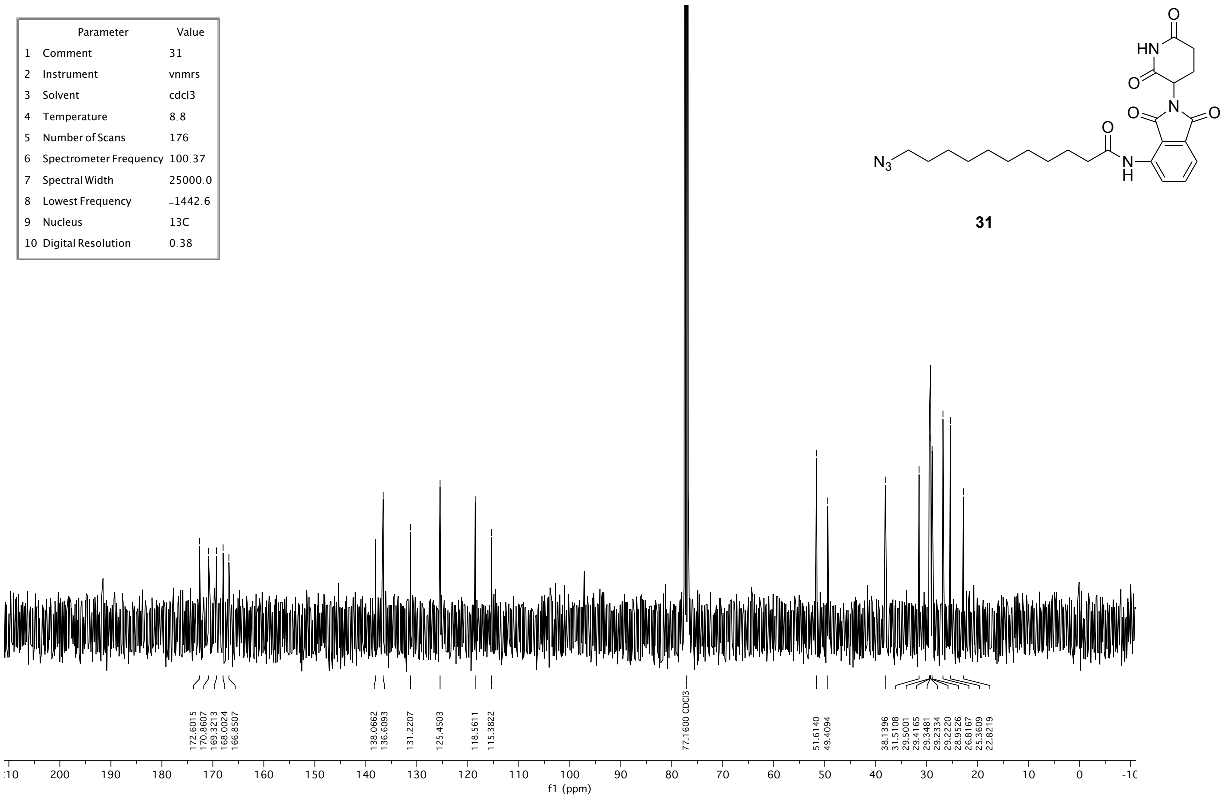

^{13}C NMR spectrum of compound **31**

	Parameter	Value
1	Comment	32
2	Instrument	vnmrs
3	Solvent	cdcl3
4	Temperature	8.8
5	Number of Scans	16
6	Spectrometer Frequency	399.15
7	Spectral Width	6377.6
8	Lowest Frequency	-793.5
9	Nucleus	1H
10	Digital Resolution	0.10

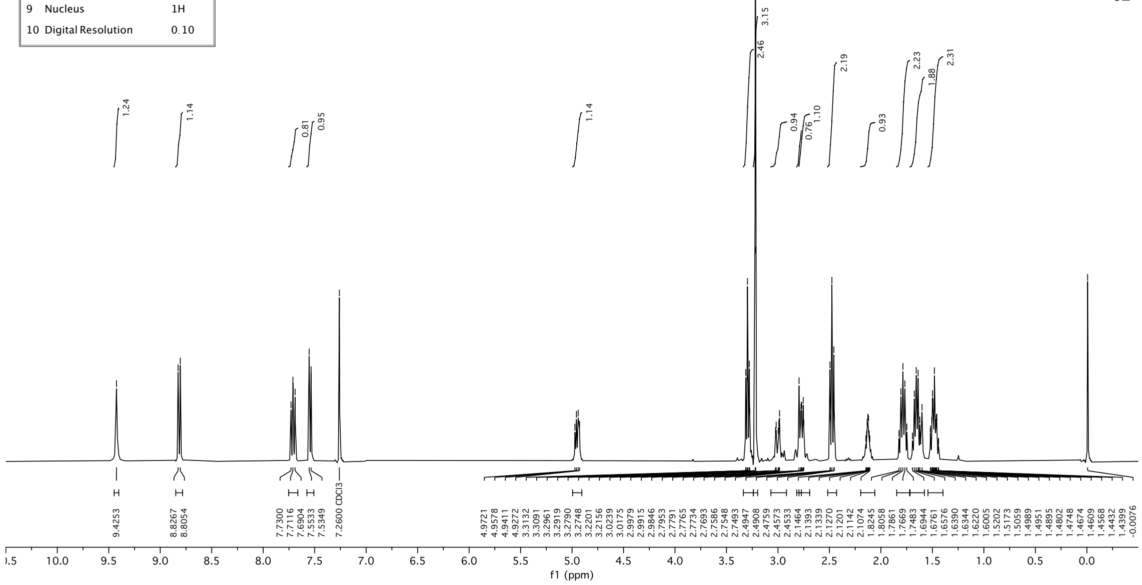

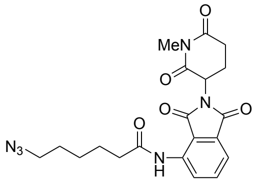

32

^{1}H NMR spectrum of compound **32**

	Parameter	Value
1	Comment	32
2	Instrument	vnmrs
3	Solvent	cdcl3
4	Temperature	8.8
5	Number of Scans	160
6	Spectrometer Frequency	100.38
7	Spectral Width	25000.0
8	Lowest Frequency	-1445.4
9	Nucleus	13C
10	Digital Resolution	0.38

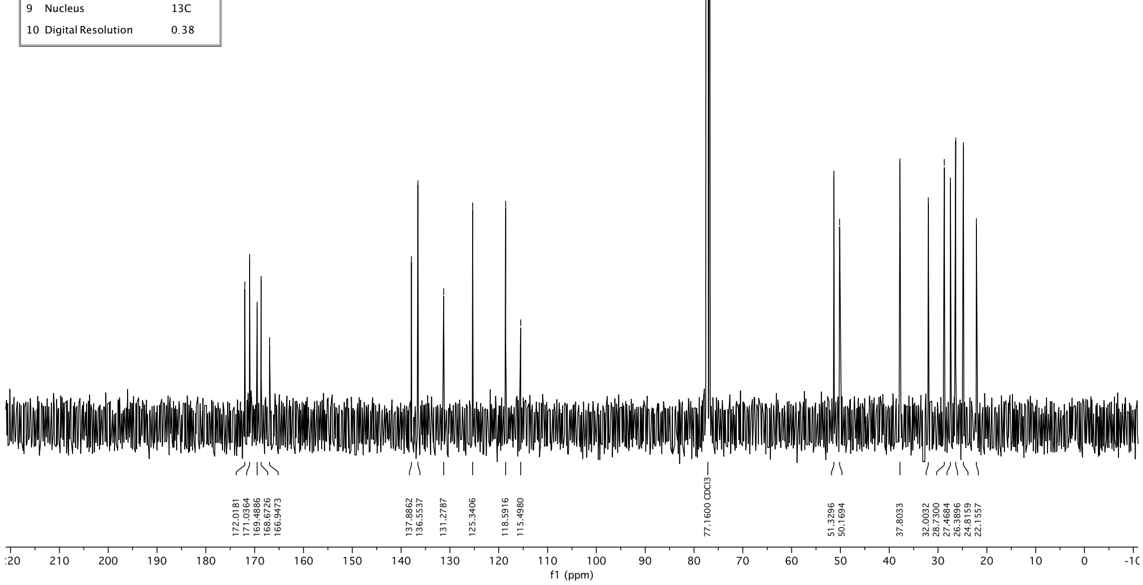

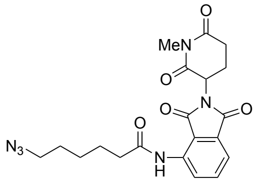

32

^{13}C NMR spectrum of compound **32**

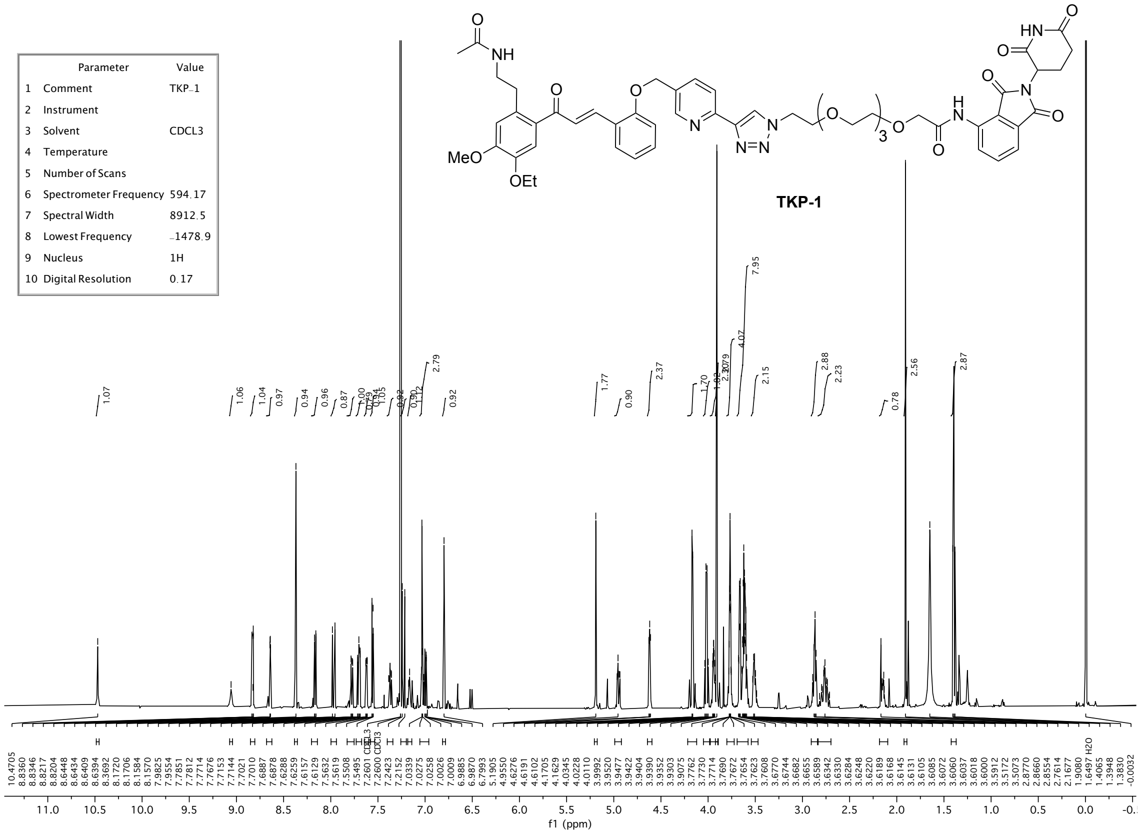

^{1}H NMR spectrum of **TKP-1**

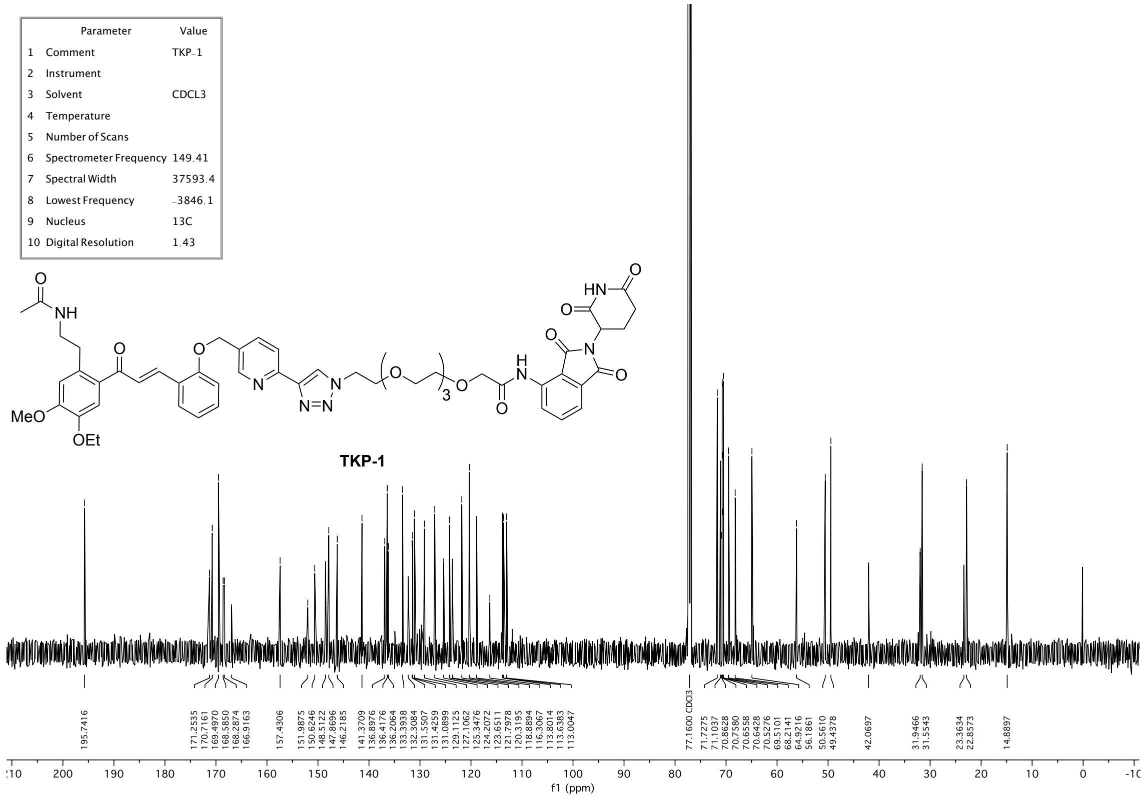

^{13}C NMR spectrum of **TKP-1**

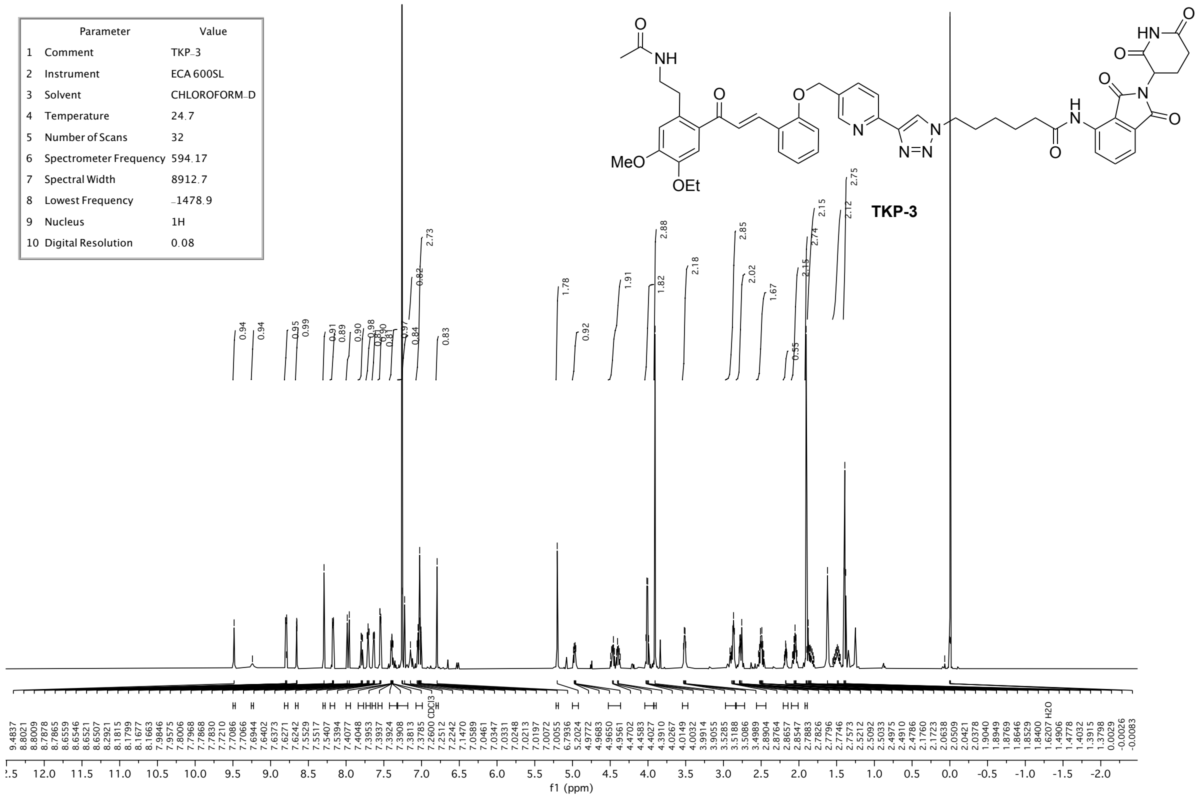

^{1}H NMR spectrum of **TKP-3**

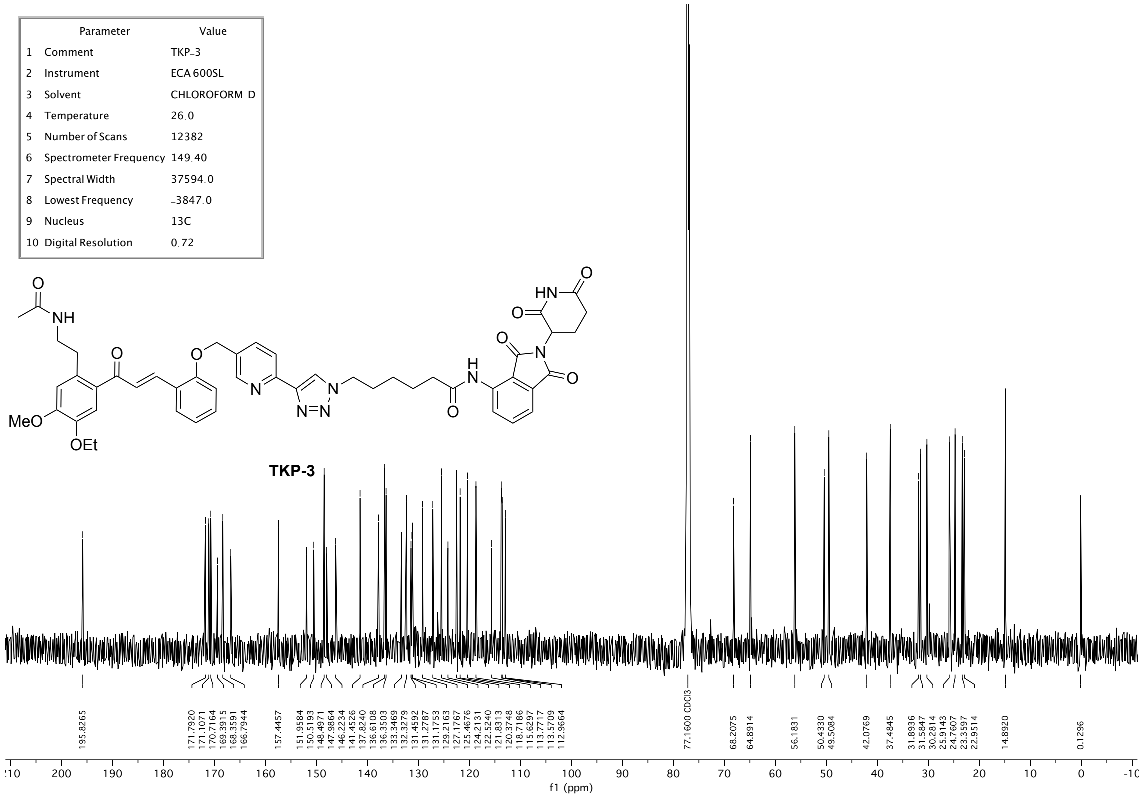

^{13}C NMR spectrum of **TKP-3**

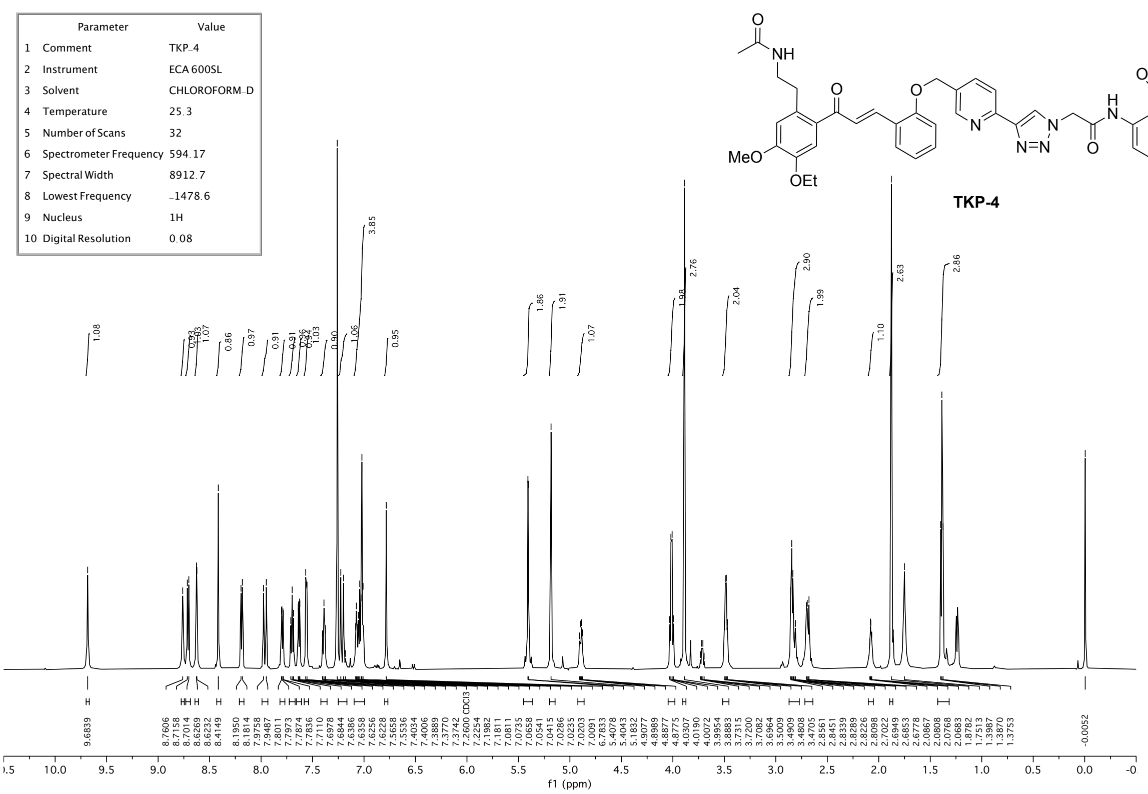

^1H NMR spectrum of **TKP-4**

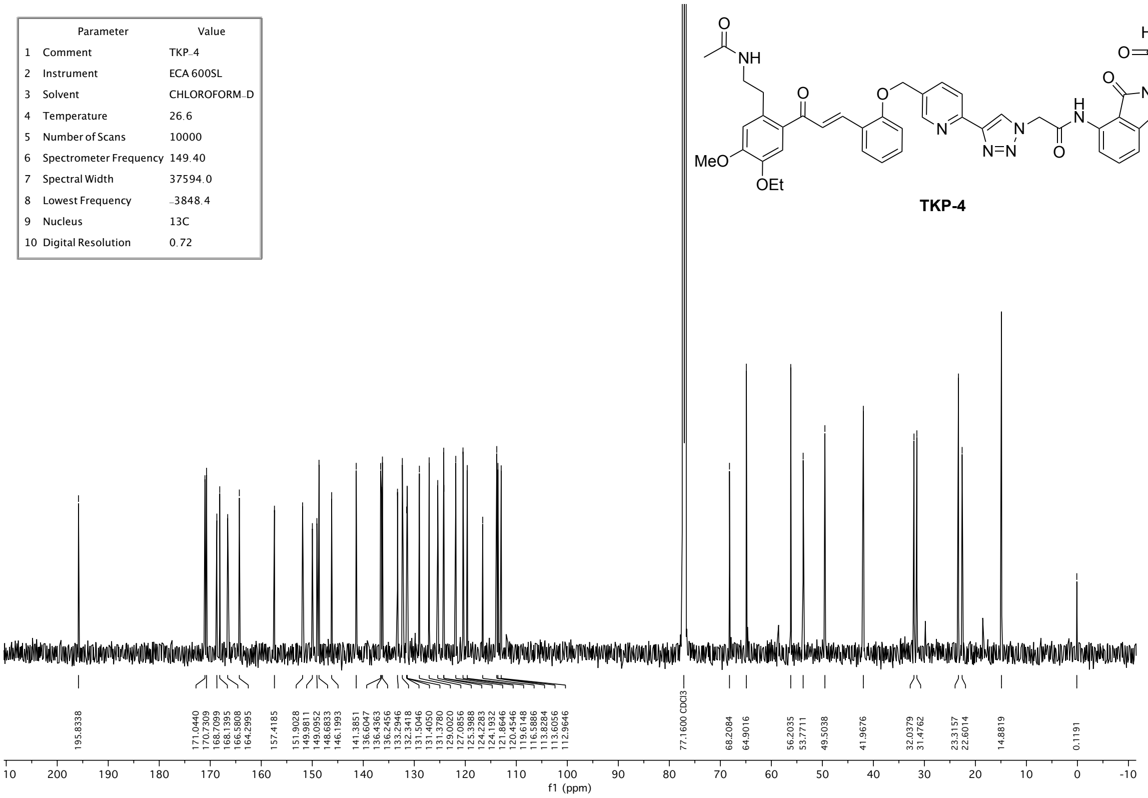

^{13}C NMR spectrum of **TKP-4**

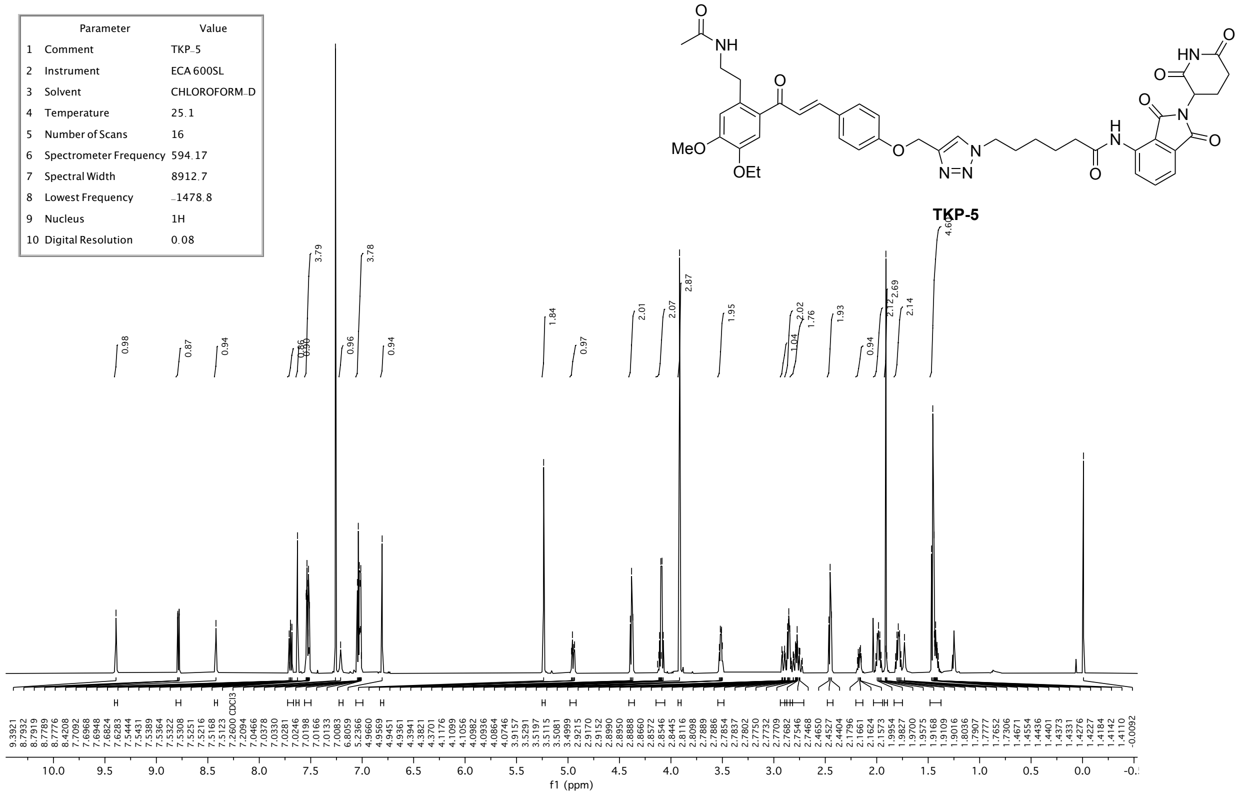

^{1}H NMR spectrum of **TKP-5**

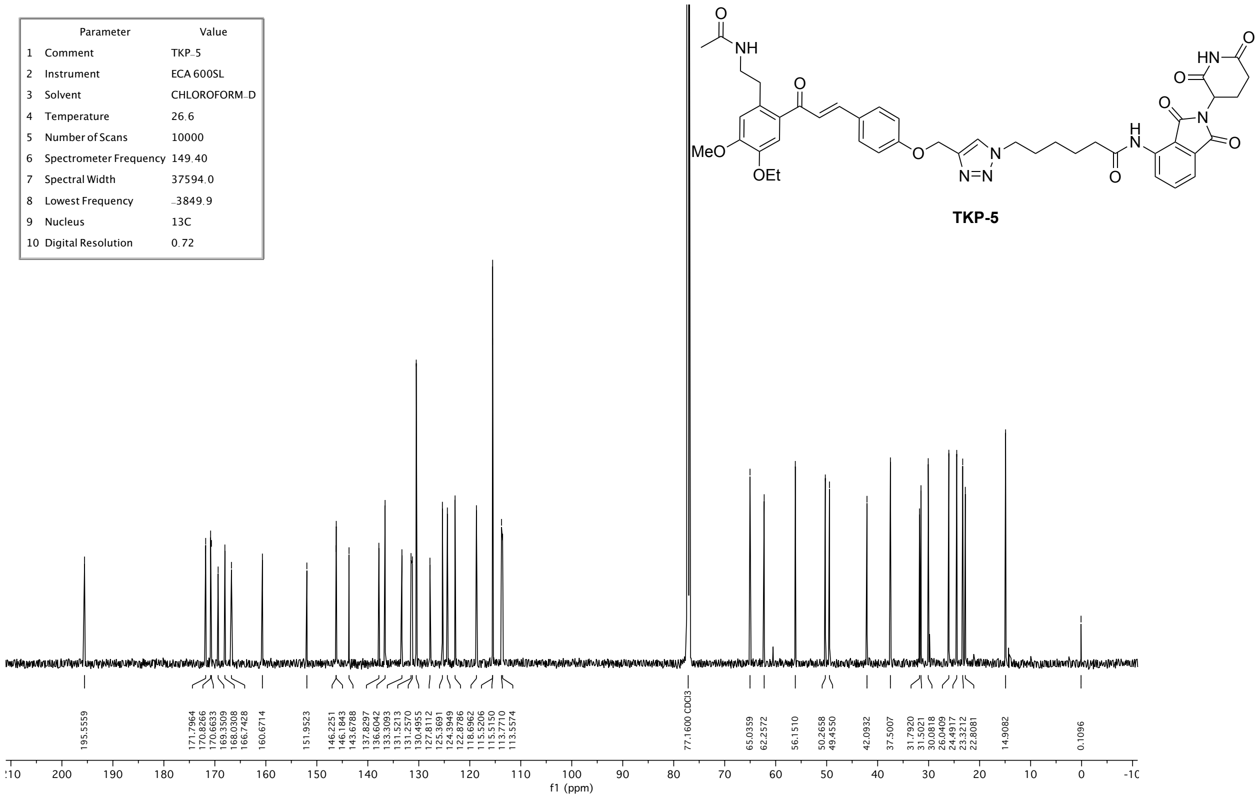

^{13}C NMR spectrum of **TKP-5**

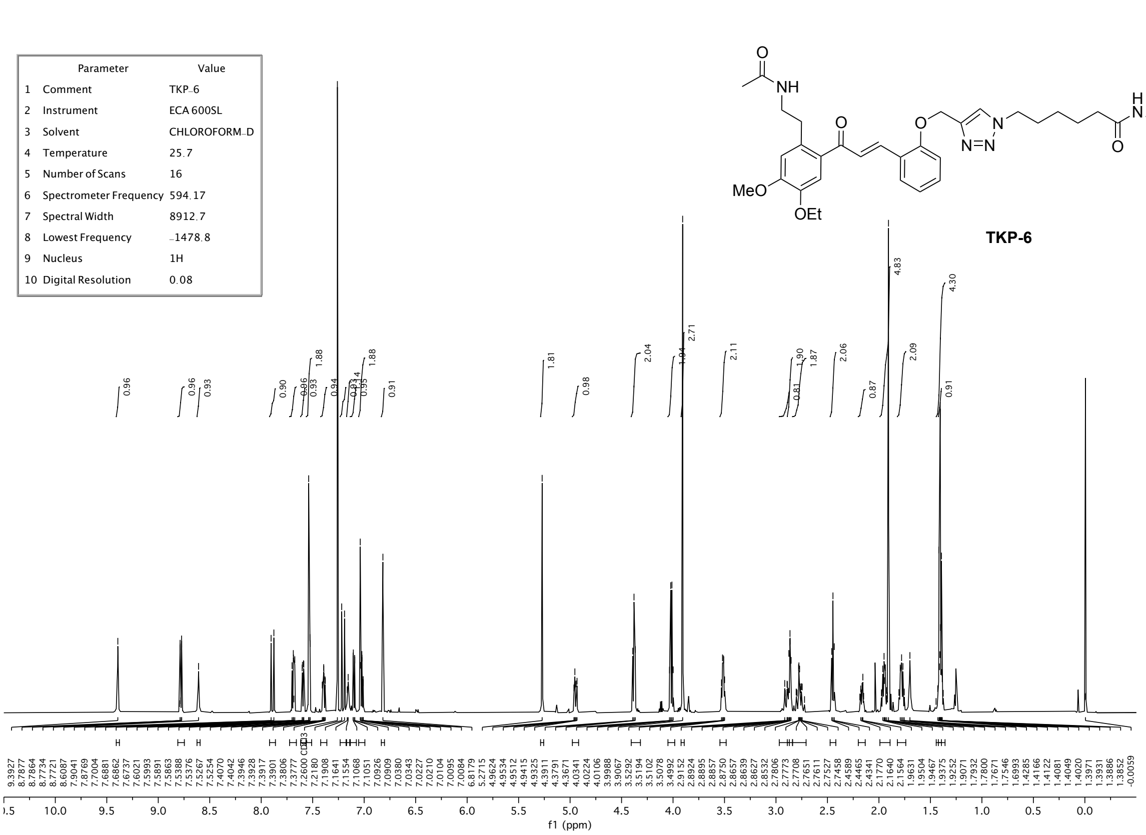

^{1}H NMR spectrum of **TKP-6**

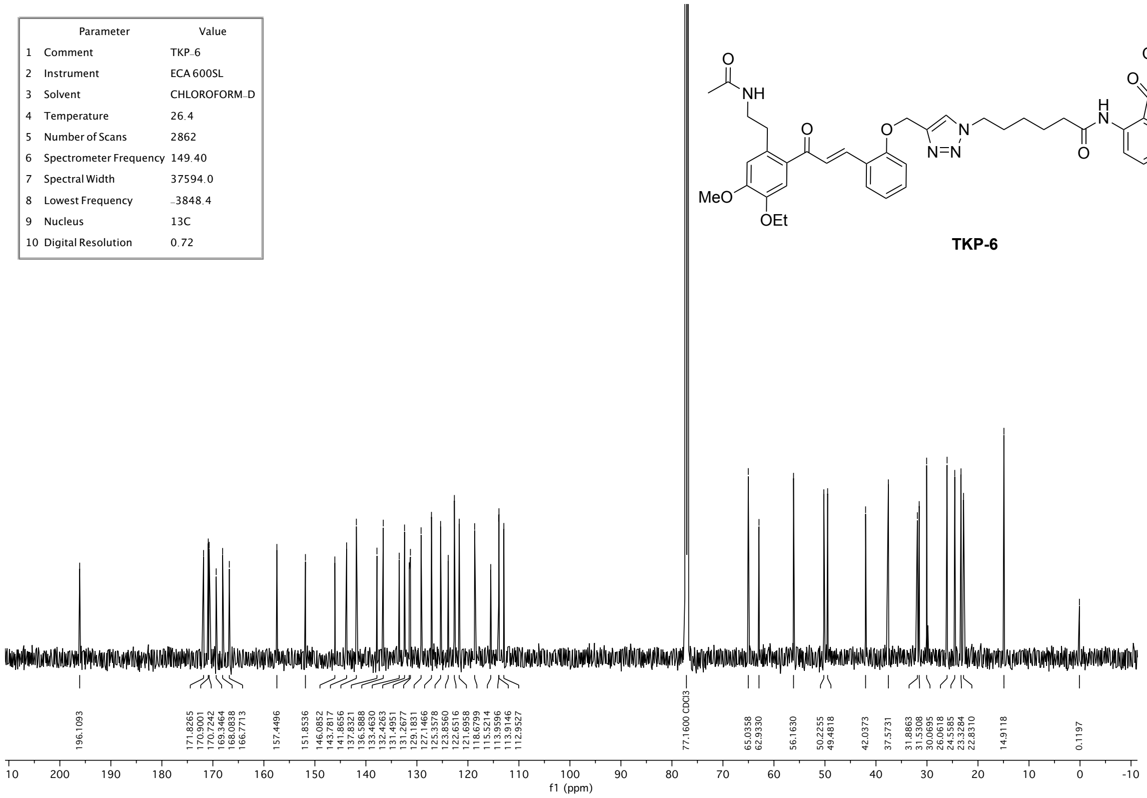

^{13}C NMR spectrum of **TKP-6**

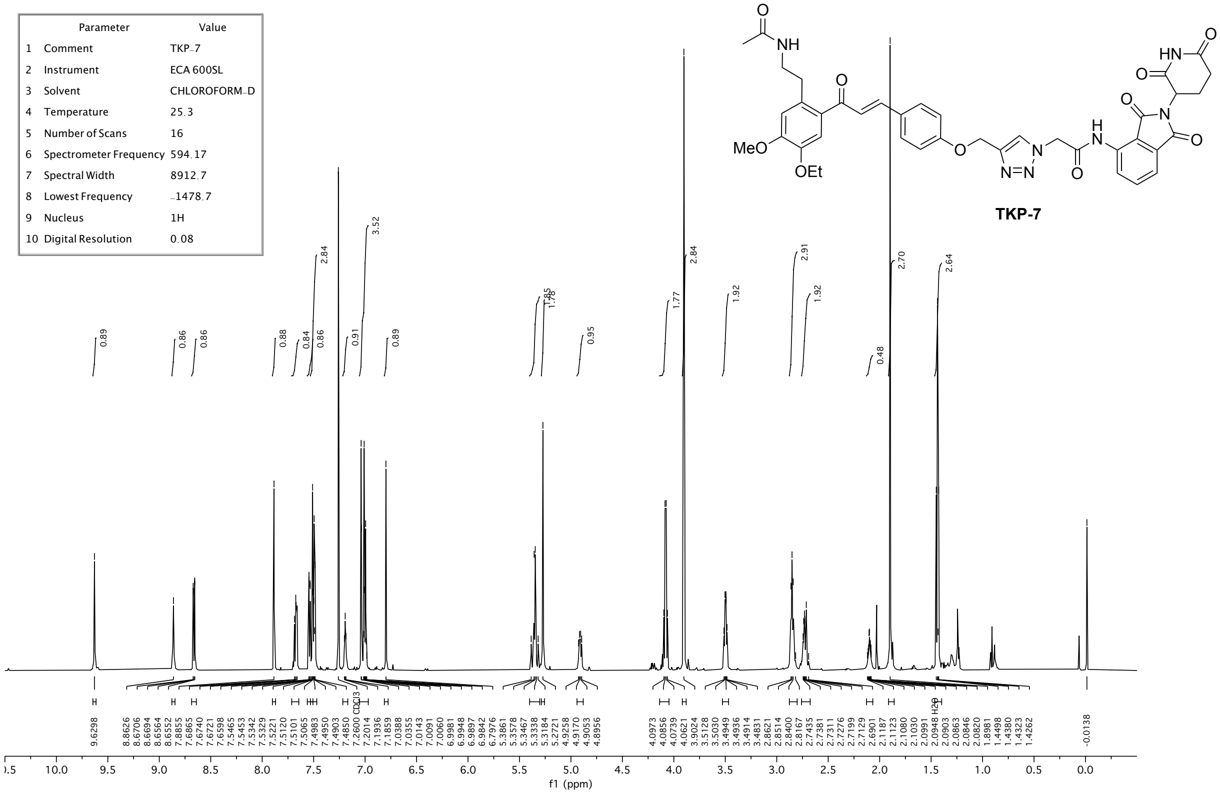

	Parameter	Value
1	Comment	TKP_7
2	Instrument	ECA 600SL
3	Solvent	CHLOROFORM_D
4	Temperature	25.3
5	Number of Scans	16
6	Spectrometer Frequency	594.17
7	Spectral Width	8912.7
8	Lowest Frequency	_1478.7
9	Nucleus	1H
10	Digital Resolution	0.08

^{1}H NMR spectrum of **TKP-7**

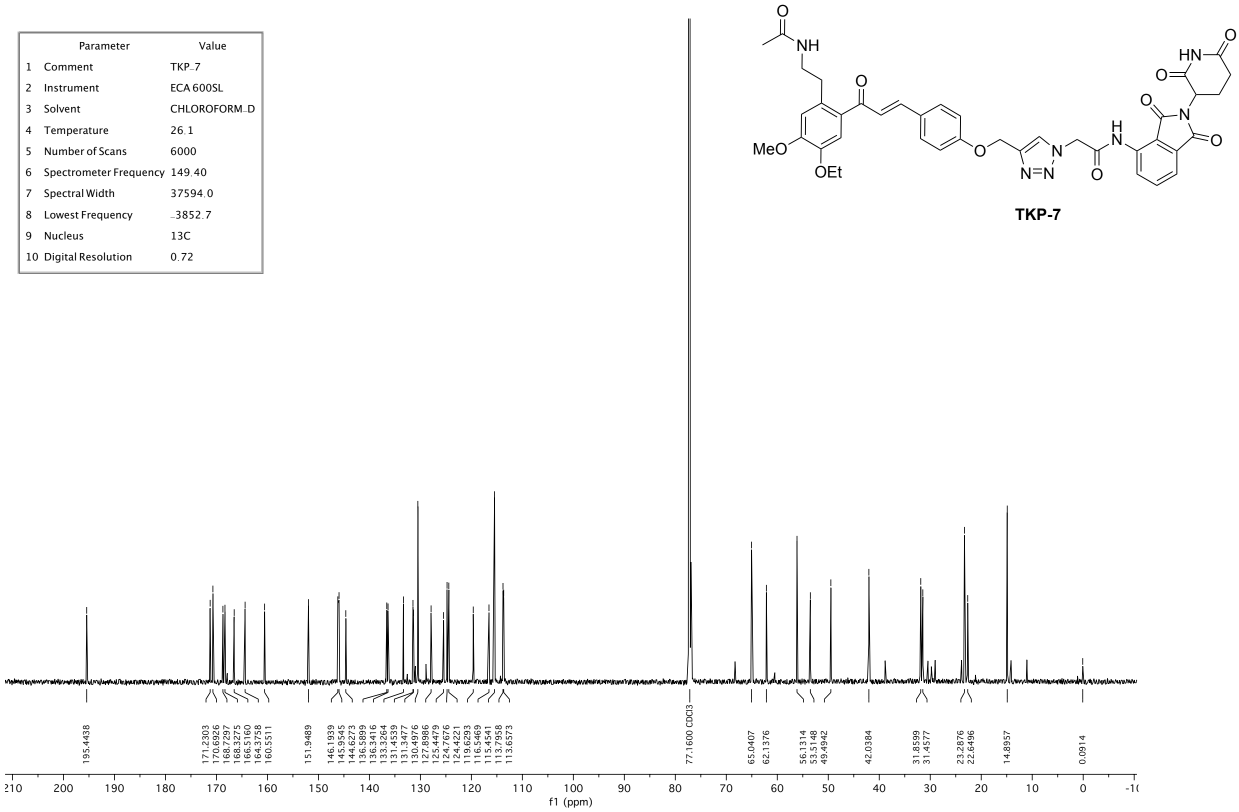

	Parameter	Value
1	Comment	TKP_7
2	Instrument	ECA 600SL
3	Solvent	CHLOROFORM_D
4	Temperature	26.1
5	Number of Scans	6000
6	Spectrometer Frequency	149.40
7	Spectral Width	37594.0
8	Lowest Frequency	_3852.7
9	Nucleus	13C
10	Digital Resolution	0.72

^{13}C NMR spectrum of **TKP-7**

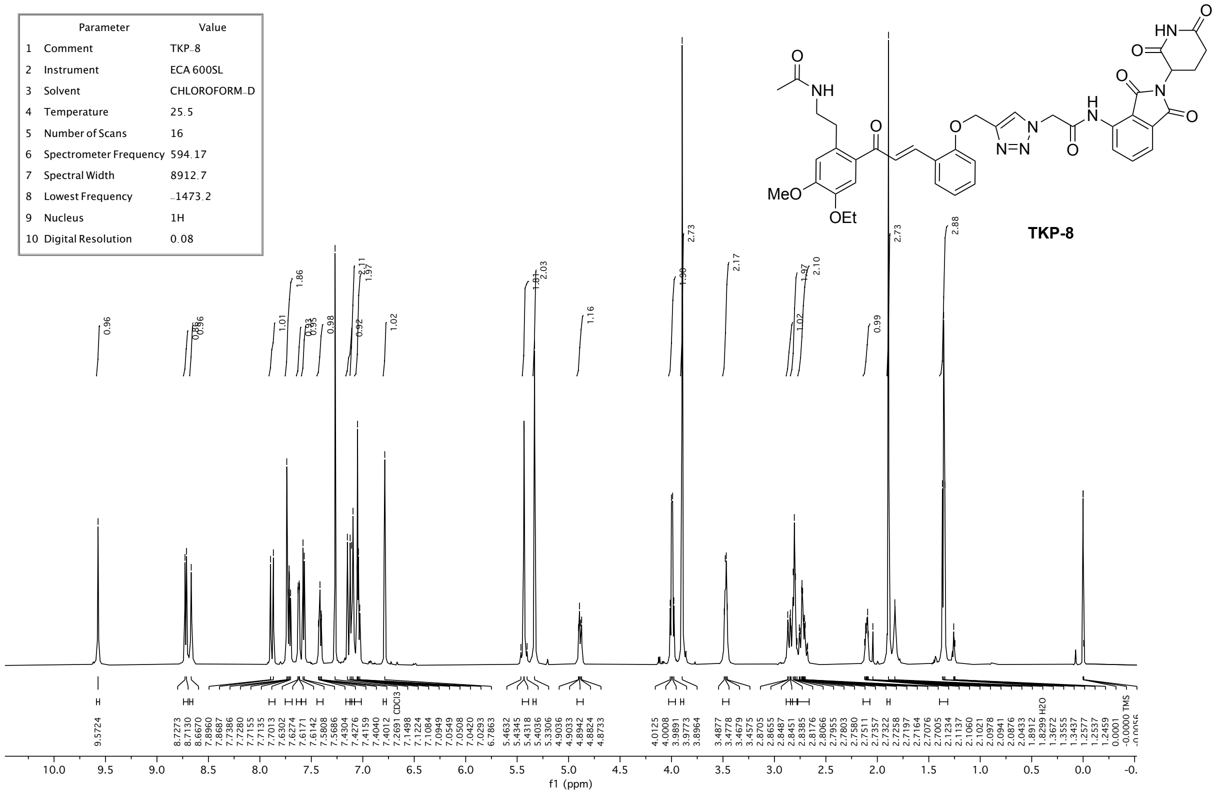

^{1}H NMR spectrum of **TKP-8**

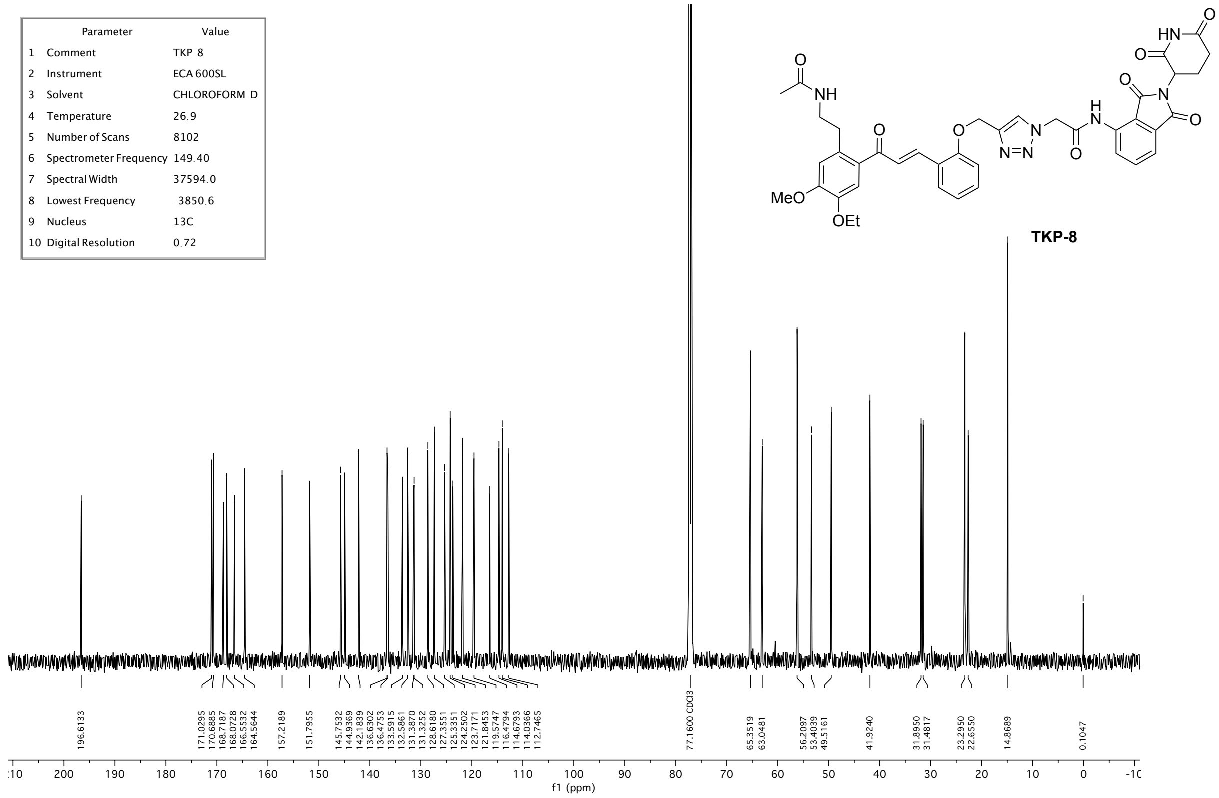

^{13}C NMR spectrum of **TKP-8**

	Parameter	Value
1	Comment	TKP_9
2	Instrument	ECA 600SL
3	Solvent	CHLOROFORM_D
4	Temperature	25.2
5	Number of Scans	16
6	Spectrometer Frequency	594.17
7	Spectral Width	8912.7
8	Lowest Frequency	_1478.8
9	Nucleus	1H
10	Digital Resolution	0.08

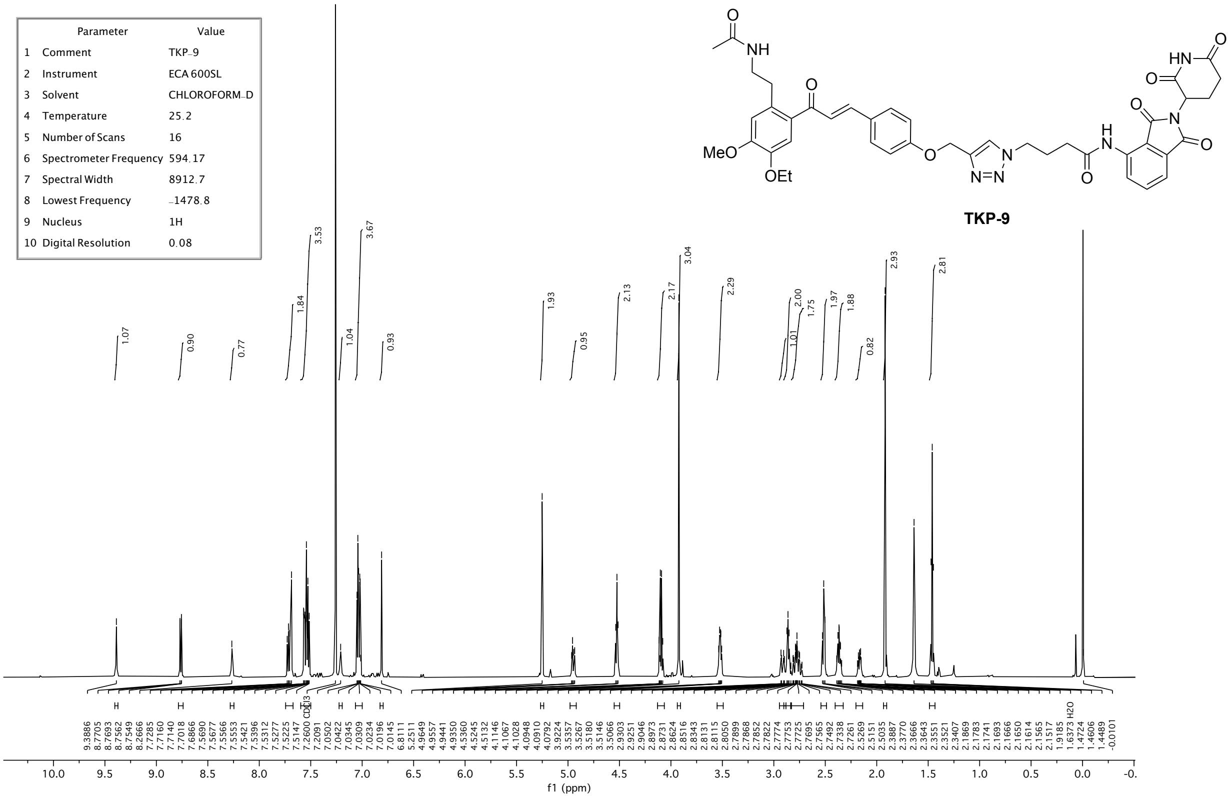

^{1}H NMR spectrum of **TKP-9**

	Parameter	Value
1	Comment	TKP_9
2	Instrument	ECA 600SL
3	Solvent	CHLOROFORM_D
4	Temperature	26.3
5	Number of Scans	8000
6	Spectrometer Frequency	149.40
7	Spectral Width	37594.0
8	Lowest Frequency	_3847.7
9	Nucleus	13C
10	Digital Resolution	0.72

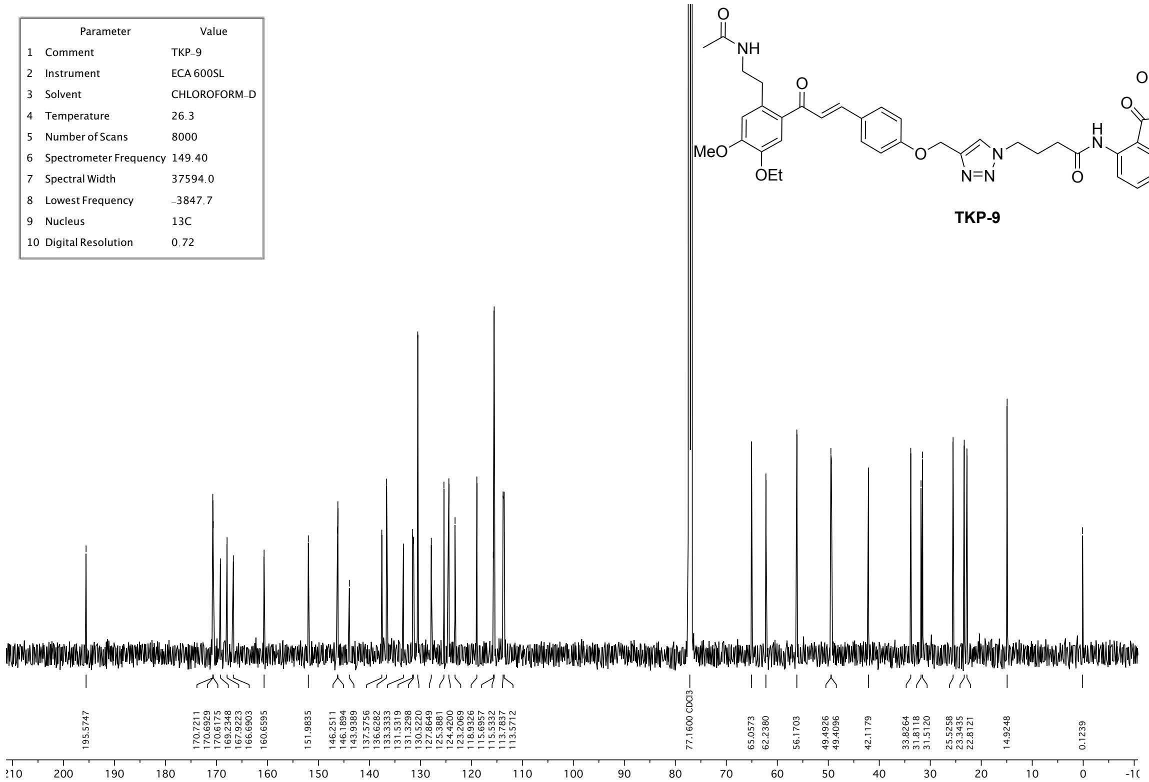

^{13}C NMR spectrum of **TKP-9**

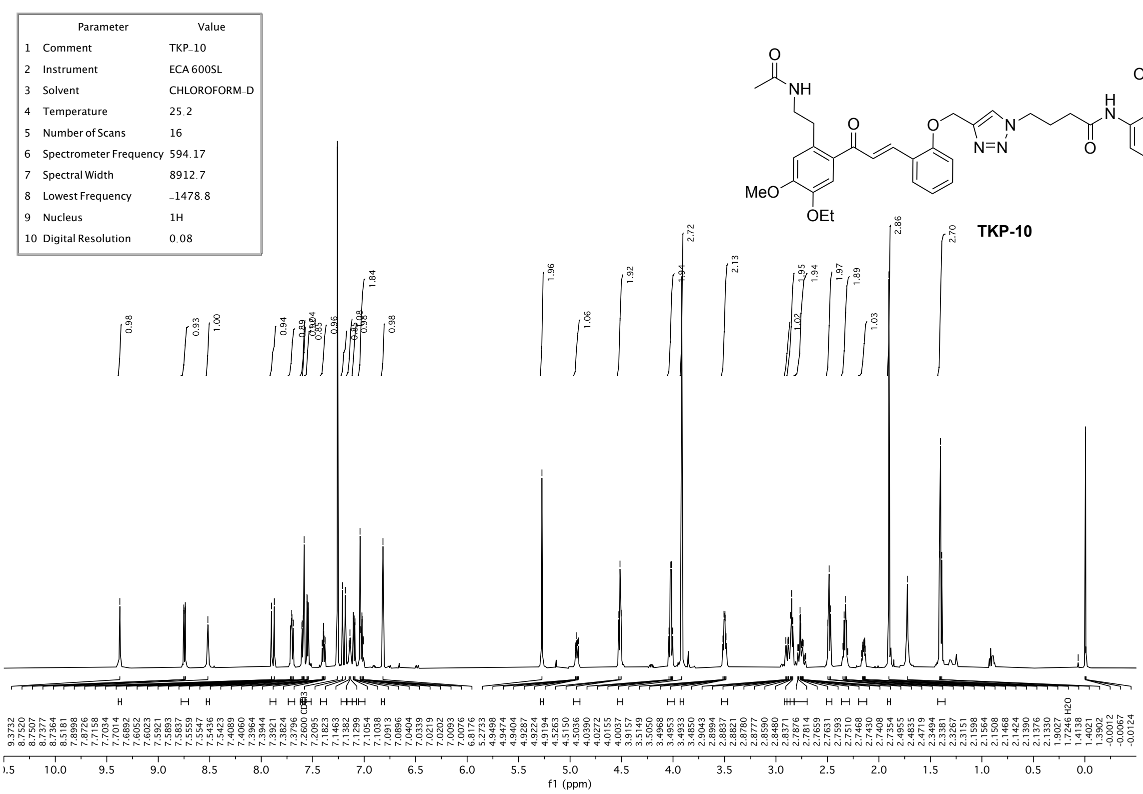

	Parameter	Value
1	Comment	TKP_10
2	Instrument	ECA 600SL
3	Solvent	CHLOROFORM_D
4	Temperature	25.2
5	Number of Scans	16
6	Spectrometer Frequency	594.17
7	Spectral Width	8912.7
8	Lowest Frequency	_1478.8
9	Nucleus	1H
10	Digital Resolution	0.08

^{1}H NMR spectrum of **TKP-10**

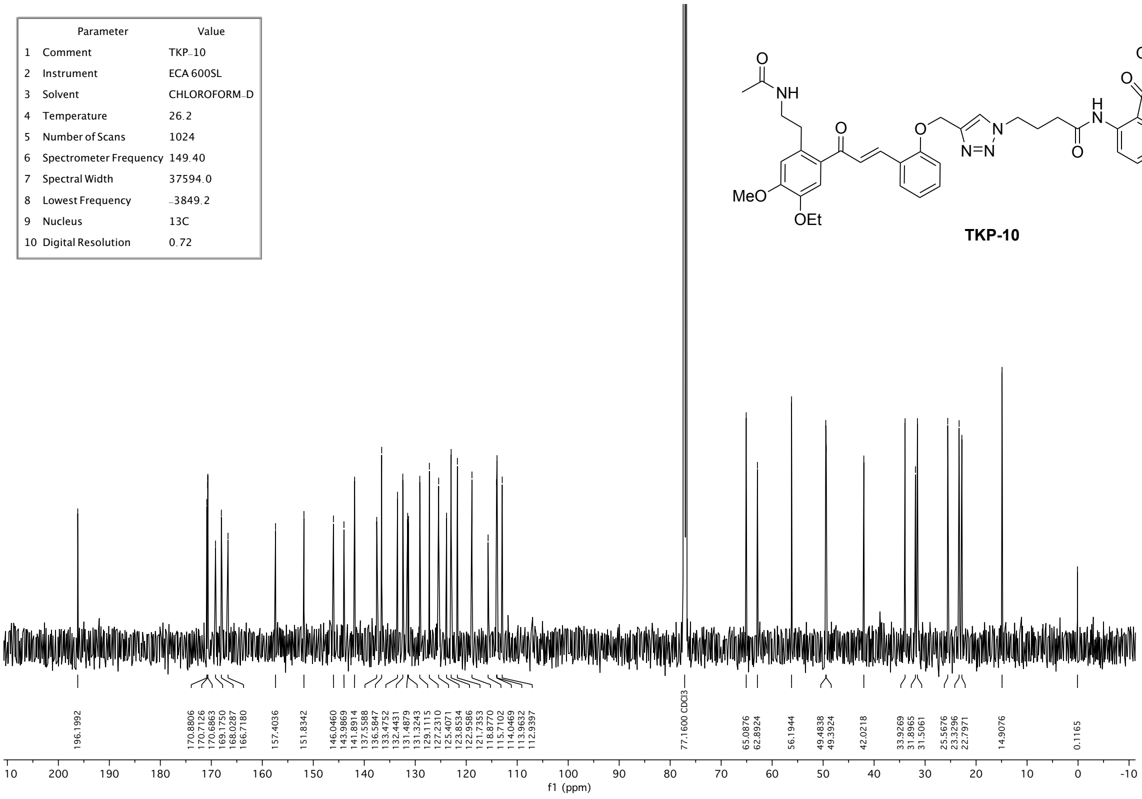

	Parameter	Value
1	Comment	TKP_10
2	Instrument	ECA 600SL
3	Solvent	CHLOROFORM_D
4	Temperature	26.2
5	Number of Scans	1024
6	Spectrometer Frequency	149.40
7	Spectral Width	37594.0
8	Lowest Frequency	_3849.2
9	Nucleus	13C
10	Digital Resolution	0.72

^{13}C NMR spectrum of **TKP-10**

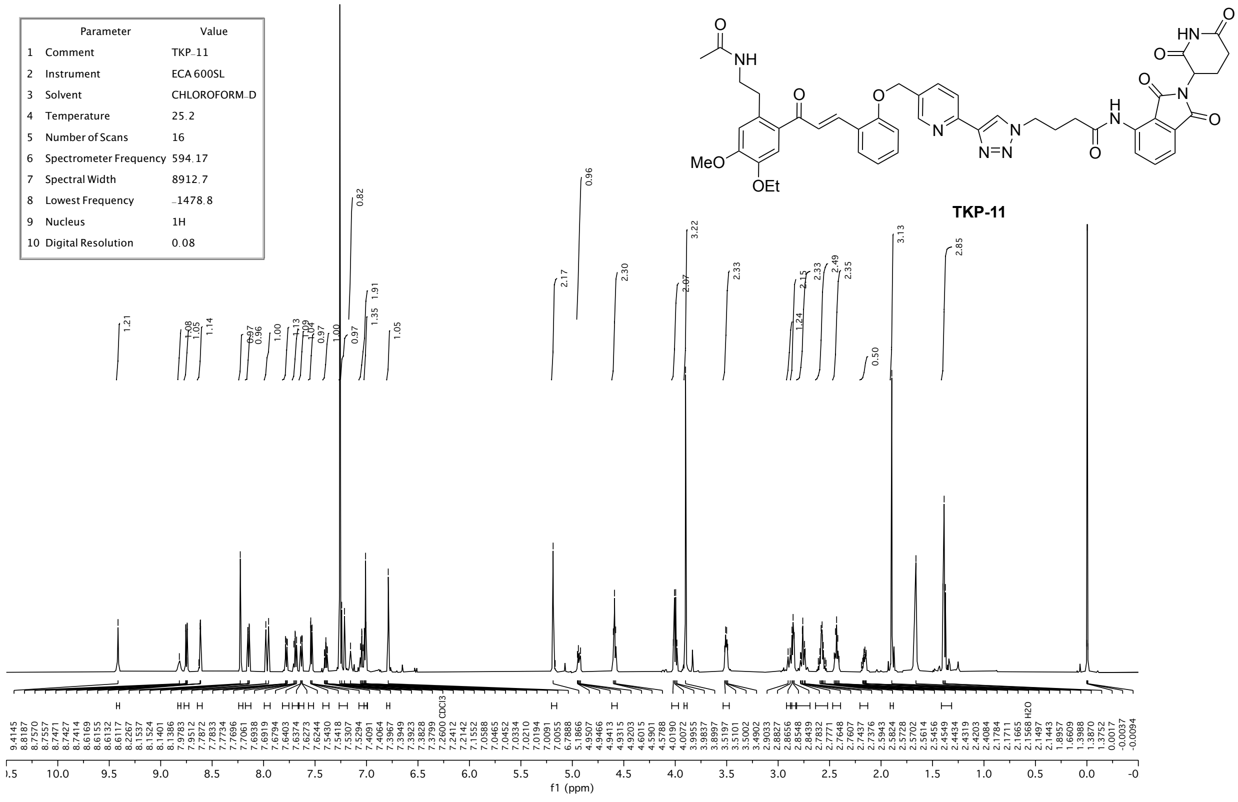

^{1}H NMR spectrum of **TKP-11**

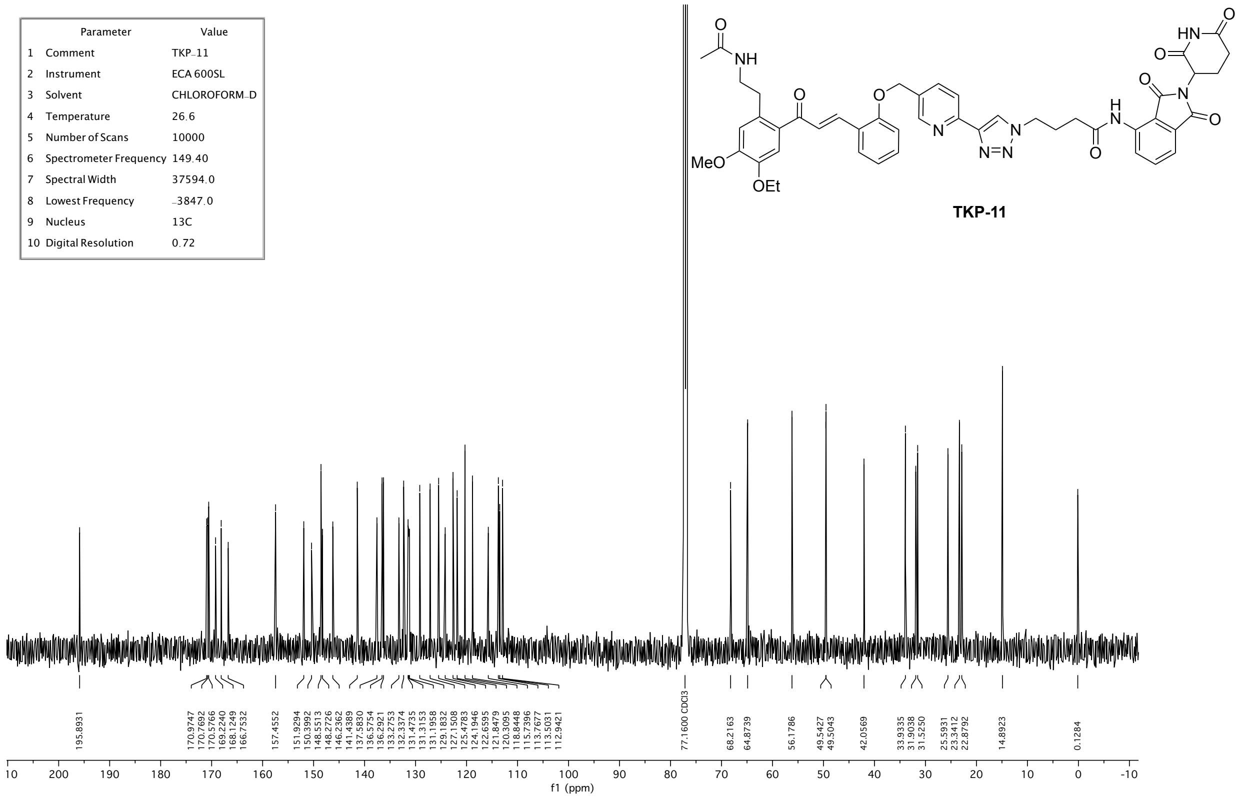

^{13}C NMR spectrum of **TKP-11**

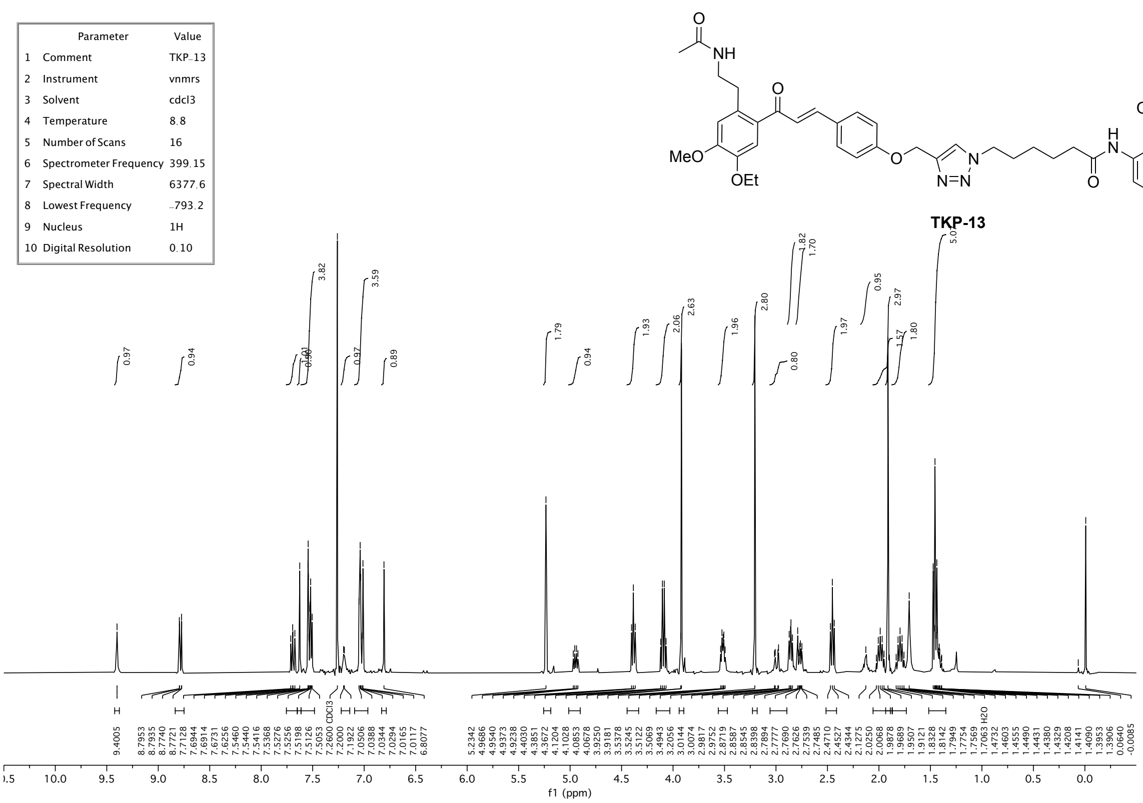

^{1}H NMR spectrum of **TKP-13**

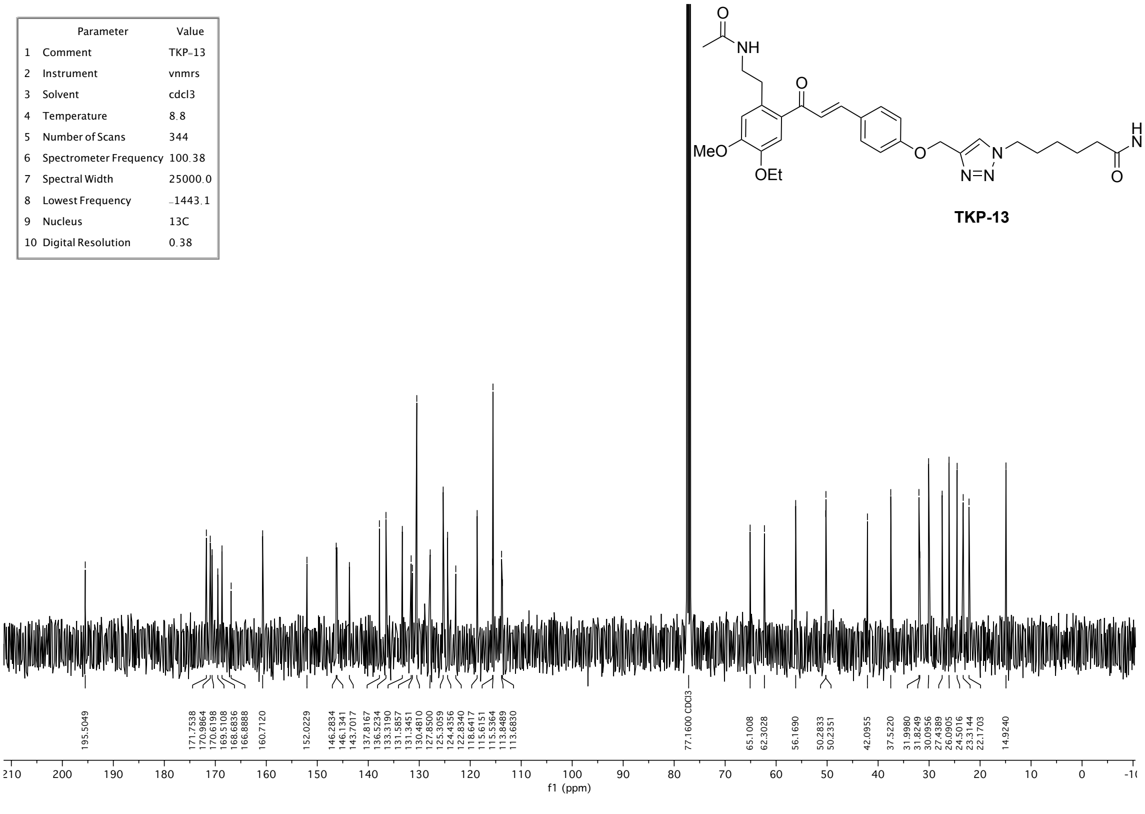

^{13}C NMR spectrum of **TKP-13**

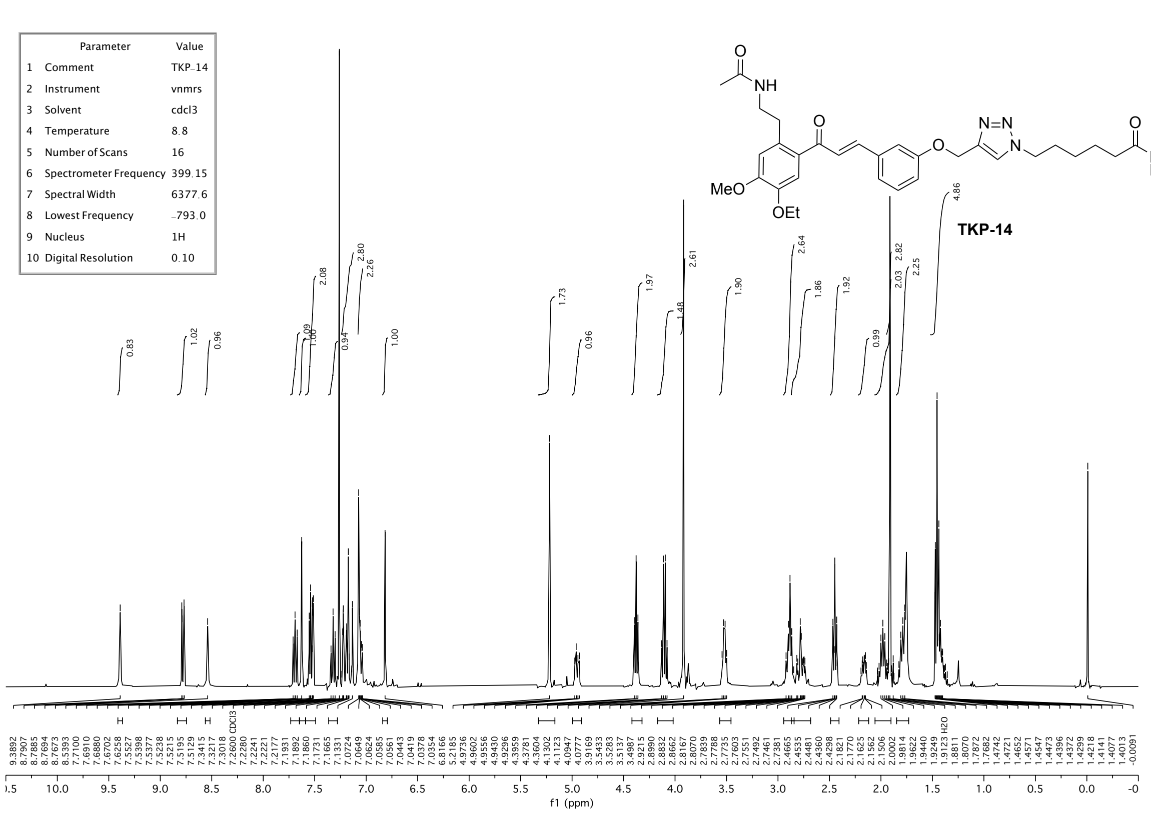

^{1}H NMR spectrum of **TKP-14**

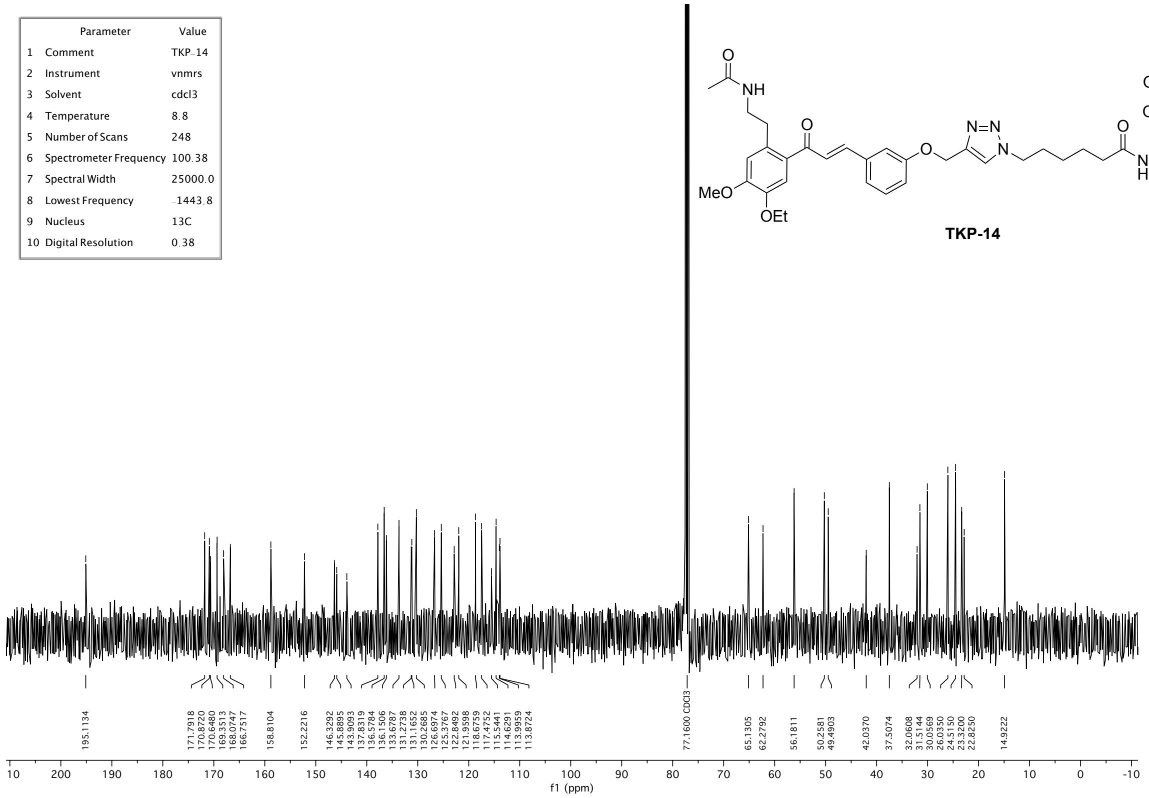

^{13}C NMR spectrum of **TKP-14**

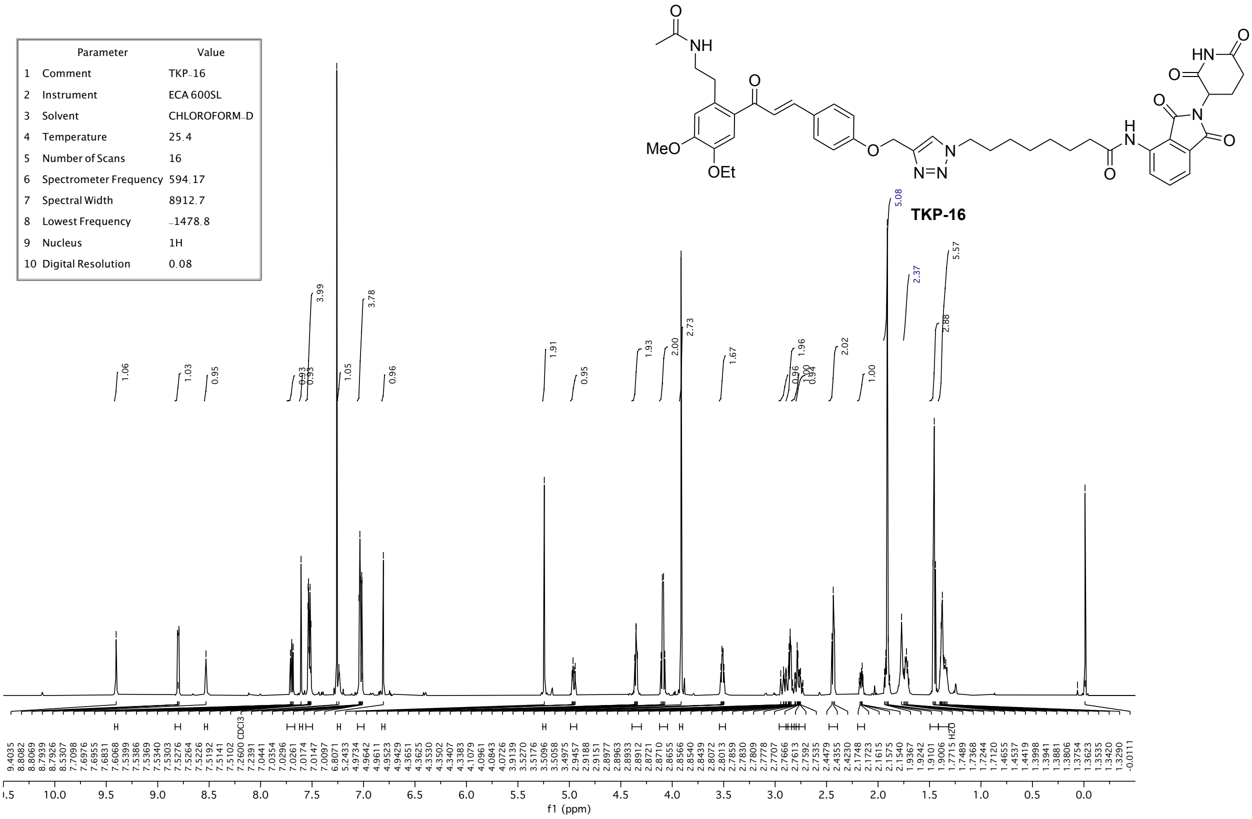

^{1}H NMR spectrum of **TKP-16**

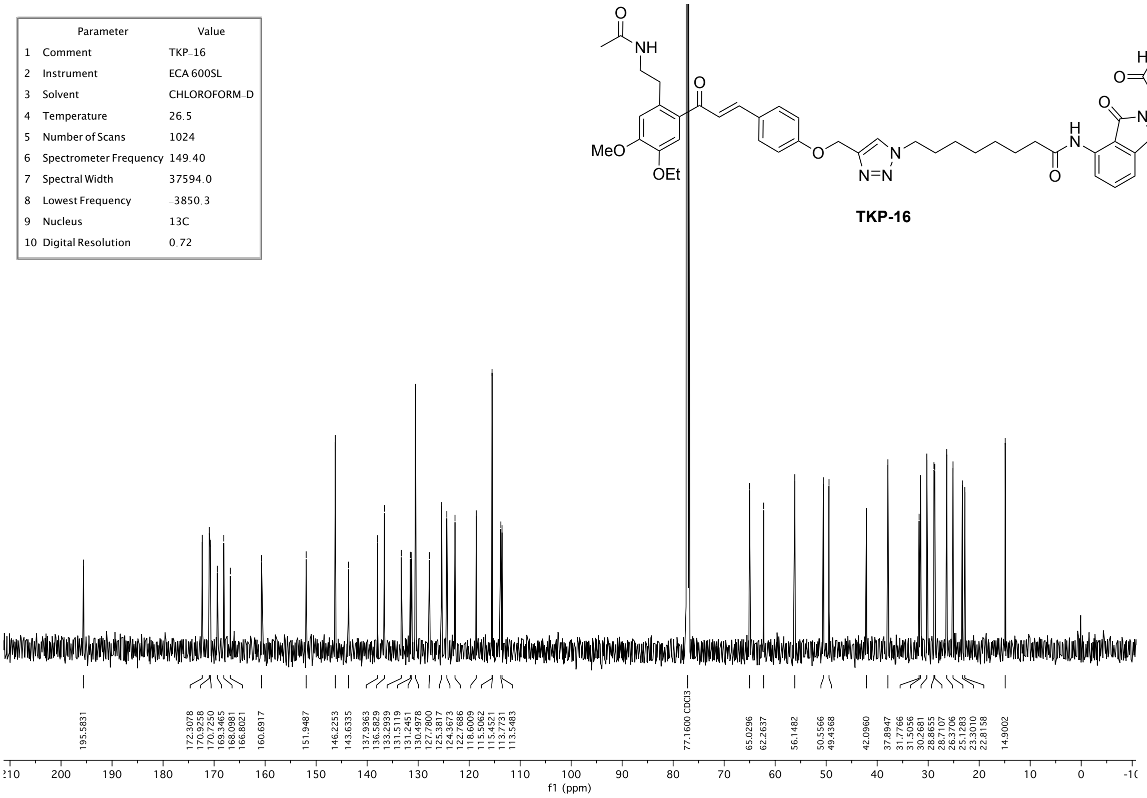

^{13}C NMR spectrum of **TKP-16**

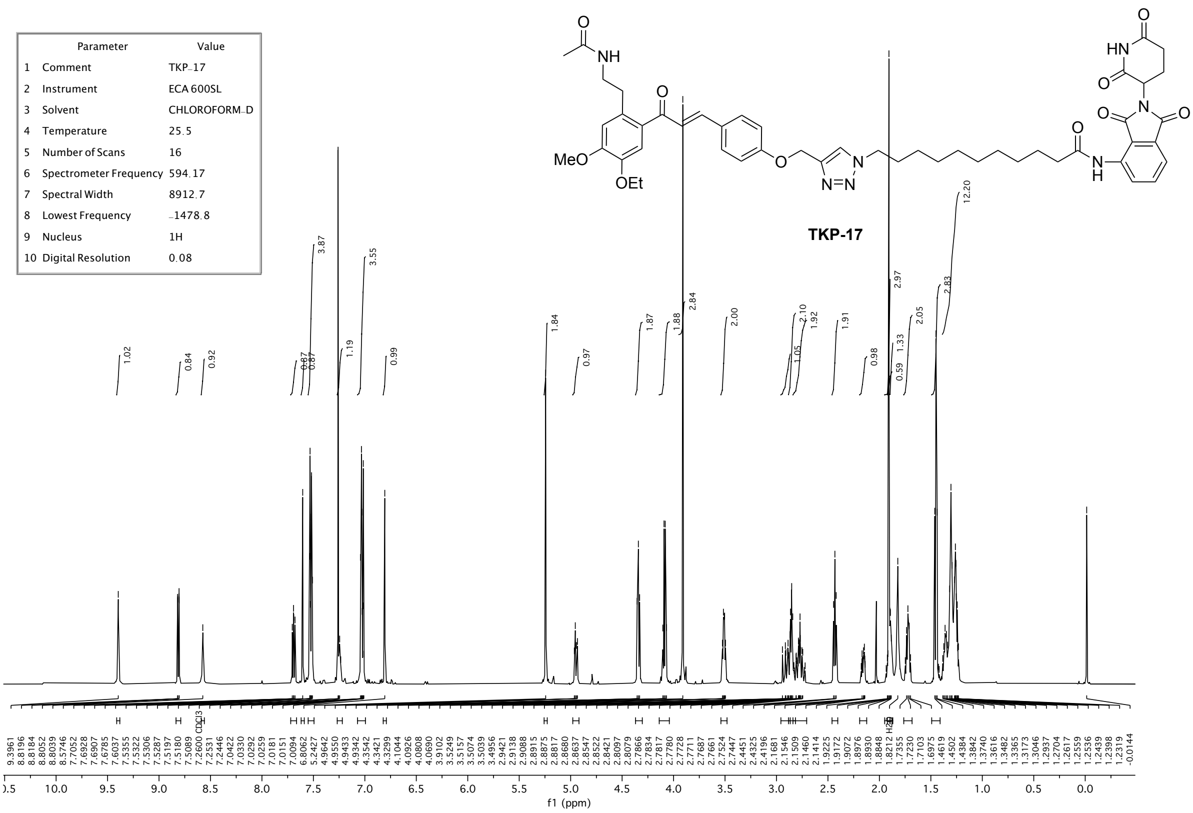

Parameter		Value
1	Comment	TKP_17
2	Instrument	ECA 600SL
3	Solvent	CHLOROFORM_D
4	Temperature	25.5
5	Number of Scans	16
6	Spectrometer Frequency	594.17
7	Spectral Width	8912.7
8	Lowest Frequency	-1478.8
9	Nucleus	1H
10	Digital Resolution	0.08

^{1}H NMR spectrum of **TKP-17**

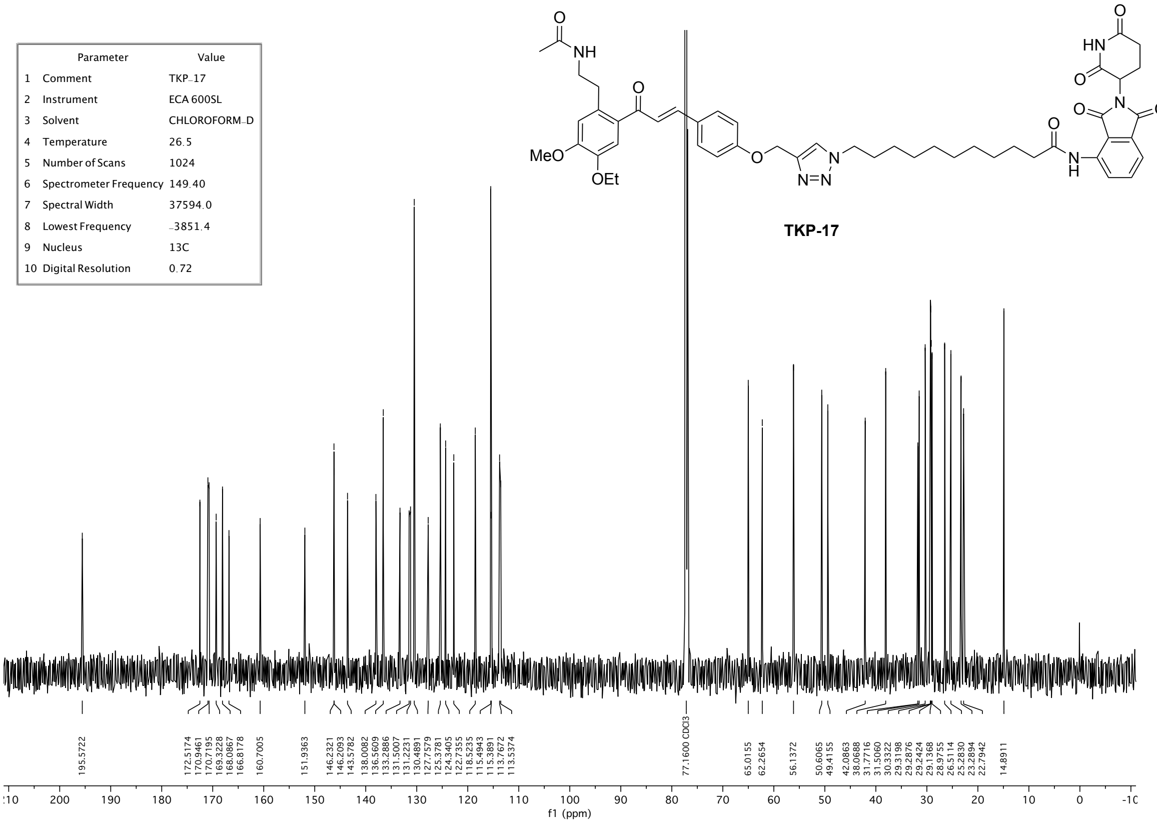

Parameter		Value
1	Comment	TKP_17
2	Instrument	ECA 600SL
3	Solvent	CHLOROFORM_D
4	Temperature	26.5
5	Number of Scans	1024
6	Spectrometer Frequency	149.40
7	Spectral Width	37594.0
8	Lowest Frequency	-3851.4
9	Nucleus	13C
10	Digital Resolution	0.72

^{13}C NMR spectrum of **TKP-17**

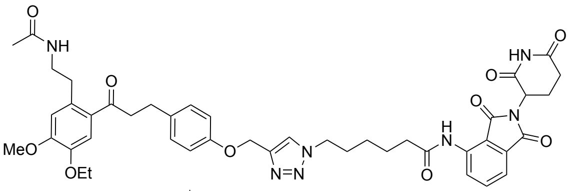

TKP-18

	Parameter	Value
1	Comment	TKP_18
2	Instrument	ECA 600SL
3	Solvent	CHLOROFORM_D
4	Temperature	25.5
5	Number of Scans	16
6	Spectrometer Frequency	594.17
7	Spectral Width	8912.7
8	Lowest Frequency	-1478.8
9	Nucleus	1H
10	Digital Resolution	0.08

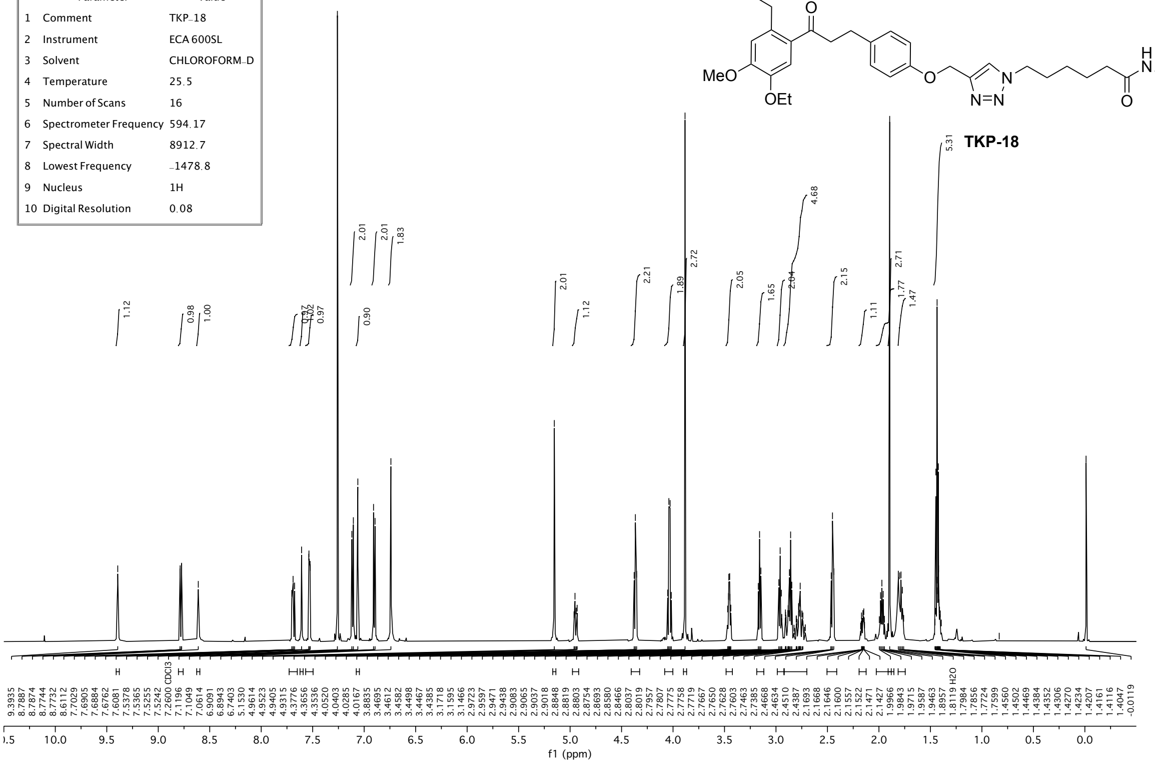

^{1}H NMR spectrum of **TKP-18**

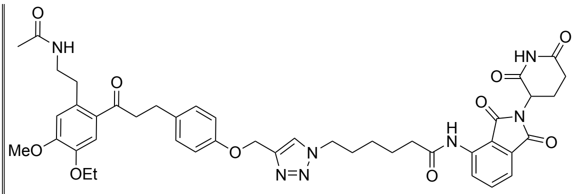

TKP-18

	Parameter	Value
1	Comment	TKP_18
2	Instrument	ECA 600SL
3	Solvent	CHLOROFORM_D
4	Temperature	26.6
5	Number of Scans	1024
6	Spectrometer Frequency	149.40
7	Spectral Width	37594.0
8	Lowest Frequency	-3851.3
9	Nucleus	13C
10	Digital Resolution	0.72

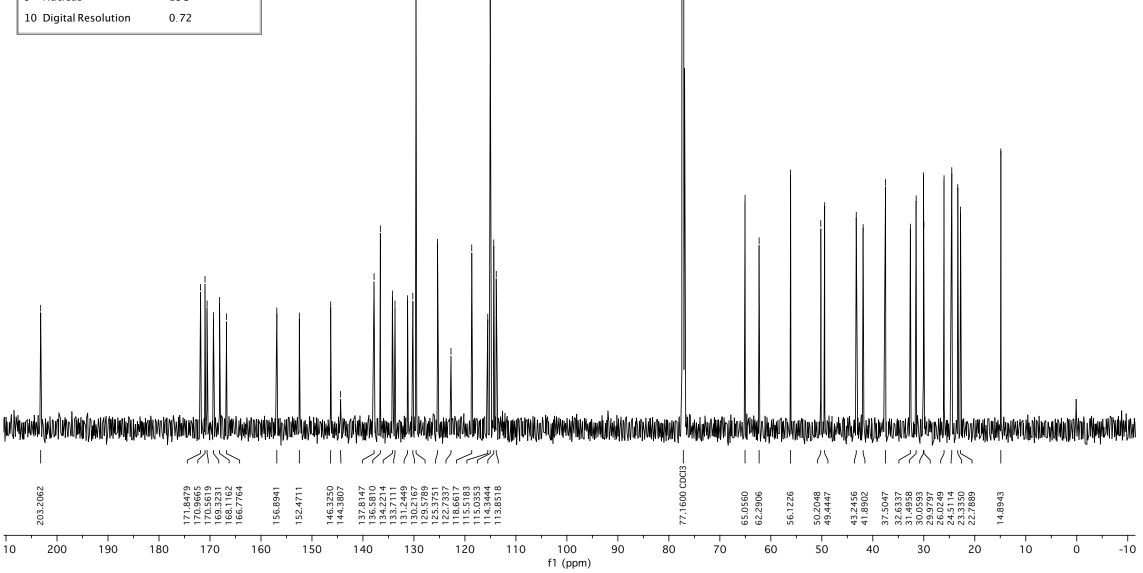

^{13}C NMR spectrum of **TKP-18**

	Parameter	Value
1	Comment	TKP-20
2	Instrument	vnmrs
3	Solvent	cdcl3
4	Temperature	8.8
5	Number of Scans	16
6	Spectrometer Frequency	399.14
7	Spectral Width	6377.6
8	Lowest Frequency	-786.5
9	Nucleus	1H
10	Digital Resolution	0.10

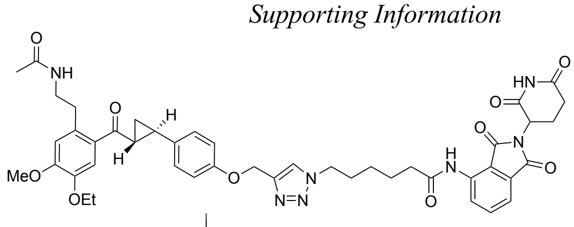

TKP-20

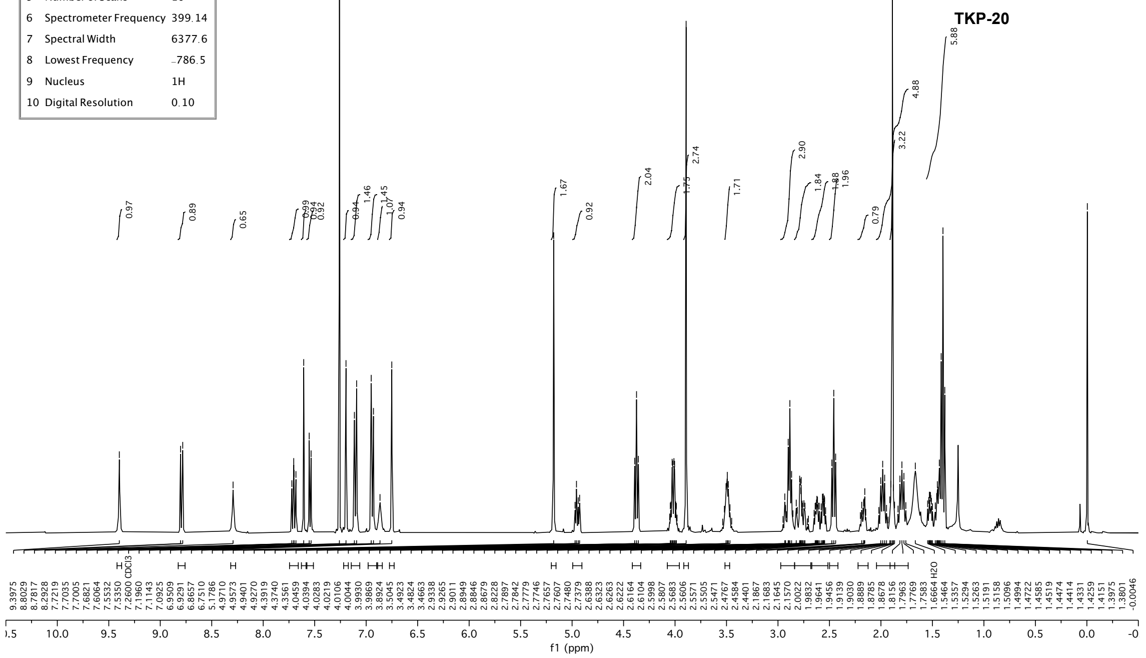

^{1}H NMR spectrum of **TKP-20**

	Parameter	Value
1	Comment	TKP-20
2	Instrument	vnmrs
3	Solvent	cdcl3
4	Temperature	8.8
5	Number of Scans	768
6	Spectrometer Frequency	100.37
7	Spectral Width	25000.0
8	Lowest Frequency	-1444.0
9	Nucleus	13C
10	Digital Resolution	0.38

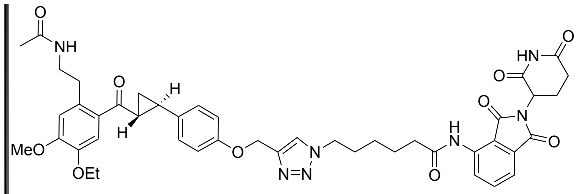

TKP-20

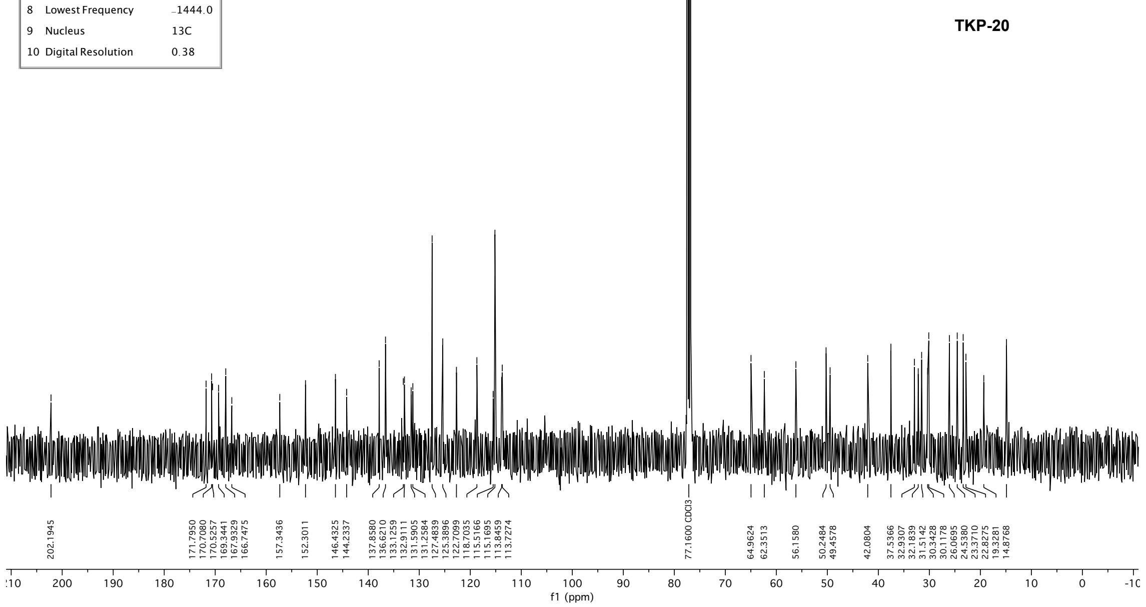

^{13}C NMR spectrum of **TKP-20**

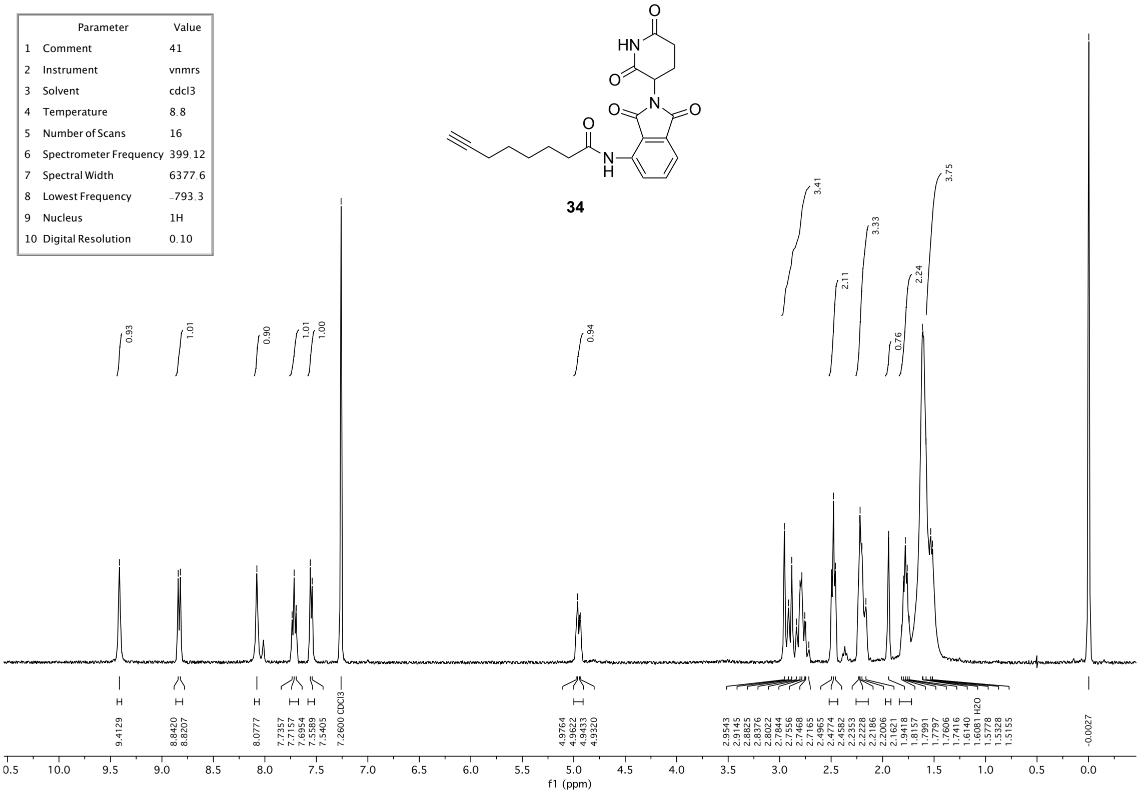

^{1}H NMR spectrum of compound **34**

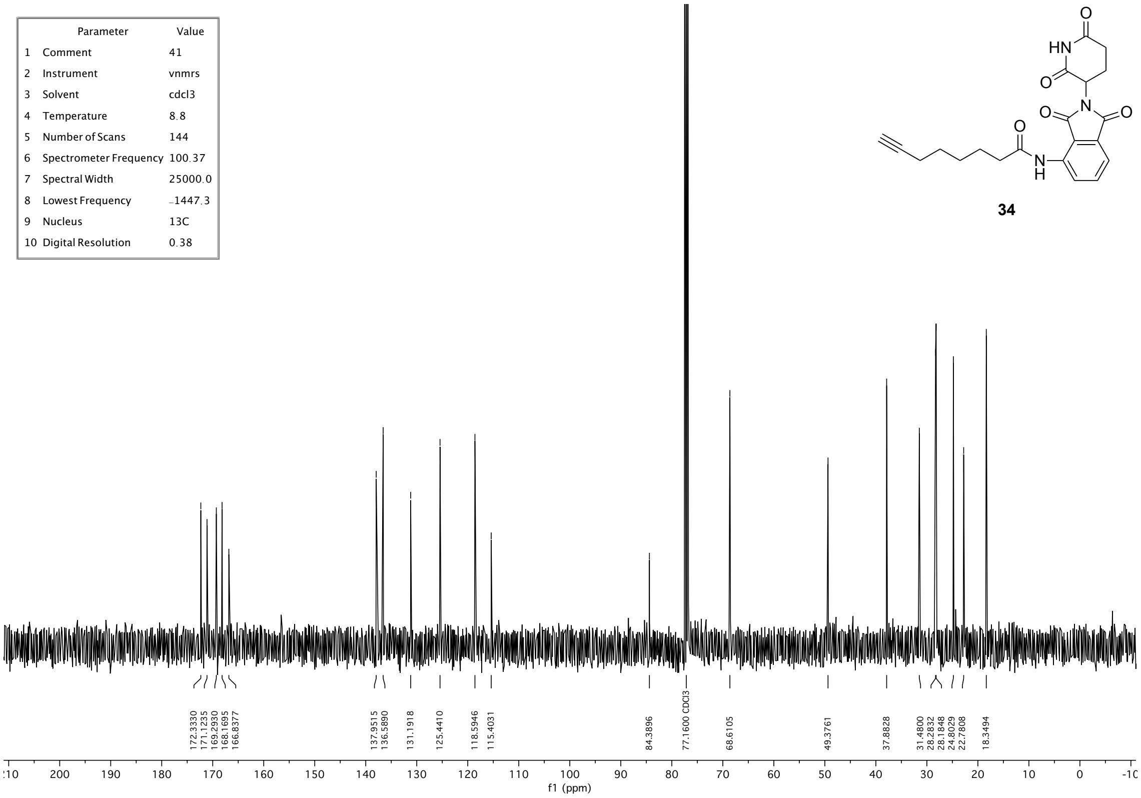

^{13}C NMR spectrum of compound **34**

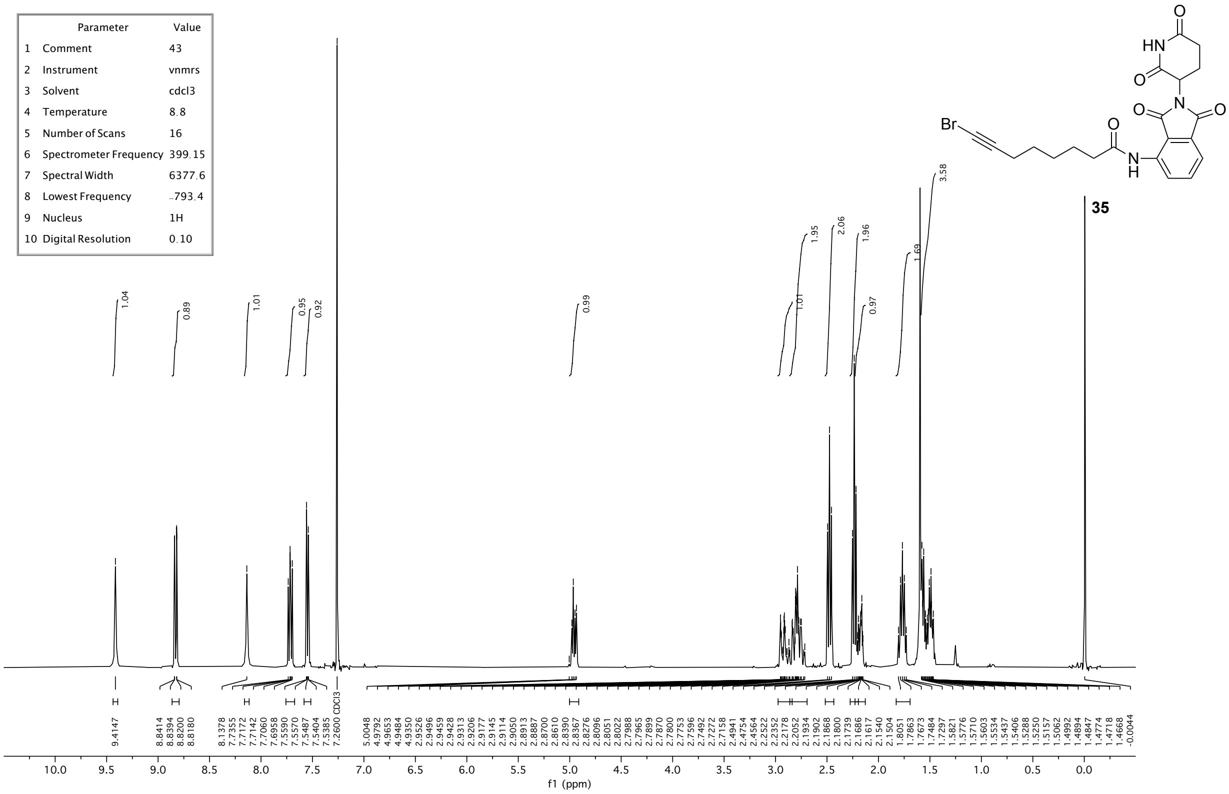

^{1}H NMR spectrum of compound **35**

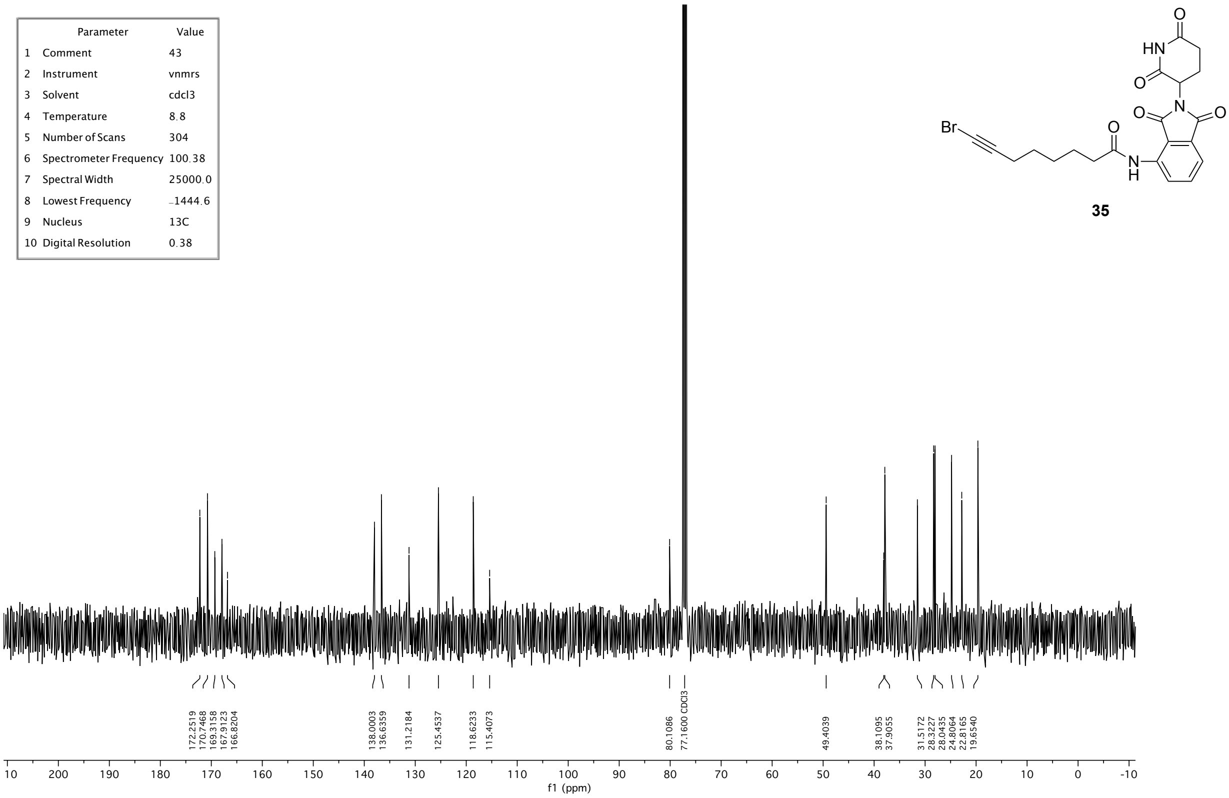

^{13}C NMR spectrum of compound **35**

	Parameter	Value
1	Comment	42
2	Instrument	JNM-ECZ600R/ S1
3	Solvent	DMSO-D6
4	Temperature	22.8
5	Number of Scans	16
6	Spectrometer Frequency	600.17
7	Spectral Width	9025.3
8	Lowest Frequency	-1489.3
9	Nucleus	1H
10	Digital Resolution	0.09

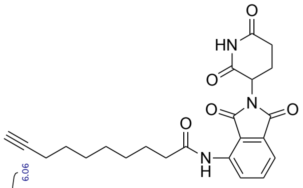

36

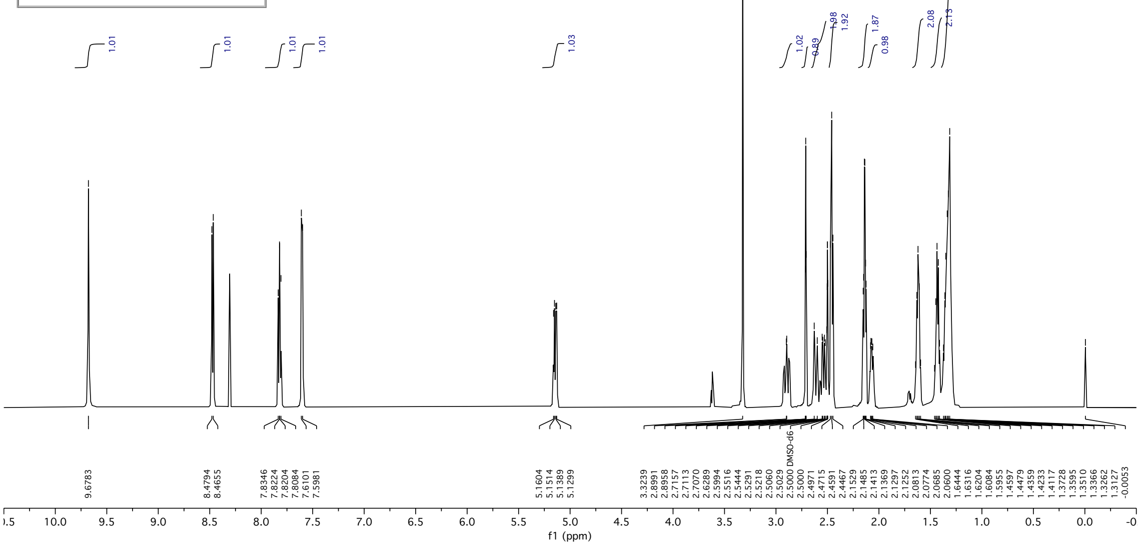

^{1}H NMR spectrum of compound **36**

	Parameter	Value
1	Comment	42
2	Instrument	JNM-ECZ600R/ S1
3	Solvent	DMSO-D6
4	Temperature	21.8
5	Number of Scans	512
6	Spectrometer Frequency	150.91
7	Spectral Width	37878.8
8	Lowest Frequency	-3928.7
9	Nucleus	13C
10	Digital Resolution	0.36

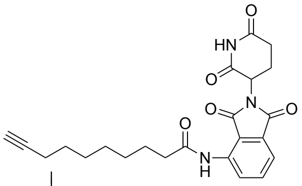

36

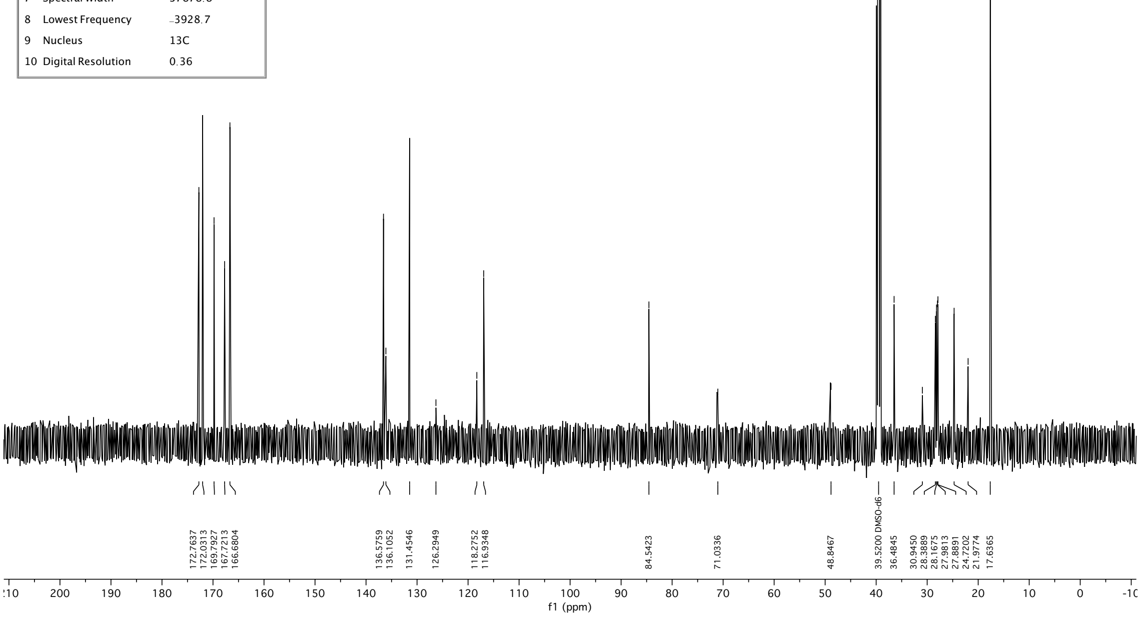

^{13}C NMR spectrum of compound **36**

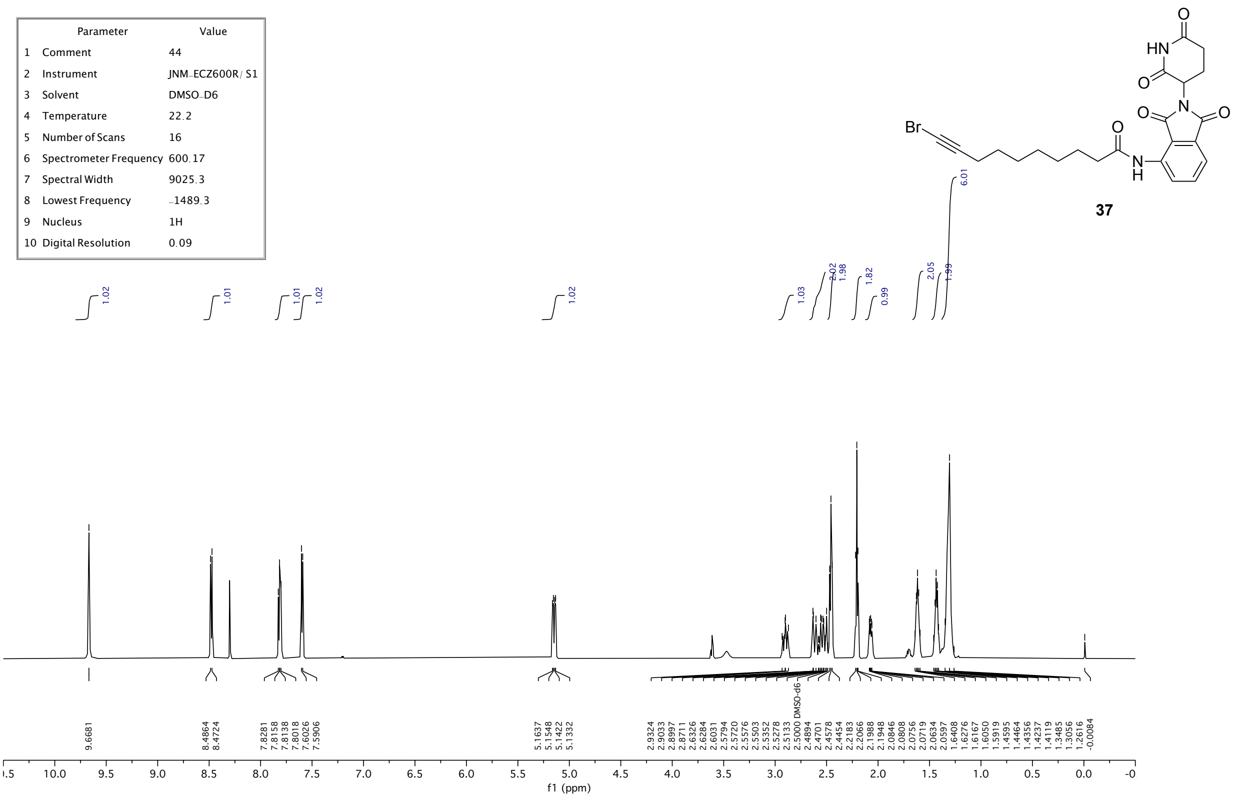

^{1}H NMR spectrum of compound **37**

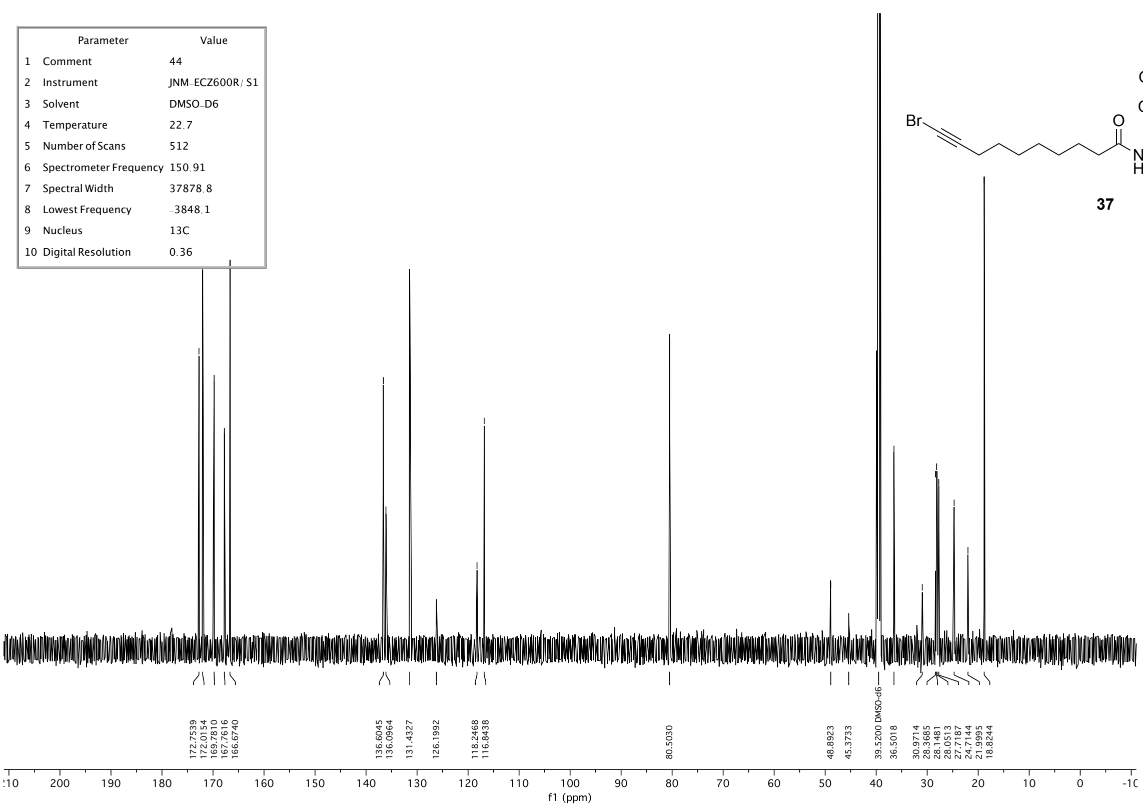

^{13}C NMR spectrum of compound **37**

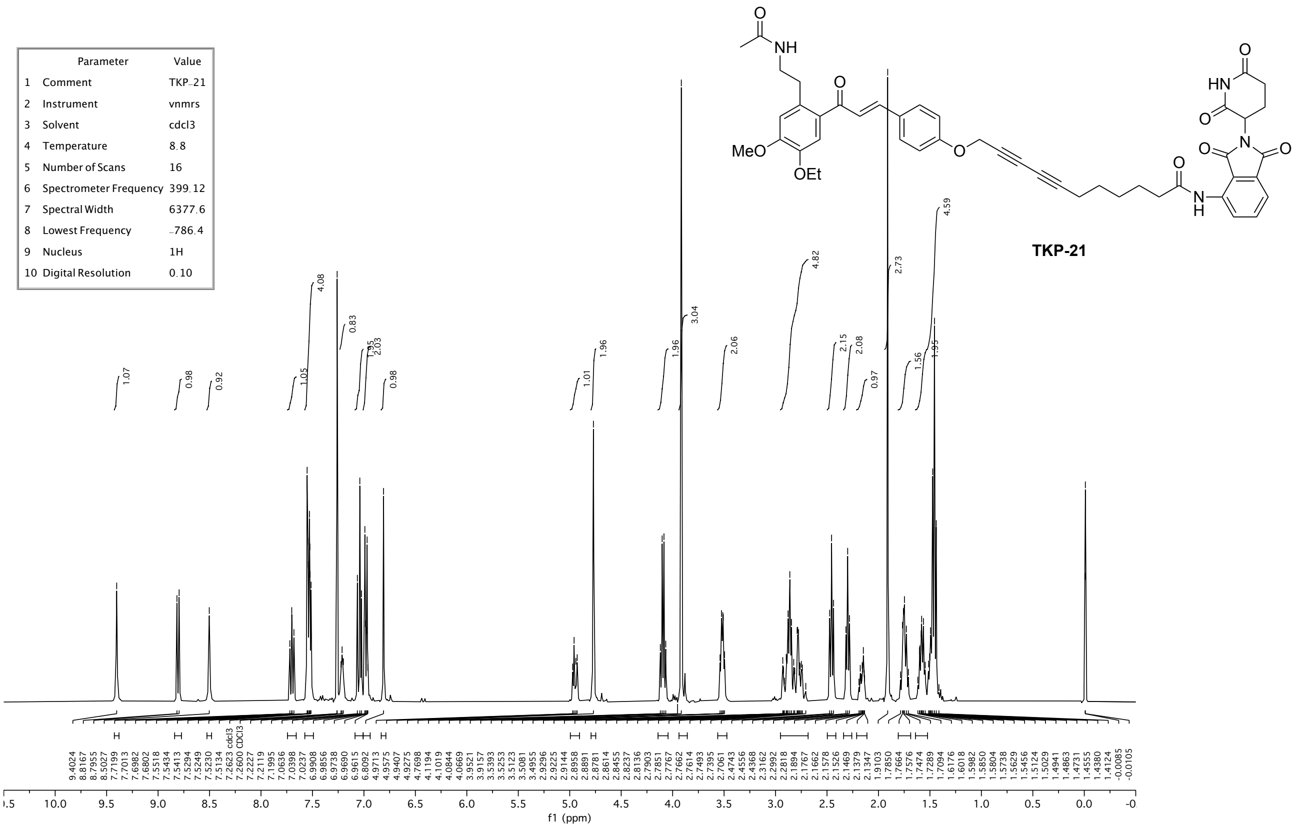

^{1}H NMR spectrum of **TKP-21**

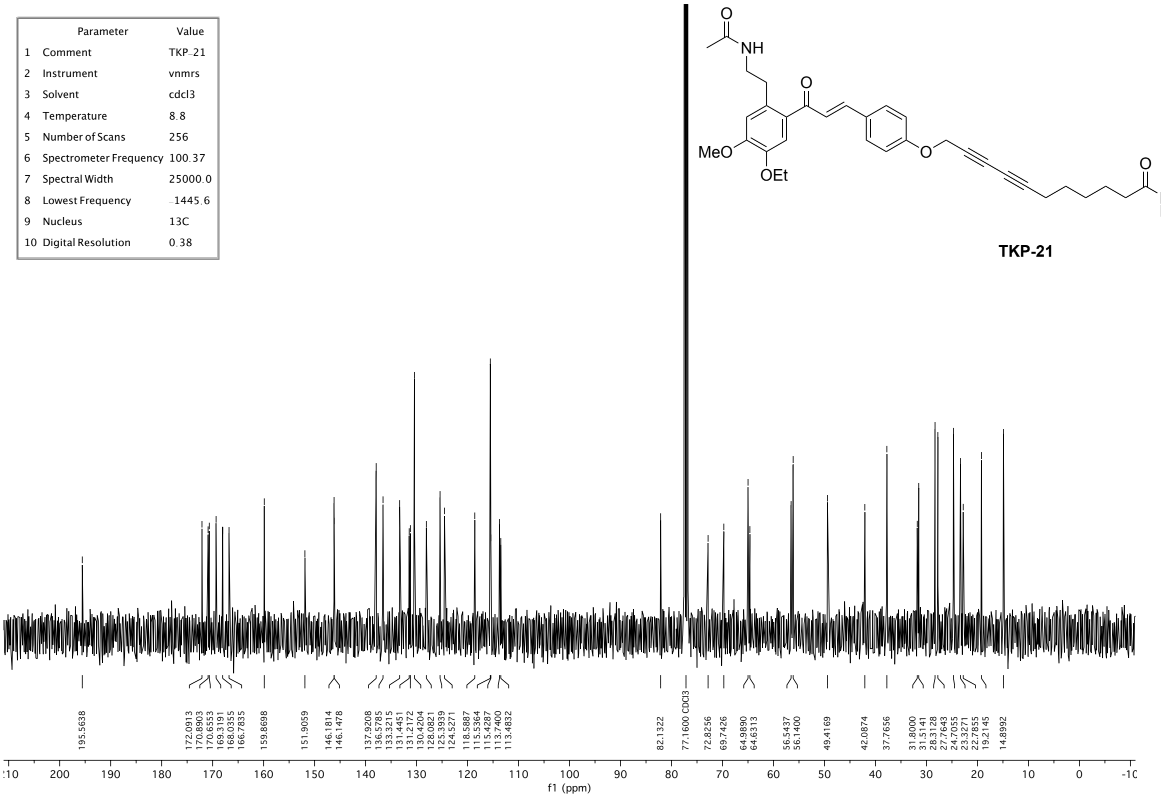

^{13}C NMR spectrum of **TKP-21**

	Parameter	Value
1	Comment	46
2	Instrument	JNM-ECZ600R/S1
3	Solvent	CHLOROFORM-D
4	Temperature	23.4
5	Number of Scans	16
6	Spectrometer Frequency	600.17
7	Spectral Width	9025.3
8	Lowest Frequency	-1504.9
9	Nucleus	1H
10	Digital Resolution	0.09

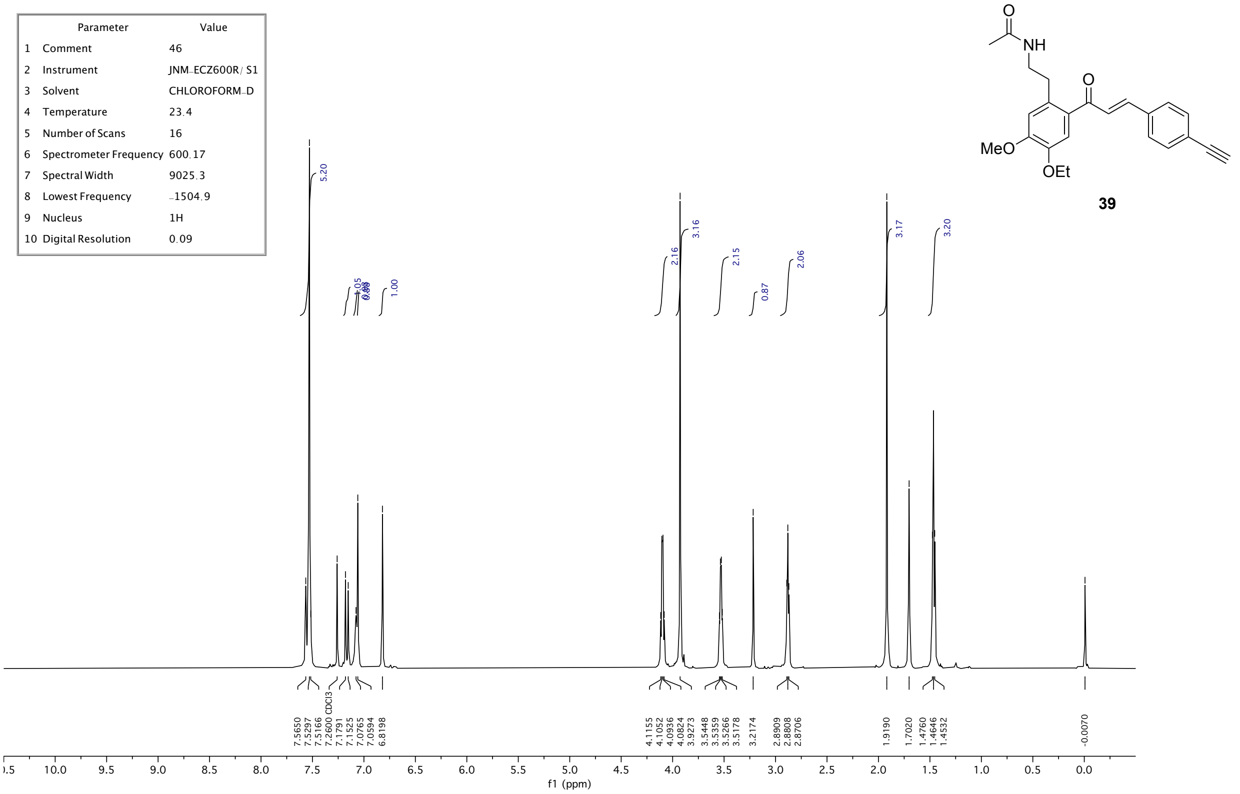

^{1}H NMR spectrum of compound **39**

	Parameter	Value
1	Comment	46
2	Instrument	JNM-ECZ600R/S1
3	Solvent	CHLOROFORM-D
4	Temperature	23.5
5	Number of Scans	256
6	Spectrometer Frequency	150.91
7	Spectral Width	37878.8
8	Lowest Frequency	-3840.1
9	Nucleus	13C
10	Digital Resolution	0.36

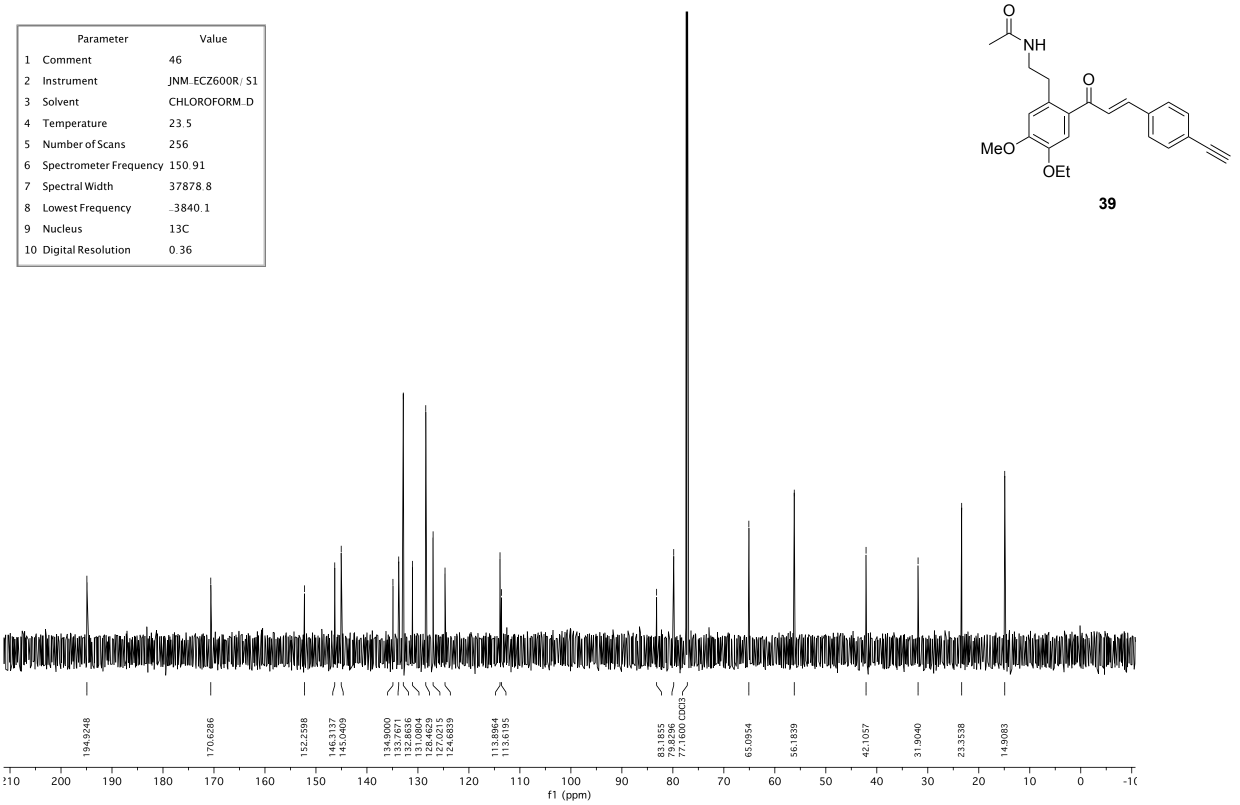

^{13}C NMR spectrum of compound **39**

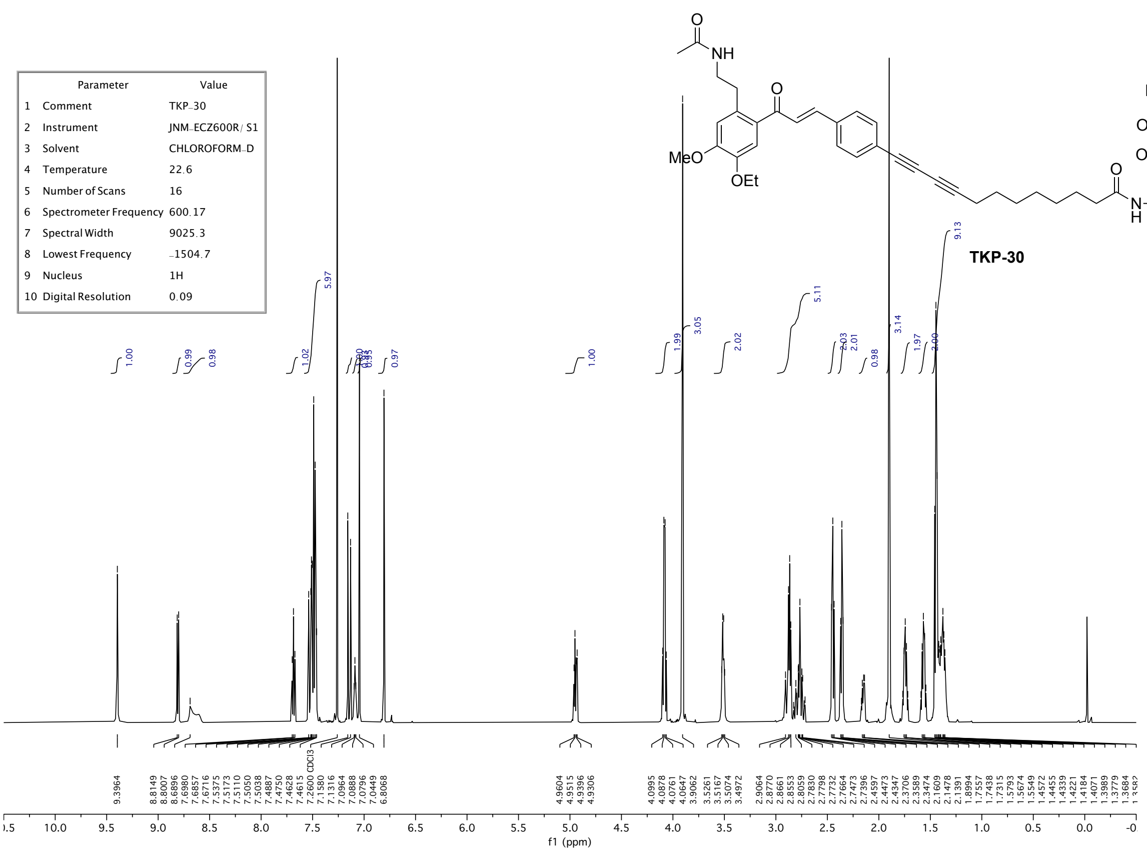

^{1}H NMR spectrum of **TKP-30**

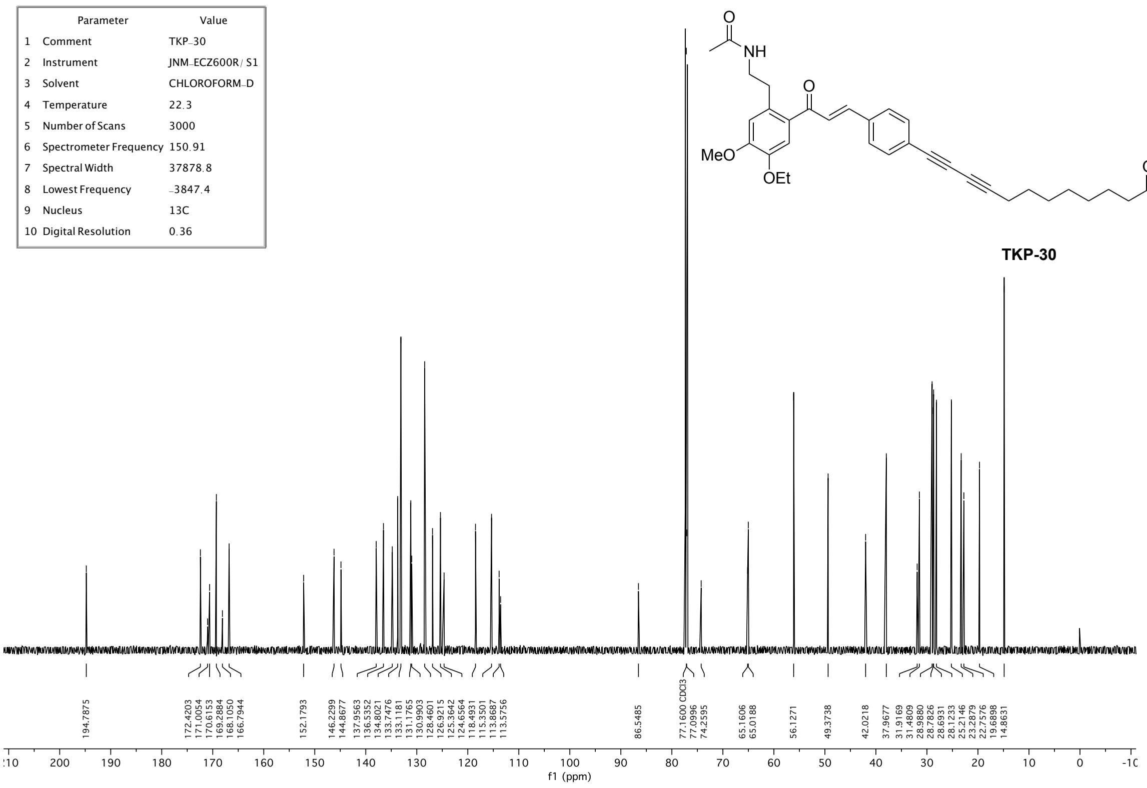

^{13}C NMR spectrum of **TKP-30**

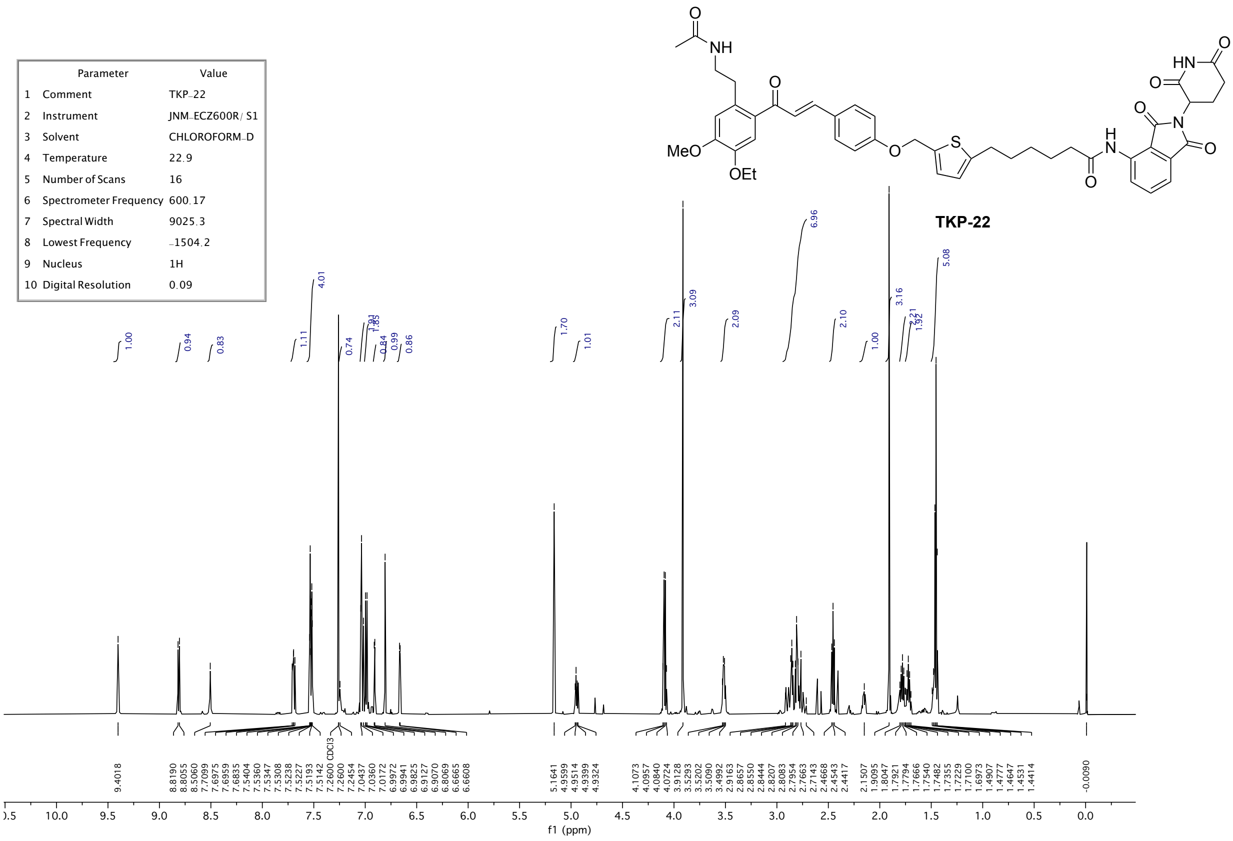

^{1}H NMR spectrum of **TKP-22**

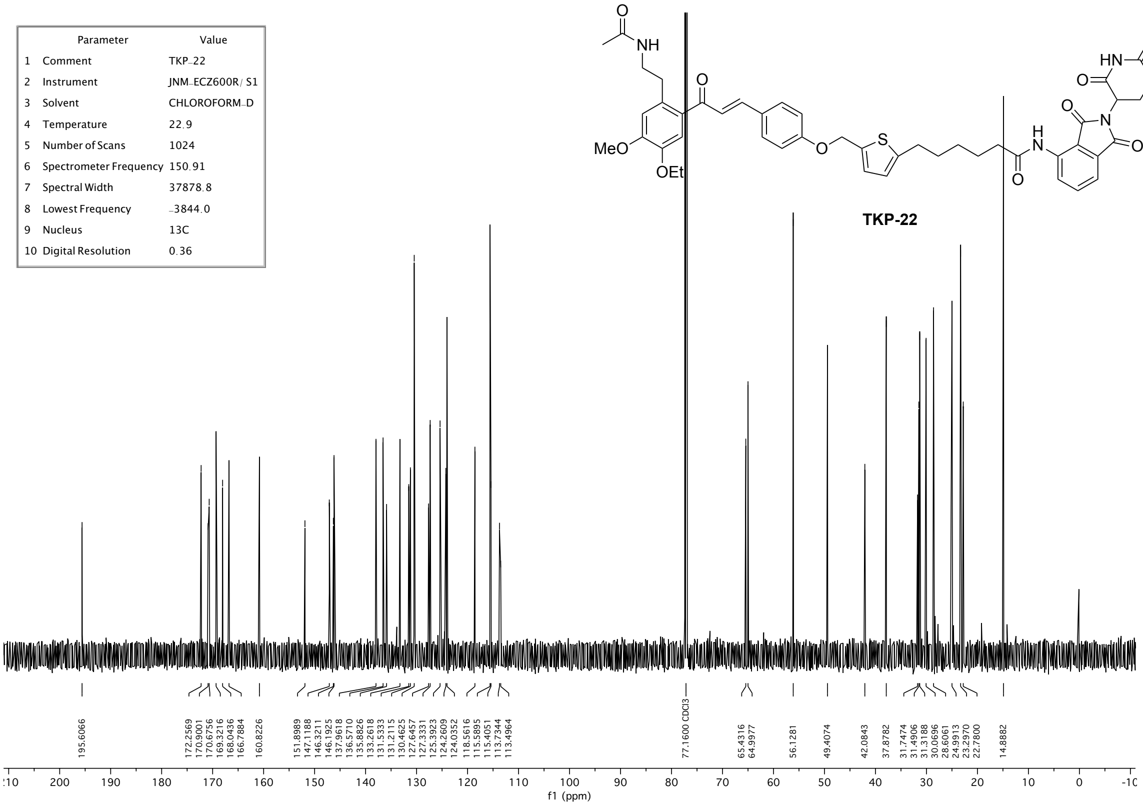

^{13}C NMR spectrum of **TKP-22**

	Parameter	Value
1	Comment	TKP-44
2	Instrument	JNM-ECA600
3	Solvent	CHLOROFORM-D
4	Temperature	23.1
5	Number of Scans	16
6	Spectrometer Frequency	597.17
7	Spectral Width	8960.6
8	Lowest Frequency	-1489.0
9	Nucleus	1H
10	Digital Resolution	0.09

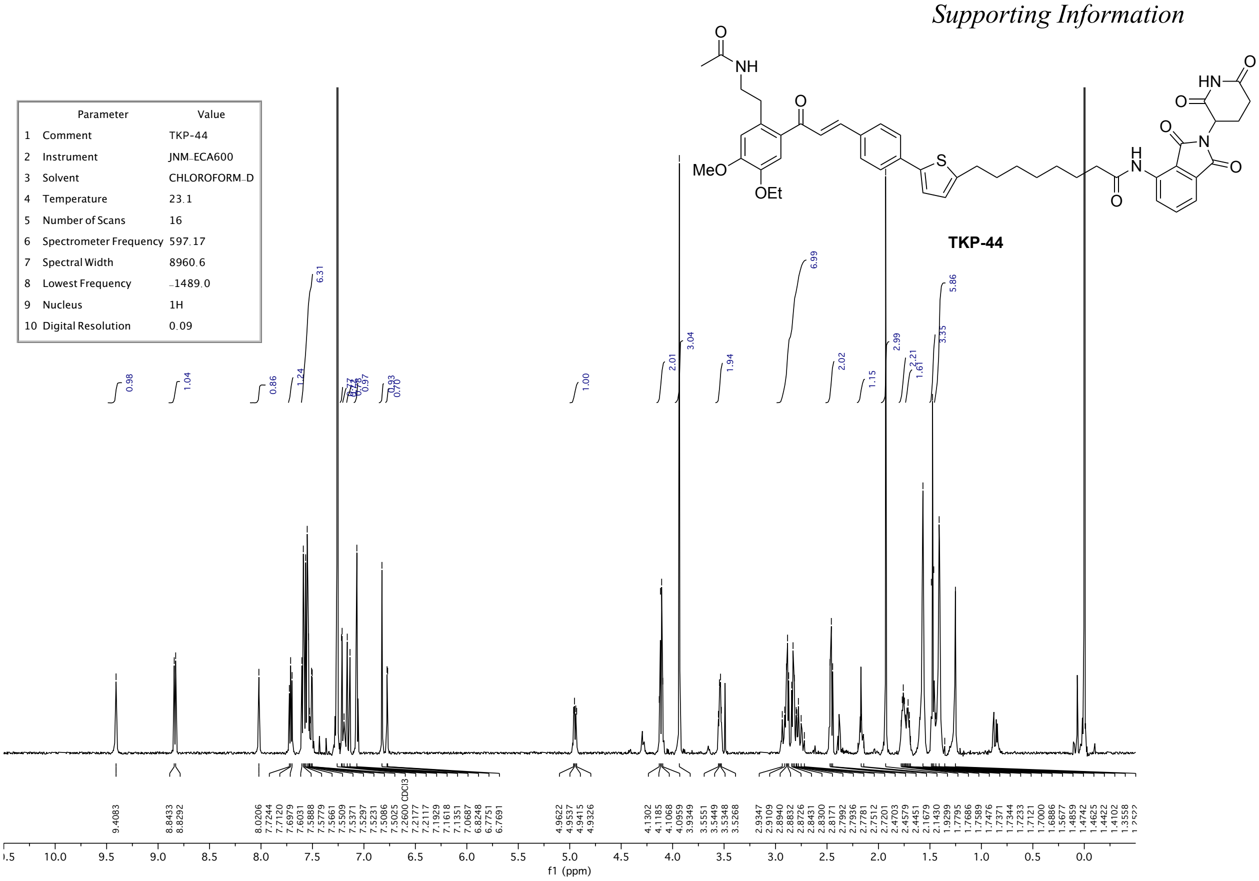

^{1}H NMR spectrum of **TKP-44**

	Parameter	Value
1	Comment	TKP-44
2	Instrument	JNM-ECA600
3	Solvent	CHLOROFORM-D
4	Temperature	23.3
5	Number of Scans	40000
6	Spectrometer Frequency	150.16
7	Spectral Width	37594.0
8	Lowest Frequency	-3774.7
9	Nucleus	13C
10	Digital Resolution	0.72

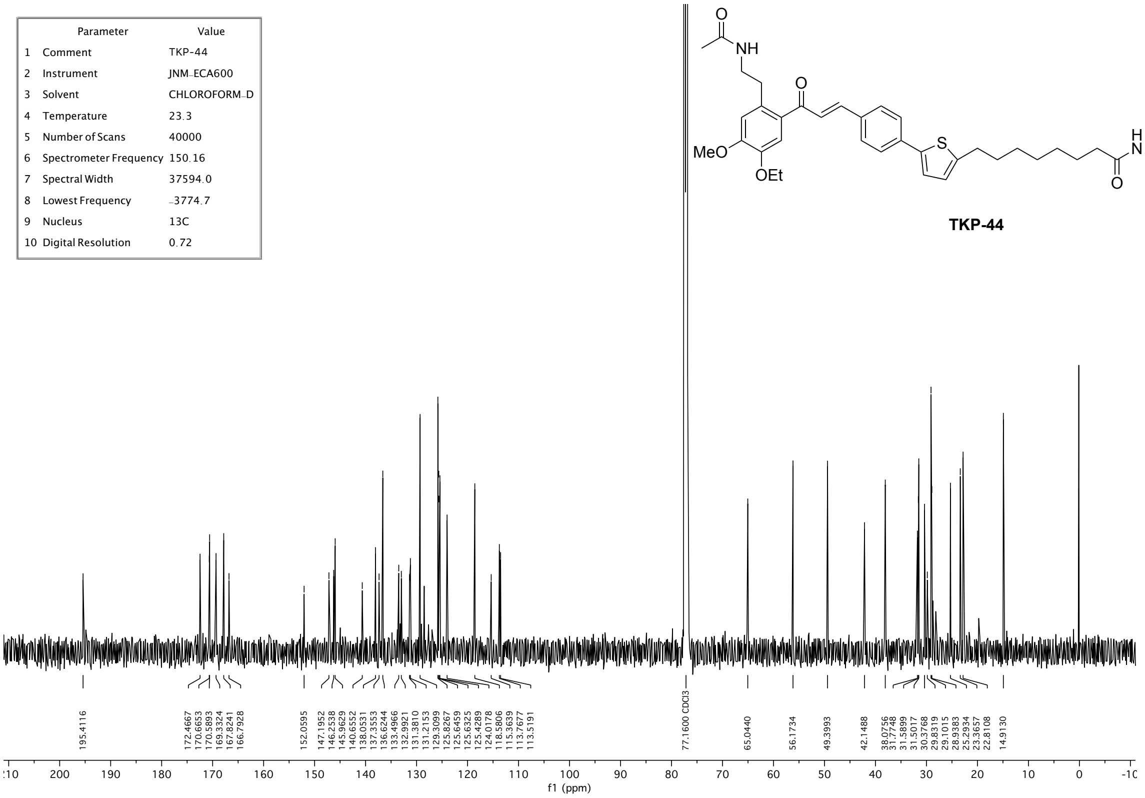

^{13}C NMR spectrum of **TKP-44**

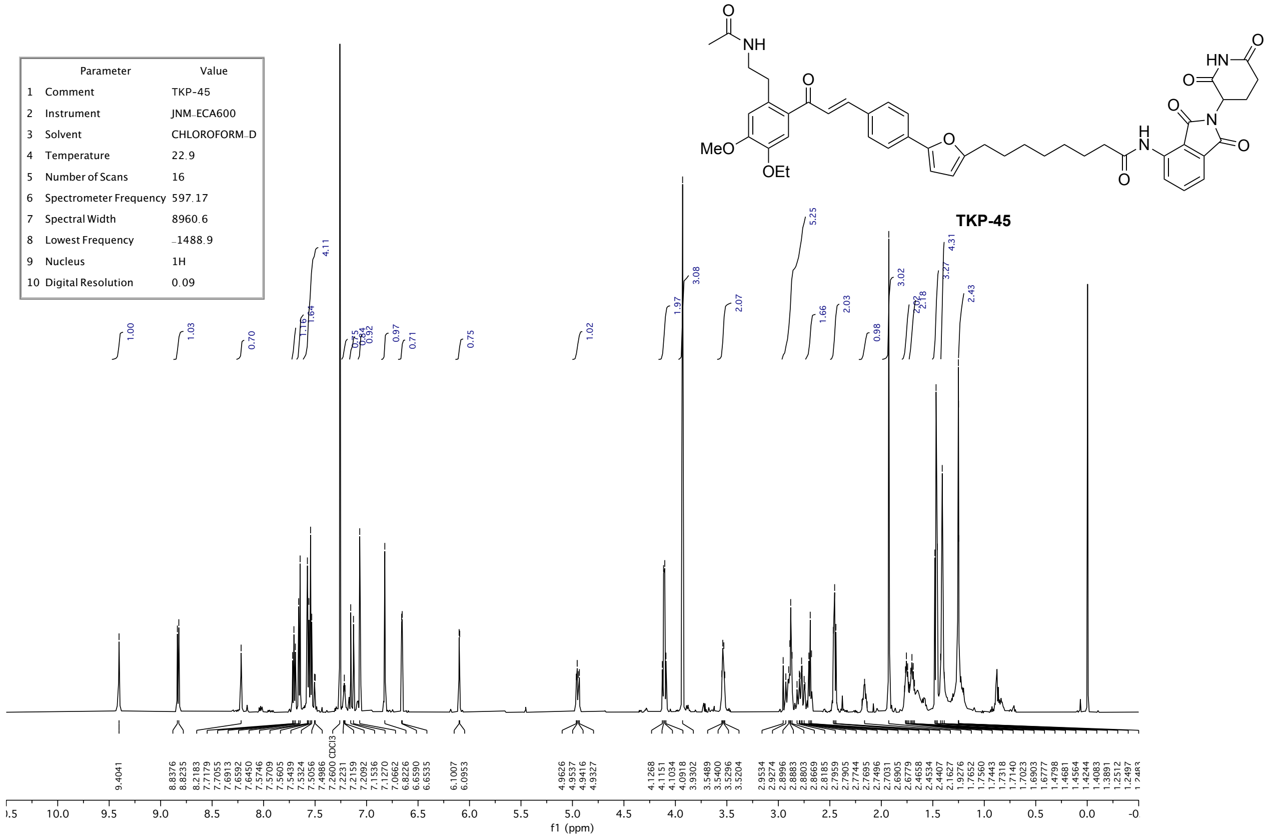

^{1}H NMR spectrum of **TKP-45**

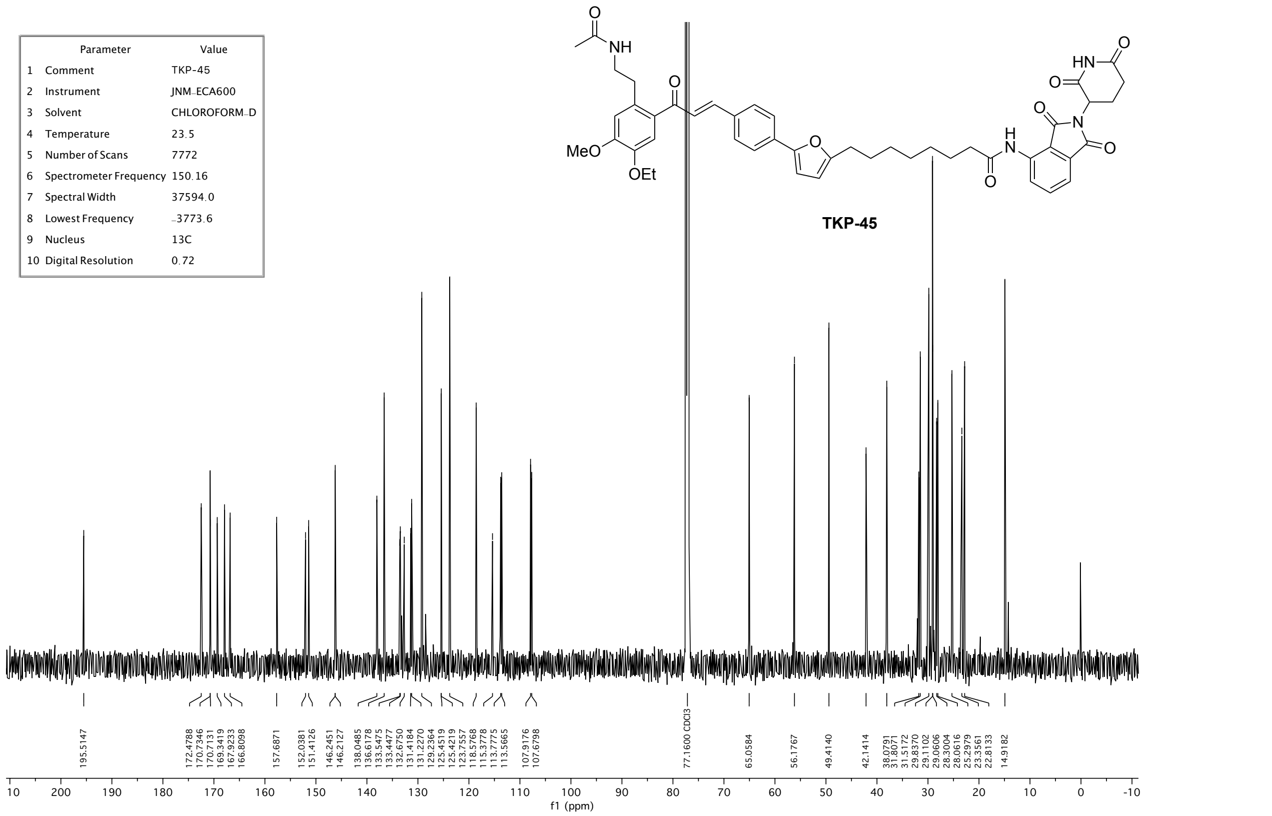

^{13}C NMR spectrum of **TKP-45**

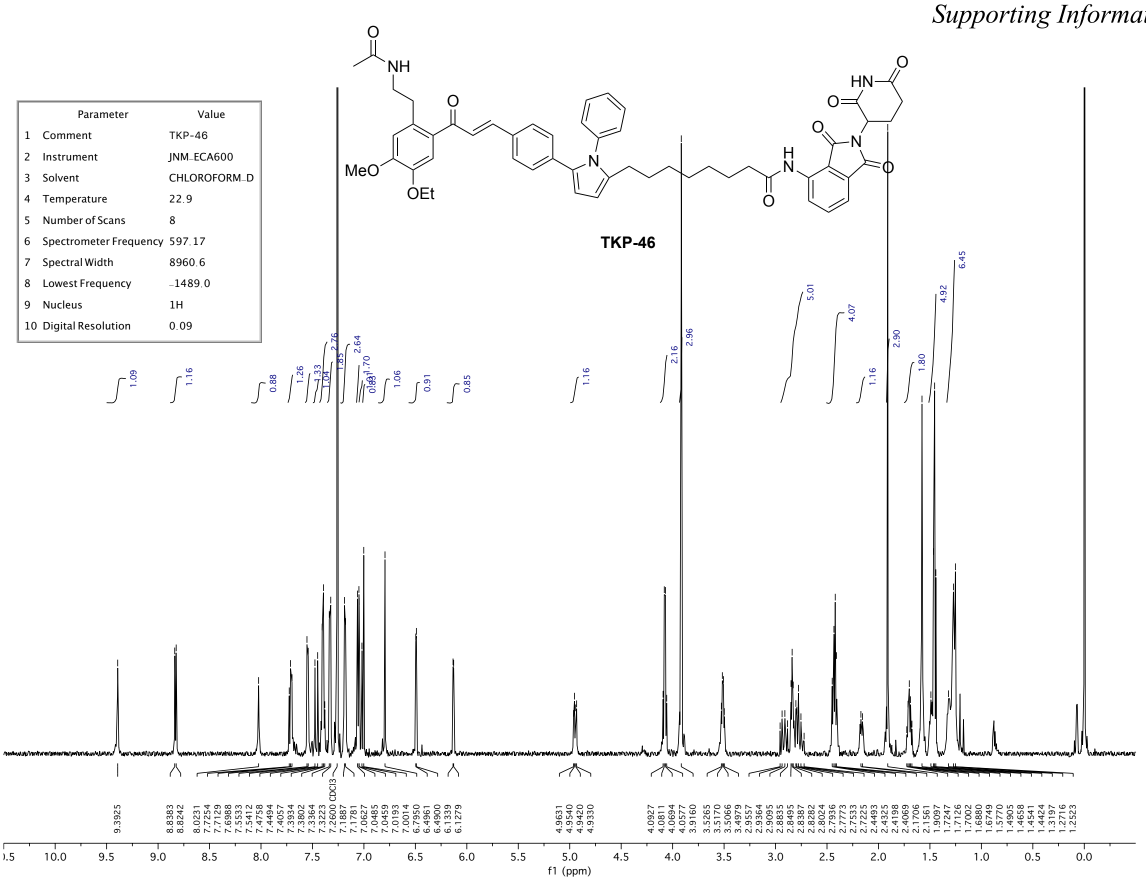

^{1}H NMR spectrum of **TKP-46**

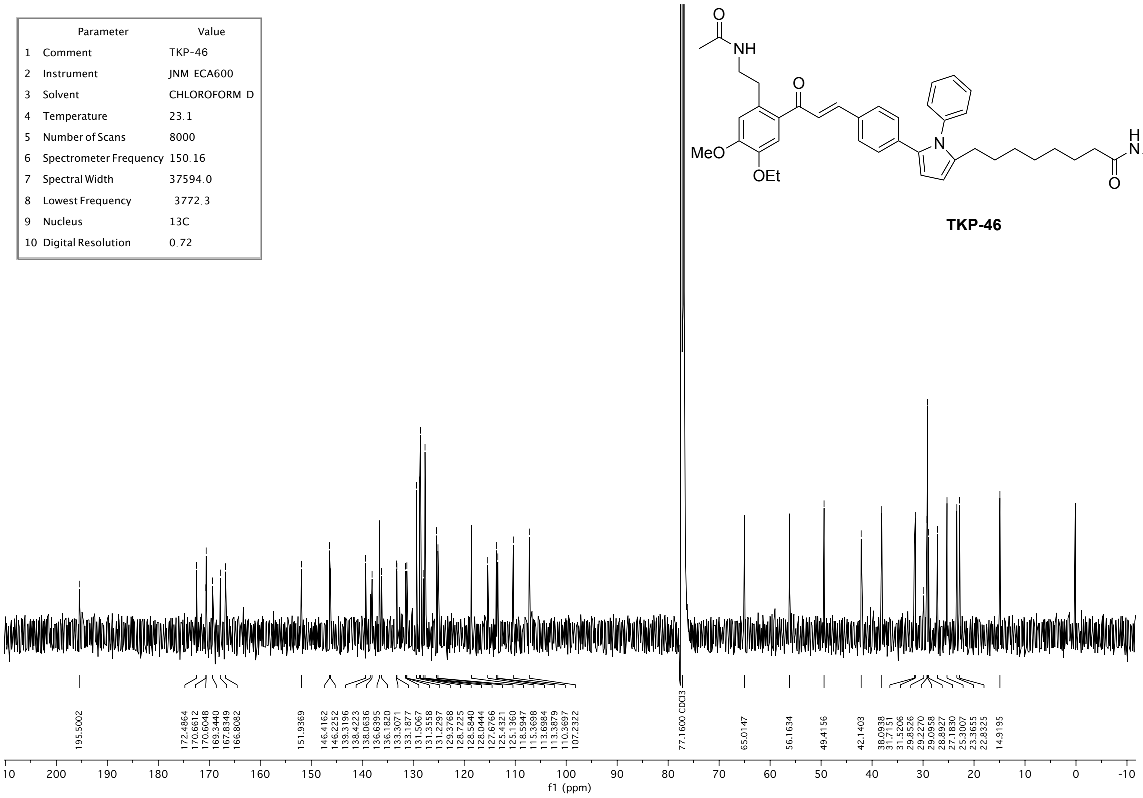

^{13}C NMR spectrum of **TKP-46**

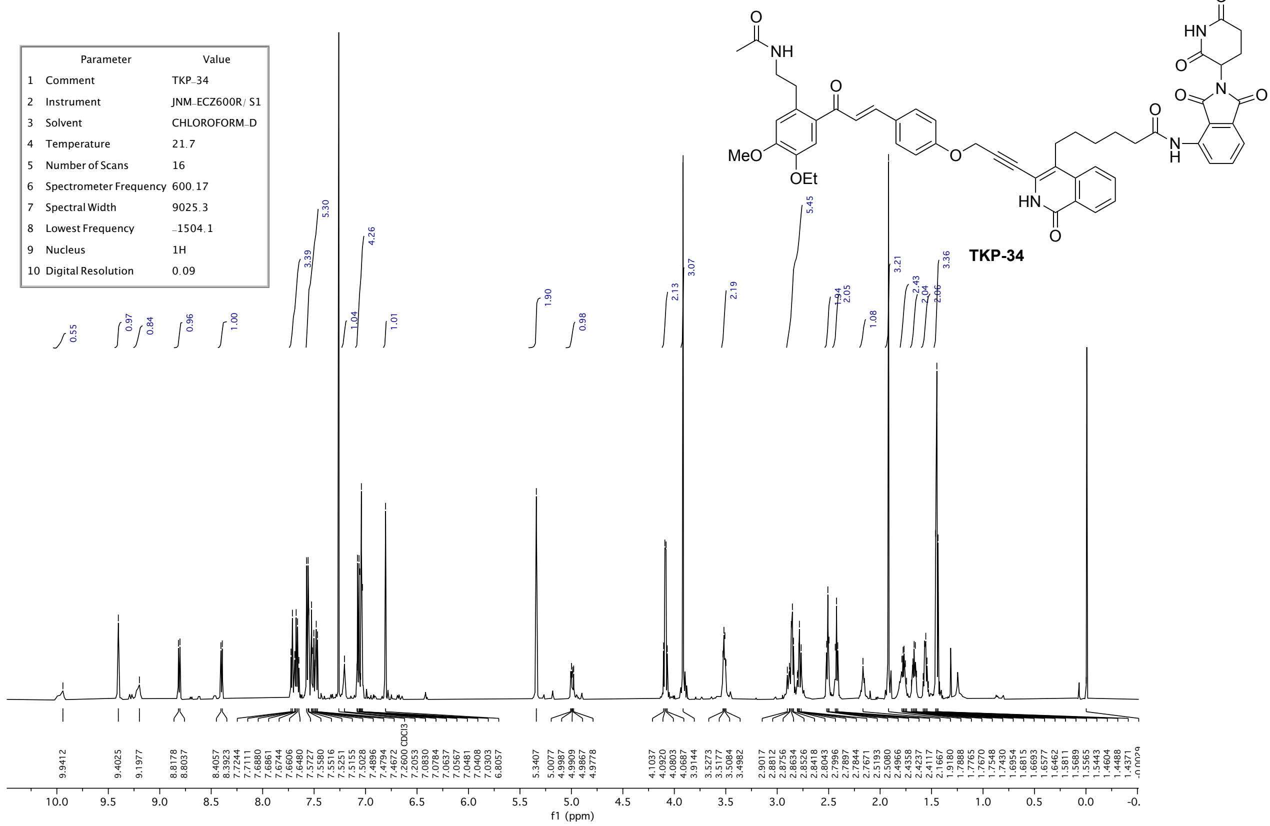

^{1}H NMR spectrum of **TKP-34**

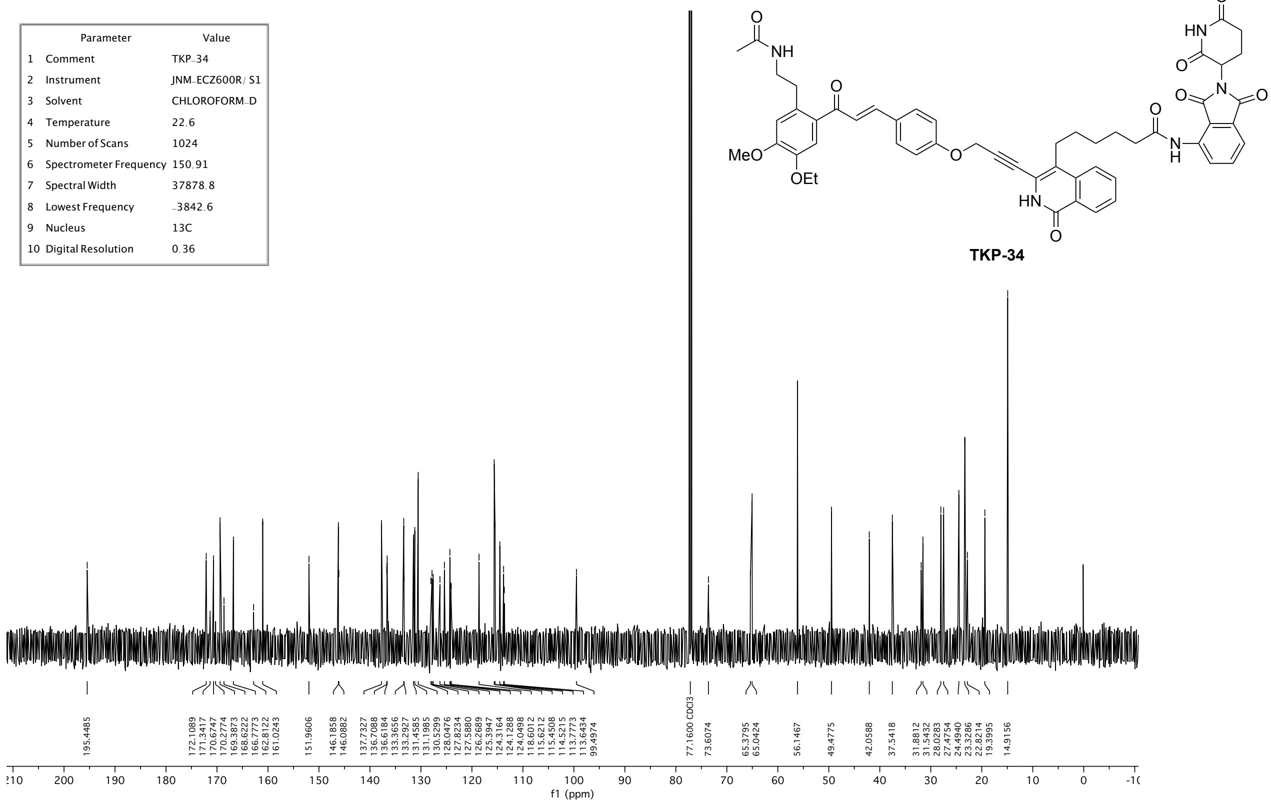

^{13}C NMR spectrum of **TKP-34**

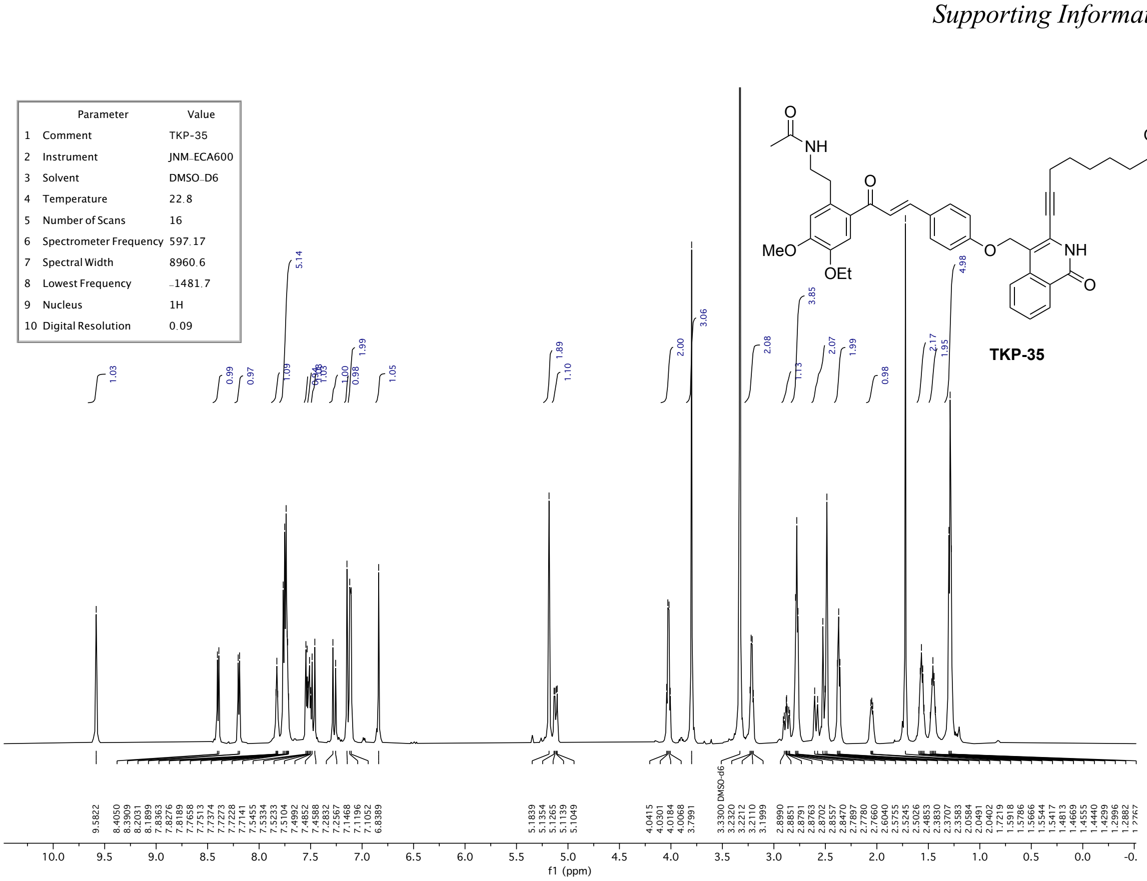

^{1}H NMR spectrum of **TKP-35**

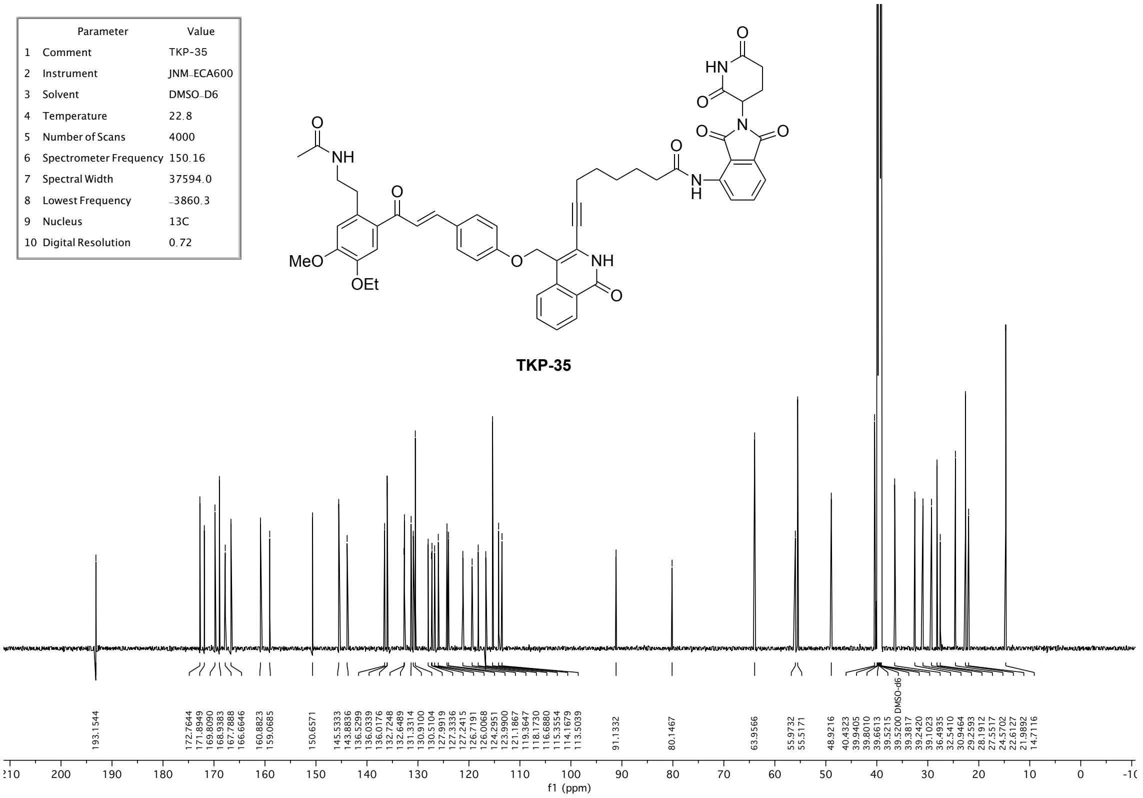

^{13}C NMR spectrum of **TKP-35**

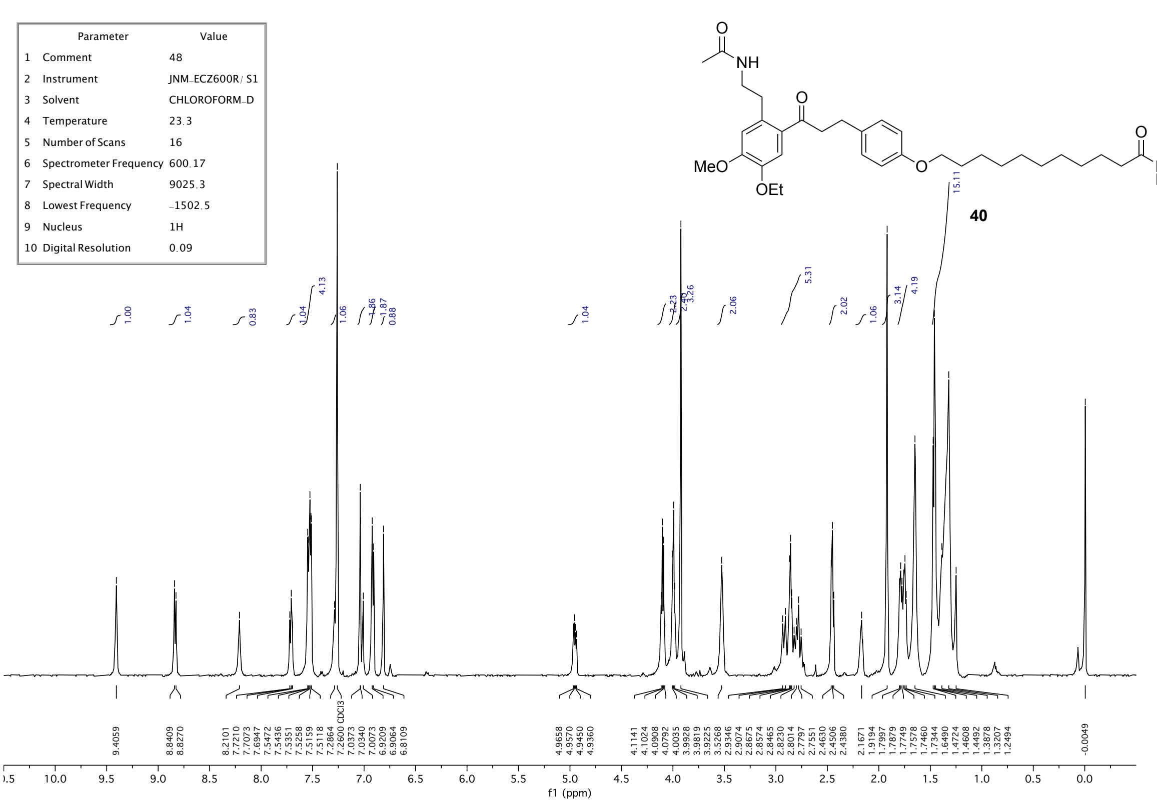

	Parameter	Value
1	Comment	48
2	Instrument	JNM-ECZ600R/S1
3	Solvent	CHLOROFORM-D
4	Temperature	23.3
5	Number of Scans	16
6	Spectrometer Frequency	600.17
7	Spectral Width	9025.3
8	Lowest Frequency	-1502.5
9	Nucleus	1H
10	Digital Resolution	0.09

^{1}H NMR spectrum of **40**

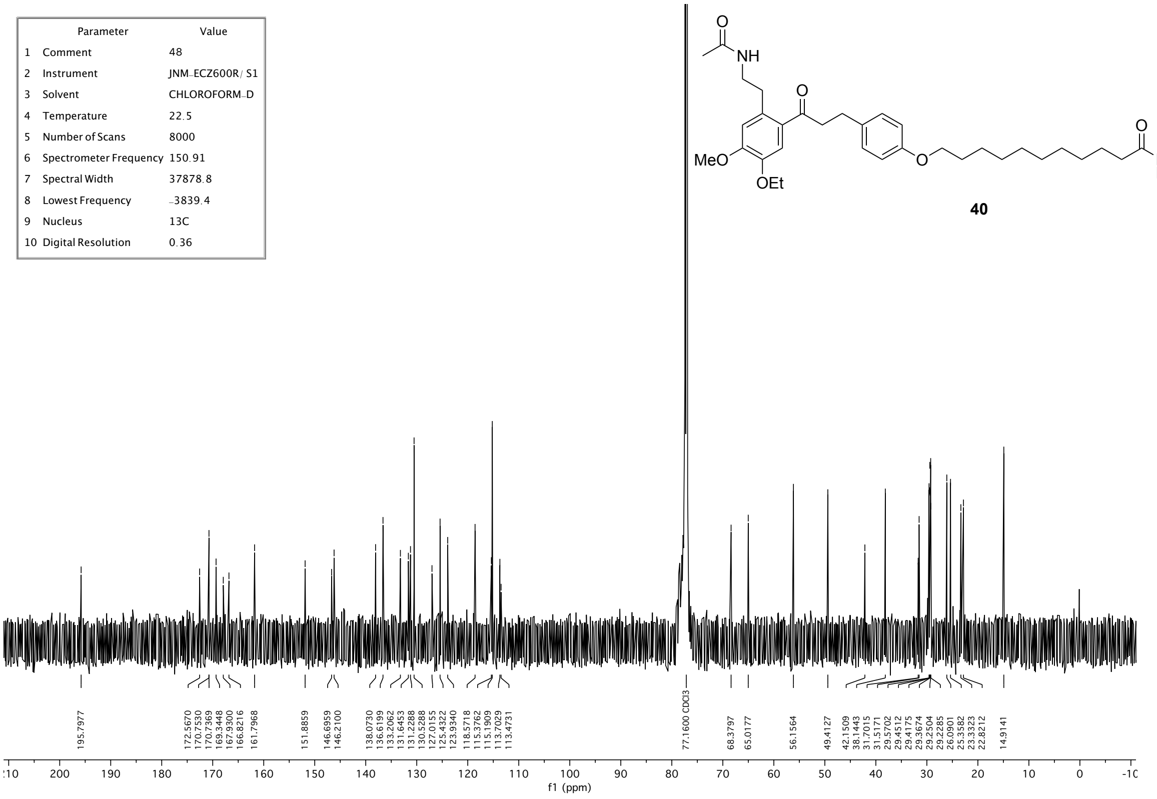

	Parameter	Value
1	Comment	48
2	Instrument	JNM-ECZ600R/S1
3	Solvent	CHLOROFORM-D
4	Temperature	22.5
5	Number of Scans	8000
6	Spectrometer Frequency	150.91
7	Spectral Width	37878.8
8	Lowest Frequency	-3839.4
9	Nucleus	13C
10	Digital Resolution	0.36

^{13}C NMR spectrum of **40**

	Parameter	Value
1	Comment	TKP-26
2	Instrument	JNM-ECZ600R/S1
3	Solvent	NONE
4	Temperature	22.7
5	Number of Scans	16
6	Spectrometer Frequency	600.17
7	Spectral Width	9025.3
8	Lowest Frequency	-1259.6
9	Nucleus	1H
10	Digital Resolution	0.09

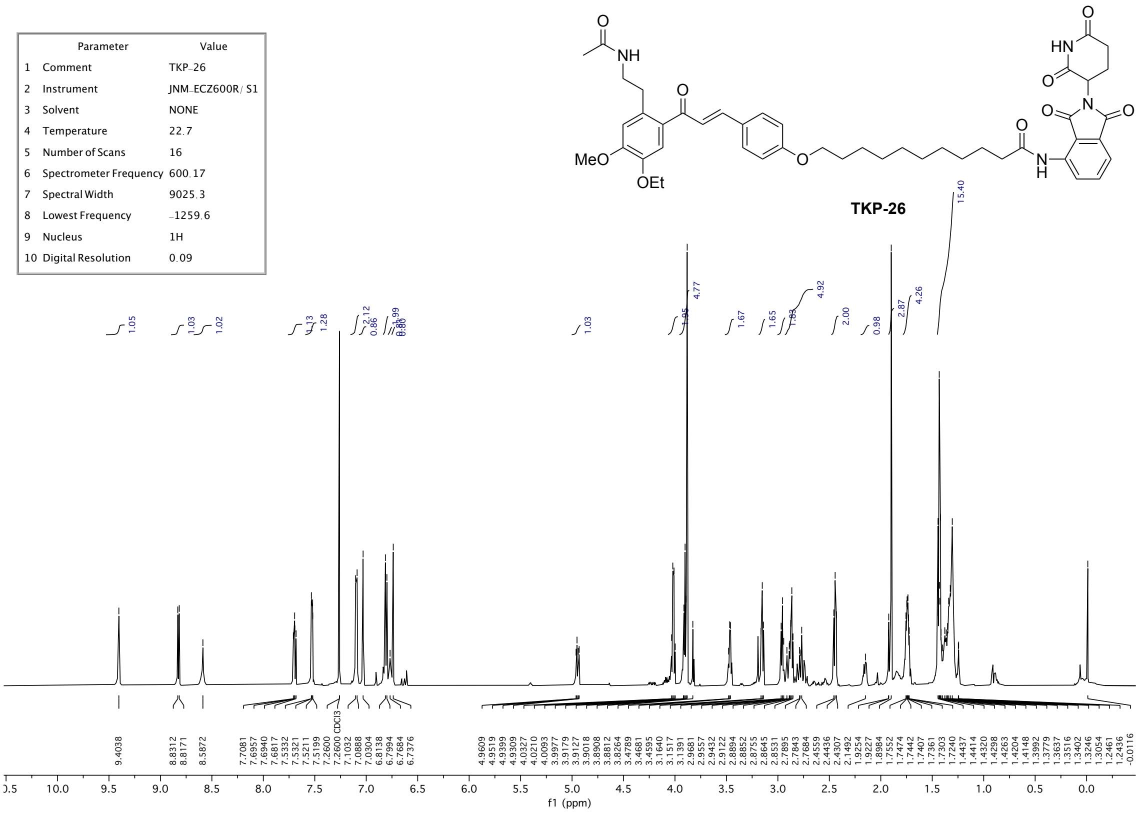

^{1}H NMR spectrum of **TKP-26**

	Parameter	Value
1	Comment	TKP-26
2	Instrument	JNM-ECZ600R/S1
3	Solvent	NONE
4	Temperature	23.6
5	Number of Scans	512
6	Spectrometer Frequency	150.91
7	Spectral Width	37878.8
8	Lowest Frequency	-3784.5
9	Nucleus	13C
10	Digital Resolution	0.36

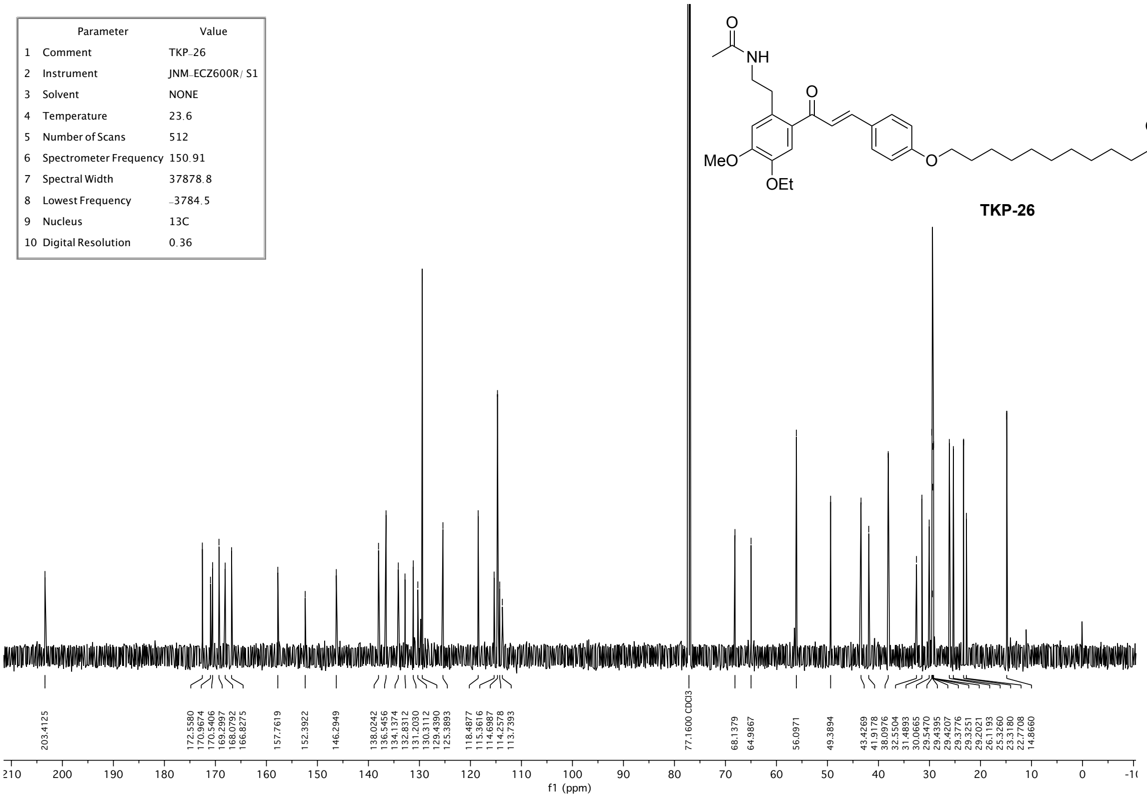

^{13}C NMR spectrum of **TKP-26**

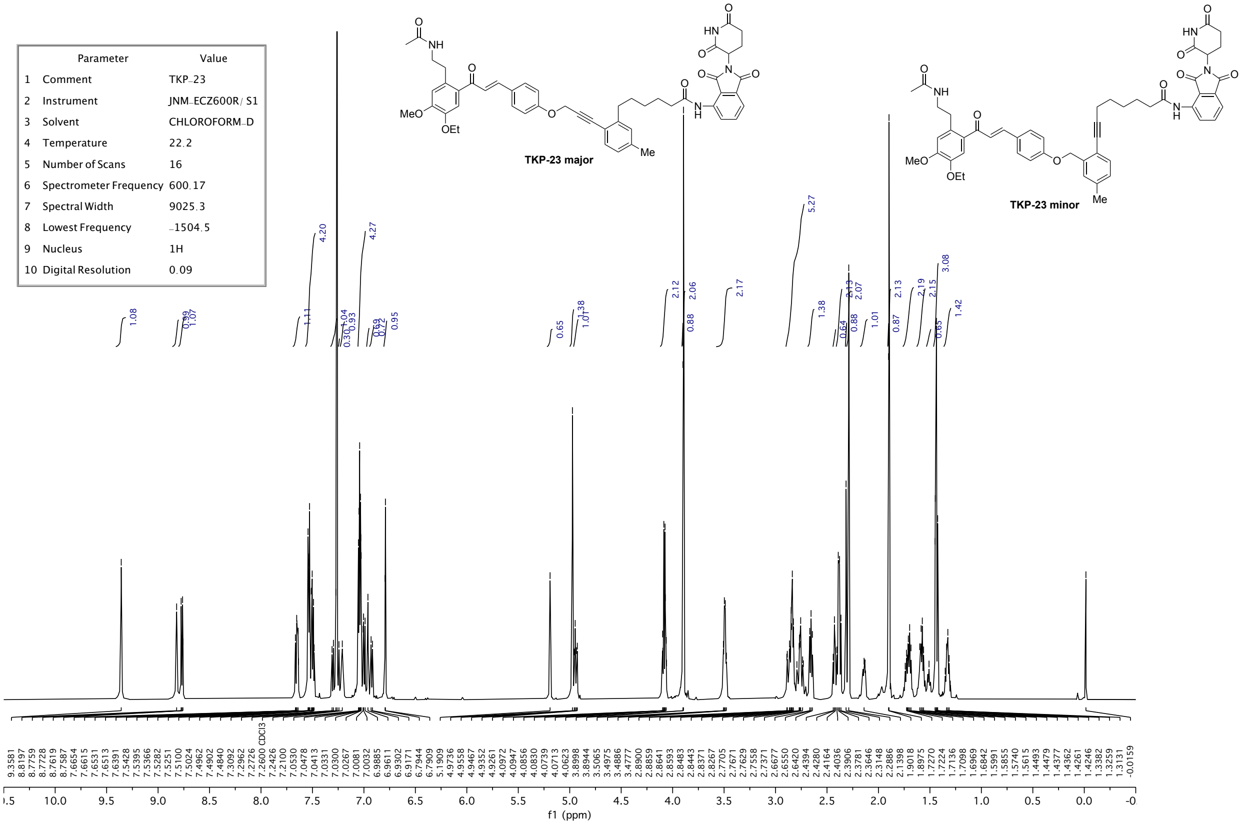

^{1}H NMR spectrum of **TKP-23**

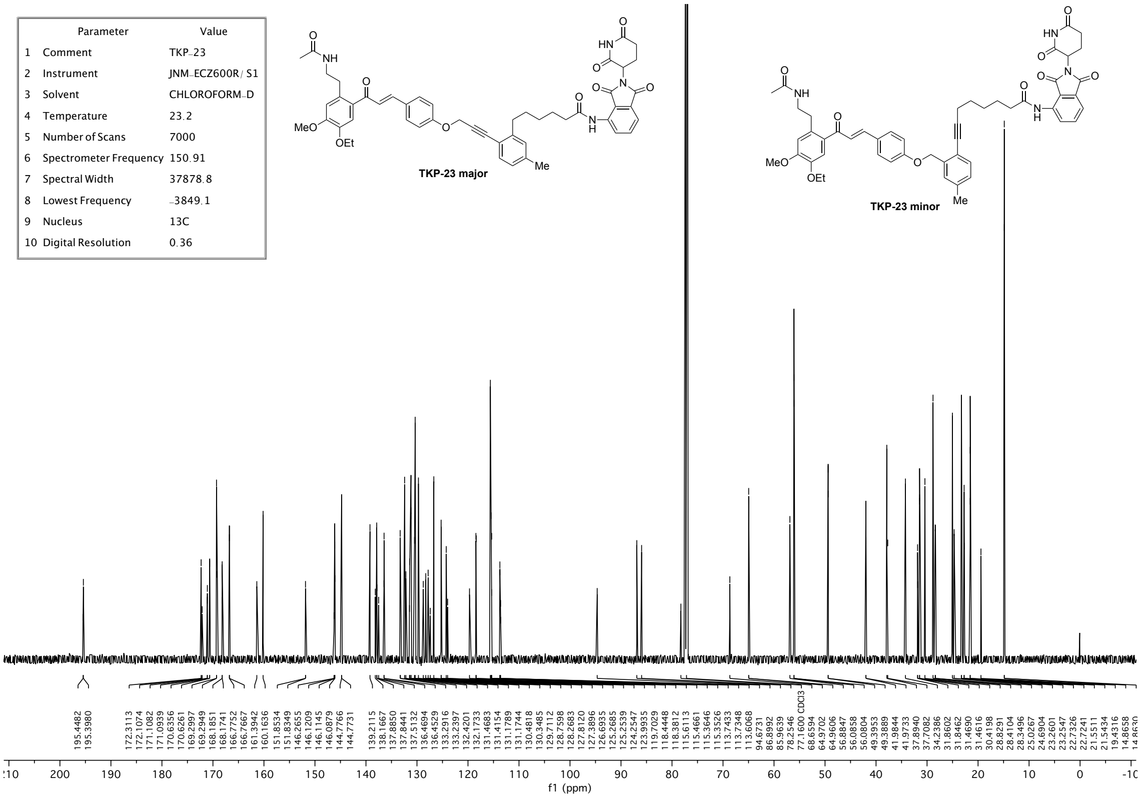

^{13}C NMR spectrum of **TKP-23**

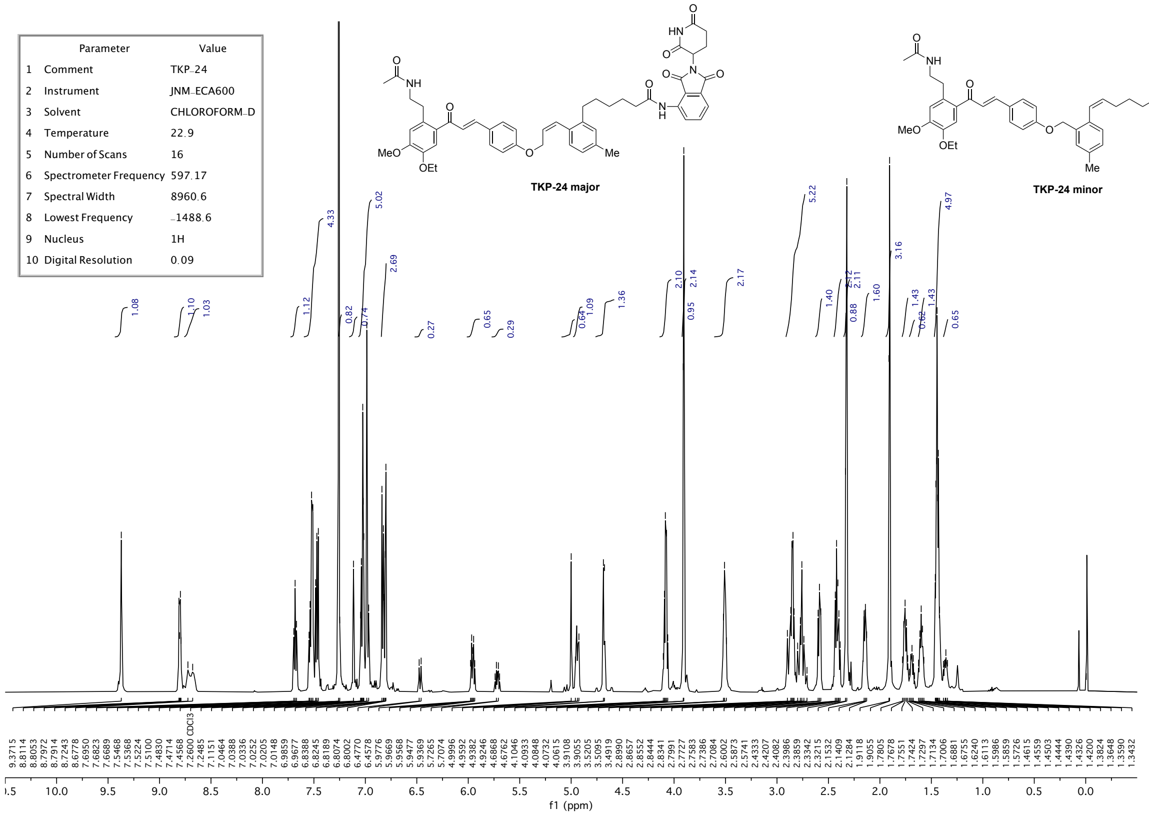

^{1}H NMR spectrum of **TKP-24**

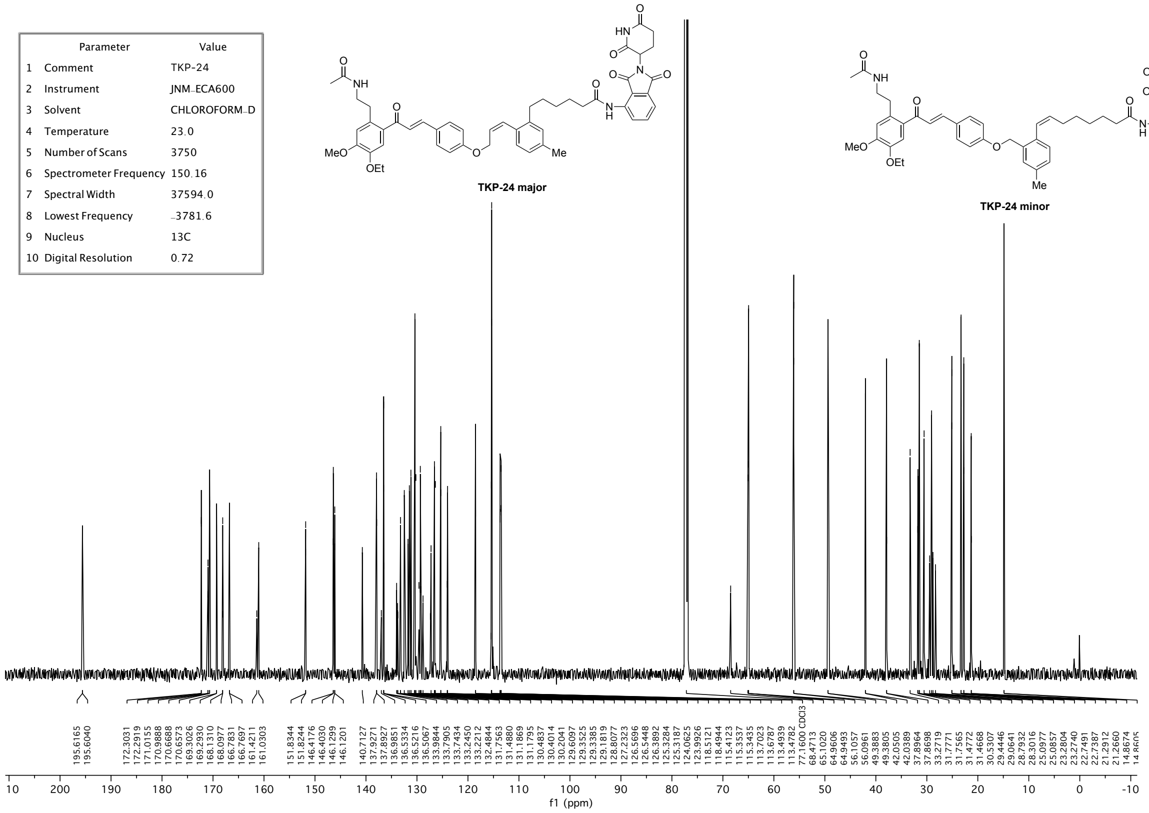

^{13}C NMR spectrum of **TKP-24**

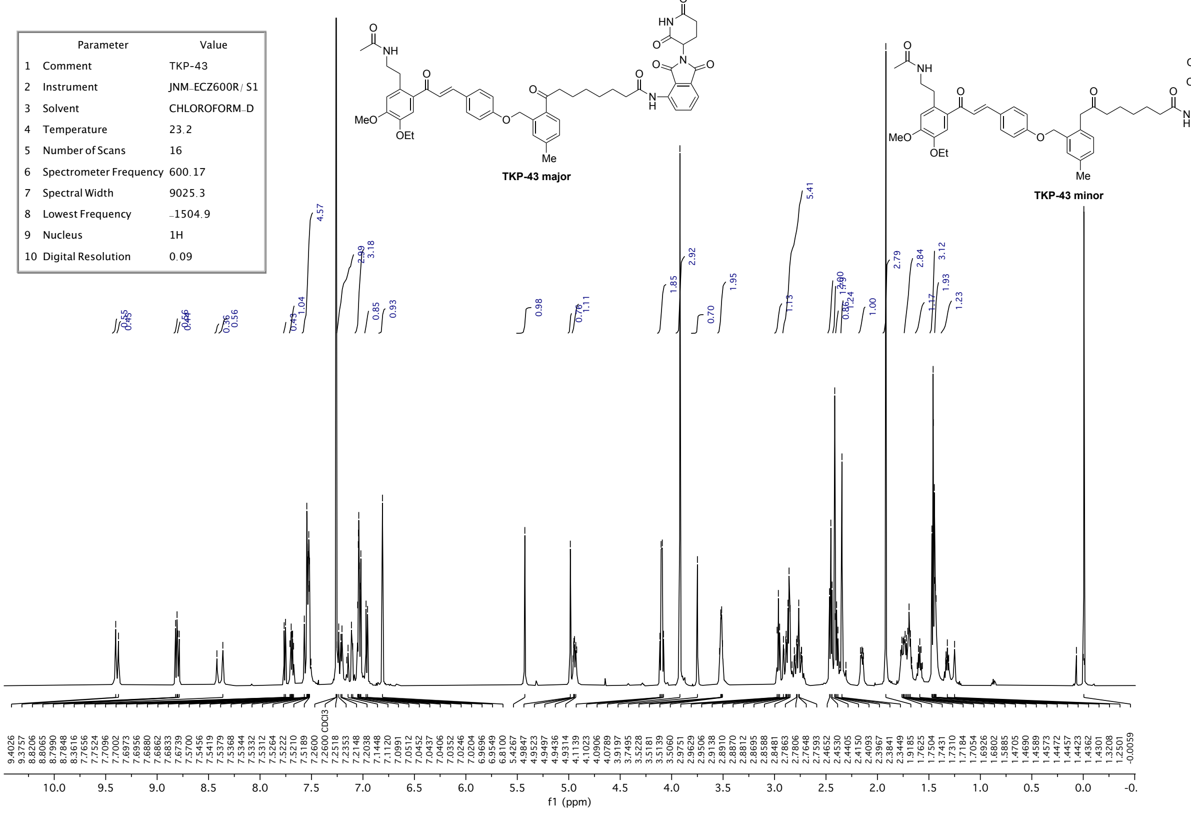

^{1}H NMR spectrum of **TKP-43**

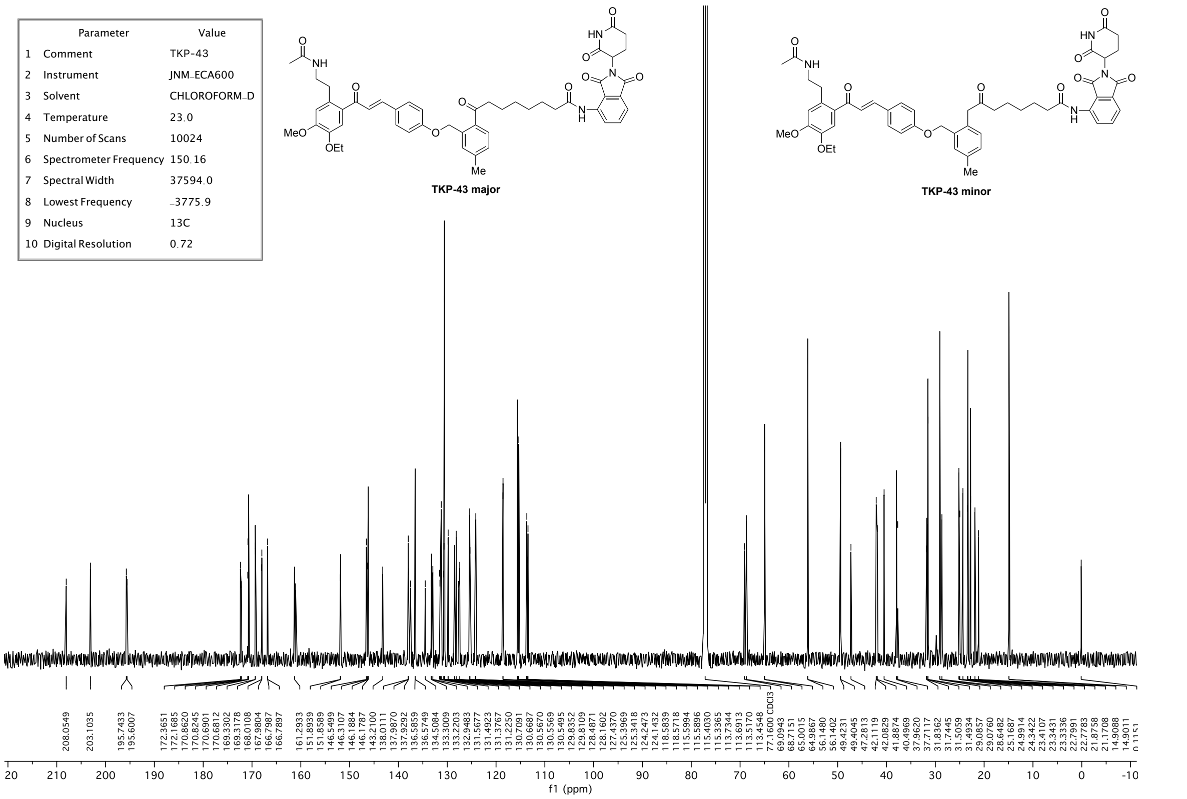

^{13}C NMR spectrum of **TKP-43**

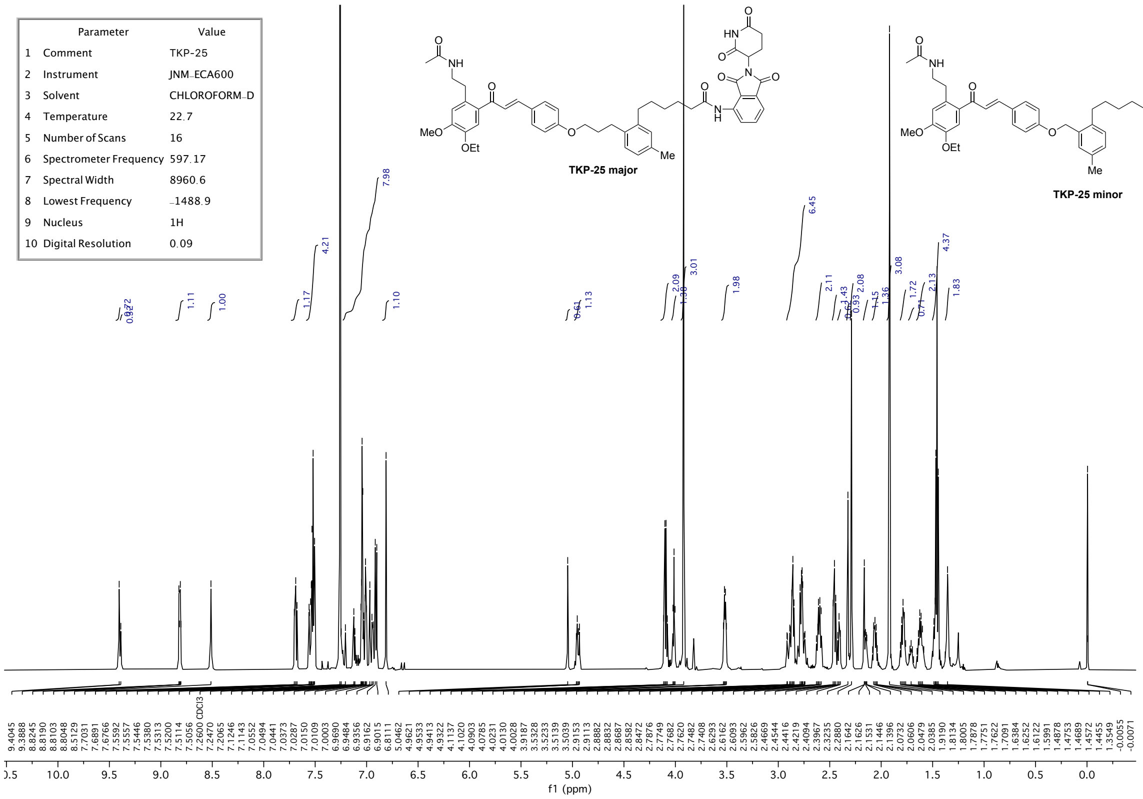

	Parameter	Value
1	Comment	TKP-25
2	Instrument	JNM-ECA600
3	Solvent	CHLOROFORM-D
4	Temperature	22.7
5	Number of Scans	16
6	Spectrometer Frequency	597.17
7	Spectral Width	8960.6
8	Lowest Frequency	-1488.9
9	Nucleus	1H
10	Digital Resolution	0.09

^{1}H NMR spectrum of **TKP-25**

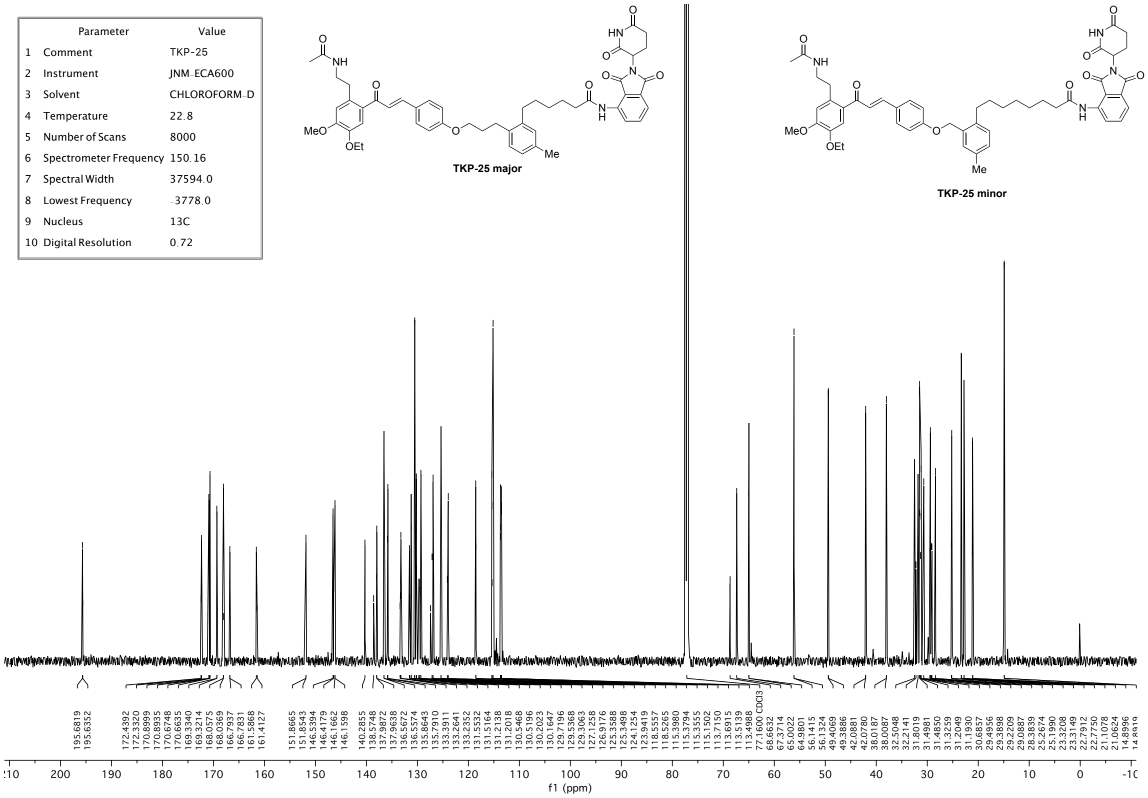

	Parameter	Value
1	Comment	TKP-25
2	Instrument	JNM-ECA600
3	Solvent	CHLOROFORM-D
4	Temperature	22.8
5	Number of Scans	8000
6	Spectrometer Frequency	150.16
7	Spectral Width	37594.0
8	Lowest Frequency	-3778.0
9	Nucleus	13C
10	Digital Resolution	0.72

^{13}C NMR spectrum of **TKP-25**

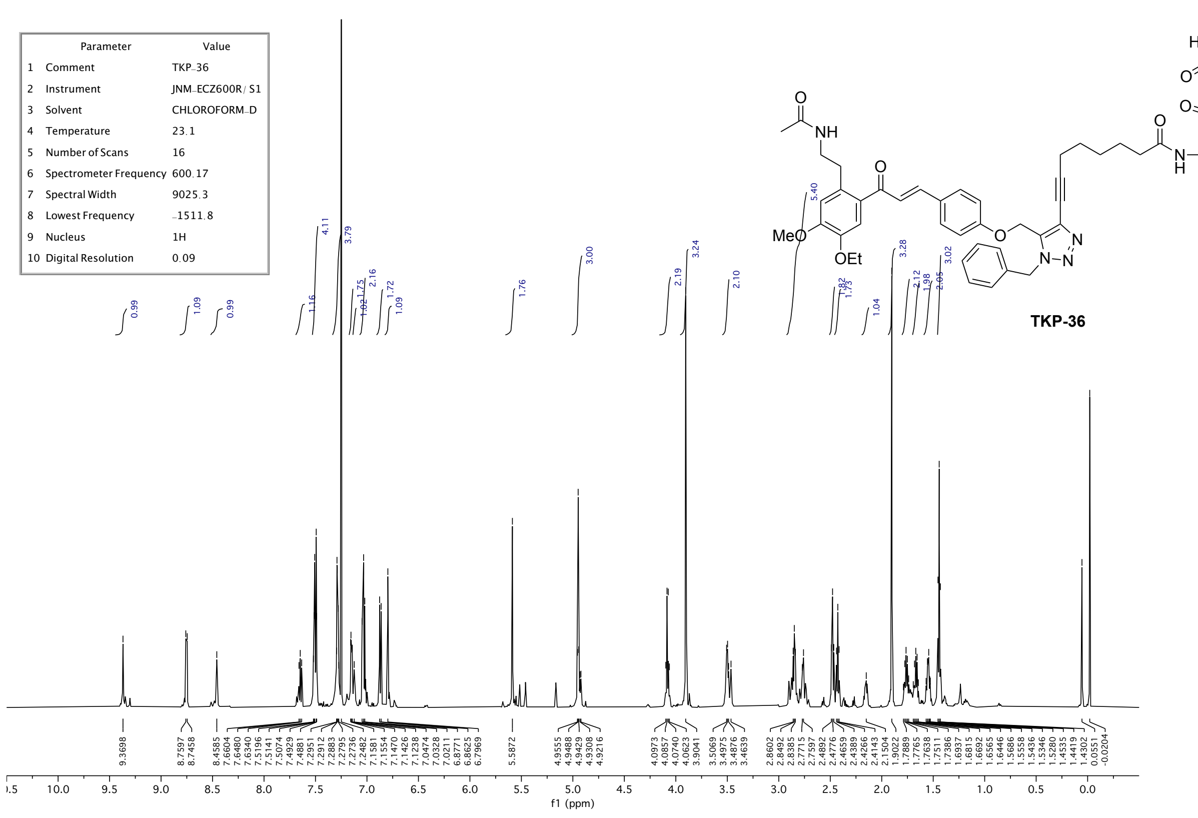

^{1}H NMR spectrum of **TKP-36**

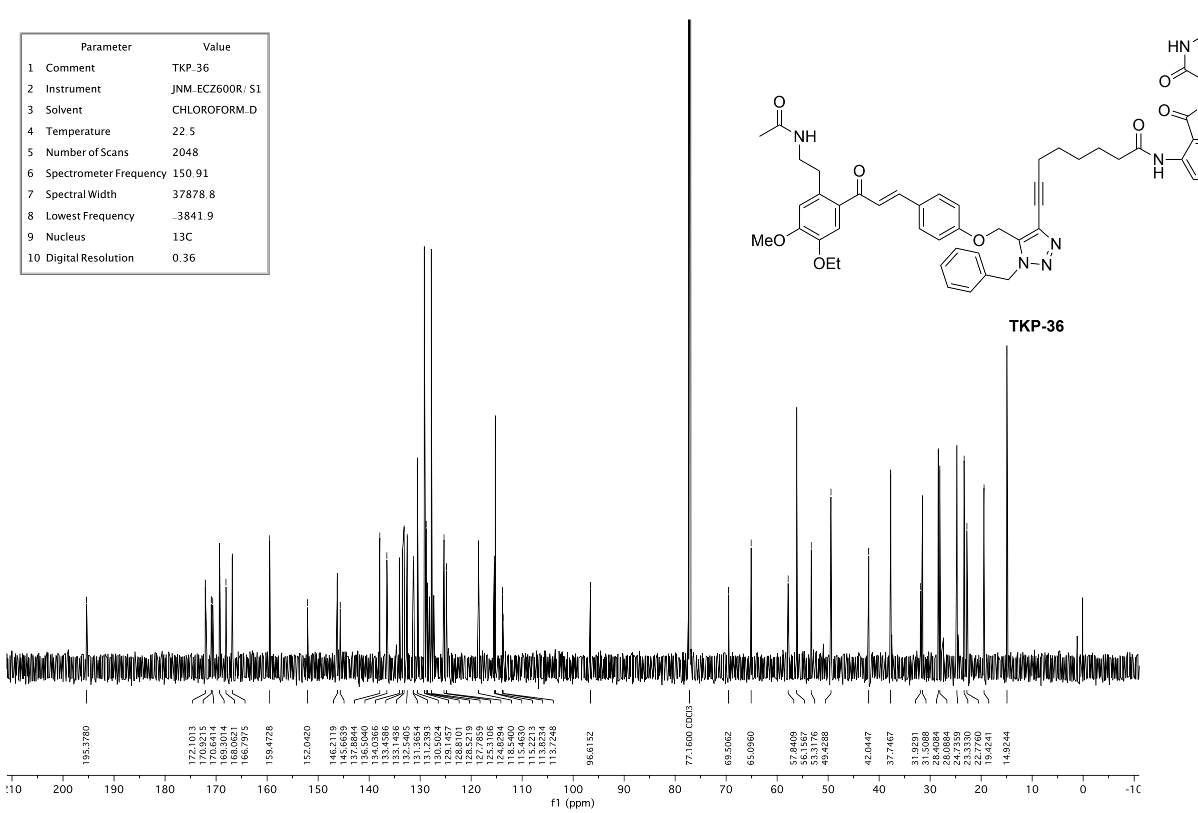

^{13}C NMR spectrum of **TKP-36**

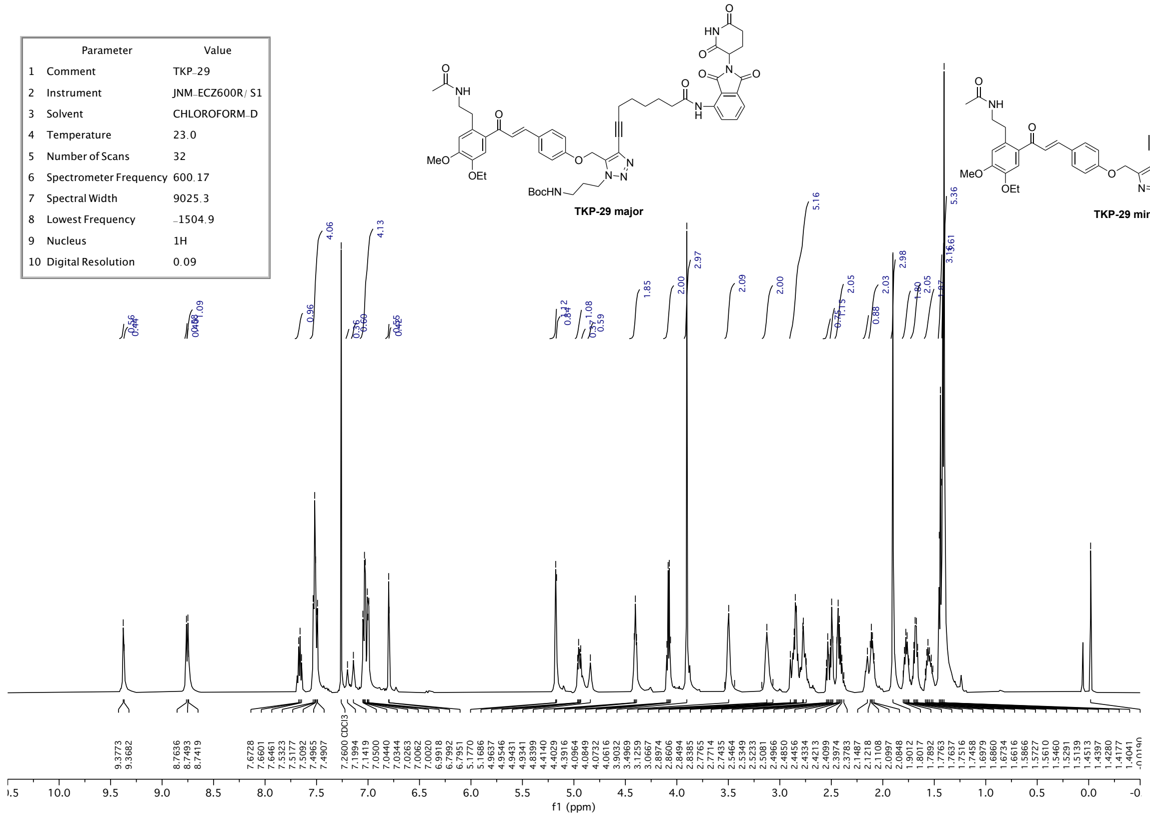

^{1}H NMR spectrum of **TKP-29**

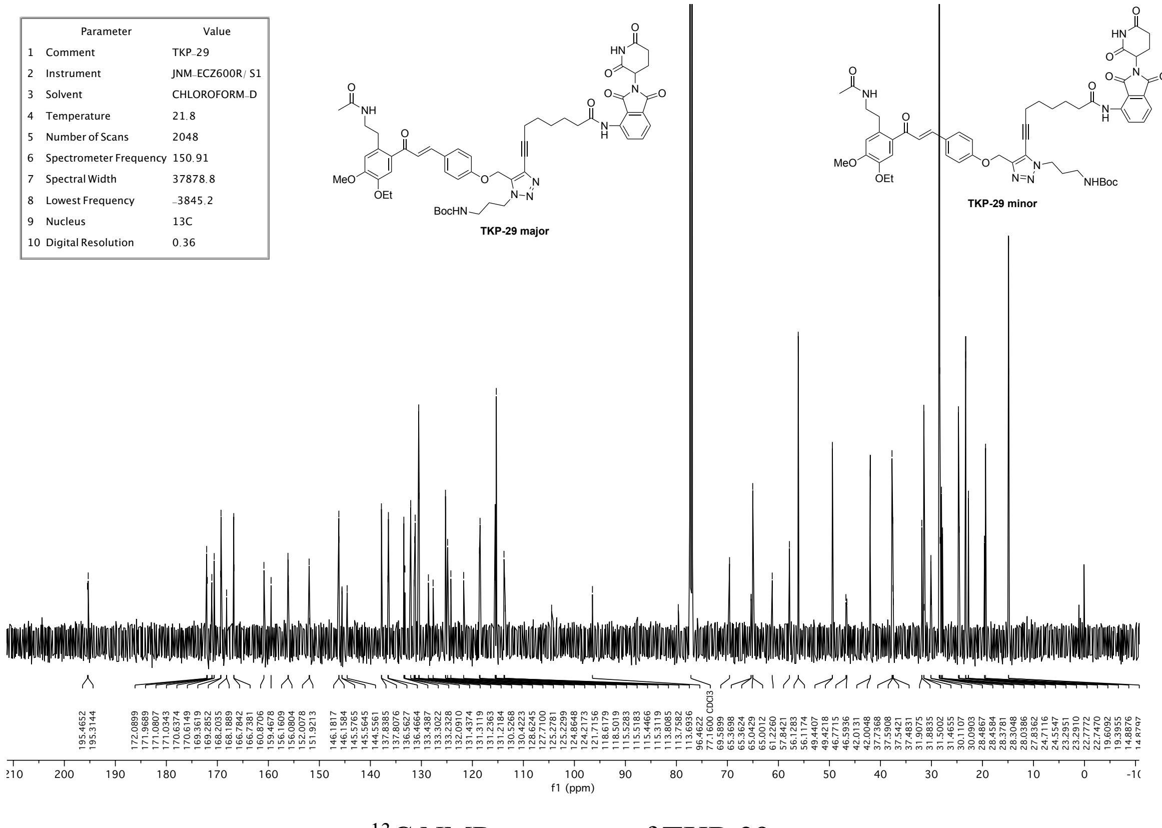

^{13}C NMR spectrum of **TKP-29**

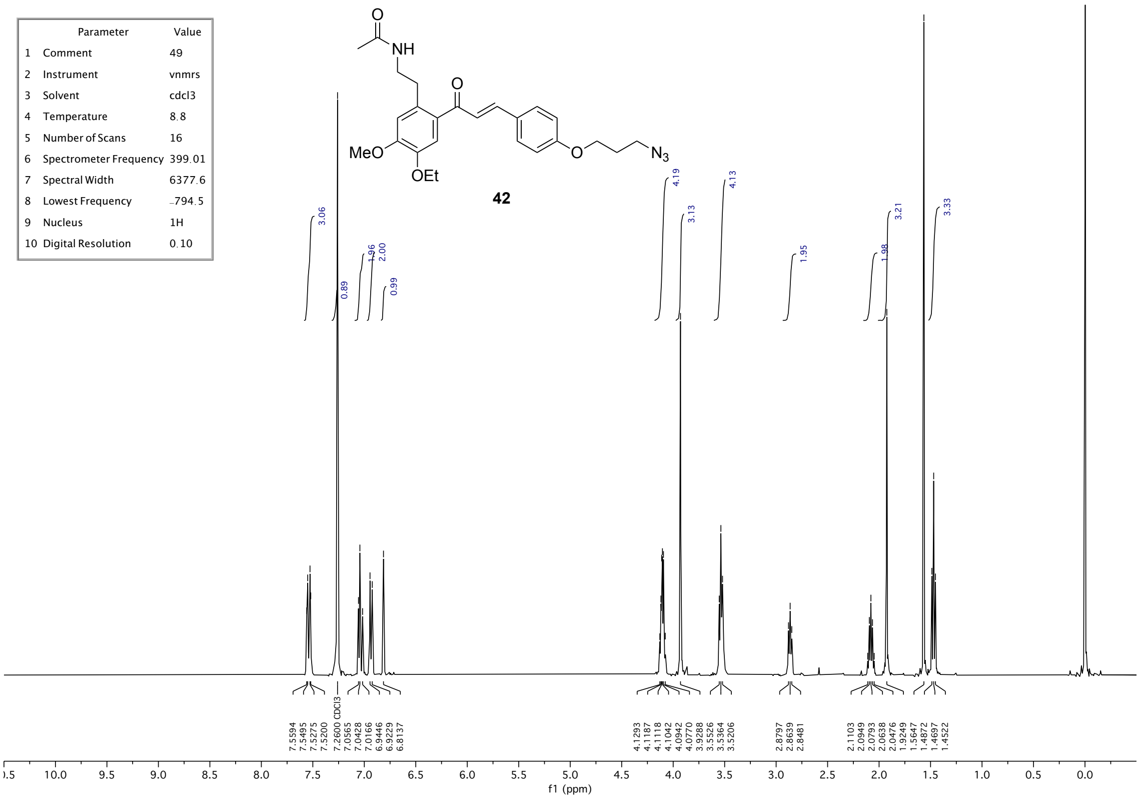

	Parameter	Value
1	Comment	49
2	Instrument	vnmrs
3	Solvent	cdcl3
4	Temperature	8.8
5	Number of Scans	16
6	Spectrometer Frequency	399.01
7	Spectral Width	6377.6
8	Lowest Frequency	-794.5
9	Nucleus	1H
10	Digital Resolution	0.10

^{1}H NMR spectrum of compound **42**

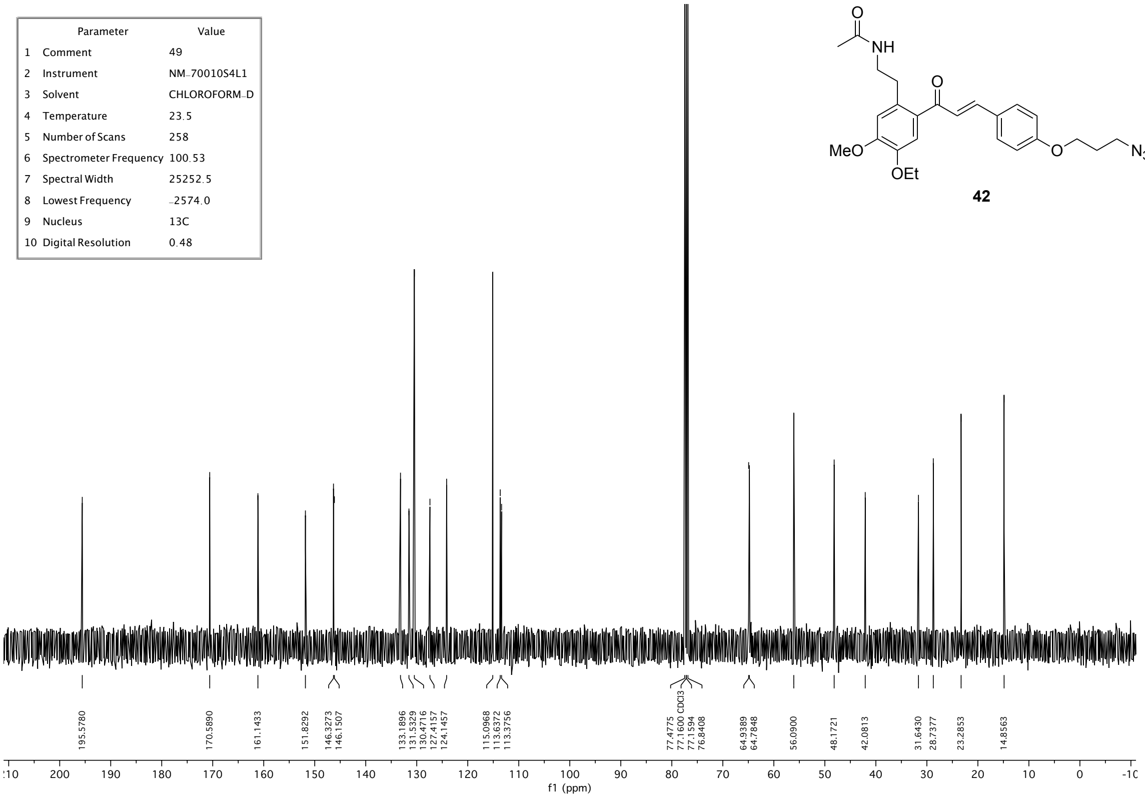

	Parameter	Value
1	Comment	49
2	Instrument	NM-70010S4L1
3	Solvent	CHLOROFORM-D
4	Temperature	23.5
5	Number of Scans	258
6	Spectrometer Frequency	100.53
7	Spectral Width	25252.5
8	Lowest Frequency	-2574.0
9	Nucleus	13C
10	Digital Resolution	0.48

^{13}C NMR spectrum of compound **42**

	Parameter	Value
1	Comment	TKP_27
2	Instrument	JNM_ECA600
3	Solvent	CHLOROFORM_D
4	Temperature	22.9
5	Number of Scans	8
6	Spectrometer Frequency	597.17
7	Spectral Width	8960.6
8	Lowest Frequency	_1489.0
9	Nucleus	1H
10	Digital Resolution	0.09

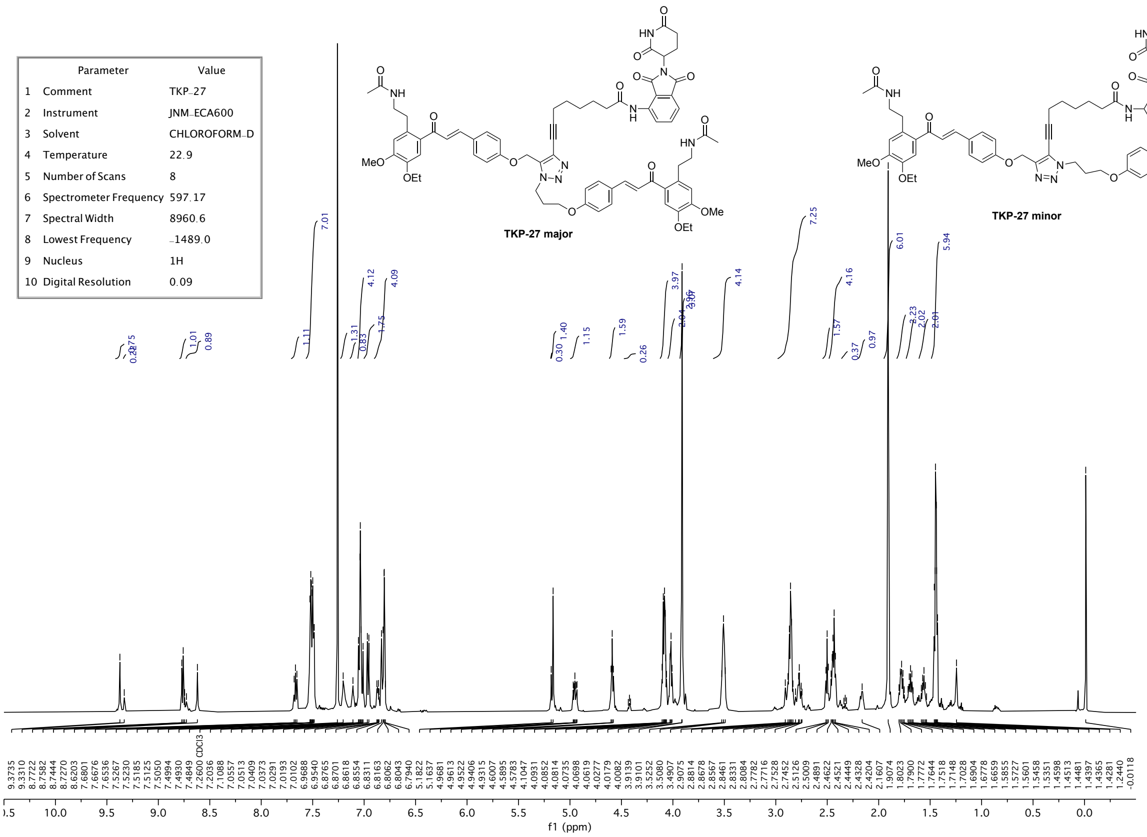

^{1}H NMR spectrum of **TKP-27**

	Parameter	Value
1	Comment	TKP_27
2	Instrument	JNM_ECA600
3	Solvent	CHLOROFORM_D
4	Temperature	23.0
5	Number of Scans	6500
6	Spectrometer Frequency	150.16
7	Spectral Width	37594.0
8	Lowest Frequency	_3777.3
9	Nucleus	13C
10	Digital Resolution	0.72

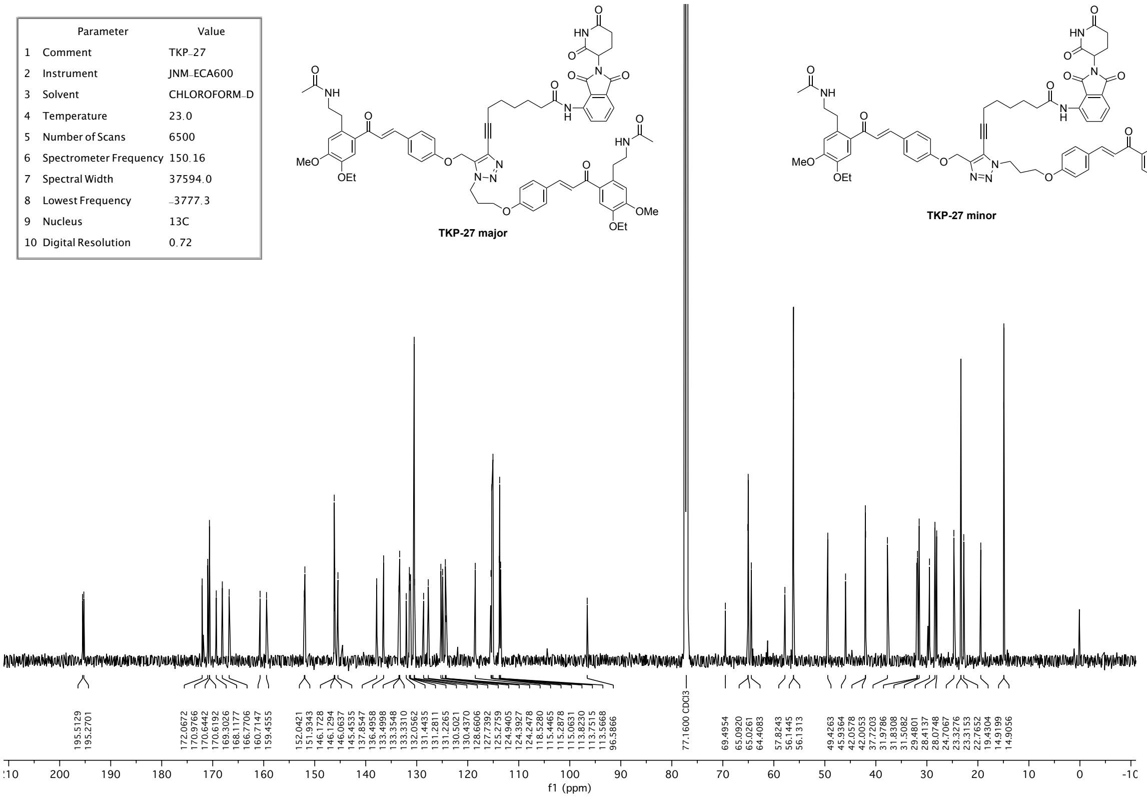

^{13}C NMR spectrum of **TKP-27**

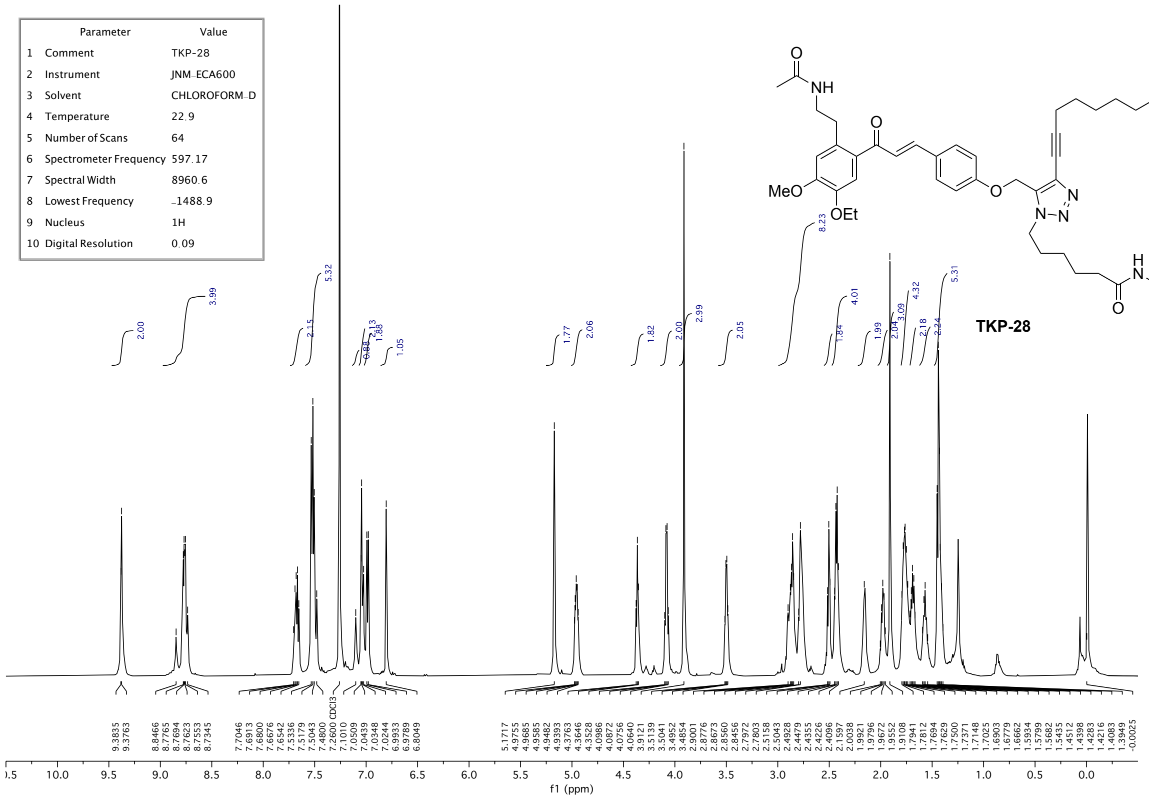

^{1}H NMR spectrum of **TKP-28**

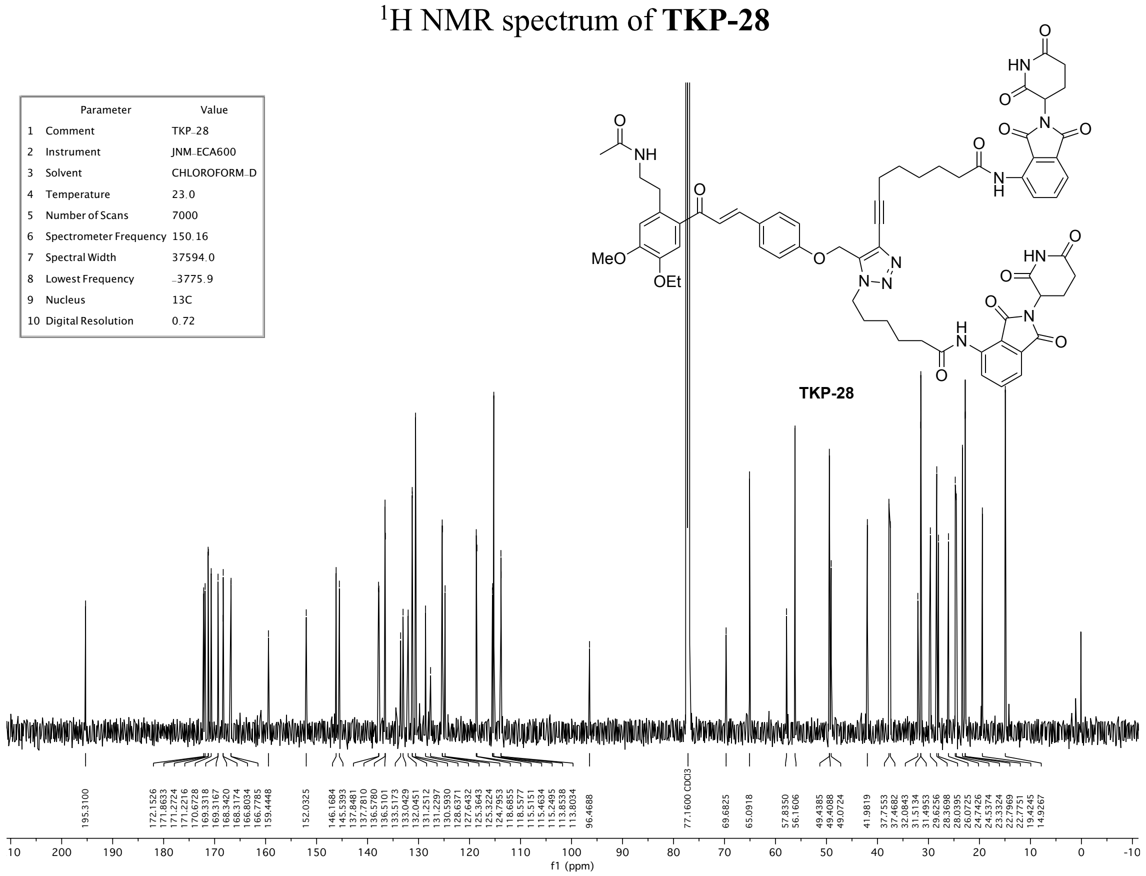

^{13}C NMR spectrum of **TKP-28**

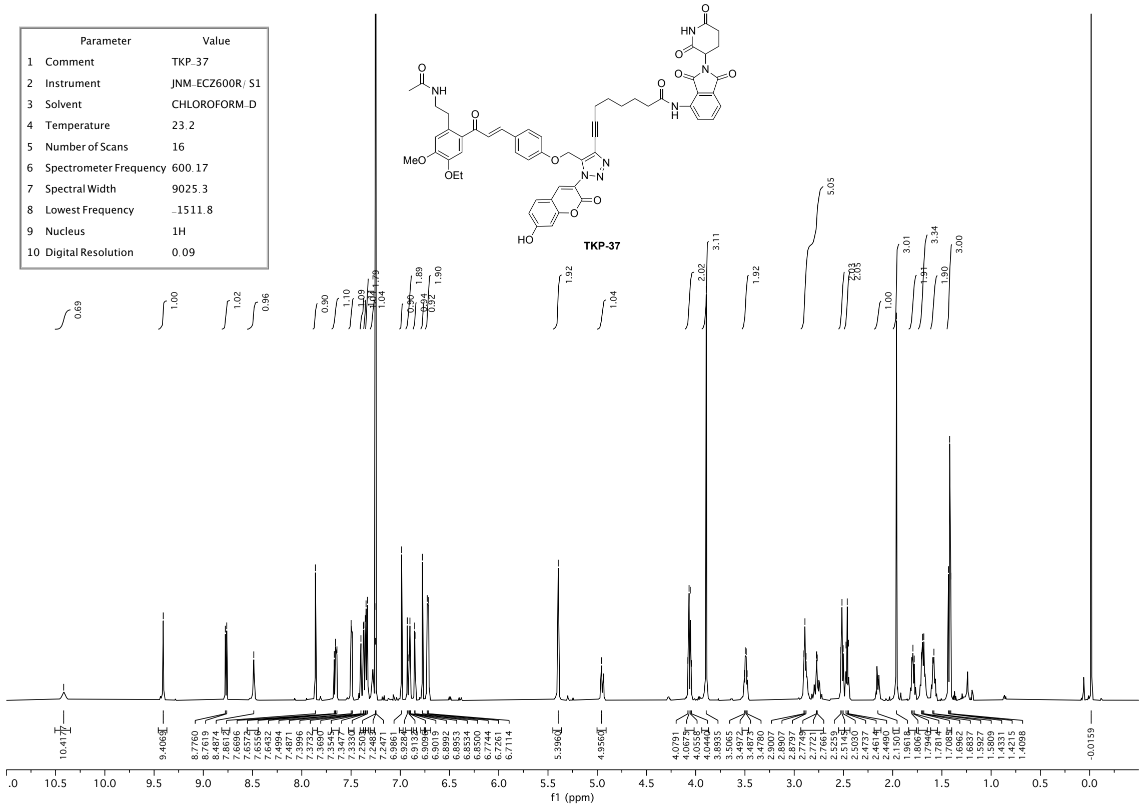

^{1}H NMR spectrum of **TKP-37**

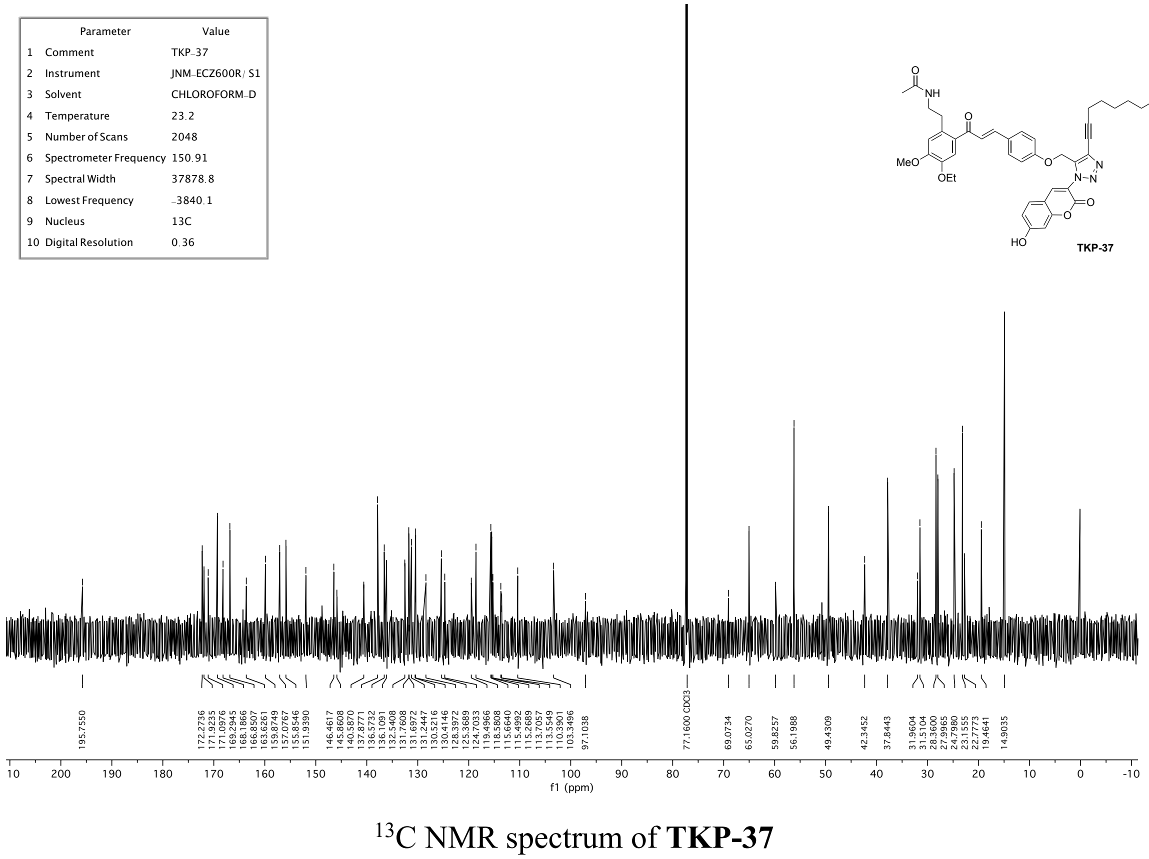

^{13}C NMR spectrum of **TKP-37**

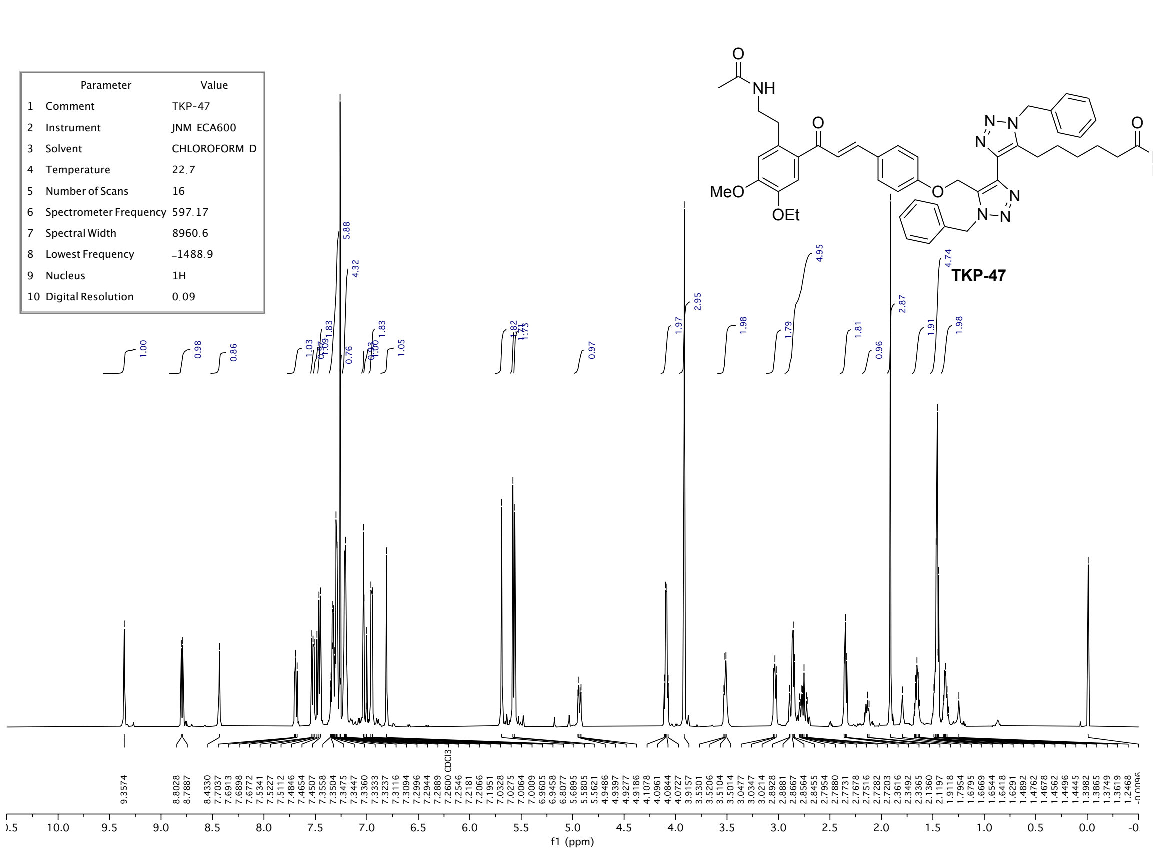

^{1}H NMR spectrum of **TKP-47**

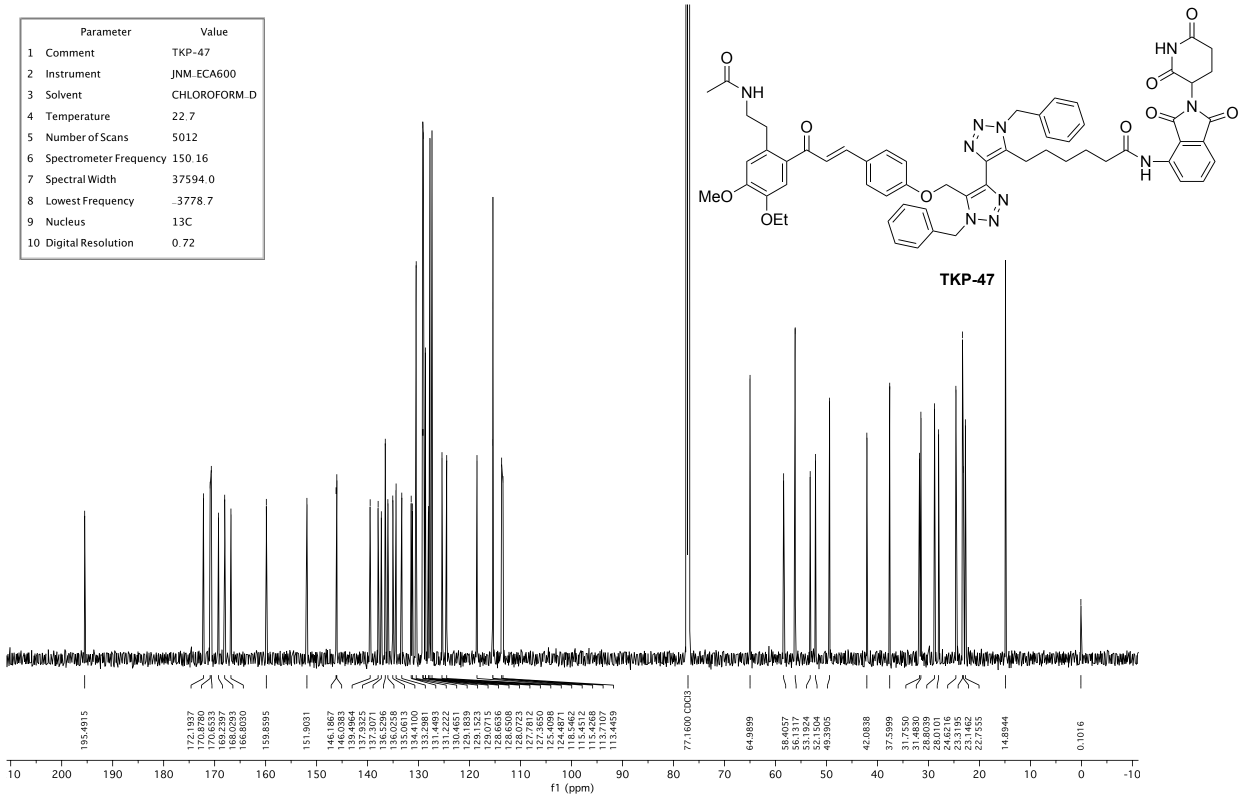

^{13}C NMR spectrum of **TKP-47**

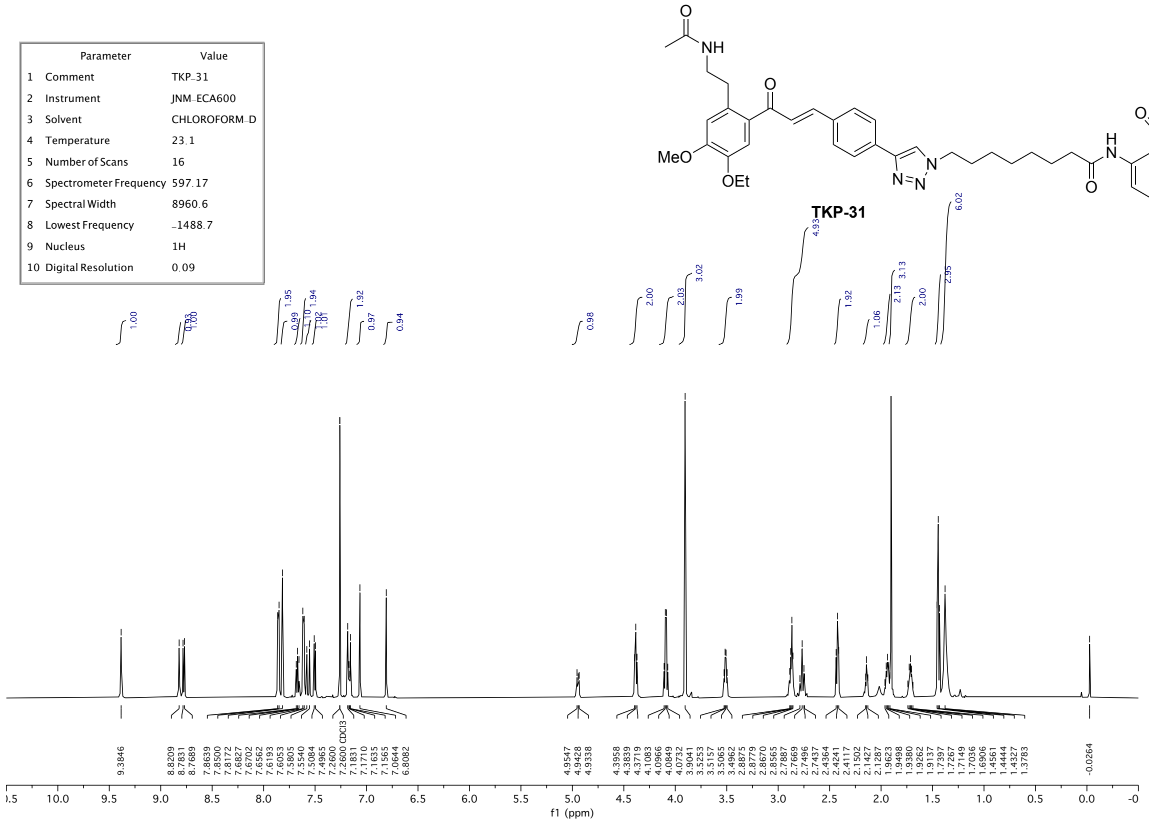

^{1}H NMR spectrum of **TKP-31**

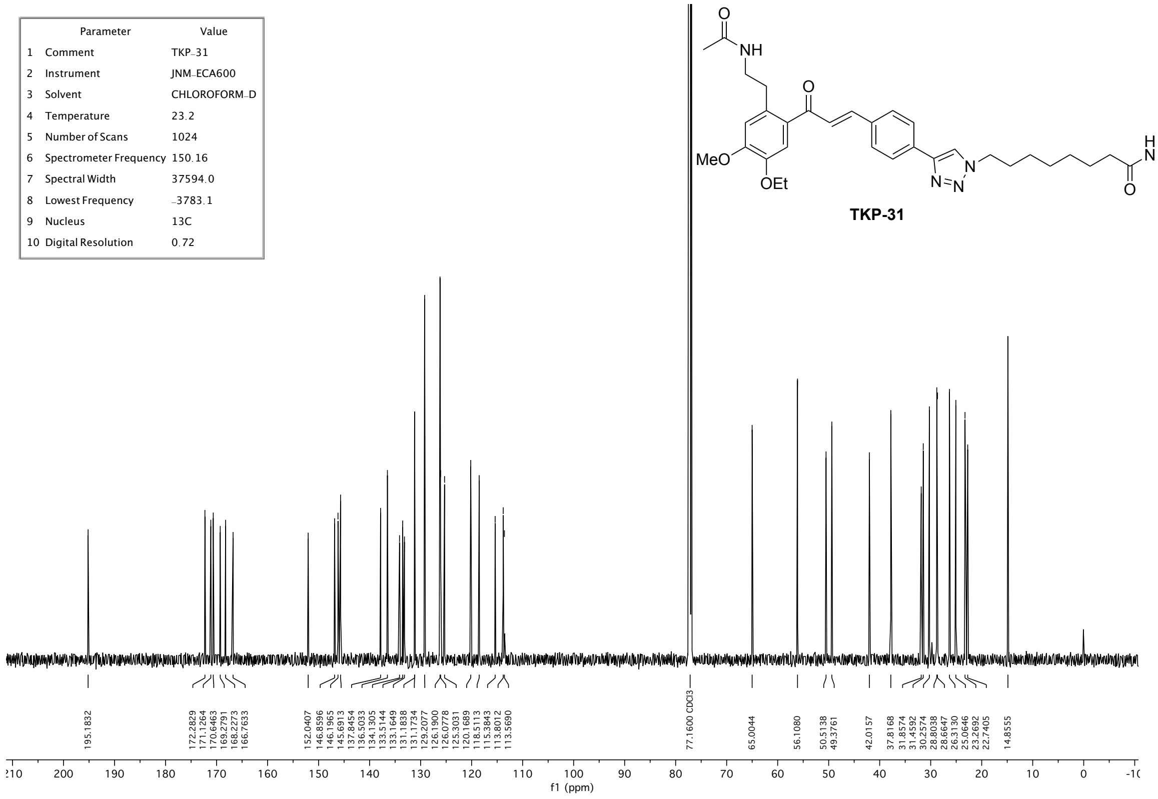

^{13}C NMR spectrum of **TKP-31**

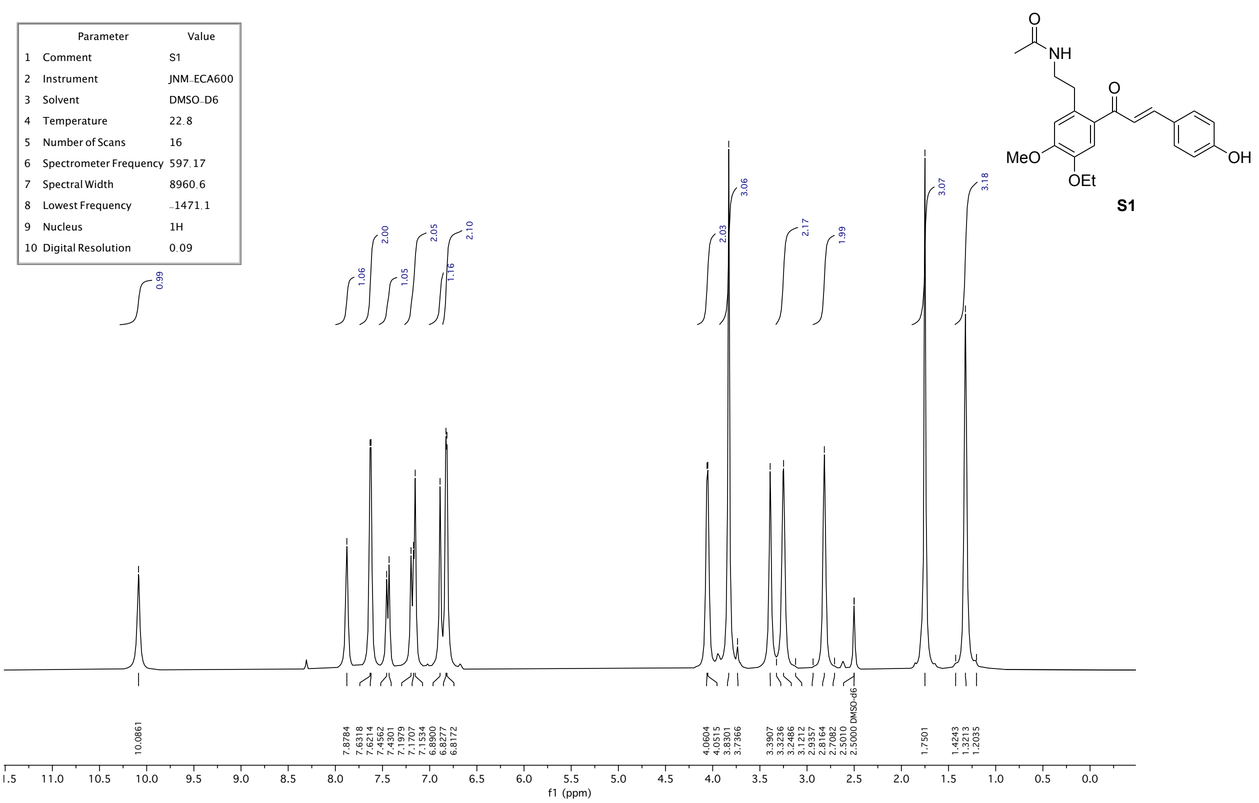

^{1}H NMR spectrum of compound **S1**

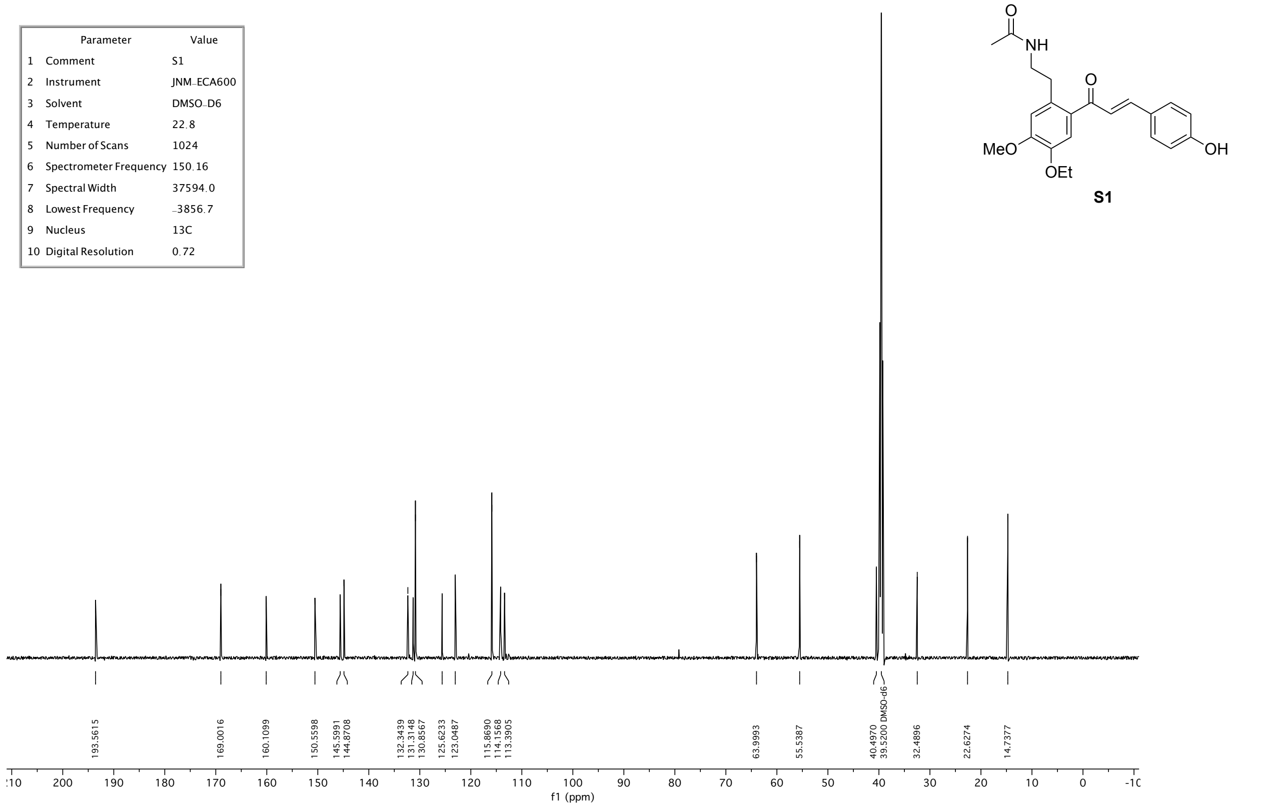

^{13}C NMR spectrum of compound **S1**

	Parameter	Value
1	Comment	S2
2	Instrument	JNM-ECA600
3	Solvent	CHLOROFORM-D
4	Temperature	23.2
5	Number of Scans	16
6	Spectrometer Frequency	597.17
7	Spectral Width	8960.6
8	Lowest Frequency	-1488.7
9	Nucleus	1H
10	Digital Resolution	0.09

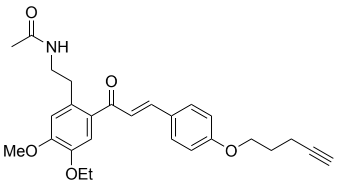

S2

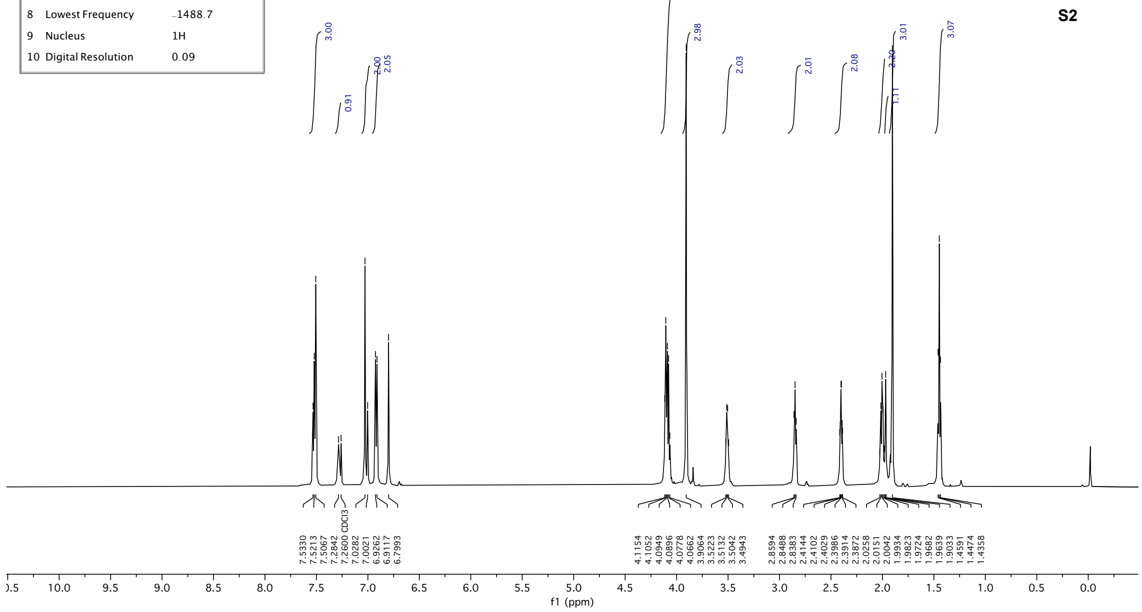

^{1}H NMR spectrum of compound **S2**

	Parameter	Value
1	Comment	S2
2	Instrument	JNM-ECA600
3	Solvent	CHLOROFORM-D
4	Temperature	23.2
5	Number of Scans	266
6	Spectrometer Frequency	150.16
7	Spectral Width	37594.0
8	Lowest Frequency	-3780.9
9	Nucleus	13C
10	Digital Resolution	0.72

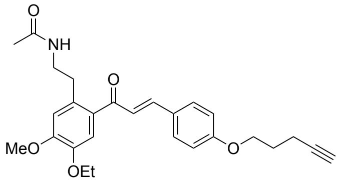

S2

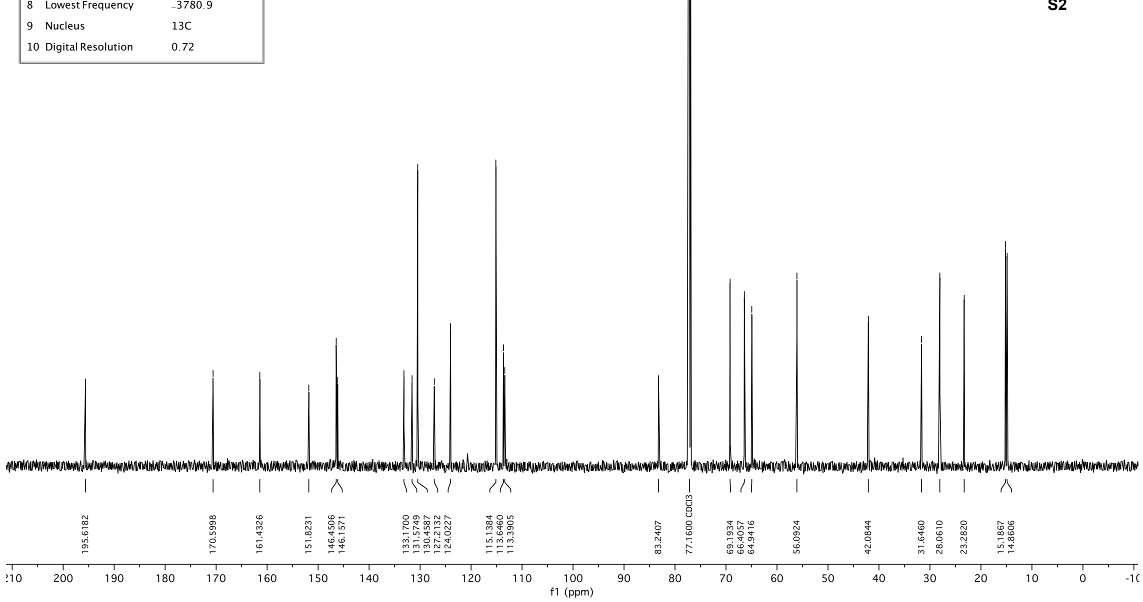

^{13}C NMR spectrum of compound **S2**

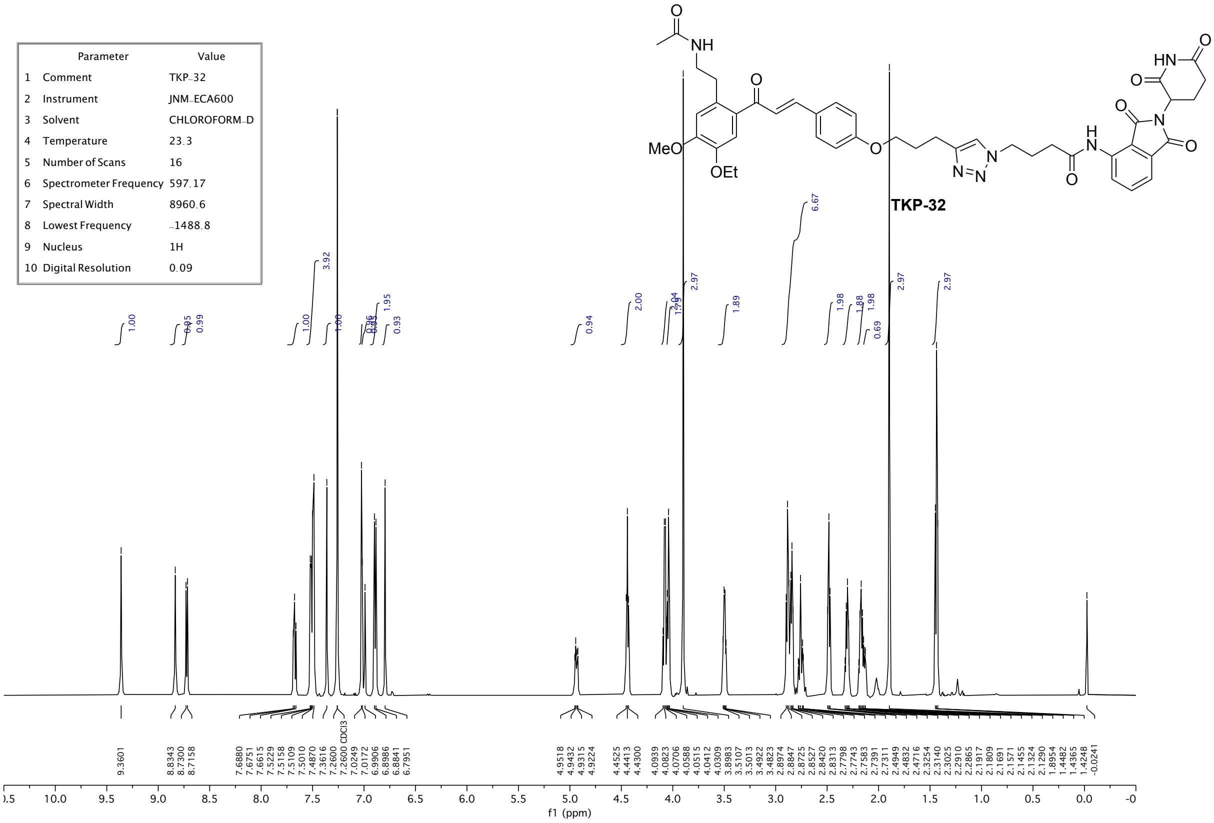

^1H NMR spectrum of **TKP-32**

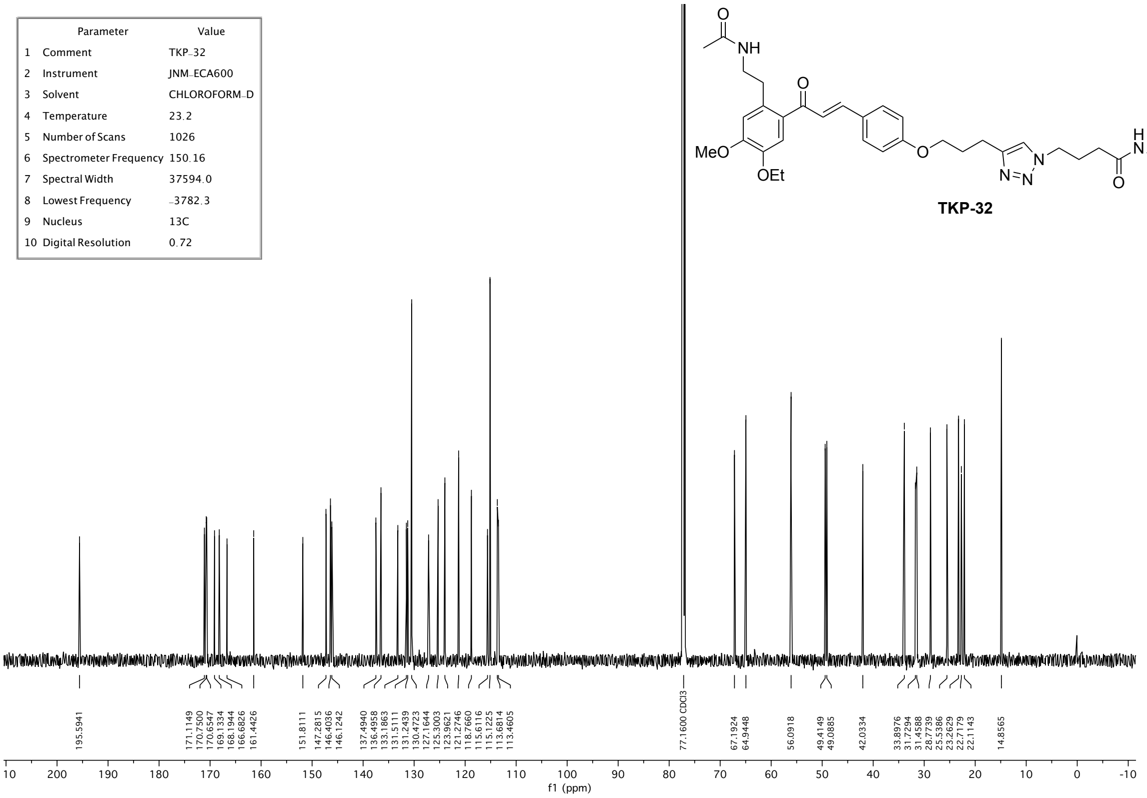

^{13}C NMR spectrum of **TKP-32**

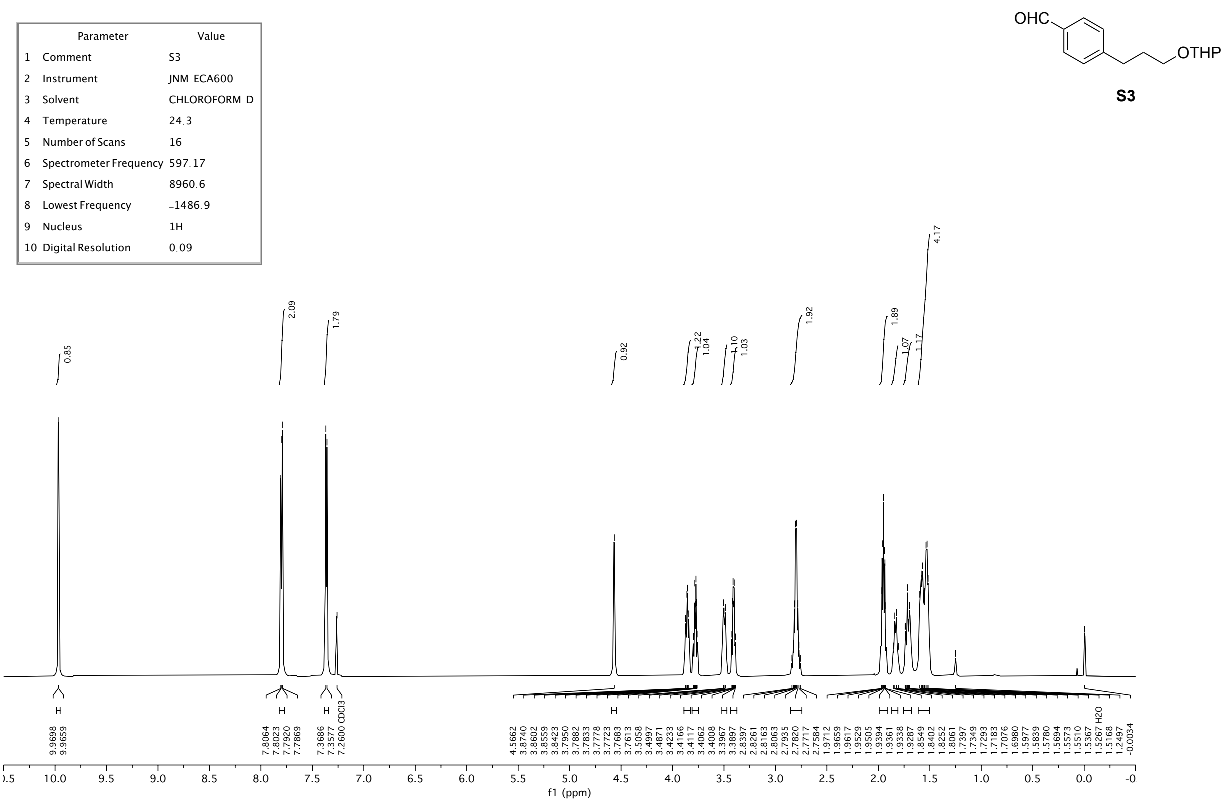

^{1}H NMR spectrum of compound **S3**

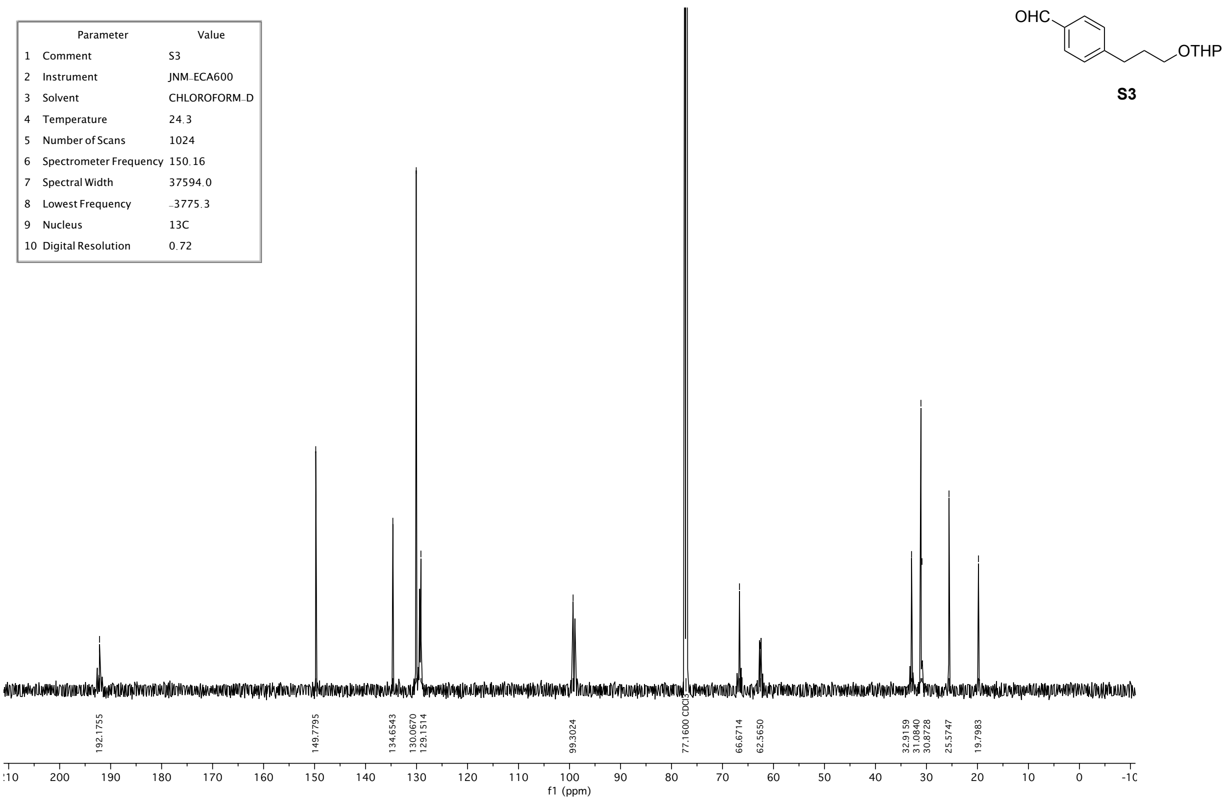

^{13}C NMR spectrum of compound **S3**

	Parameter	Value
1	Comment	S4
2	Instrument	JNM-ECZ600R/S1
3	Solvent	CHLOROFORM-D
4	Temperature	22.7
5	Number of Scans	16
6	Spectrometer Frequency	600.17
7	Spectral Width	9025.3
8	Lowest Frequency	-1504.1
9	Nucleus	1H
10	Digital Resolution	0.09

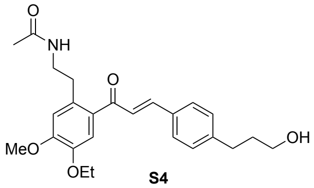

S4

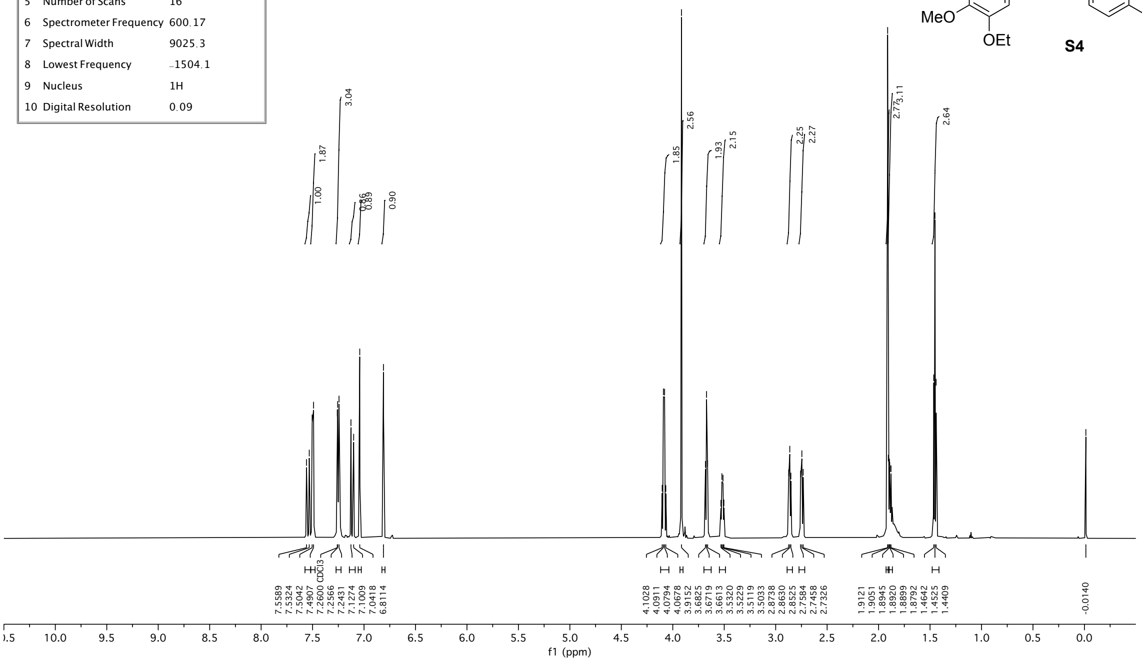

^{1}H NMR spectrum of compound **S4**

	Parameter	Value
1	Comment	S4
2	Instrument	JNM-ECZ600R/S1
3	Solvent	CHLOROFORM-D
4	Temperature	22.7
5	Number of Scans	1024
6	Spectrometer Frequency	150.91
7	Spectral Width	37878.8
8	Lowest Frequency	-3844.1
9	Nucleus	13C
10	Digital Resolution	0.36

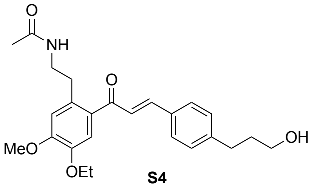

S4

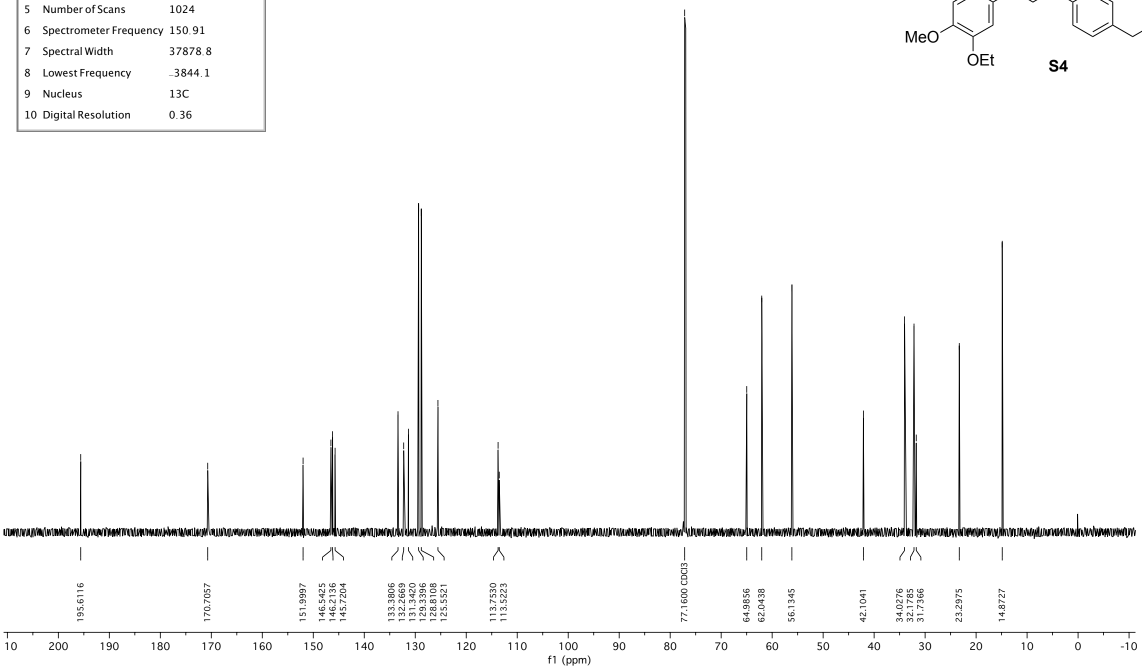

^{13}C NMR spectrum of compound **S4**

	Parameter	Value
1	Comment	S5
2	Instrument	JNM-ECZ600R/S1
3	Solvent	CHLOROFORM-D
4	Temperature	23.0
5	Number of Scans	16
6	Spectrometer Frequency	600.17
7	Spectral Width	9025.3
8	Lowest Frequency	-1504.3
9	Nucleus	1H
10	Digital Resolution	0.09

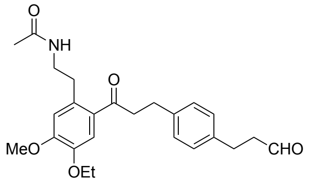

S5

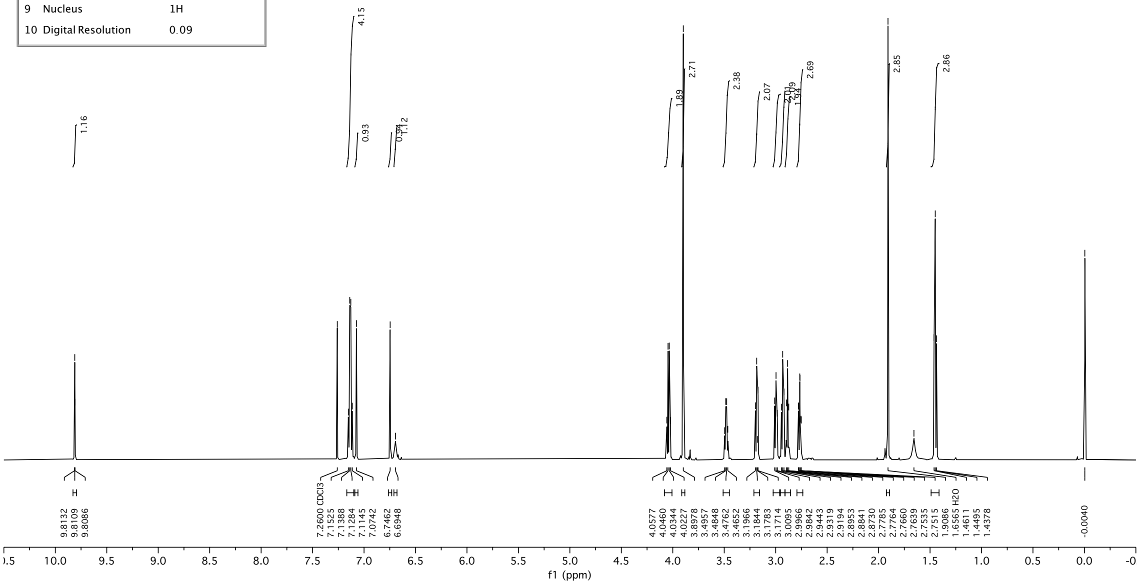

^{1}H NMR spectrum of compound **S5**

	Parameter	Value
1	Comment	S5
2	Instrument	JNM-ECZ600R/S1
3	Solvent	CHLOROFORM-D
4	Temperature	23.3
5	Number of Scans	1024
6	Spectrometer Frequency	150.91
7	Spectral Width	37878.8
8	Lowest Frequency	-3839.8
9	Nucleus	13C
10	Digital Resolution	0.36

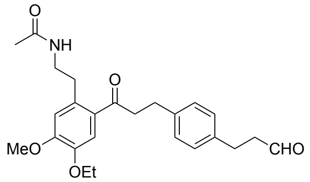

S5

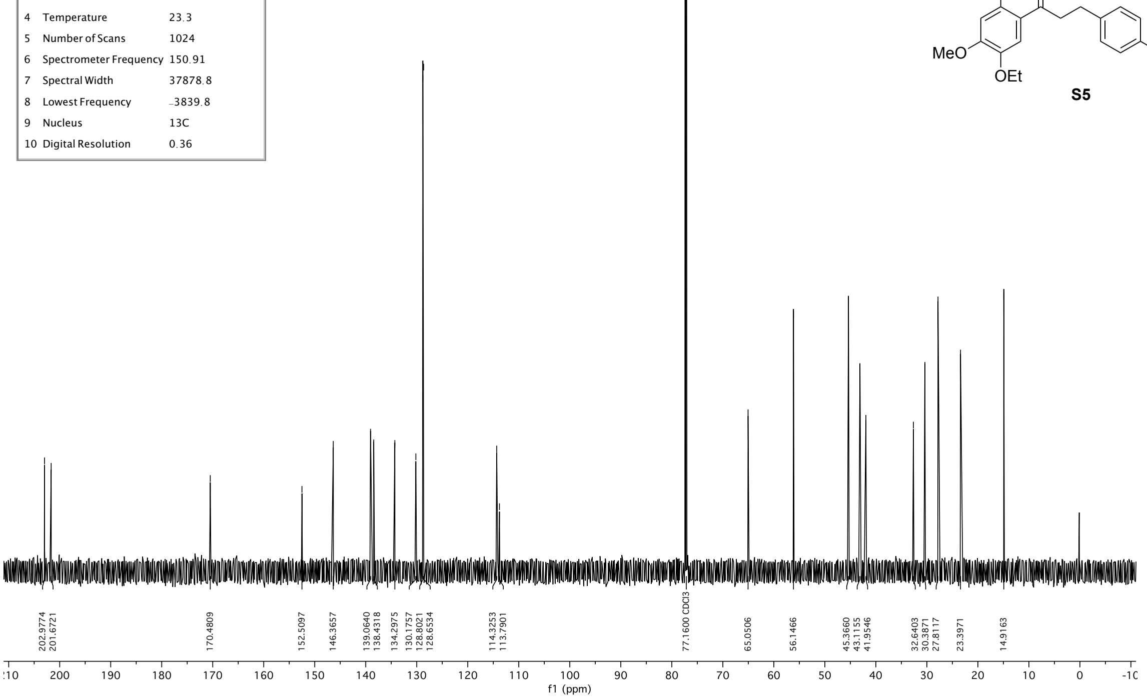

^{13}C NMR spectrum of compound **S5**

	Parameter	Value
1	Comment	S6
2	Instrument	JNM-ECA600
3	Solvent	CHLOROFORM-D
4	Temperature	24.3
5	Number of Scans	32
6	Spectrometer Frequency	597.17
7	Spectral Width	8960.6
8	Lowest Frequency	-1489.2
9	Nucleus	1H
10	Digital Resolution	0.09

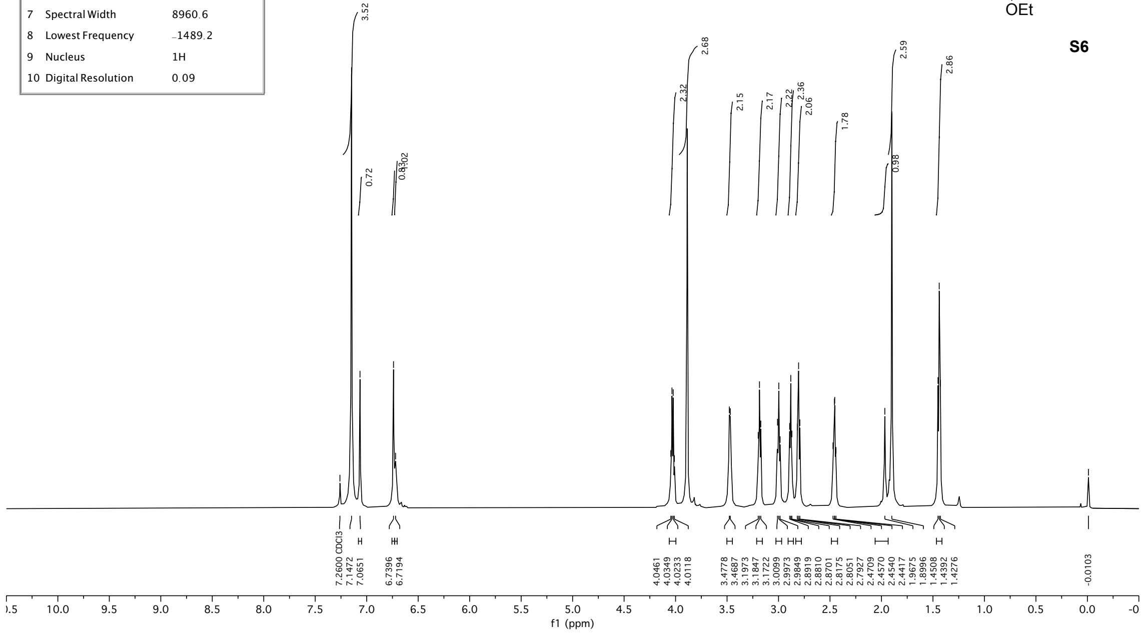

^{1}H NMR spectrum of compound **S6**

	Parameter	Value
1	Comment	S6
2	Instrument	JNM-ECA600
3	Solvent	CHLOROFORM-D
4	Temperature	24.4
5	Number of Scans	1024
6	Spectrometer Frequency	150.16
7	Spectral Width	37594.0
8	Lowest Frequency	-3780.2
9	Nucleus	13C
10	Digital Resolution	0.72

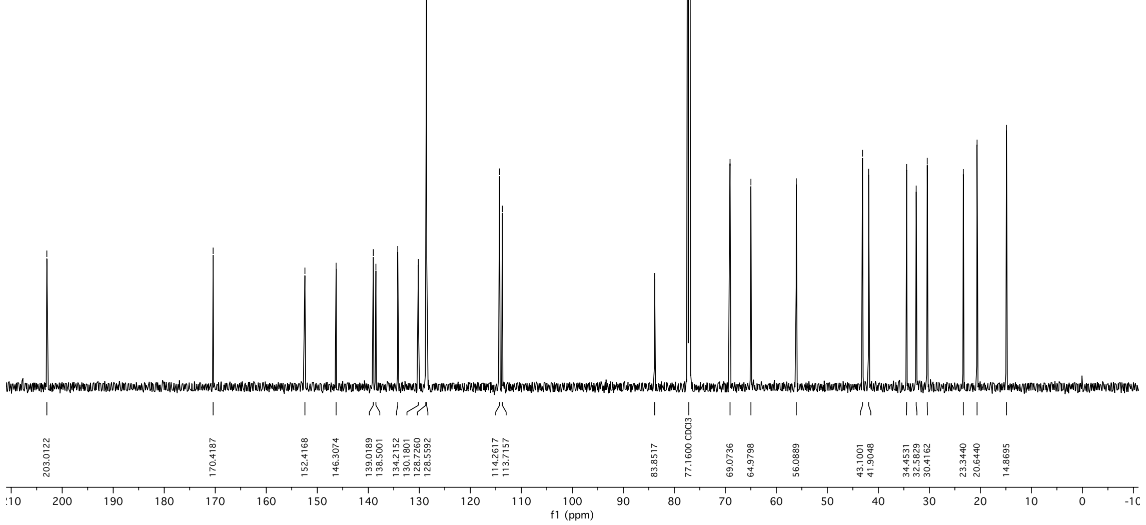

^{13}C NMR spectrum of compound **S6**

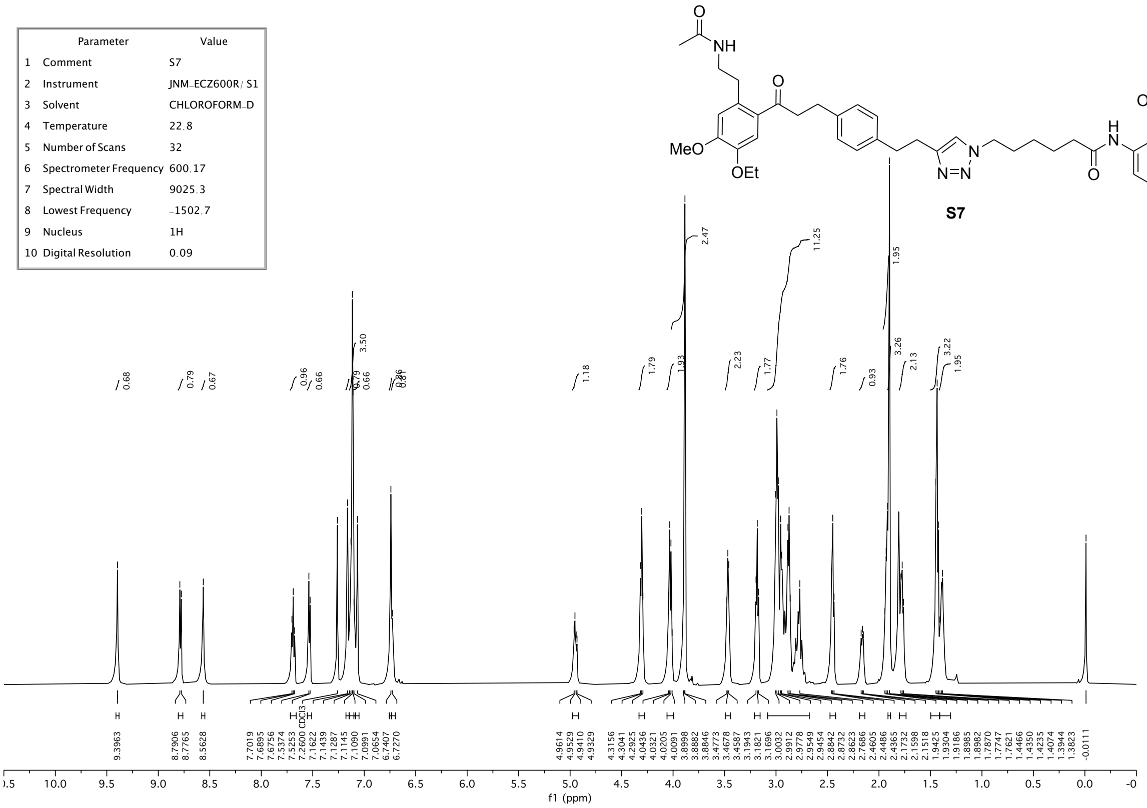

^{1}H NMR spectrum of compound **S7**

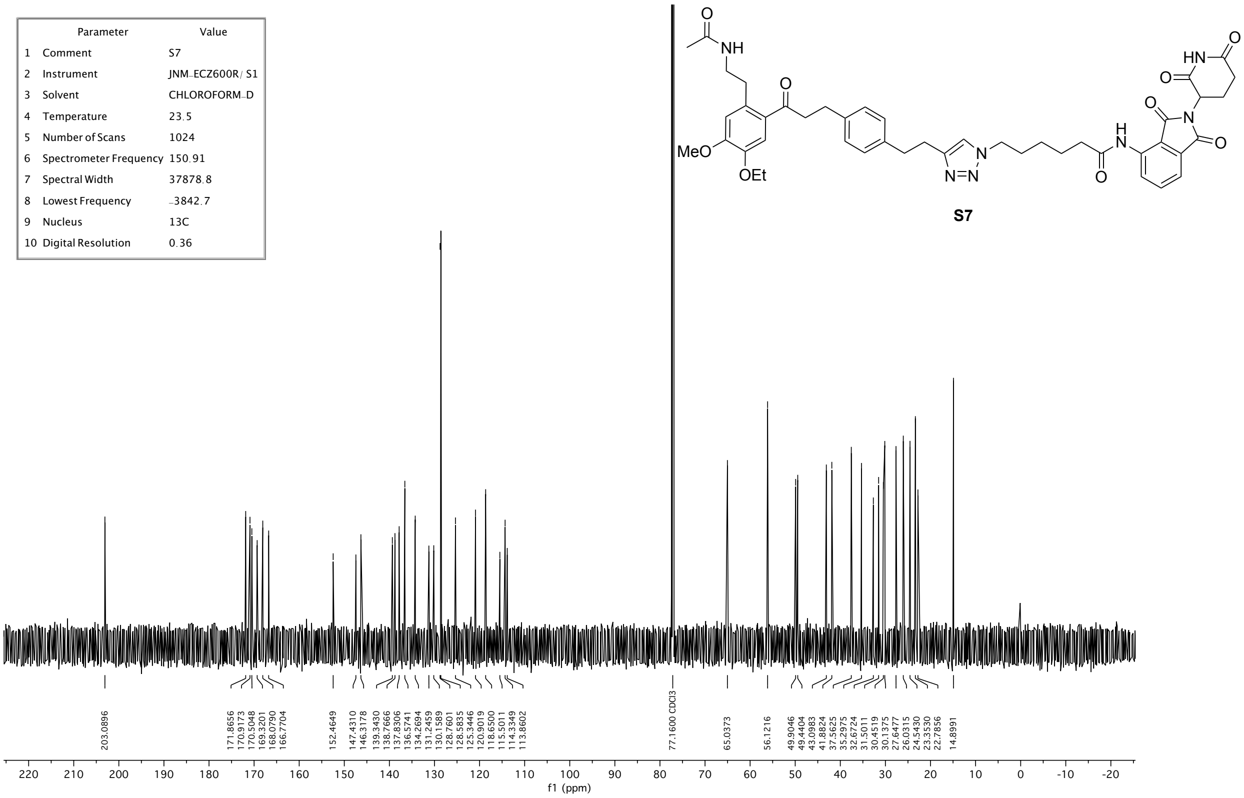

^{13}C NMR spectrum of compound **S7**

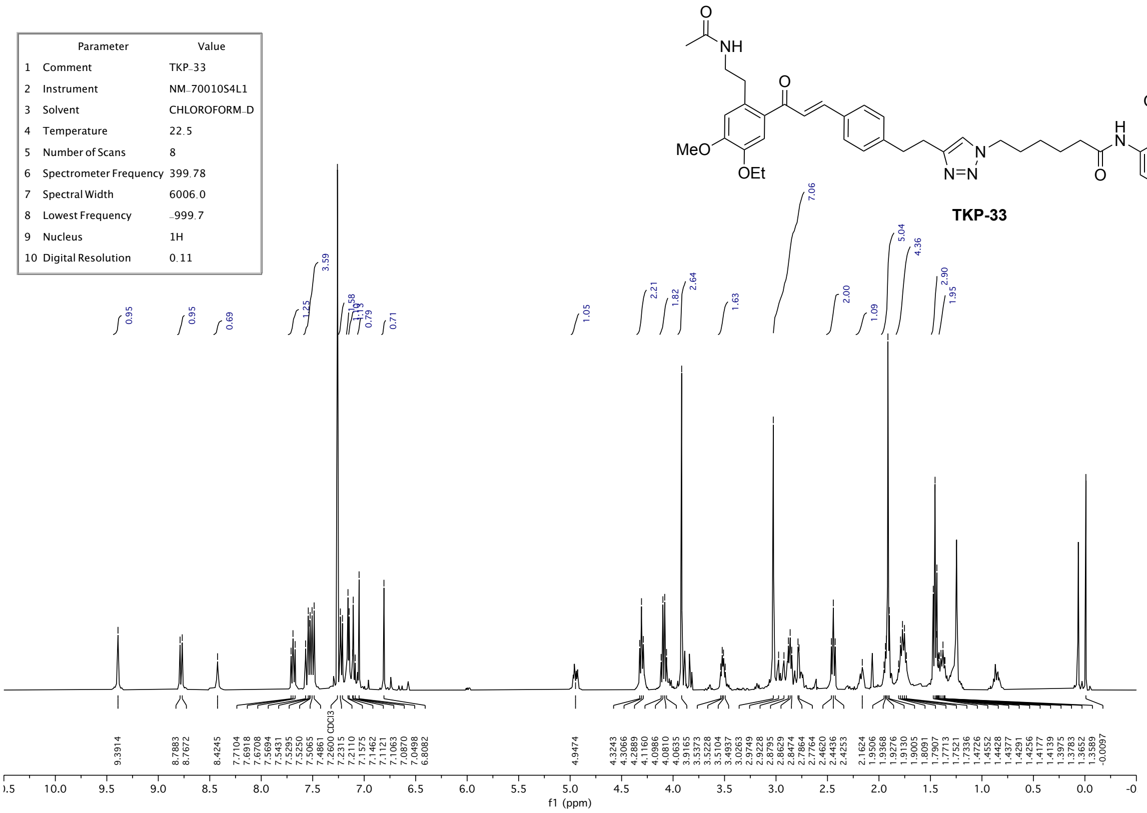

	Parameter	Value
1	Comment	TKP-33
2	Instrument	NM-70010S4L1
3	Solvent	CHLOROFORM-D
4	Temperature	22.5
5	Number of Scans	8
6	Spectrometer Frequency	399.78
7	Spectral Width	6006.0
8	Lowest Frequency	-999.7
9	Nucleus	1H
10	Digital Resolution	0.11

TKP-33

^{1}H NMR spectrum of **TKP-33**

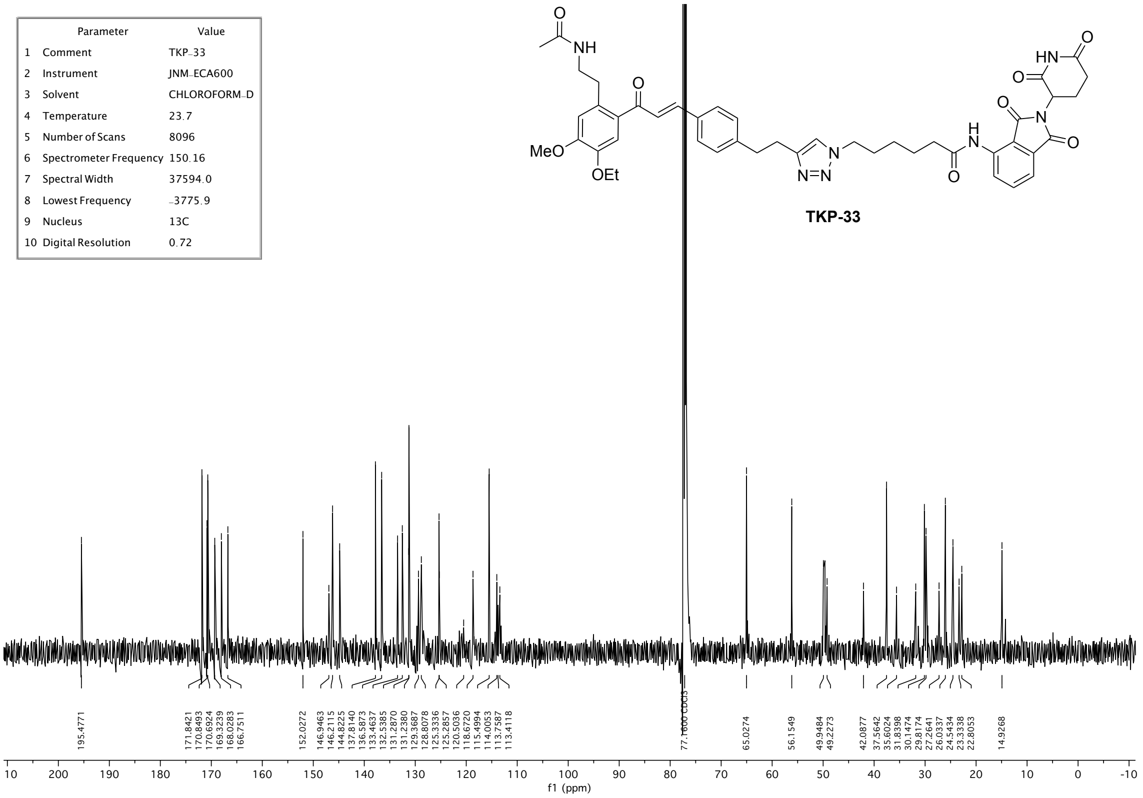

	Parameter	Value
1	Comment	TKP-33
2	Instrument	JNM-ECA600
3	Solvent	CHLOROFORM-D
4	Temperature	23.7
5	Number of Scans	8096
6	Spectrometer Frequency	150.16
7	Spectral Width	37594.0
8	Lowest Frequency	-3775.9
9	Nucleus	13C
10	Digital Resolution	0.72

TKP-33

^{13}C NMR spectrum of **TKP-33**
